# Supplementary material for: Remote-carbonyl-directed sequential Heck/isomerization/C(sp2)–H arylation of alkenes for modular synthesis of stereodefined tetrasubstituted olefins
Source: Nat Commun. 2024 Feb 26;15:1723. doi: 10.1038/s41467-024-46051-y (PMC10897343; doi:10.1038/s41467-024-46051-y)
Supplement: Supplementary file 1 — Supplementary Information [file 41467_2024_46051_MOESM1_ESM.pdf]

Supplementary Information

Remote-Carbonyl-Directed Sequential Heck/Isomerization/C(sp^2)–H Arylation of Alkenes for Modular Synthesis of Stereodefined Tetrasubstituted Olefins

Runze Luan[1,2,3], Ping Lin[1,2], Kun Li[1,2,4], Yu Du[1,2,3,4]* and Weiping Su[1,2,3,4]*

[1] State Key Laboratory of Structural Chemistry, Fujian Institute of Research on the Structure of Matter, Chinese Academy of Sciences, 155 Yangqiao Road West, Fuzhou, Fujian 350002, P. R. China.

[2] Fujian Science & Technology Innovation Laboratory for Optoelectronic Information of China, Fuzhou, Fujian 350108, P. R. China.

[3] University of Chinese Academy of Sciences, Beijing, 100049, P. R. China.

[4] College of Chemistry & Materials Science, Fujian Normal University, Fuzhou 350007, P. R. China.

E-mail: duyu@fjirsm.ac.cn (Y. Du), wpsu@fjirsm.ac.cn (W. Su)

Table of Contents

1. Supplementary Methods 3
1.1. General Information 3
1.2. Synthesis of Starting Materials 4
1.3. Optimization of the Reaction Conditions 11
1.3.1. Optimization of the Mono-Arylation Conditions 11
1.3.2. Optimization of the β-Diarylation Conditions 15
2. Supplementary Discussion 20
2.1. General Procedures and Product Characterization 20
2.1.1. General Procedure A and Product Characterization of Mono-Arylation 20
2.1.2. General Procedure B and Product Characterization of β-Diarylation 32
2.1.3. General Procedure C and Product Characterization of Stepwise Double Arylation 46
2.1.4. Synthetic Procedures and Product Characterization of By-Products. 60
2.2. Control Experiments for Mechanistic Study 62
2.2.1. Parallel Model Reactions Employing a Smaller Amount of Aryl Iodide **2a** 62
2.2.2. *E*/*Z* Isomerization of the Internal Alkene **3b** 64
2.2.3. The Second Arylation of the Mixed Isomers **3b** 67
2.2.4. Deuterium KIE Studies 68
2.3. Synthetic Applications 76
2.3.1. Gram-Scale Experiments 76
2.3.2. Photophysical Properties of **4s** 78
3. Supplementary Tables and Figures 80
3.1. X-ray Data 80
3.2. NMR Spectra 85
4. Supplementary References 181

1. Supplementary Methods

1.1. General Information

Unless otherwise noted, all reactions were performed in Schlenk tubes under an atmosphere of air with dry solvents, and checked for completion by TLC analysis and plates were visualized with short-wave UV light (254 nm). For reactions that require heating, oil bath or hotplate was used as the heat source. Chemical reagents were purchased from commercial supplies (Accela, Acros Organics, Adamas-beta®, Alfa Aesar, Aladdin, Bidepharmatech, Energy Chemical, TCI Chemicals, Innochem, J&K Chemicals, Laajoo, Leyan, Sigma-Aldrich, Sinocompound, and 3A Chemicals) and used directly without further purification. Flash chromatography was performed with Sepaflash columns produced by Santai Technologies. NMR spectra were recorded at 20 ºC (293 K) on Bruker-BioSpin AVANCE III HD 400, AVANCE III HD 500, JEOL JNM-ECZ400S and JEOL JNM-ECZ600R NMR spectrometers using $CDCl_3$ and D_2O as solutions. Chemical shifts are reported in parts per million (δ value) calibrated against the residual solvent peak. Signal patterns are indicated as follows: s, singlet; d, doublet; t, triplet; q, quartet; quin, quintet; hept, heptet; m, multiplet. Coupling constants (*J*) are given in hertz (Hz). High-resolution mass spectra were recorded on a Bruker Impact II UHR TOF LC/MS Mass Spectrometry (Operation Mode: ESI Positive Ion Mode or ESI Negative Ion Mode) and Thermo Scientific Q Exactive HF Orbitrap-FTMS (National Center for Organic Mass Spectrometry in Shanghai, Shanghai Institute of Organic Chemistry, Chinese Academic of Sciences. Operation Mode: AP-MALDI Positive Ion Mode).

1.2. Synthesis of Starting Materials

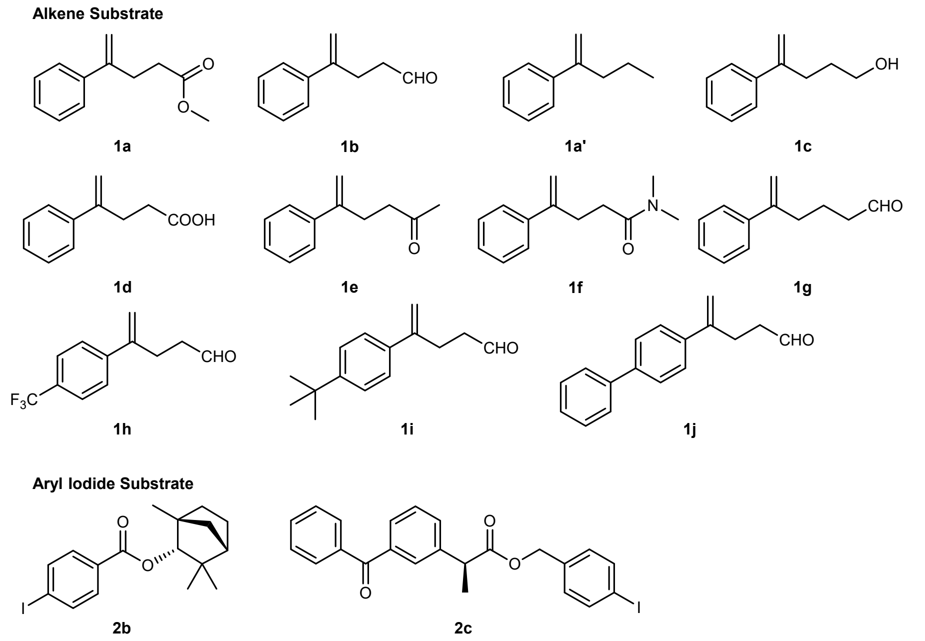

Supplementary Figure 1. The starting materials used in experiments

Synthesis of **1b**, **1c**, **1g**, **1h**, **1i**, **1j**

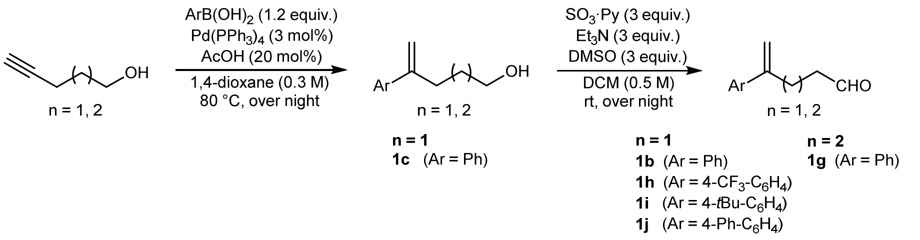

These compounds were prepared according to reported literature procedures[1-3]. The spectroscopic properties were consistent with the data available in literatures (**1c**[1], **1b**[2], **1g**[3], **1h**[4], **1i**[5]).

General procedure I: To an oven-dried 100 mL round bottom flask with previously placed magnetic stir-bar were added $Pd(PPh_3)_4$ (173 mg, 0.15 mmol, 0.03 equiv.), $ArB(OH)_2$ (6.0 mmol, 1.2 equiv.), followed by addition of dry 1,4-dioxane (50 mL) and alkyne substrate (5 mmol, 1.0 equiv.). The resulting mixture was treated with AcOH (30 μL, 0.5 mmol, 0.1 equiv.). The mixture was stirred for 10 mins at room temperature and for 10 hours at 80 °C. After completion of the reaction, the resultant solution was filtered through a short pad of 1:1 mixture of Celite and silica gel. The solvent was removed under reduced pressure, and the residue was purified by flash column chromatography on silica gel to afford the desired product.

1c was prepared according to the General procedure I. After purified by flash chromatography on silica gel (eluent = PE/EA= 5:1), the colorless oil product was obtained in 81% yield (665 mg, 4.1 mmol). **^{1}H NMR** (500 MHz, CD_3Cl): δ 7.45–7.39 (m, 2H), 7.37–7.30 (m, 2H), 7.31–7.24 (m, 1H), 5.31 (d, J = 1.4 Hz, 1H), 5.11 (q, J = 1.4 Hz, 1H), 3.66 (t, J = 6.5 Hz, 2H), 2.61 (td, J = 7.5, 1.3 Hz, 2H), 1.78–1.68 (m, 2H), 1.62 (brs, 1H); **^{13}C NMR** (126 MHz, CD_3Cl): δ 147.9, 140.9, 128.3, 127.4, 126.0, 112.5, 62.3, 31.5, 31.1.

General procedure II: Alkenyl alcohol (18.4 mmol, 1.0 equiv.), Et_3N (7.7 mL, 55 mmol, 3.0 equiv.) and DMSO (3.9 mL, 55 mmol, 3.0 equiv.) were dissolved in dry DCM (40 mL). Sulfur trioxide pyridine complex (8.79 g, 55.2 mmol) was added at 0 °C. The reaction mixture was allowed to warm to room temperature, and stirred for 12 hours. After quenching with cold water, the organic layer was extracted with DCM and dried over anhydrous Na_2SO_4, filtered, and concentrated to afford a crude oil. The residue was purified by flash column chromatography on silica gel to afford the desired product.

1b was prepared according to the General procedure II. After purified by flash chromatography on silica gel (eluent = PE/EA = 20:1), the colorless oil product was obtained in 63% yield (1.85 g, 11.6 mmol). **^{1}H NMR** (400 MHz, $CDCl_3$): δ 9.79 (t, J = 1.5 Hz, 1H), 7.41–7.27 (m, 5H), 5.33 (s, 1H), 5.10 (d, J = 1.2 Hz, 1H), 2.85 (t, J = 7.5 Hz, 2H), 2.61 (ddd, J = 8.7, 6.9, 1.5 Hz, 2H); **^{13}C NMR** (101 MHz, $CDCl_3$): δ 201.8, 146.6, 140.4, 128.5, 127.7, 126.1, 113.1, 42.4, 27.7.

1g was prepared according to the General procedure II. After purified by flash chromatography on silica gel (eluent = PE/EA = 20:1), the colorless oil product was obtained in 60% yield (1.92 g, 11.0 mmol). **^{1}H NMR** (400 MHz, $CDCl_3$): δ 9.75 (t, J = 1.6 Hz, 1H), 7.44–7.26 (m, 5H), 5.31 (s, J = 1.4 Hz, 1H), 5.05 (s, 1H), 2.57 (td, J = 7.4, 1.3 Hz, 2H), 2.46 (td, J = 7.3, 1.6 Hz, 2H), 1.80 (m, 2H); **^{13}C NMR** (101 MHz, $CDCl_3$): δ 202.5, 147.5, 140.8, 128.5, 127.7, 126.2, 113.3, 43.3, 34.6, 20.7.

1h was prepared according to the General procedure II. After purified by flash chromatography on silica gel (eluent = PE/EA= 20:1), the colorless oil product was obtained in 53% yield (2.2 g, 9.75 mmol). **^{1}H NMR** (400 MHz, $CDCl_3$): δ 9.80 (t, J = 1.3 Hz, 1H), 7.62–7.57 (m, 2H), 7.51–7.47 (m, 2H), 5.39 (d, J = 0.4 Hz, 1H), 5.20 (d,

J = 0.7 Hz, 1H), 2.86 (t, J = 7.5 Hz, 2H), 2.66–2.59 (m, 2H); **^{13}C NMR** (101 MHz, $CDCl_3$): δ 201.2, 145.6, 144.1, 129.8 (q, J = 32.5 Hz), 126.4, 125.4 (q, J = 3.7 Hz), 122.1 (d, J = 270.8 Hz), 115.0, 42.2, 27.4; **^{19}F NMR** (376 MHz, $CDCl_3$): δ –57.8.

1i was prepared according to the General procedure II. After purified by flash chromatography on silica gel (eluent = PE/EA = 20:1), the colorless oil product was obtained in 55% yield (2.1 g, 10.1 mmol). **^{1}H NMR** (400 MHz, $CDCl_3$): δ 9.82(s, 1H), 7.40 (d, J = 7.8 Hz, 2H), 7.37 (d, J = 8.8 Hz, 2H), 5.36 (s, 1H), 5.09 (s, 1H), 2.88 (t, J = 7.6 Hz, 2H), 2.65 (m, 2H), 1.36 (s, 9H); **^{13}C NMR** (101MHz, $CDCl_3$): δ 202.4, 151.2, 146.7, 137.7, 126.1, 125.8, 112.7, 42.9, 34.9, 31.7, 28.0.

1j was prepared according to the General procedure II. After purified by flash chromatography on silica gel (eluent = PE/EA = 10:1), the light-yellow solid product was obtained in 61% yield. **^{1}H NMR** (400 MHz, $CDCl_3$): δ 9.75 (s, 1H), 7.58 (d, J = 7.8 Hz, 2H), 7.55 (d, J = 7.8 Hz, 2H), 7.45 (d, J = 7.7 Hz, 2H), 7.41 (d, J = 7.7 Hz, 2H), 7.32 (t, J = 7.3 Hz, 1H), 5.38 (s, 1H), 5.09 (s, 1H), 2.85 (t, J = 7.5 Hz, 2H), 2.60 (t, J = 7.6 Hz, 2H); **^{13}C NMR** (101 MHz, $CDCl_3$): δ 201.85, 146.09, 140.60, 140.58, 139.27, 128.90, 127.47, 127.21, 127.04, 126.53, 113.14, 42.49, 27.58; **HRMS** (ESI) *m/z* $[M+Na]^+$ Calcd for $C_{17}H_{16}NaO$: 259.1093, found: 259.1094.

Synthesis of **1a'**, **1d**

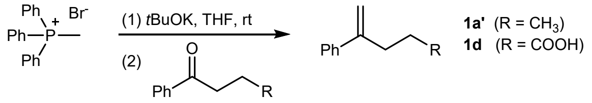

These compounds were prepared according to reported literature procedures (**1a'**[6], **1d**[7]). The spectroscopic properties were consistent with the data available in literatures.

To a suspension of *t*-BuOK (1.35 g, 12 mmol, 1.2 equiv.) in anhydrous THF (20 mL) was added $MePPh_3Br$ (4.28 g, 12 mmol, 1.2 equiv.) under argon atmosphere. The suspension was stirred at room temperature for 1 hr, and then 2-cyclopentyl-1-(*p*-tolyl)ethanone (2.02 g, 10 mmol) was added. The resulting mixture was stirred at same temperature for 1 hr. After quenching with H_2O, the organic layer was extracted with EA and dried over anhydrous Na_2SO_4, filtered, and concentrated to afford a crude oil. Purification by column chromatography on silica

gel (eluent = PE) to afford the desired colorless oil product **1a'** (1.31 g, 9 mmol, 90% yield). **^{1}H NMR** (400 MHz, $CDCl_3$): δ 7.64–7.57 (m, 2H), 7.53–7.47 (m, 2H), 7.47–7.39 (m, 1H), 5.48 (d, *J* = 1.3 Hz, 1H), 5.26 (d, *J* = 1.3 Hz, 1H), 2.68 (t, *J* = 7.5 Hz, 2H), 1.74–1.60 (m, 2H), 1.13 (t, *J* = 7.4 Hz, 3H); **^{13}C NMR** (101 MHz, $CDCl_3$): δ 148.6, 141.6, 128.3, 127.3, 126.3, 112.3, 37.6, 21.5, 13.9.

To a suspension of *t*-BuOK (24.56 g, 218.9 mmol, 2.6 equiv.) in anhydrous THF (200 mL) was added $MePPh_3Br$ (39.1 g, 109.5 mmol, 1.3 equiv.). The mixture was stirred at room temperature for 0.5 hr. 3-Benzoylpropionic acid (15.0 g, 84.2 mmol) was added and the reaction was stirred at room temperature overnight. Solvent was removed in vacuo, the residue was diluted with DCM (100 mL) and washed with aqueous NaOH (1 M, 100 mL). The aqueous layer was separated, washed with DCM (50 mL) and acidified to pH = 1 with concentrated HCl. **1d** was obtained as a light-yellow solid (13.6 g, 77.46 mmol, 92% yield) via Büchner filtration. **^{1}H NMR** ($CDCl_3$, 500 MHz): δ 7.43 (d, *J* = 8.0 Hz, 2H), 7.36 (dd, *J* = 8.0, 7.2 Hz, 2H), 7.30 (t, *J* = 7.2 Hz, 1H), 5.35 (s, 1H), 5.13 (s, 1H), 2.87 (t, *J* = 7.6 Hz, 2H), 2.58 (t, *J* = 7.6 Hz, 2H); **^{13}C NMR** ($CDCl_3$, 125 MHz): δ 179.9, 146.5, 140.3, 128.4, 127.8, 126.0, 112.9, 33.0, 30.1.

Synthesis of methyl 4-phenylpent-4-enoate (**1a**)

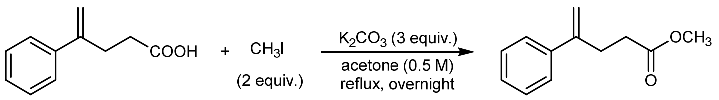

4-phenylpent-4-enoic acid (2.5 g, 14 mmol) and K_2CO_3 (5.8 g, 42 mmol, 3.0 equiv.) were placed in a 100 mL round bottom flask. Acetone (28 mL) and iodomethane (1.8 mL, 28 mmol, 2.0 equiv.) were added dropwise, and the mixture was heated at 70 ºC for 10 hours. After cooling to room temperature, 20 mL water was added and the mixture was extracted 3 times with EtOAc (20 mL × 3), the combined organic layers were dried over anhydrous Na_2SO_4 and filtered, concentrated under reduced pressure. The residue was purified by flash chromatography on a silica gel (PE:EA = 10:1) to provide methyl 4-phenylpent-4-enoate **1a** (2.3 g, 11.2 mmol) in 86% yield. The spectroscopic properties were consistent with the data available in the literature[8]. **^{1}H NMR** (400 MHz, $CDCl_3$): δ 7.26–7.42 (m, 5H), 5.31 (s, 1H), 5.10 (s, 1 H), 3.66 (s, 3H), 2.84 (t, *J* = 7.8 Hz, 2H), 2.45–2.53 (m, 2 H); **^{13}C NMR** (101 MHz, $CDCl_3$): δ 173.7, 147.0, 140.7, 128.5, 127.8, 126.2, 113.0, 51.7, 33.2, 30.6.

Synthesis of 5-phenylhex-5-en-2-one (**1e**)

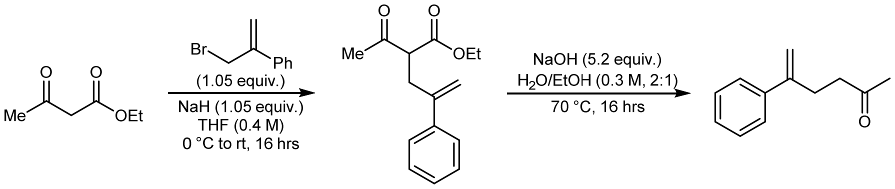

This compound was prepared according to reported literature procedure[9]. The spectroscopic properties were consistent with the data available in the literature[10].

The NaH 60% dispersion in mineral oil (966 mg, 24.2 mmol, 1.05 equiv.) was dispensed into a flame-dried round bottom flask, which was subsequently purged with N_2. The NaH was suspended in dry THF (0.4 mmol/mL) and the stirred suspension was cooled to 0 °C in an ice bath. At 0 °C, ethyl acetoacetate (3 g, 23.0 mmol) was added dropwise with stirring. The solution was stirred at 0 °C for 30 minutes after effervescence ceased. At 0 °C, (3-bromoprop-1-en-2-yl)benzene (4.77 g, 24.2 mmol, 1.05 equiv.) was added to the mixture dropwise. The reaction was allowed to warm to room temperature and stirred for 16 hours overnight. The reaction was then quenched with concentrated NH_4Cl solution and diluted with water and diethyl ether. The organic layer was separated and aqueous layer extracted three times with diethyl ether in a separatory funnel. The combined organic layers were washed with brine, dried with anhydrous Na_2SO_4, filtered, and concentrated to afford a crude oil, which was taken on to the next step without further purification. Decarboxylation of the crude alkylation product was carried out as follows: NaOH (4.78 g, 119.6 mmol, 5.2 equiv.) was weighed into a round bottom flask containing a 2:1 water/ethanol mixture (approx. concentration 0.3 mmol/mL) and stirred to dissolution. At room temperature the crude alkylation product was introduced dropwise, the reaction was stirred at 70 °C overnight. The reaction was cooled to room temperature and quenched with concentrated NH_4Cl. The organic phase was separated and the aqueous phase extracted three times with diethyl ether. The combined organic layers were washed with brine, dried with anhydrous Na_2SO_4, filtered, and concentrated to afford a crude oil. Purification by column chromatography on silica gel (PE/EA = 90:5) to afford the desired colorless oil product **1e** (1.48 g, 8.51 mmol, 37% yield). **^{1}H NMR** (400 MHz, $CDCl_3$): δ 7.40–7.27 (m, 5H), 5.28 (s, 1H), 5.06 (d, J = 1.2 Hz, 1H), 2.79 (t, J = 7.6 Hz, 2H), 2.60–2.56 (m, 2H), 2.12 (s, 3H); **^{13}C NMR** (100 MHz, $CDCl_3$): δ 208.1, 147.2, 140.6, 128.4, 127.6, 126.1, 112.8, 42.4, 30.0, 29.3.

Synthesis of *N*,*N*-dimethyl-4-phenylpent-4-enamide (**1f**)

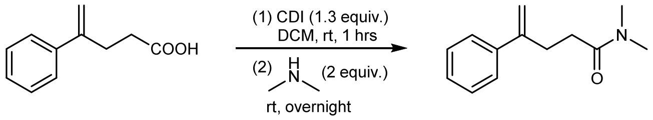

This compound was prepared according to reported literature procedure[11].

To an oven-dried 250 mL round bottom flask with previously placed magnetic stir-bar were added 4-phenylpent-4-enoic acid (3.0 g, 17 mmol) and dry DCM (100 mL) under N_2 atmosphere. 1,1'-carbonyldiimidazole (CDI) (3.58 g, 22.1 mmol, 1.3 equiv.) was added slowly and the mixture was stirred for 1 hour. Then dimethylamine (1.73 mL, 34 mmol, 2 equiv.) were added dropwise and the reaction was stirred overnight. The reaction mixture was poured into a separatory funnel containing DCM and 10% HCl. The organic layers were collected and extracted again with saturated sodium bicarbonate. The organic layers were washed with brine, dried over anhydrous Na_2SO_4 and concentrated in vacuo to furnish crude product. Purification by column chromatography on silica gel (PE/EA = 2:1) to afford the desired light-yellow oil product **1f** (1.87 g, 9.2 mmol, 54% yield). **^{1}H NMR** (400 MHz, $CDCl_3$): δ 7.44–7.38 (m, 2H), 7.35–7.28 (m, 2H), 7.28–7.23 (m, 1H), 5.29 (s, 1H), 5.09 (s, 1H), 2.91 (s, 3H), 2.89 (s, 3H), 2.88–2.83 (m, 2H), 2.46–2.39 (m, 2H); **^{13}C NMR** (101 MHz, $CDCl_3$): δ 172.48, 147.63, 140.63, 128.50, 127.66, 126.18, 112.87, 37.28, 35.51, 32.36, 30.85.

Synthesis of **2b**, **2c**

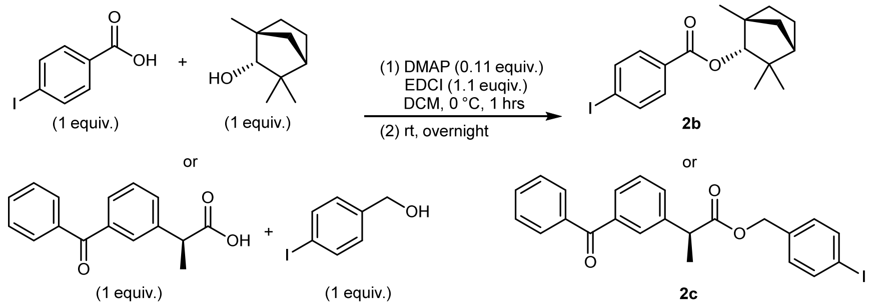

This compound was prepared according to reported literature procedure[12,13]. The spectroscopic properties were consistent with the data available in the literature[12].

General procedure III: The Acid (3.0 mmol, 1.0 equiv.), hydroxyl compound (3.0 mmol, 1.0 equiv.), DMAP (0.33 mmol, 0.11 equiv.) and EDCI (3.3 mmol, 1.1 equiv.) were dissolved in DCM (30 mL) and stirred at 0 °C for 1 hour. Then the reaction was stirred at room temperature overnight. Upon completion, the reaction mixture was

washed with 60 mL H_2O, then the aqueous layer was extracted with DCM (30 mL) for 3 times. The organic layers were combined, dried over anhydrous Na_2SO_4, and concentrated under vacuum. The residue was purified by flash column chromatography on silica gel to afford the desired product.

2b was prepared according to the General procedure III, using 4-iodobenzoic acid (3.0 mmol, 744 mg), (+)-Fenchol (3.0 mmol, 463 mg), the target compound was obtained as a white solid (934 mg, 81%). **^{1}H NMR** (400 MHz, CD_3Cl): δ 7.77 (d, *J* = 8.4 Hz, 2H), 7.81 (d, *J* = 8.4 Hz, 2H), 4.61 (s, 1H), 1.93–1.86 (m, 1H), 1.81–1.73 (m, 2H), 1.66 (d, *J* = 10.2 Hz, 1H), 1.56–1.46 (m, 1H), 1.30–1.20 (m, 2H), 1.18 (s, 3H), 1.10 (s, 3H), 0.82 (s, 3H); **^{13}C NMR** (101 MHz, $CDCl_3$): δ 166.4, 137.8, 131.0, 130.2, 100.6, 87.0, 48.6, 48.4, 41.5, 39.9, 29.8, 26.9, 25.9, 20.3, 19.5.

2c was prepared according to the General procedure III, using 2-(3-benzoylphenyl)propanoic acid (3 mmol, 763 mg), (4-iodophenyl)methanol (3 mmol, 702 mg), the target compound was obtained as a colorless oil (1.16 g, 82%). **^{1}H NMR** (400 MHz, CD_3Cl): δ 7.76 (m, 3H), 7.66 (d, *J* = 7.6 Hz, 1H), 7.59 (d, *J* = 8.3 Hz, 2H), 7.55 (d, *J* = 7.5 Hz, 1H), 7.51 (d, *J* = 7.9 Hz, 1H), 7.43 (m, 3H), 6.95 (d, *J* = 8.1 Hz, 2H), 5.03 (s, 2H), 3.84 (q, *J* = 7.1 Hz, 1H), 1.54 (d, *J* = 7.2 Hz, 3H); **^{13}C NMR** (101 MHz, $CDCl_3$): δ 196.3, 173.7, 140.6, 138.0, 137.6, 137.5, 135.5, 132.6, 131.6, 130.1, 129.8, 129.2, 129.1, 128.6, 128.4, 94.0, 65.9, 45.4, 18.4.

1.3. Optimization of the Reaction Conditions

1.3.1. Optimization of the Mono-Arylation Conditions

Supplementary Table 1. Optimization of Ligands for Mono-Arylation

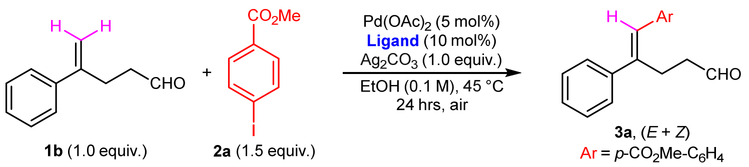

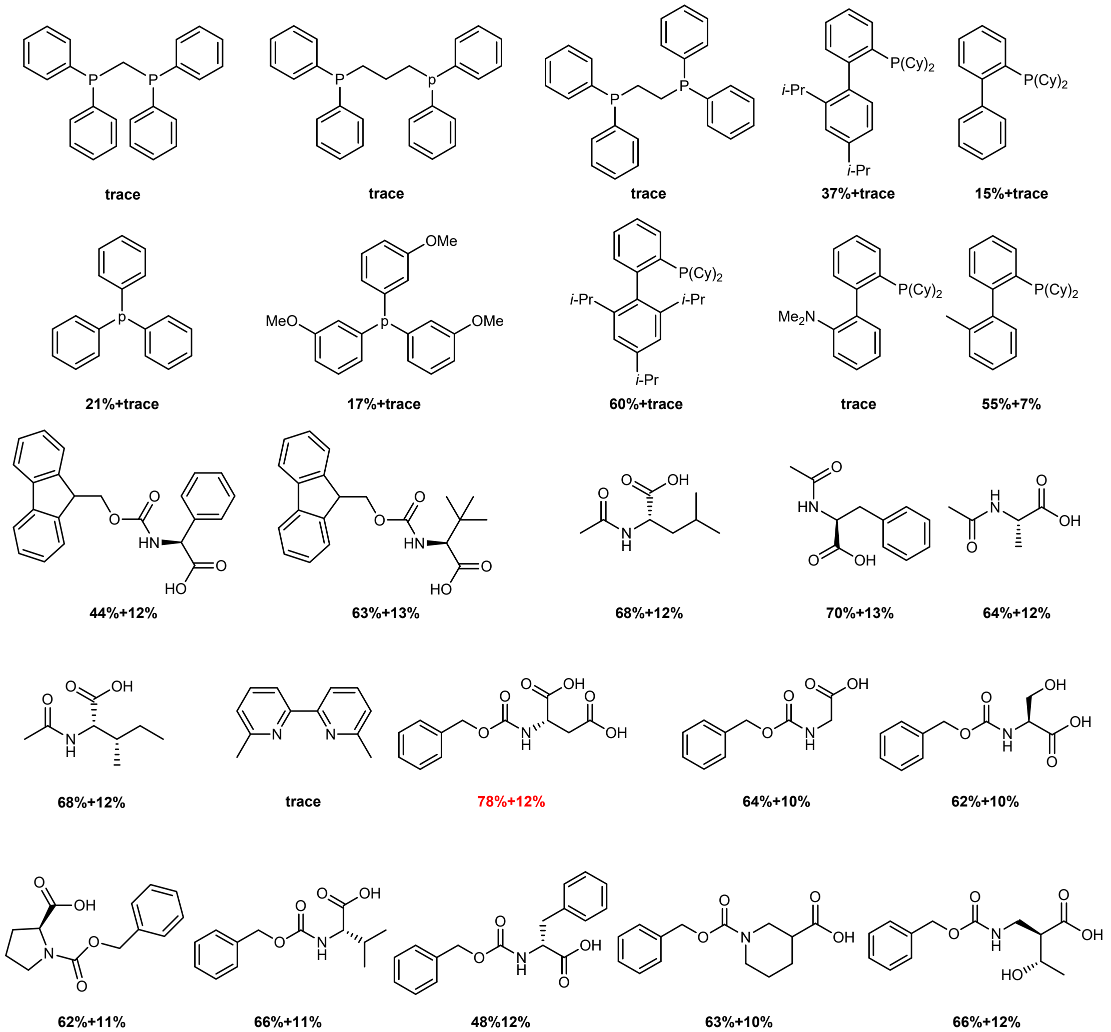

Reaction conditions: **1b** (0.2 mmol), **2a** (0.3 mmol), $Pd(OAc)_2$ (0.01 mmol), Ag_2CO_3 (0.2 mmol), Ligand (0.02 mmol), EtOH (2 mL), 45 °C, 24 hours, air. Yield is calculated based on ^{1}H NMR of the crude reaction mixture using 1,1,2,2-tetrachloroethane as the internal standard.

Supplementary Table 2. Optimization of Temperature for Mono-Arylation

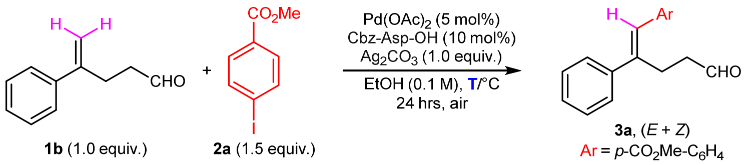

Entry	T/°C	Yield of **3a** (*E* + *Z*) (%)
1	25	82 + 10
2	70	58 + 12
3	80	47 + 10

Reaction conditions: **1b** (0.2 mmol), **2a** (0.3 mmol), $Pd(OAc)_2$ (0.01 mmol), Ag_2CO_3 (0.2 mmol), Cbz-Asp-OH (0.02 mmol), EtOH (2 mL), T/°C, 24 hours, air. Yield is calculated based on ^1H NMR of the crude reaction mixture using 1,1,2,2-tetrachloroethane as the internal standard.

Supplementary Table 3. Optimization of Ag Salts for Mono-Arylation

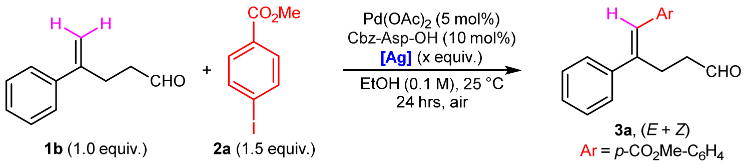

Entry	[Ag]	x equiv.	Yield of **3a** (*E* + *Z*) (%)
1	AgOAc	1	65 + 7
2	Ag_2SO_4	1	10 + trace
3	$AgSbF_6$	1	8 + trace
4	Ag_2O	1	24 + trace
5	$AgNO_3$	1	trace
6	CF_3COOAg	1	7 + trace
7	Ag_3PO_4	1	40 + trace
8	CF_3SO_3Ag	1	5 + trace
9	$C_7H_7SO_3Ag$	1	trace
10	Ag_2CO_3	1.5	72 + 7
11	Ag_2CO_3	2	67 + 6

Reaction conditions: **1b** (0.2 mmol), **2a** (0.3 mmol), $Pd(OAc)_2$ (0.01 mmol), Ag salt, Cbz-Asp-OH (0.02 mmol), EtOH (2 mL), 25 °C, 24 hours, air. Yield is calculated based on ^1H NMR of the crude reaction mixture using 1,1,2,2-tetrachloroethane as the internal standard.

Supplementary Table 4. Optimization of Palladium Catalysts for Mono-Arylation

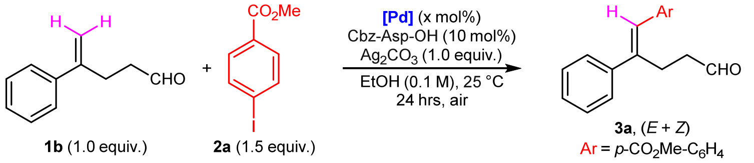

Entry	[Pd]	x mol%	Yield of **3a** (*E* + *Z*) (%)
1	$Pd(dppb)Cl_2$	5	trace
2	$Pd(MeCN)_2Cl_2$	5	73 + 15
3	$Pd(Ph_3P)_4$	5	12 + trace
4	$[Pd(\pi\text{-allyl})Cl]_2$	5	60+10
5	$Pd(MeCN)_4(BF_4)_2$	5	19 + trace
6	$Pd(CF_3COO)_2$	5	8 + trace
7	$PdCl_2$	5	71 + 10
8	$Pd(OAc)_2$	10	72 + 12

Reaction conditions: **1b** (0.2 mmol), **2a** (0.3 mmol), Pd catalyst, Ag_2CO_3 (0.2 mmol), Cbz-Asp-OH (0.02 mmol), EtOH (2 mL), 25 °C, 24 hours, air. Yield is calculated based on ^1H NMR of the crude reaction mixture using 1,1,2,2-tetrachloroethane as the internal standard.

Supplementary Table 5. Optimization of Solvents for Mono-Arylation

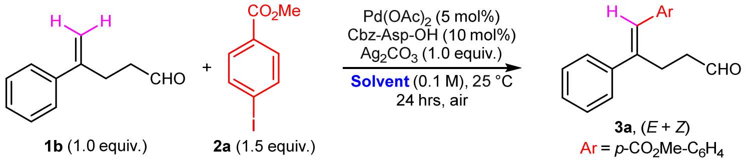

Entry	Solvent	Yield of **3a** (*E* + *Z*) (%)
1	toluene	22 + trace
2	DCM	40 + trace
3	DMF	72 + trace
4	THF	33 + trace
5	CH_3CN	24 + trace
6	CH_3OH	81 + 12
7	*i*-PrOH	69 + 7
8	*t*-BuOH	33 + trace
9	acetone	54 + trace
10	1,4-Dioxane	16 + trace

Reaction conditions: **1b** (0.2 mmol), **2a** (0.3 mmol), $Pd(OAc)_2$ (0.01 mmol), Ag_2CO_3 (0.2 mmol), Cbz-Asp-OH (0.02 mmol), Solvent (2 mL), 25 °C, 24 hours, air. Yield is calculated based on ^1H NMR of the crude reaction mixture using 1,1,2,2-tetrachloroethane as the internal standard.

Supplementary Table 6. Optimization of Substrate Equivalent for Mono-Arylation

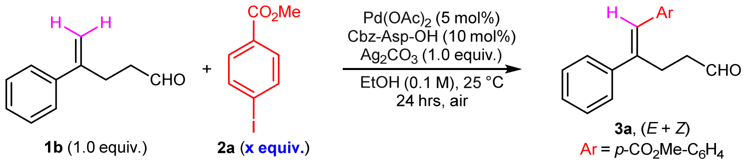

Entry	**2a** (x equiv.)	Yield of **3a** (*E* + *Z*) (%)
1	1	60 + 5
2	2	70 + 9

Reaction conditions: **1b** (0.2 mmol), **2a** (x equiv.), $Pd(OAc)_2$ (0.01 mmol), Ag_2CO_3 (0.2 mmol), Cbz-Asp-OH (0.02 mmol), EtOH (2 mL), 25 °C, 24 hours, air. Yield is calculated based on ^1H NMR of the crude reaction mixture using 1,1,2,2-tetrachloroethane as the internal standard.

Supplementary Table 7. Optimization of Solvent Volume for Mono-Arylation

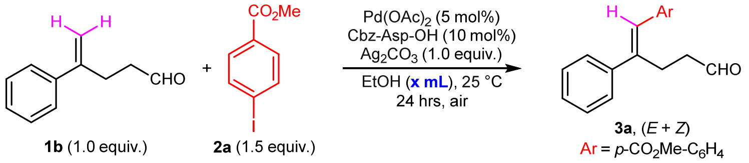

Entry	EtOH (x mL)	Yield of **3a** (*E* + *Z*) (%)
1	1	40 + 6
2	3	73 + 8
3	4	67 + 12

Reaction conditions: **1b** (0.2 mmol), **2a** (0.3 mmol), $Pd(OAc)_2$ (0.01 mmol), Ag_2CO_3 (0.2 mmol), Cbz-Asp-OH (0.02 mmol), EtOH (x mL), 25 °C, 24 hours, air. Yield is calculated based on ^1H NMR of the crude reaction mixture using 1,1,2,2-tetrachloroethane as the internal standard.

1.3.2. Optimization of the *β*-Diarylation Conditions

Supplementary Table 8. Optimization of Ligands for *β*-Diarylation

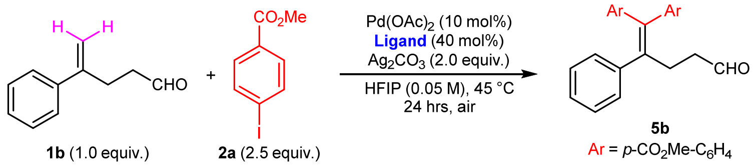

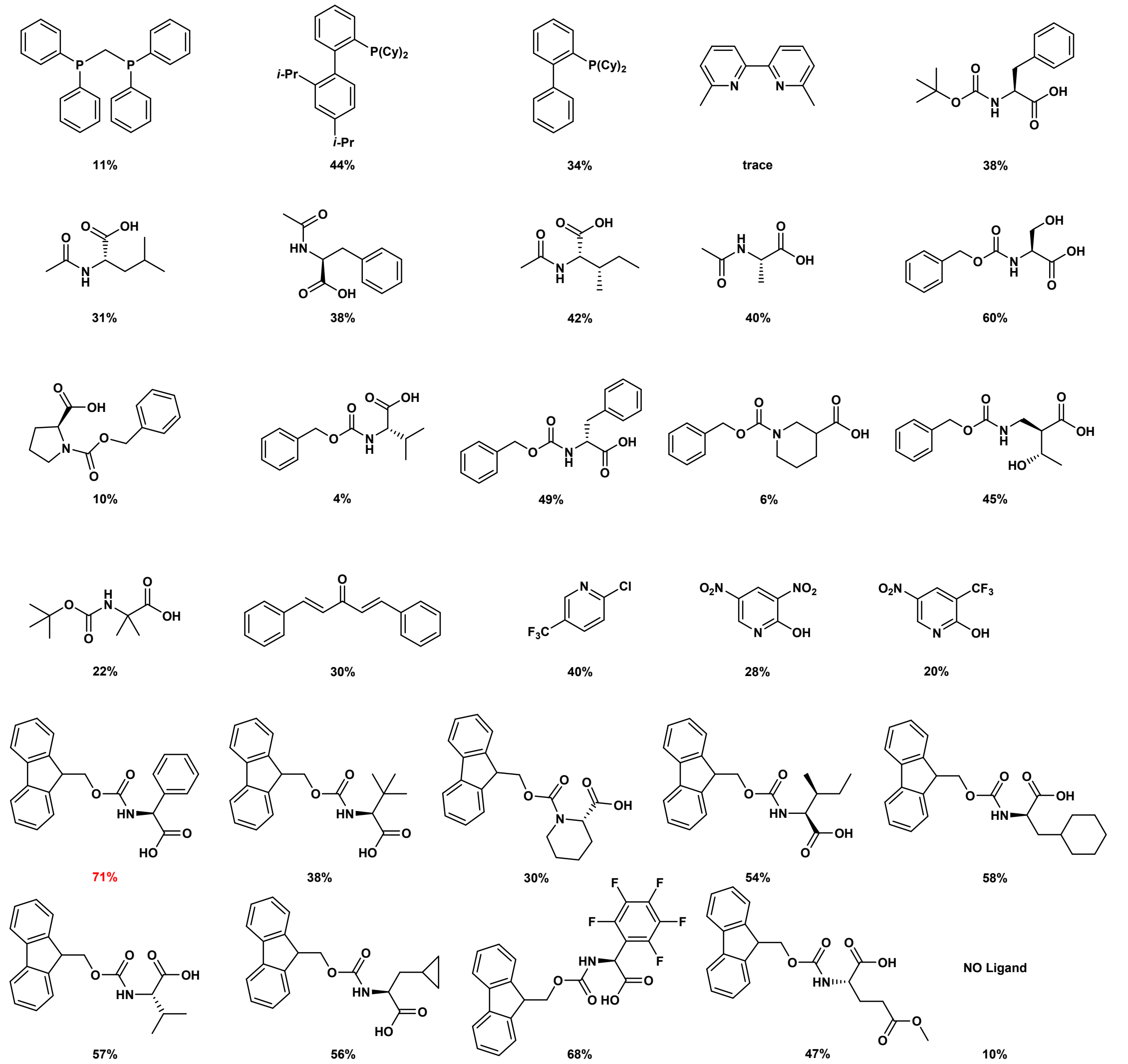

Reaction conditions: **1b** (0.1 mmol), **2a** (0.25 mmol), $Pd(OAc)_2$ (0.01 mmol), Ag_2CO_3 (0.2 mmol), Ligand (0.04 mmol), HFIP (2 mL), 45 °C, 24 hours, air. Yield is calculated based on ^{1}H NMR of the crude reaction mixture using 1,1,2,2-tetrachloroethane as the internal standard.

Supplementary Table 9. Optimization of Ag Salts for β-Diarylation

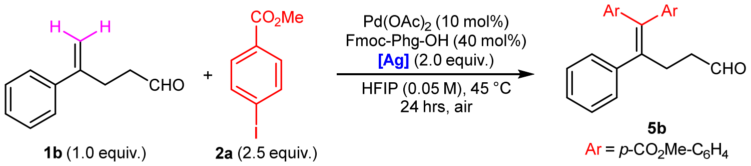

Entry	[Ag]	Yield of **5b** (%)
1	$AgNO_3$	trace
2	Ag_2O	25
3	Ag_3PO_4	16
4	$C_7H_7SO_3Ag$	7
5	CH_3SO_3Ag	11
6	AgOAc	24
7	$AgPF_6$	trace
8	$AgSbF_6$	trace
9	Ag_2SO_4	trace
10	CF_3COOAg	9

Reaction conditions: **1b** (0.1 mmol), **2a** (0.25 mmol), $Pd(OAc)_2$ (0.01 mmol), Ag salt (0.2 mmol), Fmoc-Phg-OH (0.04 mmol), HFIP (2 mL), 45 °C, 24 hours, air. Yield is calculated based on ^1H NMR of the crude reaction mixture using 1,1,2,2-tetrachloroethane as the internal standard.

Supplementary Table 10. Optimization of Palladium Catalysts for β-Diarylation

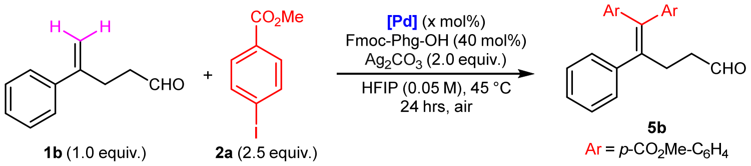

Entry	[Pd]	x mol%	Yield of **5b** (%)
1	$Pd(MeCN)_2Cl_2$	10	35
2	$Pd(Ph_3P)_4$	10	20
3	$[Pd(\pi\text{-allyl})Cl]_2$	10	trace
4	$Pd(MeCN)_4(BF_4)_2$	10	trace
5	$Pd(CF_3COO)_2$	10	19
6	$PdCl_2$	10	16
7	$Pd(OAc)_2$	5	38

Reaction conditions: **1b** (0.1 mmol), **2a** (0.25 mmol), Pd catalyst, Ag_2CO_3 (0.2 mmol), Fmoc-Phg-OH (0.04 mmol), HFIP (2 mL), 45 °C, 24 hours, air. Yield is calculated based on ^{1}H NMR of the crude reaction mixture using 1,1,2,2-tetrachloroethane as the internal standard.

Supplementary Table 11. Optimization of Additives for *β*-Diarylation

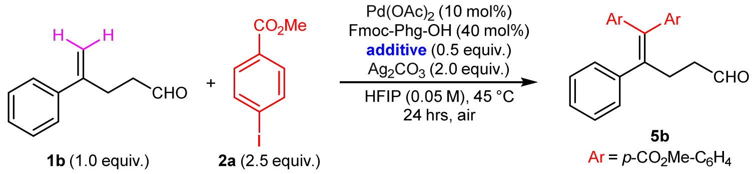

Entry	Additive	Yield of **5b** (%)
1	TBAC	64
2	Tributylamine	35
3	4-methoxybenzoborate	82
4	1,4-Benzoquinone	42
5	3,4,5-Trifluorobenzoic acid	59

Reaction conditions: **1b** (0.1 mmol), **2a** (0.25 mmol), $Pd(OAc)_2$ (0.01 mmol), Ag_2CO_3 (0.2 mmol), Fmoc-Phg-OH (0.04 mmol), additive (0.05 mmol), HFIP (2 mL), 45 °C, 24 hours, air. Yield is calculated based on ^{1}H NMR of the crude reaction mixture using 1,1,2,2-tetrachloroethane as the internal standard.

Supplementary Table 12. Optimization of Temperature for *β*-Diarylation

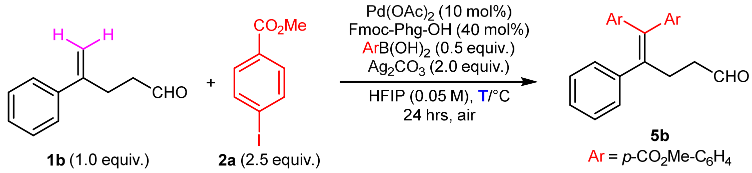

Entry	T/°C	Yield of **5b** (%)
1	25	74
2	65	61

Reaction conditions: **1b** (0.1 mmol), **2a** (0.25 mmol), $Pd(OAc)_2$ (0.01 mmol), Ag_2CO_3 (0.2 mmol), Fmoc-Phg-OH (0.04 mmol), 4-methoxybenzoborate (0.05 mmol), HFIP (2 mL), T/°C, 24 hours, air. Yield is calculated based on ^{1}H NMR of the crude reaction mixture using 1,1,2,2-tetrachloroethane as the internal standard.

Supplementary Table 13. Optimization of Ligand Equivalent for *β*-Diarylation

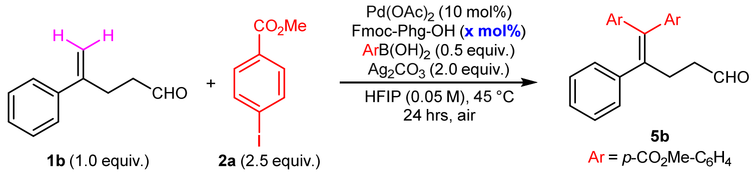

Entry	x mol%	Yield of **5b** (%)
1	20	73
2	30	82

Reaction conditions: **1b** (0.1 mmol), **2a** (0.25 mmol), $Pd(OAc)_2$ (0.01 mmol), Ag_2CO_3 (0.2 mmol), Fmoc-Phg-OH (x mol%), 4-methoxybenzoborate (0.05 mmol), HFIP (2 mL), 45 °C, 24 hours, air. Yield is calculated based on ^1H NMR of the crude reaction mixture using 1,1,2,2-tetrachloroethane as the internal standard.

Supplementary Table 14. Optimization of Substrate Equivalent for *β*-Diarylation

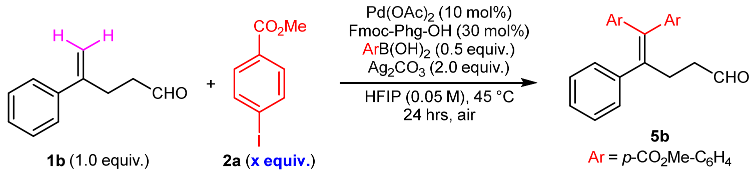

Entry	**2a** (x equiv.)	Yield of **5b** (%)
1	2	69
2	3.5	77

Reaction conditions: **1b** (0.1 mmol), **2a** (x equiv.), $Pd(OAc)_2$ (0.01 mmol), Ag_2CO_3 (0.2 mmol), Fmoc-Phg-OH (0.03 mmol), 4-methoxybenzoborate (0.05 mmol), HFIP (2 mL), 45 °C, 24 hours, air. Yield is calculated based on ^1H NMR of the crude reaction mixture using 1,1,2,2-tetrachloroethane as the internal standard.

Supplementary Table 15. Optimization of Additive Equivalent for *β*-Diarylation

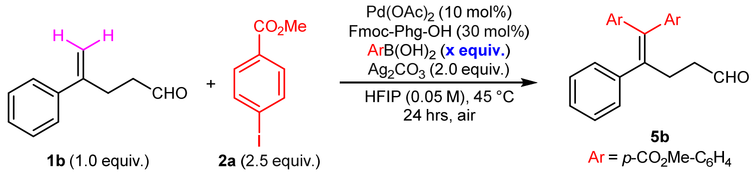

Entry	4-methoxybenzoborate (x equiv.)	Yield of **5b** (%)
1	0.2	73
2	0.8	77

Reaction conditions: **1b** (0.1 mmol), **2a** (0.25 mmol), $Pd(OAc)_2$ (0.01 mmol), Ag_2CO_3 (0.2 mmol), Fmoc-Phg-OH (0.03 mmol), 4-methoxybenzoborate (x equiv.), HFIP (2 mL), 45 °C, 24 hours, air.

Yield is calculated based on ^{1}H NMR of the crude reaction mixture using 1,1,2,2-tetrachloroethane as the internal standard.

Supplementary Table 16. Optimization of Aryl Boronic Acids for *β*-Diarylation

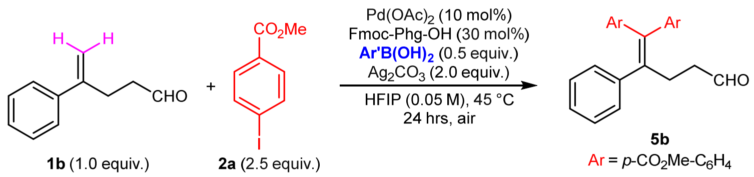

Entry	Ar’	Yield of **5b** (%)
1	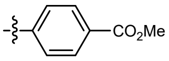 (Ar’ = Ar)	80
2	w/o Ar’B(OH)$_2$	67
3	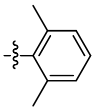	69
4	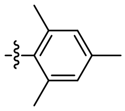	68
5	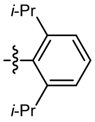	68
6	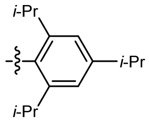	54
7	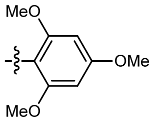	65
8	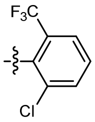	66

Reaction conditions: **1b** (0.1 mmol), **2a** (0.25 mmol), Pd(OAc)$_2$ (0.01 mmol), Ag_2CO_3 (0.2 mmol), Fmoc-Phg-OH (0.03 mmol), aryl boronic acid (0.05 mmol), HFIP (2 mL), 45 °C, 24 hours, air. Yield is calculated based on ^{1}H NMR of the crude reaction mixture using 1,1,2,2-tetrachloroethane as the internal standard, w/o = without.

2. Supplementary Discussion

2.1. General Procedures and Product Characterization

2.1.1. General Procedure A and Product Characterization of Mono-Arylation

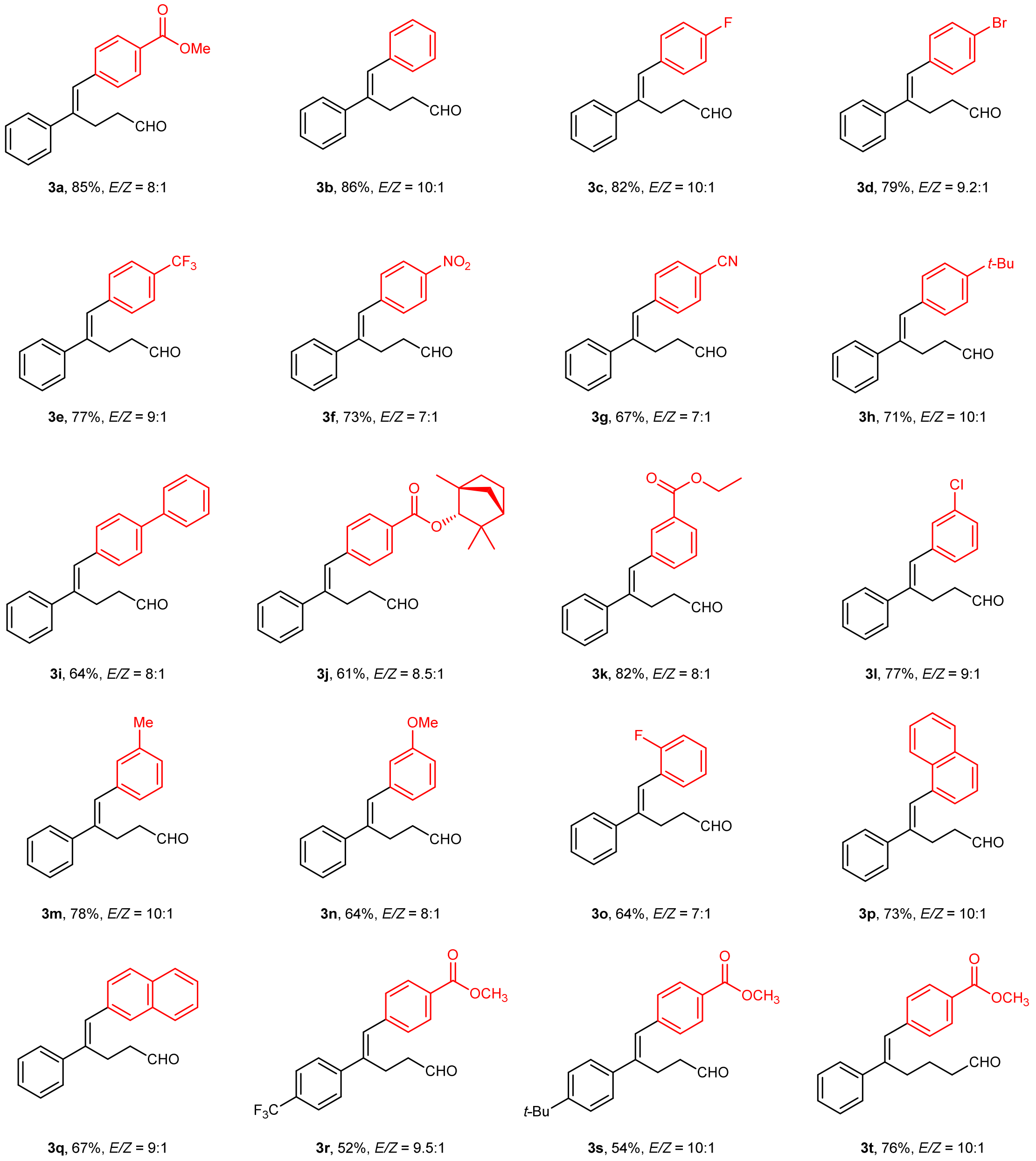

Supplementary Figure 2. The substrate scope for mono-arylation

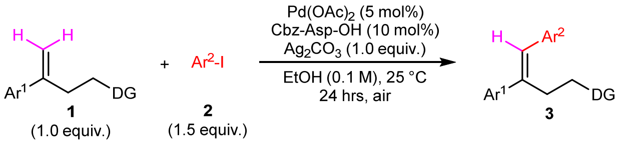

General Procedure A: To an oven-dried 35 mL Schlenk tube with previously placed magnetic stir-bar were added aryl iodide **2** (0.3 mmol, 1.5 equiv.), $Pd(OAc)_2$ (2.2 mg, 0.01 mmol, 5 mol%), Cbz-Asp-OH (5.3 mg, 0.02 mmol, 10 mol%), Ag_2CO_3 (55 mg, 0.2 mmol, 1 equiv.), followed by addition of EtOH (2 mL) and alkene substrate **1** (0.2 mmol). The tube was sealed with a screw cap and the reaction mixture was stirred vigorously at room temperature (25 °C). After stirring for 24 hours, the resultant solution was filtered through a short pad of 1:1 mixture of Celite and silica gel, and the column was washed with ethyl acetate (15 mL). The combined organic solutions were concentrated under reduced pressure, and the residue was purified by flash column chromatography on silica gel to afford the desired product **3**.

Product Characterization

methyl (*E*)-4-(5-oxo-2-phenylpent-1-en-1-yl)benzoate (3a)

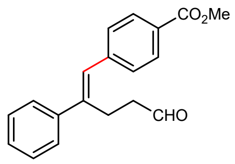

Following the General Procedure A. After concentration and purification by flash chromatography on silica gel (eluent = petroleum ether/ ethyl acetate = 5:1), the product was obtained in 85% yield, *E*/*Z* = 8:1 (49.8 mg, colorless oil). ^{1}**H NMR** (400 MHz, $CDCl_3$): δ 9.69 (s, 1H), 8.04 (d, *J* = 8.3 Hz, 2H), 7.46–7.31 (m, 7H), 6.77 (s, 1H), 3.93 (s, 3H), 3.05 (t, *J* = 7.9 Hz, 2H), 2.54 (t, *J* = 8.0 Hz, 2H); ^{13}**C NMR** (101 MHz, $CDCl_3$): δ 201.15, 166.89, 142.70, 142.40, 141.44, 129.78, 128.74, 128.68, 128.56, 128.47, 127.98, 126.66, 52.17, 42.71, 22.80; **HRMS** (ESI) *m*/*z* $[M+Na]^+$ Calcd for $C_{19}H_{18}NaO_3$: 317.1148, found: 317.1149.

(*E*)-4,5-diphenylpent-4-enal (3b)

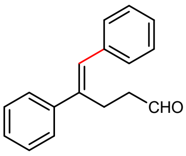

Following the General Procedure A. After concentration and purification by flash chromatography on silica gel (eluent = petroleum ether/ ethyl acetate = 10:1), the product was obtained in 86% yield, *E*/*Z* = 10:1 (40.6 mg, colorless oil). **^{1}H NMR** (400 MHz, $CDCl_3$): δ 9.69 (s, 1H), 7.44 (d, *J* = 7.3 Hz, 2H), 7.37 (m, 4H), 7.34–7.23 (m, 4H), 6.77 (s, 1H), 3.05 (t, *J* = 8.1 Hz, 2H), 2.53 (t, *J* = 8.1 Hz, 2H); **^{13}C NMR** (101 MHz, $CDCl_3$): δ 201.62, 141.84, 140.75, 137.69, 129.54, 128.67, 128.66, 128.48, 127.64, 126.95, 126.66, 42.87, 22.74; **HRMS** (ESI) *m*/*z* $[M+Na]^+$ Calcd for $C_{17}H_{16}NaO$: 259.1093, found: 259.1095.

(*E*)-5-(4-fluorophenyl)-4-phenylpent-4-enal (3c)

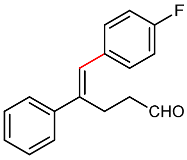

Following the General Procedure A. After concentration and purification by flash chromatography on silica gel (eluent = petroleum ether/ ethyl acetate = 10:1), the product was obtained in 82% yield, *E*/*Z* = 10:1 (41.7 mg, colorless oil). **^{1}H NMR** (400 MHz, $CDCl_3$): δ 9.69 (s, 1H), 7.44–7.31 (m, 5H), 7.26 (t, *J* = 7.0 Hz, 2H), 7.06 (t, *J* = 8.7 Hz, 2H), 6.71 (s, 1H), 3.01 (t, *J* = 7.8 Hz, 2H), 2.52 (t, *J* = 7.8 Hz, 2H); **^{13}C NMR** (101 MHz, $CDCl_3$): δ 201.41, 161.69 (d, *J* = 246.5 Hz), 141.65, 140.88, 133.67 (d, *J* = 3.5 Hz), 130.28 (d, *J* = 7.8 Hz), 128.68, 128.39, 127.71, 126.63, 115.41 (d, *J* = 21.4 Hz), 42.75, 22.62; **^{19}F NMR** (376 MHz, $CDCl_3$): δ –115.02; **HRMS** (ESI) *m*/*z* $[M+Na]^+$ Calcd for $C_{17}H_{15}FNaO$: 277.0999, found: 277.0997.

(*E*)-5-(4-bromophenyl)-4-phenylpent-4-enal (3d)

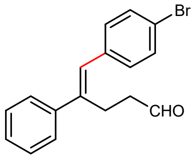

Following the General Procedure A. After concentration and purification by flash chromatography on silica gel (eluent = petroleum ether/ ethyl acetate = 10:1), the product was obtained in 79% yield, *E*/*Z* = 9:1 (49.5 mg, colorless oil). **^{1}H NMR** (600 MHz, $CDCl_3$): δ 9.69 (s, 1H), 7.50 (d, *J* = 8.4 Hz, 2H), 7.42 (d, *J* = 7.3 Hz, 2H), 7.38 (t, *J* = 7.5 Hz, 2H), 7.32 (t, *J* = 7.2 Hz, 1H), 7.20–7.14 (m, 2H), 6.68 (s, 1H), 3.01 (t, *J* = 7.8 Hz, 2H), 2.54 (t, *J* = 7.8 Hz, 2H); **^{13}C NMR** (151 MHz, $CDCl_3$): δ 201.38, 141.68, 141.61, 136.64, 131.68, 130.42, 128.80, 128.35, 127.92, 126.70, 120.91, 42.77, 22.75; **HRMS** (ESI) *m*/*z* $[M+Na]^+$ Calcd for $C_{17}H_{15}BrNaO$: 337.0198, found: 337.0197.

(*E*)-4-phenyl-5-(4-(trifluoromethyl)phenyl)pent-4-enal (3e)

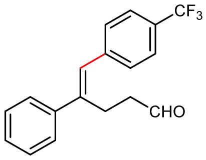

Following the General Procedure A. After concentration and purification by flash chromatography on silica gel (eluent = petroleum ether/ ethyl acetate = 10:1), the product was obtained in 77% yield, *E*/*Z* = 9:1 (46.9 mg, colorless oil). **^{1}H NMR** (400 MHz, $CDCl_3$): δ 9.69 (s, 1H), 7.63 (d, *J* = 8.0 Hz, 2H), 7.47–7.34 (m, 7H), 6.76 (s, 1H), 3.03 (t, *J* = 7.9 Hz, 2H), 2.54 (t, *J* = 7.8 Hz, 2H); **^{13}C NMR** (101 MHz, $CDCl_3$): δ 201.10, 142.80, 141.30, 141.26, 128.96, 128.76, 128.06, 128.03, 126.66, 125.42 (q, *J* = 3.8 Hz), 124.20 (q, *J* = 272.0 Hz), 42.66, 22.69; **^{19}F NMR** (376 MHz, $CDCl_3$): δ –62.44; **HRMS** (ESI) *m*/*z* $[M+Na]^+$ Calcd for $C_{18}H_{15}F_3NaO$: 327.0967, found: 327.0970.

(*E*)-5-(4-nitrophenyl)-4-phenylpent-4-enal (3f)

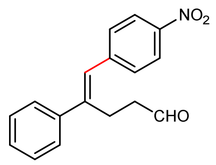

Following the General Procedure A, reaction temperature at 45 ºC. After concentration and purification by flash chromatography on silica gel (eluent = petroleum ether/ ethyl acetate = 5:1), the product was obtained in 73% yield, *E*/*Z* = 7:1 (41.1 mg, yellow solid). **^{1}H NMR** (400 MHz, $CDCl_3$): δ 9.70 (s, 1H), 8.23 (s, 2H), 7.49–7.36 (m, 7H), 6.76 (s, 1H), 3.04 (t, *J* = 7.7 Hz, 2H), 2.56 (t, *J* = 7.7 Hz, 2H); **^{13}C NMR** (101 MHz, $CDCl_3$): δ 200.78, 146.41, 144.48, 144.42, 140.98, 129.50, 128.84, 128.34, 127.36, 126.67, 123.83, 42.50, 22.78; **HRMS** (ESI) *m*/*z* $[M+Na]^+$ Calcd for $C_{17}H_{15}NNaO_3$: 304.0944, found: 304.0947.

(*E*)-4-(5-oxo-2-phenylpent-1-en-1-yl)benzonitrile (3g)

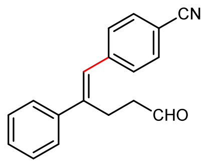

Following the General Procedure A, reaction temperature at 45 ºC. After concentration and purification by flash chromatography on silica gel (eluent = petroleum ether/ ethyl acetate = 5:1), the product was obtained in 67% yield, *E*/*Z* = 7:1 (35.1 mg, colorless oil). **^{1}H NMR** (400 MHz, $CDCl_3$): δ 9.69 (s, 1H), 7.66 (d, *J* = 8.3 Hz, 2H), 7.44–7.33 (m, 7H), 6.72 (s, 1H), 3.02 (t, *J* = 7.8 Hz, 2H), 2.54 (t, *J* = 7.7 Hz, 2H); **^{13}C NMR** (101 MHz, $CDCl_3$): δ 200.87, 143.80, 142.41, 141.06, 132.28, 129.39, 128.81, 128.24, 127.72, 126.66, 118.95, 110.38, 42.53, 22.72; **HRMS** (ESI) *m*/*z* $[M+Na]^+$ Calcd for $C_{18}H_{15}NNaO$: 284.1046, found: 284.1045.

(*E*)-5-(4-(tert-butyl)phenyl)-4-phenylpent-4-enal (3h)

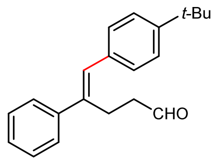

Following the General Procedure A. After concentration and purification by flash chromatography on silica gel (eluent = petroleum ether/ ethyl acetate = 10:1), the product was obtained in 71% yield, *E*/*Z* = 10:1 (41.5 mg, colorless oil). **^{1}H NMR** (400 MHz, $CDCl_3$): δ 9.71 (s, 1H), 7.44–7.34 (m, 6H), 7.30 (d, *J* = 7.2 Hz, 1H), 7.24 (d, *J* = 8.1 Hz, 2H), 6.74 (s, 1H), 3.08 (t, *J* = 7.9 Hz, 2H), 2.54 (t, *J* = 7.9 Hz, 2H), 1.34 (s, 9H); **^{13}C NMR** (101 MHz, $CDCl_3$): δ 201.78, 149.95, 142.05, 140.15, 134.75, 129.43, 128.63, 128.43, 127.53, 126.65, 125.42, 42.95, 34.61, 31.36, 22.81; **HRMS** (ESI) *m*/*z* $[M+Na]^+$ Calcd for $C_{21}H_{24}NaO$: 315.1719, found: 315.1722.

(*E*)-5-([1,1'-biphenyl]-4-yl)-4-phenylpent-4-enal (3i)

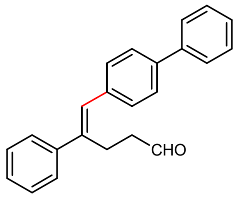

Following the General Procedure A. After concentration and purification by flash chromatography on silica gel (eluent = petroleum ether/ ethyl acetate = 10:1), the product was obtained in 64% yield, *E*/*Z* = 8:1 (39.9 mg, yellow solid). **^{1}H NMR** (400 MHz, $CDCl_3$): δ 9.74 (d, *J* = 1.5 Hz, 1H), 7.63 (dd, *J* = 7.8, 3.3 Hz, 4H), 7.47 (m, 4H), 7.42–7.32 (m, 6H), 6.81 (s, 1H), 3.13 (t, *J* = 7.9 Hz, 2H), 2.58 (t, *J* = 7.9 Hz, 2H); **^{13}C NMR** (101 MHz, $CDCl_3$): δ 201.60, 141.90, 140.95, 140.67, 139.70, 136.68, 129.17, 129.14, 128.86, 128.69, 127.68, 127.39, 127.16, 127.03, 126.67, 42.89, 22.85; **HRMS** (ESI) *m*/*z* $[M+Na]^+$ Calcd for: $C_{23}H_{20}NaO$, 335.1406, found: 335.1409.

(1*R*,2*R*,4*S*)-1,3,3-trimethylbicyclo[2.2.1]heptan-2-yl 4-((*E*)-5-oxo-2-phenylpent-1-en-1-yl)benzoate (3j)

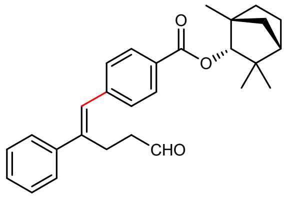

Following the General Procedure A. After concentration and purification by flash chromatography on silica gel (eluent = petroleum ether/ ethyl acetate = 5:1), the product was obtained in 61% yield, *E*/*Z* = 8.5:1 (50.8 mg, colorless oil). ^{1}**H NMR** (400 MHz, $CDCl_3$): δ 9.70 (s, 1H), 8.07 (d, *J* = 8.2 Hz, 2H), 7.47–7.42 (m, 2H), 7.39 (m, 4H), 7.35–7.31 (m, 1H), 6.78 (s, 1H), 4.63 (d, *J* = 1.9 Hz, 1H), 3.06 (t, *J* = 7.8 Hz, 2H), 2.58–2.52 (t, *J* = 7.8 Hz, 2H), 1.95 (m, 1H), 1.79 (dd, *J* = 6.1, 4.3 Hz, 2H), 1.68 (m, 2H), 1.53 (m, 1H), 1.30–1.22 (m, 1H), 1.20 (s, 3H), 1.13 (s, 3H), 0.87 (s, 3H); ^{13}**C NMR** (101 MHz, $CDCl_3$): δ 201.21, 166.66, 142.60, 142.28, 141.43, 129.74, 129.02, 128.74, 128.69, 128.62, 127.98, 126.67, 86.70, 48.67, 48.44, 42.73, 41.49, 39.88, 29.79, 26.93, 25.96, 22.80, 20.38, 19.56; **HRMS** (ESI) *m*/*z* $[M+Na]^+$ Calcd for $C_{28}H_{32}NaO_3$: 439.2244, found: 439.2239.

ethyl (*E*)-3-(5-oxo-2-phenylpent-1-en-1-yl)benzoate (3k)

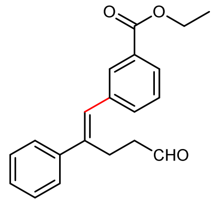

Following the General Procedure A. After concentration and purification by flash chromatography on silica gel (eluent = petroleum ether/ ethyl acetate = 5:1), the product was obtained in 82% yield, *E*/*Z* = 8:1 (50.5 mg, colorless oil). ^{1}**H NMR** (400 MHz, $CDCl_3$): δ 9.69 (s, 1H), 8.01–7.92 (m, 2H), 7.48–7.30 (m, 7H), 6.78 (s, 1H), 4.39 (q, *J* = 7.1 Hz, 2H), 3.03 (t, *J* = 7.8 Hz, 2H), 2.55 (t, *J* = 7.8 Hz, 2H), 1.40 (t, *J* = 7.1 Hz, 3H); ^{13}**C NMR** (101 MHz, $CDCl_3$): δ 201.36, 166.60, 142.00, 141.57, 137.97, 133.07, 130.77, 129.85, 128.79, 128.63, 128.53, 128.08, 127.93, 126.74, 61.20, 42.84, 22.78, 14.42; **HRMS** (ESI) *m*/*z* $[M+Na]^+$ Calcd for $C_{20}H_{20}NaO_3$: 331.1305, found: 331.1309.

(*E*)-5-(3-chlorophenyl)-4-phenylpent-4-enal (3l)

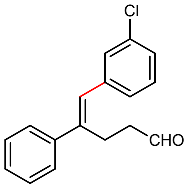

Following the General Procedure A. After concentration and purification by flash chromatography on silica gel (eluent = petroleum ether/ ethyl acetate = 10:1), the product was obtained in 77% yield, *E*/*Z* = 9:1 (41.6 mg, colorless oil). ^{1}**H NMR** (400 MHz, $CDCl_3$): δ 9.68 (s, 1H), 7.44–7.29 (m, 6H), 7.28–7.22 (m, 2H), 7.20–7.15 (m, 1H), 6.68 (s, 1H), 3.02 (t, *J* = 7.8 Hz, 2H), 2.52 (t, *J* = 7.7 Hz, 2H); ^{13}**C NMR** (101 MHz, $CDCl_3$): δ 201.26, 142.14, 141.38, 139.49, 134.30, 129.72, 128.76, 128.72, 128.06, 127.90, 127.01, 126.77, 126.65, 42.69, 22.69; **HRMS** (ESI) *m*/*z* $[M+Na]^+$ Calcd for $C_{17}H_{15}ClNaO$: 293.0704, found: 293.0707.

(*E*)-4-phenyl-5-(*m*-tolyl)pent-4-enal (3m)

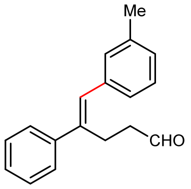

Following the General Procedure A. After concentration and purification by flash chromatography on silica gel (eluent = petroleum ether/ ethyl acetate = 10:1), the product was obtained in 78% yield, *E*/*Z* = 10:1 (39.1 mg, colorless oil). ^{1}**H NMR** (600 MHz, $CDCl_3$): δ 9.69 (s, 1H), 7.43 (dd, *J* = 8.3, 1.4 Hz, 2H), 7.37 (dd, *J* = 8.4, 6.9 Hz, 2H), 7.32–7.29 (m, 1H), 7.28–7.24 (m, 1H), 7.12–7.07 (m, 3H), 6.74 (s, 1H), 3.05 (t, *J* = 7.9 Hz, 2H), 2.52 (t, *J* = 8.2 Hz, 2H), 2.37 (s, 3H); ^{13}**C NMR** (151 MHz, $CDCl_3$): δ 201.77, 141.99, 140.64, 138.13, 137.72, 129.73, 129.57, 128.73, 128.45, 127.80, 127.66, 126.74, 125.70, 42.98, 22.83, 21.61; **HRMS** (ESI) *m*/*z* $[M+Na]^+$ Calcd for $C_{18}H_{18}NaO$: 273.1250, found: 273.1250.

(*E*)-5-(3-methoxyphenyl)-4-phenylpent-4-enal (3n)

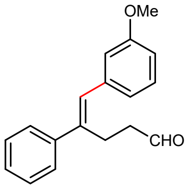

Following the General Procedure A. After concentration and purification by flash chromatography on silica gel (eluent = petroleum ether/ ethyl acetate = 5:1), the product was obtained in 64% yield, *E*/*Z* = 8:1 (34.1 mg, light-yellow oil). **^{1}H NMR** (400 MHz, $CDCl_3$): δ 9.70 (s, 1H), 7.46–7.42 (m, 2H), 7.41–7.36 (m, 2H), 7.34–7.29 (m, 2H), 6.92–6.81 (m, 3H), 6.75 (s, 1H), 3.83 (s, 3H), 3.07 (t, *J* = 7.9, 2H), 2.54 (t, *J* = 7.9, 2H); **^{13}C NMR** (101 MHz, $CDCl_3$): δ 201.69, 159.69, 141.84, 141.08, 139.16, 129.56, 129.47, 128.74, 127.75, 126.74, 121.15, 114.35, 112.53, 55.33, 42.93, 22.91; **HRMS** (ESI) *m*/*z* $[M+Na]^+$ Calcd for $C_{18}H_{18}NaO_2$: 289.1199, found: 289.1202.

(*E*)-5-(2-fluorophenyl)-4-phenylpent-4-enal (3o)

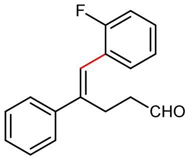

Following the General Procedure A, reaction temperature at 45 ºC. After concentration and purification by flash chromatography on silica gel (eluent = petroleum ether/ ethyl acetate = 5:1), the product was obtained in 64% yield, *E*/*Z* = 7:1 (32.5 mg, colorless oil). **^{1}H NMR** (400 MHz, $CDCl_3$): δ 9.68 (s, 1H), 7.47–7.42 (m, 2H), 7.38 (t, *J* = 7.4 Hz, 2H), 7.34–7.26 (m, 3H), 7.17–7.13 (m, 1H), 7.11–7.06 (m, 1H), 6.67 (s, 1H), 2.95 (t, *J* = 7.8 Hz, 2H), 2.51 (t, *J* = 7.8 Hz, 2H); **^{13}C NMR** (101 MHz, $CDCl_3$): δ 201.58, 160.14 (d, *J* = 246.3 Hz), 143.02, 141.20, 130.48 (d, *J* = 3.3 Hz), 128.88 (d, *J* = 8.1 Hz), 128.67, 127.88, 126.73, 125.28 (d, *J* = 15.2 Hz), 123.97 (d, *J* = 3.6 Hz), 122.10 (d, *J* = 2.3 Hz), 115.67 (d, *J* = 22.3 Hz), 42.56, 23.13; **^{19}F NMR** (376 MHz, $CDCl_3$): δ –114.21; **HRMS** (ESI) *m*/*z* $[M+Na]^+$ Calcd for $C_{17}H_{15}FNaO$: 277.0999, found: 277.1002.

(*E*)-5-(naphthalen-1-yl)-4-phenylpent-4-enal (3p)

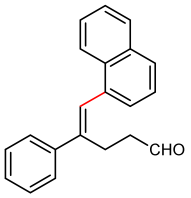

Following the General Procedure A. After concentration and purification by flash chromatography on silica gel (eluent = petroleum ether/ ethyl acetate = 10:1), the product was obtained in 73% yield, *E*/*Z* = 10:1 (41.8 mg, colorless oil). **^{1}H NMR** (400 MHz, $CDCl_3$): δ 9.57 (s, 1H), 8.03–7.97 (m, 1H), 7.92–7.87 (m, 1H), 7.82 (d, *J* = 8.2 Hz, 1H), 7.59–7.55 (m, 2H), 7.51 (dd, *J* = 6.6, 2.8 Hz, 3H), 7.45 (t, *J* = 7.6 Hz, 2H), 7.39 (dd, *J* = 7.2, 5.9 Hz, 2H), 7.18 (s, 1H), 2.92 (t, *J* = 7.8 Hz, 2H), 2.46 (t, *J* = 7.8 Hz, 2H); **^{13}C NMR** (101 MHz, $CDCl_3$): δ 201.64, 142.35, 141.34, 135.13, 133.66, 132.16, 128.78, 128.54, 127.83, 127.68, 127.65, 126.76, 126.13, 126.08, 126.05, 125.50, 125.06, 42.88, 23.14; **HRMS** (ESI) *m/z* $[M+Na]^+$ Calcd for $C_{21}H_{18}NaO$: 309.1250, found: 309.1254.

(*E*)-5-(naphthalen-2-yl)-4-phenylpent-4-enal (3q)

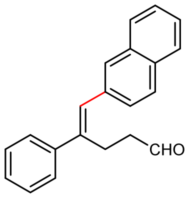

Following the General Procedure A. After concentration and purification by flash chromatography on silica gel (eluent = petroleum ether/ ethyl acetate = 10:1), the product was obtained in 67% yield, *E*/*Z* = 9:1 (38.3 mg, colorless oil). **^{1}H NMR** (400 MHz, $CDCl_3$): δ 9.71 (d, *J* = 1.6 Hz, 1H), 7.89–7.82 (m, 3H), 7.77 (s, 1H), 7.53–7.39 (m, 7H), 7.37–7.33 (m, 1H), 6.94 (s, 1H), 3.15 (t, *J* = 7.8 Hz, 2H), 2.58 (t, *J* = 7.8 Hz, 2H); **^{13}C NMR** (101 MHz, $CDCl_3$): δ 201.56, 141.86, 141.17, 135.21, 133.39, 132.30, 129.54, 128.71, 128.03, 128.00, 127.71, 127.68, 127.41, 127.01, 126.69, 126.30, 126.00, 42.88, 22.84; **HRMS** (ESI) *m/z* $[M+Na]^+$ Calcd for $C_{21}H_{18}NaO$: 309.1250, found: 309.1249.

methyl (*E*)-4-(5-oxo-2-(4-(trifluoromethyl)phenyl)pent-1-en-1-yl)benzoate (3r)

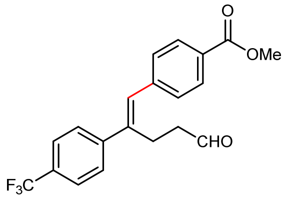

Following the General Procedure A. After concentration and purification by flash chromatography on silica gel (eluent = petroleum ether/ ethyl acetate = 5:1), the product was obtained in 52% yield, *E*/*Z* = 9.5:1 (37.5 mg, colorless oil). **^{1}H NMR** (400 MHz, $CDCl_3$): δ 9.69 (s, 1H), 8.06 (d, *J* = 8.1 Hz, 2H), 7.64 (d, *J* = 8.1 Hz, 2H), 7.54 (d, *J* = 8.2 Hz, 2H), 7.37 (d, *J* = 8.1 Hz, 2H), 6.81 (s, 1H), 3.93 (s, 3H), 3.05 (t, *J* = 7.7 Hz, 2H), 2.53 (t, *J* = 7.7 Hz, 2H); **^{13}C NMR** (101 MHz, $CDCl_3$): δ 200.61, 166.78, 145.14, 141.73, 141.49, 130.22, 129.85, 128.88, 128.67, 127.01, 125.70 (q, *J* = 3.7 Hz), δ 124.08 (q, *J* = 272.0 Hz), 52.21, 42.43, 22.67; **^{19}F NMR** (376 MHz, $CDCl_3$): δ –62.52; **HRMS** (AP-MALDI) *m/z* $[M+NH_4]^+$ Calcd for $C_{20}H_{21}F_3NO_3$: 380.1468, found: 380.1455.

methyl (*E*)-4-(2-(4-(*tert*-butyl)phenyl)-5-oxopent-1-en-1-yl)benzoate (3s)

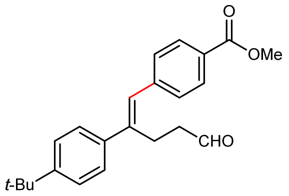

Following the General Procedure A. After concentration and purification by flash chromatography on silica gel (eluent = petroleum ether/ ethyl acetate = 5:1), the product was obtained in 54% yield, *E*/*Z* = 10:1 (37.8 mg, colorless oil). **^{1}H NMR** (400 MHz, $CDCl_3$): δ 9.70 (s, 1H), 8.04 (d, *J* = 8.4 Hz, 2H), 7.42–7.34 (m, 6H), 6.77 (s, 1H), 3.92 (s, 3H), 3.07–3.00 (m, 2H), 2.56 (t, *J* = 7.9 Hz, 2H), 1.34 (s, 9H); **^{13}C NMR** (101 MHz, $CDCl_3$): δ 201.44, 167.01, 151.20, 142.67, 142.50, 138.40, 129.85, 128.74, 128.41, 128.01, 126.32, 125.73, 52.24, 42.91, 34.69, 31.41, 22.74; **HRMS** (ESI) *m*/*z* $[M+Na]^+$ Calcd for $C_{23}H_{26}NaO_3$: 373.1774, found: 373.1771.

methyl (*E*)-4-(6-oxo-2-phenylhex-1-en-1-yl)benzoate (3t)

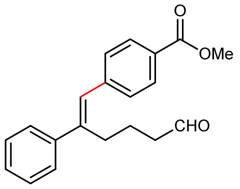

Following the General Procedure A. After concentration and purification by flash chromatography on silica gel (eluent = petroleum ether/ ethyl acetate = 5:1), the product was obtained in 76% yield, *E*/*Z* = 10:1 (46.8 mg, colorless oil). **^{1}H NMR** (400 MHz, $CDCl_3$): δ 9.67 (s, 1H), 8.05 (d, *J* = 8.4 Hz, 2H), 7.49–7.43 (m, 2H), 7.39 (t, *J* = 7.3 Hz, 4H), 7.35–7.31 (m, 1H), 6.75 (s, 1H), 3.93 (s, 3H), 2.78–2.72 (m, 2H), 2.40 (td, *J* = 7.2, 1.5 Hz, 2H), 1.77–1.70 (m, 2H); **^{13}C NMR** (101 MHz, $CDCl_3$): δ 201.97, 166.97, 143.93, 142.71, 142.00, 129.71, 128.72, 128.63, 128.30, 128.18, 127.80, 126.63, 52.16, 43.44, 29.47, 21.01; **HRMS** (ESI) *m*/*z* $[M+Na]^+$ Calcd for $C_{20}H_{20}NaO_3$: 331.1305, found: 331.1303.

2.1.2. General Procedure B and Product Characterization of *β*-Diarylation

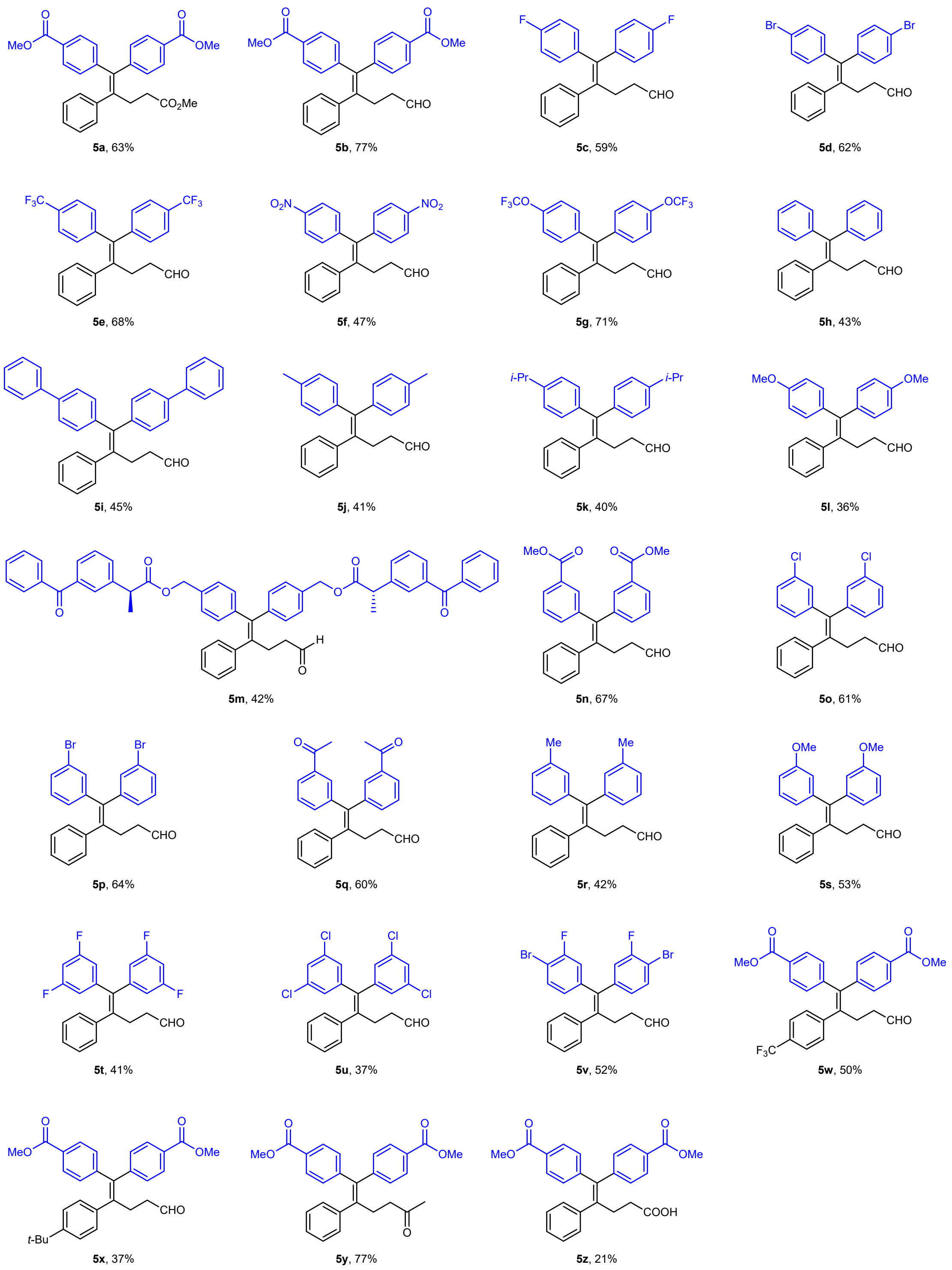

Supplementary Figure 3. The substrate scope for *β*-diarylation

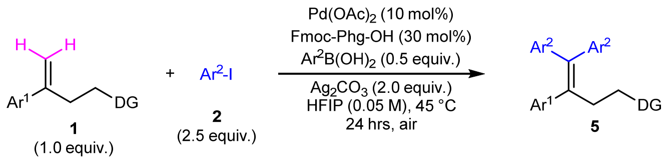

General Procedure B: To an oven-dried 35 mL Schlenk tube with previously placed magnetic stir-bar were added aryl iodide **2** (0.5 mmol, 2.5 equiv.), aryl boronic acid (0.1 mmol, 0.5 equiv.), $Pd(OAc)_2$ (4.5 mg, 0.02 mmol, 10 mol%), Fmoc-Phg-OH (22.4 mg, 0.06 mmol, 30 mol%), Ag_2CO_3 (110 mg, 0.4 mmol, 2 equiv.), followed by addition of HFIP (4 mL) and alkene substrate **1** (0.2 mmol). The tube was sealed with a screw cap and the reaction mixture was stirred vigorously on a hotplate at 45 °C for 24 hours. After completion of the reaction, the resultant solution was filtered through a short pad of 1:1 mixture of Celite and silica gel, and the column was washed with ethyl acetate (15 mL). The combined organic solutions were concentrated under reduced pressure, and the residue was purified by flash column chromatography on silica gel to afford the desired product **5**.

Product Characterization

dimethyl 4,4'-(5-methoxy-5-oxo-2-phenylpent-1-ene-1,1-diyl)dibenzoate (5a)

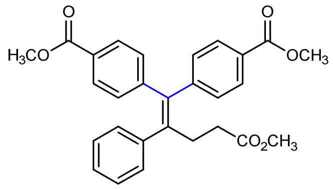

Following the General Procedure B. After concentration and purification by flash chromatography on silica gel (eluent = petroleum ether/ ethyl acetate = 4:1), the product was obtained in 63% yield (57.3 mg, colorless oil). **^{1}H NMR** (400 MHz, $CDCl_3$): δ 8.05 (d, J = 8.5 Hz, 2H), 7.69 (d, J = 8.1 Hz, 2H), 7.31 (d, J = 7.7 Hz, 2H), 7.20–7.13 (m, 3H), 7.11–7.07 (m, 2H), 6.92 (d, J = 7.8 Hz, 2H), 3.92 (s, 3H), 3.82 (s, 3H), 3.55 (s, 3H), 2.82–2.74 (m, 2H), 2.35–2.28 (m, 2H); **^{13}C NMR** (101 MHz, $CDCl_3$): δ 173.09, 166.85, 166.84, 147.02, 146.75, 141.12, 140.11, 138.90, 130.58, 129.89, 129.47, 129.42, 129.01, 128.93, 128.33, 127.86, 127.25, 52.19, 52.01, 51.61, 32.98, 31.11; **HRMS** (ESI) m/z $[M+Na]^+$ Calcd for $C_{28}H_{26}NaO_6$: 481.1622, found: 481.1621.

dimethyl 4,4'-(5-oxo-2-phenylpent-1-ene-1,1-diyl)dibenzoate (5b)

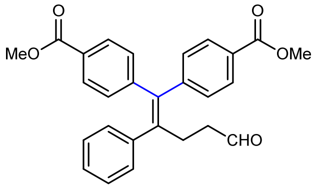

Following the General Procedure B. After concentration and purification by flash chromatography on silica gel (eluent = petroleum ether/ ethyl acetate = 4:1), the product was obtained in 77% yield (65.8 mg, colorless oil). **^{1}H NMR** (400 MHz, $CDCl_3$): δ 9.60 (d, *J* = 1.3 Hz, 1H), 8.08–8.02 (m, 2H), 7.72–7.66 (m, 2H), 7.33–7.28 (m, 2H), 7.21–7.14 (m, 3H), 7.11–7.05 (m, 2H), 6.96–6.90 (m, 2H), 3.92 (s, 3H), 3.82 (s, 3H), 2.77 (t, *J* = 8.7 Hz, 2H), 2.47 (t, *J* = 8.8 Hz, 2H); **^{13}C NMR** (101 MHz, $CDCl_3$): δ 201.04, 166.86, 166.80, 146.97, 146.61, 140.89, 140.10, 138.94, 130.54, 129.96, 129.42, 129.37, 129.07, 128.96, 128.46, 127.91, 127.37, 52.23, 52.04, 42.76, 28.42; **HRMS** (ESI) *m/z* $[M+H]^+$ Calcd for $C_{27}H_{25}O_5$: 429.1697, found: 429.1698.

5,5-bis(4-fluorophenyl)-4-phenylpent-4-enal (5c)

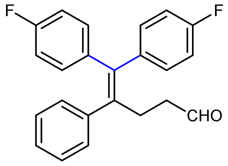

Following the General Procedure B. After concentration and purification by flash chromatography on silica gel (eluent = petroleum ether/ ethyl acetate = 9:1), the product was obtained in 59% yield (41.1 mg, colorless oil). **^{1}H NMR** (400 MHz, $CDCl_3$): δ 9.61 (s, 1H), 7.18 (m, 5H), 7.10–7.03 (m, 4H), 6.82 (dd, *J* = 8.7, 5.7 Hz, 2H), 6.71 (t, *J* = 8.7 Hz, 2H), 2.77 (t, *J* = 7.8 Hz, 2H), 2.45 (t, *J* = 7.8 Hz, 2H); **^{13}C NMR** (101 MHz, $CDCl_3$): δ 201.44, 161.88 (d, *J* = 246.6 Hz), δ 161.14 (d, *J* = 246.4 Hz), 140.77, 138.97, 138.61 (d, *J* = 3.4 Hz), 138.56, 138.21 (d, *J* = 3.3 Hz), 132.09 (d, *J* = 7.9 Hz), 130.81 (d, *J* = 8.0 Hz), 129.52, 128.36, 126.93, 115.51 (d, *J* = 21.4 Hz), 114.51 (d, *J* = 21.3 Hz), 42.91, 28.41; **^{19}F NMR** (376 MHz, $CDCl_3$): δ –114.97, -115.82; **HRMS** (ESI) *m/z* $[M+H]^+$ Calcd for $C_{23}H_{19}F_2O$: 349.1398, found: 349.1397.

5,5-bis(4-bromophenyl)-4-phenylpent-4-enal (5d)

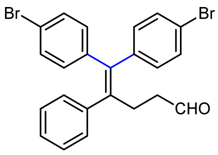

Following the General Procedure B. After concentration and purification by flash chromatography on silica gel (eluent = petroleum ether/ ethyl acetate = 9:1), the product was obtained in 62% yield (58.3 mg, light-yellow oil). **^{1}H NMR** (400 MHz, $CDCl_3$): δ 9.60 (s, 1H), 7.49 (d, *J* = 8.3 Hz, 2H), 7.24–7.16 (m, 3H), 7.14 (d, *J* = 8.5 Hz, 2H), 7.11–7.05 (m, 4H), 6.72 (d, *J* = 8.5 Hz, 2H), 2.76 (t, *J* = 7.8 Hz, 2H), 2.44 (t, *J* = 7.8 Hz, 2H); **^{13}C NMR** (101 MHz, $CDCl_3$): δ 201.22, 141.28, 140.84, 140.39, 139.67, 138.28, 132.20, 131.78, 130.96, 130.78, 129.42, 128.48, 127.17, 121.34, 120.42, 42.79, 28.47; **HRMS** (ESI) *m/z* $[M-H]^-$ Calcd for $C_{23}H_{17}Br_2O$: 466.9652, found: 466.9624.

4-phenyl-5,5-bis(4-(trifluoromethyl)phenyl)pent-4-enal (5e)

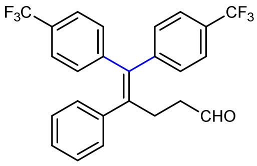

Following the General Procedure B. After concentration and purification by flash chromatography on silica gel (eluent = petroleum ether/ ethyl acetate = 9:1), the product was obtained in 68% yield (60.9 mg, colorless oil). **^{1}H NMR** (400 MHz, $CDCl_3$): δ 9.62 (s, 1H), 7.65 (d, *J* = 8.0 Hz, 2H), 7.37 (d, *J* = 7.9 Hz, 2H), 7.29 (d, *J* = 8.1 Hz, 2H), 7.24–7.18 (m, 3H), 7.11–7.07 (m, 2H), 6.98 (d, *J* = 8.1 Hz, 2H), 2.78 (t, *J* = 7.7 Hz, 2H), 2.48 (t, *J* = 7.6 Hz, 2H); **^{13}C NMR** (101 MHz, $CDCl_3$): δ 200.94, 145.78, 145.32, 141.28, 139.82, 138.04, 130.76, 129.67, 129.37, 128.55, 127.49, 125.69 (q, *J* = 3.8 Hz), δ 124.08 (q, *J* = 272.2 Hz), 124.66 (q, *J* = 3.9 Hz), δ 124.02 (q, *J* = 271.9 Hz), 42.62, 28.38; **^{19}F NMR** (376 MHz, $CDCl_3$): δ –62.51, –62.57; **HRMS** (ESI) *m/z* $[M-H]^-$ Calcd for $C_{25}H_{17}F_6O$: 447.1189, found: 447.1188.

5,5-bis(4-nitrophenyl)-4-phenylpent-4-enal (5f)

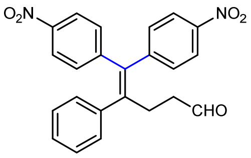

Following the General Procedure B. After concentration and purification by flash chromatography on silica gel (eluent = petroleum ether/ ethyl acetate = 4:1), the product was obtained in 47% yield (37.8 mg, light-yellow oil). **^{1}H NMR** (400 MHz, $CDCl_3$): δ 9.62 (s, 1H), 8.26 (d, *J* = 8.7 Hz, 2H), 7.89 (d, *J* = 8.8 Hz, 2H), 7.46 (d, *J* = 8.7 Hz, 2H), 7.25–7.19 (m, 3H), 7.07 (dd, *J* = 6.7, 3.0 Hz, 2H), 7.01 (d, *J* = 8.9 Hz, 2H), 2.79 (t, *J* = 7.6 Hz, 2H), 2.50 (t, *J* = 7.5 Hz, 2H); **^{13}C NMR** (101 MHz, $CDCl_3$): δ 200.58, 148.37, 148.24, 147.22, 146.20, 143.66, 139.21, 136.71, 131.43, 130.52, 129.29, 128.88, 128.12, 124.23, 123.22, 42.32, 28.41; **HRMS** (ESI) *m/z* $[M-H]^-$ Calcd for $C_{23}H_{17}N_2O_5$: 401.1143, found: 401.1140.

4-phenyl-5,5-bis(4-(trifluoromethoxy)phenyl)pent-4-enal (5g)

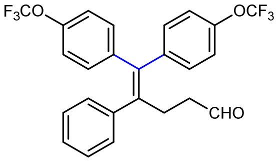

Following the General Procedure B. After concentration and purification by flash chromatography on silica gel (eluent = petroleum ether/ ethyl acetate = 9:1), the product was obtained in 71% yield (73.4 mg, colorless oil). **^{1}H NMR** (400 MHz, $CDCl_3$): δ 9.60 (s, 1H), 7.28–7.15 (m, 7H), 7.09–7.05 (m, 2H), 6.87 (s, 4H), 2.78 (t, *J* = 7.7 Hz, 2H), 2.46 (t, *J* = 7.7 Hz, 2H); **^{13}C NMR** (101 MHz, $CDCl_3$): δ 201.19, 148.31 (d, *J* = 2.0 Hz), 147.45 (d, *J* = 1.9 Hz), 140.95, 140.56, 140.29, 140.12, 137.92, 131.84, 130.68, 129.43, 128.43, 127.19, 121.03, 120.48 (d, *J* = 257.3 Hz), 120.36 (d, *J* = 257.1 Hz), 119.96, 42.74, 28.36; **^{19}F NMR** (376 MHz, $CDCl_3$): δ –57.74, –57.85; **HRMS** (ESI) *m/z* $[M-H]^-$ Calcd for $C_{25}H_{17}F_6O_3$: 479.1087, found: 479.1089.

4,5,5-triphenylpent-4-enal (5h)

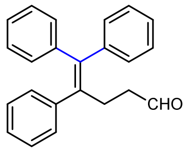

Following the General Procedure B. After concentration and purification by flash chromatography on silica gel (eluent = petroleum ether/ ethyl acetate = 9:1), the product was obtained in 43% yield (26.9 mg, colorless oil). **^{1}H NMR** (400 MHz, $CDCl_3$): δ 9.59 (s, 1H), 7.35 (dd, *J* = 8.1, 6.5 Hz, 2H), 7.28 (d, *J* = 7.2 Hz, 1H), 7.24–7.21 (m, 2H), 7.19–7.15 (m, 2H), 7.12 (m, 3H), 7.04–6.98 (m, 3H), 6.89 (dd, *J* = 7.5, 2.2 Hz, 2H), 2.79 (t, *J* = 7.8 Hz, 2H), 2.45 (t, *J* = 7.8 Hz, 2H); **^{13}C NMR** (101 MHz, $CDCl_3$): δ 201.78, 142.92, 142.39, 141.14, 140.78, 138.24, 130.56, 129.66, 129.18, 128.47, 128.19, 127.48, 127.00, 126.72, 126.09, 43.12, 28.50; **HRMS** (ESI) *m/z* $[M+H]^+$ Calcd for $C_{23}H_{21}O$: 313.1587, found: 313.1587.

5,5-di([1,1'-biphenyl]-4-yl)-4-phenylpent-4-enal (5i)

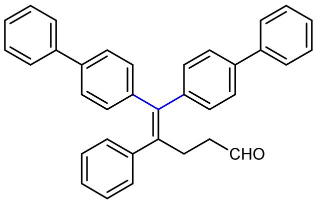

Following the General Procedure B. After concentration and purification by flash chromatography on silica gel (eluent = petroleum ether/ ethyl acetate = 9:1), the product was obtained in 45% yield (41.8 mg, light-yellow oil). **^{1}H NMR** (400 MHz, $CDCl_3$): δ 9.64 (s, 1H), 7.65–7.25 (m, 17H), 7.19 (m, 4H), 6.99 (d, *J* = 8.3 Hz, 2H), 2.88 (t, *J* = 7.8 Hz, 2H), 2.51 (t, *J* = 7.8 Hz, 2H); **^{13}C NMR** (101 MHz, $CDCl_3$): δ 201.80, 141.92, 141.39, 141.15, 140.70, 140.60, 139.97, 139.84, 138.65, 138.63, 131.10, 129.76, 129.67, 128.83, 128.68, 128.34, 127.37, 127.20, 127.15, 127.08, 126.85, 126.12, 43.17, 28.71; **HRMS** (ESI) *m/z* $[M+H]^+$ Calcd for $C_{35}H_{29}O$: 465.2213, found: 465.2213.

4-phenyl-5,5-di-*p*-tolylpent-4-enal (5j)

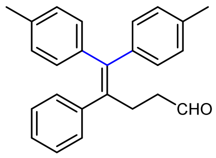

Following the General Procedure B. After concentration and purification by flash chromatography on silica gel (eluent = petroleum ether/ ethyl acetate = 9:1), the product was obtained in 41% yield (27.9 mg, colorless oil). ^{1}**H NMR** (400 MHz, $CDCl_3$): δ 9.60 (s, 1H), 7.21–7.09 (m, 9H), 6.82 (d, J = 8.0 Hz, 2H), 6.77 (d, J = 8.2 Hz, 2H), 2.79 (t, J = 7.9 Hz, 2H), 2.45 (t, J = 7.9 Hz, 2H), 2.37 (s, 3H), 2.19 (s, 3H); ^{13}**C NMR** (101 MHz, $CDCl_3$): δ 201.99, 141.51, 140.58, 140.27, 139.62, 137.42, 136.54, 135.58, 130.47, 129.67, 129.12, 129.07, 128.19, 126.54, 43.22, 28.65, 21.24, 21.10; **HRMS** (ESI) *m/z* $[M+Na]^+$ Calcd for $C_{25}H_{24}NaO$: 363.1719, found: 363.1717.

5,5-bis(4-isopropylphenyl)-4-phenylpent-4-enal (5k)

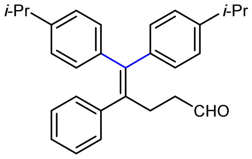

Following the General Procedure B. After concentration and purification by flash chromatography on silica gel (eluent = petroleum ether/ ethyl acetate = 9:1), the product was obtained in 40% yield (31.7 mg, colorless oil). ^{1}**H NMR** (400 MHz, $CDCl_3$): δ 9.60 (s, 1H), 7.21–7.11 (m, 9H), 6.86 (d, J = 8.3 Hz, 2H), 6.79 (d, J = 8.3 Hz, 2H), 2.94–2.89 (m, 1H), 2.79 (t, J = 7.8 Hz, 2H), 2.76–2.70 (m, 1H), 2.45 (t, J = 7.8 Hz, 2H), 1.27 (d, J = 6.9 Hz, 6H), 1.13 (d, J = 7.0 Hz, 6H); ^{13}**C NMR** (101 MHz, $CDCl_3$): δ 202.14, 147.33, 146.44, 141.53, 140.74, 140.49, 139.83, 137.37, 130.42, 129.70, 129.03, 128.09, 126.47, 126.40, 125.40, 43.25, 33.80, 33.54, 28.61, 24.01, 23.84; **HRMS** (ESI) *m/z* $[M+Na]^+$ Calcd for $C_{29}H_{32}NaO$: 419.2345, found: 419.2347.

5,5-bis(4-methoxyphenyl)-4-phenylpent-4-enal (5l)

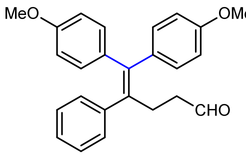

Following the General Procedure B. After concentration and purification by flash chromatography on silica gel (eluent = petroleum ether/ ethyl acetate = 5:1), the product was obtained in 36% yield (26.8 mg, light-yellow oil). **^{1}H NMR** (400 MHz, $CDCl_3$): δ 9.60 (s, 1H), 7.22–7.16 (m, 2H), 7.16–7.08 (m, 5H), 6.89 (d, *J* = 8.5 Hz, 2H), 6.78 (d, *J* = 8.7 Hz, 2H), 6.55 (d, *J* = 8.6 Hz, 2H), 3.82 (s, 3H), 3.68 (s, 3H), 2.80 (t, *J* = 7.9 Hz, 2H), 2.45 (t, *J* = 7.9 Hz, 2H); **^{13}C NMR** (101 MHz, $CDCl_3$): δ 202.00, 158.49, 157.67, 141.64, 139.80, 136.96, 135.67, 135.17, 131.82, 130.37, 129.68, 128.23, 126.47, 113.76, 112.80, 55.26, 55.03, 43.25, 28.64; **HRMS** (ESI) *m/z* $[M+H]^+$ Calcd for $C_{25}H_{25}O_3$: 373.1798, found: 373.1798.

((5-oxo-2-phenylpent-1-ene-1,1-diyl)bis(4,1-phenylene))bis(methylene) (2S,2'S)-bis(2-(3-benzoylphenyl)propanoate) (5m)

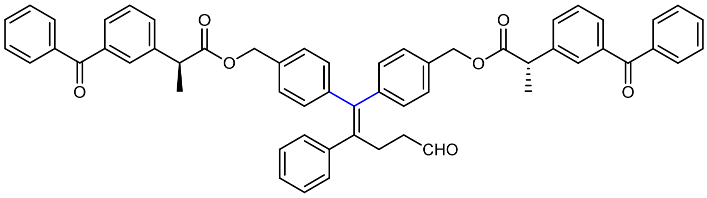

Following the General Procedure B, without using $ArB(OH)_2$. After concentration and purification by flash chromatography on silica gel (eluent = petroleum ether/ ethyl acetate = 5:1), the product was obtained in 42% yield (70.9 mg, light-yellow oil). **^{1}H NMR** (400 MHz, $CDCl_3$): δ 9.58 (s, 1H), 7.80–7.73 (m, 6H), 7.71 (s, 1H), 7.69–7.65 (m, 2H), 7.59–7.54 (m, 3H), 7.48–7.42 (m, 6H), 7.37 (d, *J* = 7.7 Hz, 1H), 7.23 (d, *J* = 7.9 Hz, 2H), 7.17–7.13 (m, 4H), 7.08–7.05 (m, 2H), 6.88 (d, *J* = 8.0 Hz, 2H), 6.80 (d, *J* = 8.2 Hz, 2H), 5.13 (q, *J* = 12.6 Hz, 2H), 4.96 (q, *J* = 12.6 Hz, 2H), 3.88 (q, *J* = 7.2 Hz, 1H), 3.78 (q, *J* = 7.2 Hz, 1H), 2.74 (dd, *J* = 8.8, 6.8 Hz, 2H), 2.47–2.39 (m, 2H), 1.57 (d, *J* = 7.2 Hz, 3H), 1.50 (d, *J* = 7.2 Hz, 3H); **^{13}C NMR** (101 MHz, $CDCl_3$): δ 201.57, 196.52, 196.49, 173.93, 173.83, 142.64, 142.10, 140.78, 140.71, 140.64, 139.62, 138.97, 137.91, 137.84, 137.46, 134.55, 133.51, 132.56, 132.53, 131.59, 131.52, 130.63, 130.11, 130.08, 129.54, 129.35, 129.32, 129.10, 129.02, 128.61, 128.56, 128.35, 128.33, 128.27, 128.05, 127.03, 126.87, 66.34, 66.28, 45.42, 45.35,

42.97, 28.47, 18.47, 18.43; **HRMS** (ESI) *m/z* $[M+Na]^+$ Calcd for $C_{57}H_{48}NaO_7$: 867.3292, found: 867.3300.

dimethyl 3,3'-(5-oxo-2-phenylpent-1-ene-1,1-diyl)dibenzoate (5n)

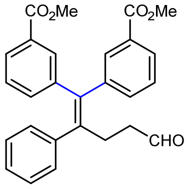

Following the General Procedure B. After concentration and purification by flash chromatography on silica gel (eluent = petroleum ether/ ethyl acetate = 4:1), the product was obtained in 67% yield (57.4 mg, colorless oil). **^{1}H NMR** (400 MHz, $CDCl_3$): δ 9.60 (s, 1H), 7.98–7.05 (m, 13H), 3.91 (s, 3H), 3.79 (s, 3H), 2.75 (t, *J* = 7.8 Hz, 2H), 2.48 (t, *J* = 7.8 Hz, 2H); **^{13}C NMR** (101 MHz, $CDCl_3$): δ 201.23, 166.91, 166.88, 142.49, 142.22, 140.44, 140.28, 138.76, 135.02, 133.78, 131.51, 130.54, 130.27, 129.65, 129.52, 128.83, 128.48, 128.36, 127.74, 127.53, 127.11, 52.27, 52.03, 42.83, 28.36; **HRMS** (ESI) *m/z* $[M+Na]^+$ Calcd for $C_{27}H_{24}NaO_5$: 451.1516, found: 451.1519.

5,5-bis(3-chlorophenyl)-4-phenylpent-4-enal (5o)

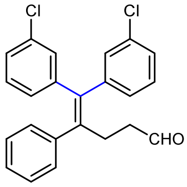

Following the General Procedure B. After concentration and purification by flash chromatography on silica gel (eluent = petroleum ether/ ethyl acetate = 9:1), the product was obtained in 61% yield (46.5 mg, light-yellow oil). **^{1}H NMR** (400 MHz, $CDCl_3$): δ 9.60 (s, 1H), 7.32–7.27 (m, 2H), 7.22–7.16 (m, 4H), 7.13–6.95 (m, 4H), 6.94 (t, *J* = 7.8 Hz, 1H), 6.86 (t, *J* = 1.8 Hz, 1H), 6.74 (d, *J* = 7.5 Hz, 1H), 2.75 (t, *J* = 7.8 Hz, 2H), 2.45 (t, *J* = 7.8 Hz, 2H); **^{13}C NMR** (101 MHz, $CDCl_3$): δ 201.16, 143.87, 143.50, 140.52, 140.12, 138.02, 134.51, 133.50, 130.37, 129.94, 129.39, 129.26, 128.87, 128.70, 128.42, 127.53, 127.36, 127.27, 126.56, 42.74, 28.37; **HRMS** (ESI) *m/z* $[M-H]^-$ Calcd for $C_{23}H_{17}Cl_2O$: 379.0662, found: 379.0660.

5,5-bis(3-bromophenyl)-4-phenylpent-4-enal (5p)

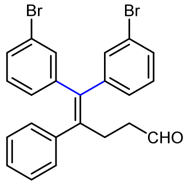

Following the General Procedure B. After concentration and purification by flash chromatography on silica gel (eluent = petroleum ether/ ethyl acetate = 9:1), the product was obtained in 64% yield (60.1 mg, light-yellow oil). **^{1}H NMR** (400 MHz, $CDCl_3$): δ 9.60 (d, *J* = 1.4 Hz, 1H), 7.50–6.75 (m, 13H), 2.75 (t, *J* = 7.8 Hz, 2H), 2.44 (t, *J* = 7.8 Hz, 2H); **^{13}C NMR** (101 MHz, $CDCl_3$): δ 201.14, 144.11, 143.75, 140.67, 140.06, 137.85, 133.26, 132.11, 130.45, 130.22, 129.47, 129.39, 129.16, 129.15, 128.43, 127.82, 127.29, 122.74, 121.75, 42.72, 28.37; **HRMS** (ESI) *m/z* $[M-H]^-$ Calcd for $C_{23}H_{17}Br_2O$: 466.9652, found: 466.9645.

5,5-bis(3-acetylphenyl)-4-phenylpent-4-enal (5q)

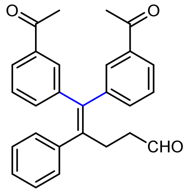

Following the General Procedure B. After concentration and purification by flash chromatography on silica gel (eluent = petroleum ether/ ethyl acetate = 4:1), the product was obtained in 60% yield (47.6 mg, light-yellow oil). **^{1}H NMR** (400 MHz, $CDCl_3$): δ 9.60 (s, 1H), 7.91–7.86 (m, 2H), 7.60 (m, 1H), 7.50–7.43 (m, 3H), 7.21–7.14 (m, 3H), 7.13–7.08 (m, 3H), 7.08–7.04 (m, 1H), 2.76 (t, *J* = 7.7 Hz, 2H), 2.60 (s, 3H), 2.49 (t, *J* = 7.7 Hz, 2H), 2.30 (s, 3H); **^{13}C NMR** (101 MHz, $CDCl_3$): δ 201.13, 198.00, 142.70, 142.12, 140.50, 140.41, 138.73, 137.43, 136.38, 135.12, 134.00, 130.81, 129.49, 129.01, 128.86, 128.49, 128.01, 127.48, 127.23, 126.14, 42.73, 28.42, 26.76, 26.49; **HRMS** (ESI) *m/z* $[M+H]^+$ Calcd for $C_{27}H_{25}O_3$: 397.1798, found: 397.1797.

4-phenyl-5,5-di-*m*-tolylpent-4-enal (5r)

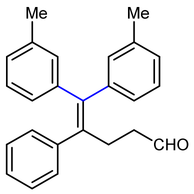

Following the General Procedure B. After concentration and purification by flash chromatography on silica gel (eluent = petroleum ether/ ethyl acetate = 9:1), the product was obtained in 42% yield (28.6 mg, light-yellow oil). **^{1}H NMR** (400 MHz, $CDCl_3$): δ 9.58 (s, 1H), 7.24 (s, 1H), 7.16 (dd, *J* = 7.8, 6.0 Hz, 2H), 7.13–7.01 (m, 6H), 6.89 (t, *J* = 7.5 Hz, 1H), 6.80 (d, *J* = 7.7 Hz, 1H), 6.72–6.63 (m, 2H), 2.76 (t, *J* = 7.8 Hz, 2H), 2.43 (t, *J* = 7.8 Hz, 2H), 2.33 (s, 3H), 2.09 (s, 3H); **^{13}C NMR** (101 MHz, $CDCl_3$): δ 201.95, 142.89, 142.23, 141.31, 140.98, 138.02, 137.88, 136.85, 131.19, 129.79, 129.64, 128.26, 128.10, 127.71, 127.63, 127.25, 126.83, 126.60, 126.13, 43.16, 28.54, 21.54, 21.32; **HRMS** (ESI) *m/z* $[M+Na]^+$ Calcd for $C_{25}H_{24}NaO$: 363.1719, found: 363.1720.

5,5-bis(3-methoxyphenyl)-4-phenylpent-4-enal (5s)

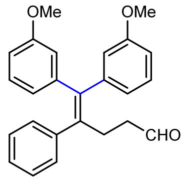

Following the General Procedure B. After concentration and purification by flash chromatography on silica gel (eluent = petroleum ether/ ethyl acetate = 4:1), the product was obtained in 53% yield (39.4 mg, light-yellow oil). **^{1}H NMR** (400 MHz, $CDCl_3$): δ 9.60 (s, 1H), 7.30–7.24 (m, 1H), 7.20–7.10 (m, 5H), 6.93 (t, *J* = 7.9 Hz, 1H), 6.82 (dd, *J* = 7.7, 1.9 Hz, 2H), 6.77 (t, *J* = 2.0 Hz, 1H), 6.55 (m, 1H), 6.49 (d, *J* = 7.7 Hz, 1H), 6.44 (dd, *J* = 2.6, 1.5 Hz, 1H), 3.79 (s, 3H), 3.50 (s, 3H), 2.78 (t, *J* = 7.8 Hz, 2H), 2.45 (t, *J* = 7.8 Hz, 2H); **^{13}C NMR** (101 MHz, $CDCl_3$): δ 201.78, 159.62, 158.65, 144.08, 143.34, 141.16, 140.40, 138.34, 129.51, 129.47, 128.40, 128.25, 126.78, 123.09, 121.55, 115.93, 114.89, 112.31, 112.14, 55.23, 54.96, 43.06, 28.58; **HRMS** (ESI) *m/z* $[M+Na]^+$ Calcd for $C_{25}H_{24}NaO_3$: 395.1618, found: 395.1620.

5,5-bis(3,5-difluorophenyl)-4-phenylpent-4-enal (5t)

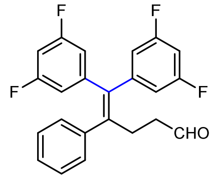

Following the General Procedure B. After concentration and purification by flash chromatography on silica gel (eluent = petroleum ether/ ethyl acetate = 9:1), the product was obtained in 41% yield (31.5 mg, colorless oil). **^{1}H NMR** (400 MHz, $CDCl_3$): δ 9.61 (s, 1H), 7.25–7.19 (m, 3H), 7.07 (dd, J = 7.5, 2.1 Hz, 2H), 6.78 (m, 3H), 6.52–6.46 (m, 1H), 6.41–6.35 (m, 2H), 2.76 (t, J = 7.7 Hz, 2H), 2.45 (t, J = 7.6 Hz, 2H); **^{13}C NMR** (101 MHz, $CDCl_3$): δ 200.72, 163.14 (dd, J = 250.0, 12.9 Hz), 162.35 (dd, J = 249.5, 12.8 Hz), 144.69 (t, J = 9.3 Hz), 144.39 (t, J = 9.4 Hz), 141.85, 139.40, 136.31, 129.05, 128.63, 127.73, 113.54–113.04 (m), 112.42–112.00 (m), 103.17 (t, J = 25.2 Hz), 102.23 (t, J = 25.4 Hz), 42.40, 28.29; **^{19}F NMR** (376 MHz, $CDCl_3$): δ –108.89, –110.48; **HRMS** (ESI) *m/z* $[M+Na]^+$ Calcd for $C_{23}H_{16}F_4NaO$: 407.1029, found: 407.1031.

5,5-bis(3,5-dichlorophenyl)-4-phenylpent-4-enal (5u)

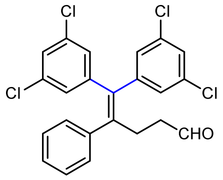

Following the General Procedure B. After concentration and purification by flash chromatography on silica gel (eluent = petroleum ether/ ethyl acetate = 9:1), the product was obtained in 37% yield (33.3 mg, colorless oil). **^{1}H NMR** (400 MHz, $CDCl_3$): δ 9.62 (s, 1H), 7.34 (t, J = 2.0 Hz, 1H), 7.25–7.20 (m, 3H), 7.14 (d, J = 1.9 Hz, 2H), 7.05 (dd, J = 7.4, 2.0 Hz, 3H), 6.72 (d, J = 1.9 Hz, 2H), 2.74 (t, J = 7.6 Hz, 2H), 2.45 (t, J = 7.6 Hz, 2H); **^{13}C NMR** (101 MHz, $CDCl_3$): δ 200.62, 144.19, 143.95, 142.65, 139.16, 135.55, 135.40, 134.29, 129.10, 128.73, 128.66, 127.87, 127.81, 127.64, 126.84, 42.33, 28.25; **HRMS** (ESI) *m/z* $[M-H]^-$ Calcd for $C_{23}H_{15}Cl_4O$: 446.9882, found: 446.9889.

5,5-bis(4-bromo-3-fluorophenyl)-4-phenylpent-4-enal (5v)

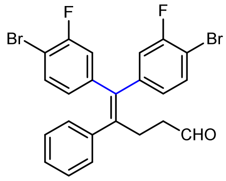

Following the General Procedure B. After concentration and purification by flash chromatography on silica gel (eluent = petroleum ether/ ethyl acetate = 9:1), the product was obtained in 52% yield (52.6 mg, light-yellow oil). **^{1}H NMR** (400 MHz, $CDCl_3$): δ 9.61 (s, 1H), 7.56 (dd, J = 8.1, 7.2 Hz, 1H), 7.24–7.16 (m, 4H), 7.09–7.04 (m, 2H), 7.00 (dd, *J* = 9.2, 2.0 Hz, 1H), 6.92 (dd, *J* = 8.2, 2.0 Hz, 1H), 6.60 (dd, *J* = 9.9, 2.1 Hz, 1H), 6.51 (dd, *J* = 8.3, 2.0 Hz, 1H), 2.76 (t, *J* = 7.7 Hz, 2H), 2.45 (t, *J* = 7.6 Hz, 2H); **^{13}C NMR** (101 MHz, $CDCl_3$): δ 200.81, 159.12 (d, *J* = 249.0 Hz), 158.41 (d, *J* = 247.4 Hz), 143.11 (d, *J* = 6.7 Hz), 142.81 (d, *J* = 6.8 Hz), 141.35, 139.66, 136.37, 133.79, 132.66, 129.17, 128.67, 127.62, 127.41 (d, *J* = 3.4 Hz), 126.20 (d, *J* = 3.4 Hz), 118.39 (d, *J* = 22.7 Hz), 117.46 (d, *J* = 22.1 Hz), 108.21 (d, *J* = 20.8 Hz), 107.16 (d, *J* = 21.0 Hz), 42.47, 28.41; **^{19}F NMR** (376 MHz, $CDCl_3$): δ –106.26, –107.82; **HRMS** (ESI) *m/z* $[M+H]^+$ Calcd for $C_{23}H_{17}Br_2F_2O$: 504.9609, found: 504.9607.

dimethyl 4,4'-(5-oxo-2-(4-(trifluoromethyl)phenyl)pent-1-ene-1,1-diyl)dibenzoate (5w)

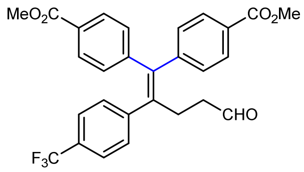

Following the General Procedure B. After concentration and purification by flash chromatography on silica gel (eluent = petroleum ether/ ethyl acetate = 4:1), the product was obtained in 50% yield (49.6 mg, light-yellow oil). **^{1}H NMR** (400 MHz, $CDCl_3$): δ 9.62 (s, 1H), 8.06 (d, *J* = 8.1 Hz, 2H), 7.73 (d, *J* = 8.2 Hz, 2H), 7.46 (d, *J* = 8.0 Hz, 2H), 7.32 (d, *J* = 8.0 Hz, 2H), 7.23 (d, *J* = 8.0 Hz, 2H), 6.93 (d, *J* = 8.3 Hz, 2H), 3.93 (s, 3H), 3.84 (s, 3H), 2.80 (t, *J* = 7.7 Hz, 2H), 2.46 (t, *J* = 7.7 Hz, 2H); **^{13}C NMR** (101 MHz, $CDCl_3$): δ 200.51, 166.68, 166.67, 146.36, 145.87, 144.11, 140.38, 139.36, 130.42, 130.04, 129.80, 129.52, 129.36, 129.20, 128.43, 125.44 (q, *J* = 3.7 Hz), 123.92 (q, *J* = 272.1 Hz), 52.26, 52.12, 42.49, 28.17; **^{19}F NMR** (376 MHz,

CDCl3): δ –62.54; **HRMS** (ESI) *m/z* [M+Na]$^+$ Calcd for $C_{28}H_{23}F_3NaO_5$: 519.1390, found: 519.1391.

dimethyl 4,4'-(2-(4-(tert-butyl)phenyl)-5-oxopent-1-ene-1,1-diyl)dibenzoate (5x)

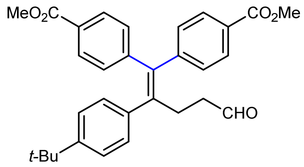

Following the General Procedure B. After concentration and purification by flash chromatography on silica gel (eluent = petroleum ether/ ethyl acetate = 4:1), the product was obtained in 37% yield (35.8 mg, colorless oil). **^{1}H NMR** (400 MHz, $CDCl_3$): δ 9.60 (d, *J* = 1.2 Hz, 1H), 8.03 (d, *J* = 7.6 Hz, 2H), 7.70–7.66 (m, 2H), 7.29 (d, *J* = 8.0 Hz, 2H), 7.18 (d, *J* = 8.5 Hz, 2H), 6.99 (d, *J* = 8.3 Hz, 2H), 6.92 (d, *J* = 8.3 Hz, 2H), 3.92 (s, 3H), 3.83 (s, 3H), 2.78–2.71 (m, 2H), 2.46 (t, *J* = 7.7 Hz, 2H), 1.25 (s, 9H); **^{13}C NMR** (101 MHz, $CDCl_3$): δ 201.39, 167.05, 166.92, 150.51, 147.39, 146.91, 140.92, 138.54, 136.93, 130.67, 130.01, 129.51, 129.11, 128.98, 127.85, 127.36, 125.39, 52.30, 52.12, 42.98, 34.59, 31.35, 28.56; **HRMS** (ESI) *m/z* [M+Na]$^+$ Calcd for $C_{31}H_{32}NaO_5$: 507.2142, found: 507.2144.

dimethyl 4,4'-(5-oxo-2-phenylhex-1-ene-1,1-diyl)dibenzoate (5y)

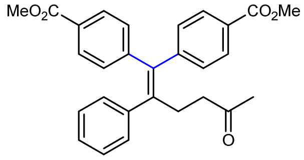

Following the General Procedure B. After concentration and purification by flash chromatography on silica gel (eluent = petroleum ether/ ethyl acetate = 4:1), the product was obtained in 77% yield (68.1 mg, colorless oil). **^{1}H NMR** (400 MHz, $CDCl_3$): δ 8.03 (d, *J* = 8.3 Hz, 2H), 7.68 (d, *J* = 8.4 Hz, 2H), 7.30 (d, *J* = 8.3 Hz, 2H), 7.16 (m, 3H), 7.10–7.05 (m, 2H), 6.91 (d, *J* = 8.4 Hz, 2H), 3.91 (s, 3H), 3.81 (s, 3H), 2.75–2.67 (m, 2H), 2.47–2.40 (m, 2H), 1.98 (s, 3H); **^{13}C NMR** (101 MHz, $CDCl_3$): δ 207.62, 166.87, 166.83, 147.13, 146.82, 141.47, 140.36, 138.57, 130.60, 129.89, 129.44, 129.42, 128.95, 128.93, 128.36, 127.80, 127.23, 52.20, 52.02, 42.31, 29.89; **HRMS** (ESI) *m/z* [M+Na]$^+$ Calcd for $C_{28}H_{26}NaO_5$: 465.1672, found: 465.1673.

2.1.3. General Procedure C and Product Characterization of Stepwise Double Arylation

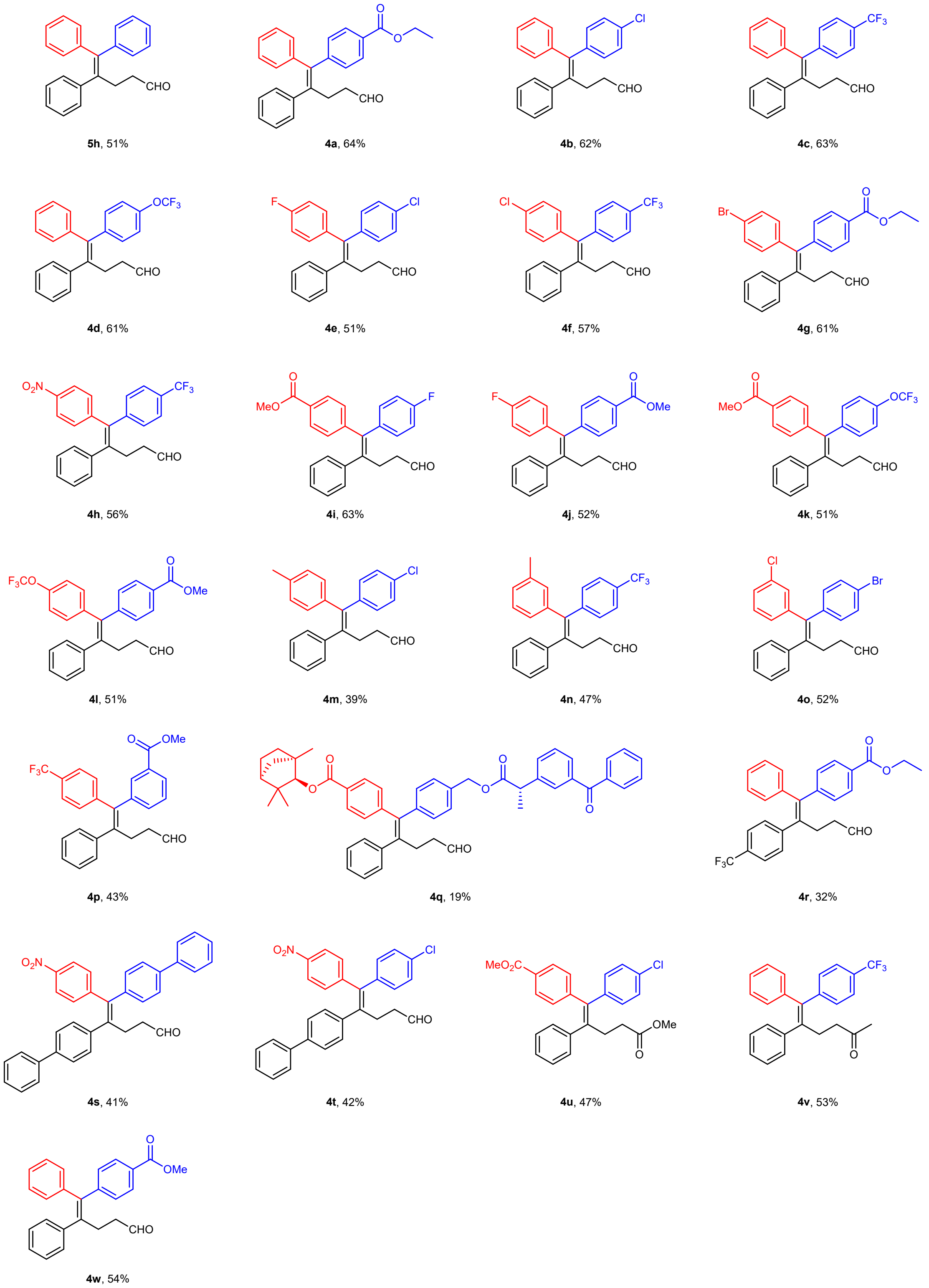

Supplementary Figure 4. Substrate scope for stepwise double arylation

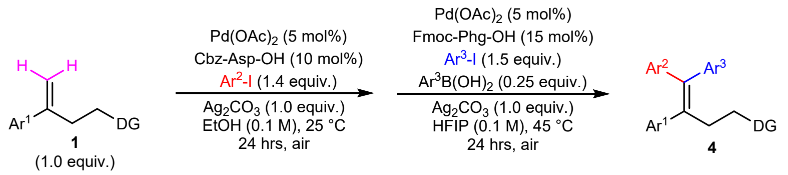

General Procedure C: To an oven-dried 35 mL Schlenk tube with previously placed magnetic stir-bar were added aryl iodide (0.28 mmol, 1.4 equiv.), $Pd(OAc)_2$ (2.2 mg, 0.01 mmol, 5 mol%), Cbz-Asp-OH (5.3 mg, 0.02 mmol, 10 mol%), Ag_2CO_3 (55 mg, 0.2 mmol, 1 equiv.), followed by addition of EtOH (2 mL) and alkene substrate **1** (0.2 mmol). The tube was sealed with a screw cap and the reaction mixture was stirred vigorously at room temperature (25 °C). After stirring for 24 hours, the resultant solution was filtered through a short pad of 1:1 mixture of Celite and silica gel, and the column was washed with ethyl acetate (15 mL). The combined organic solutions were concentrated under reduced pressure to afford the mono-arylated product **3** which was used in the next step without further purification. To another oven-dried 35 mL Schlenk tube with previously placed magnetic stir-bar were added the second aryl iodide (0.3 mmol, 1.5 equiv.), aryl boronic acid (0.05 mmol, 0.25 equiv.), $Pd(OAc)_2$ (2.2 mg, 0.01 mmol, 5 mol%), Fmoc-Phg-OH (11.2 mg, 0.03 mmol, 15 mol%), Ag_2CO_3 (55 mg, 0.2 mmol, 1 equiv.), followed by the addition of the crude mono-arylated product **3** dissolved in 2ml HFIP. The tube was sealed with a screw cap and the reaction mixture was stirred vigorously on a hotplate at 45 °C for 24 hours. After completion of the reaction, the resultant solution was filtered through a short pad of 1:1 mixture of Celite and silica gel, and the column was washed with ethyl acetate (15 mL). The combined organic solutions were concentrated under reduced pressure, and the residue was purified by flash column chromatography on silica gel to afford the desired double arylated product **4**.

Product Characterization

ethyl (*E*)-4-(5-oxo-1,2-diphenylpent-1-en-1-yl)benzoate (4a)

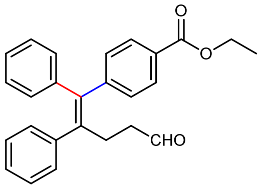

Following the General Procedure C. After concentration and purification by flash chromatography on silica gel (eluent = petroleum ether/ ethyl acetate = 5:1), the product was obtained in 64% yield (49.2 mg, light-yellow oil). **^{1}H NMR** (400 MHz, $CDCl_3$): δ 9.60 (s, 1H), 8.05 (d, *J* = 8.2 Hz, 2H), 7.32 (d, *J* = 8.3 Hz, 2H), 7.21–7.14 (m, 3H), 7.11 (dd, *J* = 8.0, 1.6 Hz, 2H), 7.05–6.99 (m, 3H), 6.87 (dd, *J* = 6.5, 2.9 Hz, 2H), 4.39 (q, *J* = 7.1 Hz, 2H), 2.77 (t, *J* = 7.7 Hz, 2H), 2.47 (t, *J* = 7.8 Hz, 2H), 1.40 (t, *J* = 7.1 Hz, 3H); **^{13}C NMR** (101 MHz, $CDCl_3$): δ 201.45, 166.52, 147.77, 141.80, 140.76, 139.96, 139.22, 130.63, 129.88, 129.64, 129.38, 129.20, 128.36, 127.72, 127.05, 126.47, 61.10, 43.03, 28.50, 14.48; **HRMS** (ESI) *m/z* $[M+Na]^+$ Calcd for $C_{26}H_{24}NaO_3$: 407.1618, found: 407.1617.

(*E*)-5-(4-chlorophenyl)-4,5-diphenylpent-4-enal (4b)

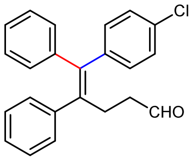

Following the General Procedure C. After concentration and purification by flash chromatography on silica gel (eluent = petroleum ether/ ethyl acetate = 9:1), the product was obtained in 62% yield (42.9 mg, light-yellow oil). **^{1}H NMR** (600 MHz, $CDCl_3$): δ 9.61 (s, 1H), 7.34 (d, *J* = 8.0 Hz, 2H), 7.21–7.16 (m, 4H), 7.14 (d, *J* = 7.0 Hz, 1H), 7.10 (d, *J* = 7.5 Hz, 2H), 7.02 (m, 3H), 6.89–6.83 (m, 2H), 2.79 (t, *J* = 7.8 Hz, 2H), 2.46 (t, *J* = 7.7 Hz, 2H); **^{13}C NMR** (151 MHz, $CDCl_3$): δ 201.56, 142.07, 141.44, 140.89, 139.61, 138.96, 132.99, 130.71, 130.62, 129.63, 128.79, 128.33, 127.68, 126.96, 126.39, 43.04, 28.49; **HRMS** (ESI) *m/z* $[M-H]^-$ Calcd for $C_{23}H_{18}ClO$: 345.1052, found: 345.1050.

(E)-4,5-diphenyl-5-(4-(trifluoromethyl)phenyl)pent-4-enal (4c)

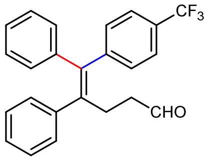

Following the General Procedure C. After concentration and purification by flash chromatography on silica gel (eluent = petroleum ether/ ethyl acetate = 9:1), the product was obtained in 63% yield (47.9 mg, light-yellow oil). **^{1}H NMR** (600 MHz, $CDCl_3$): δ 9.62 (s, 1H), 7.63 (d, J = 8.0 Hz, 2H), 7.38 (d, J = 8.0 Hz, 2H), 7.20 (dd, J = 8.1, 6.4 Hz, 2H), 7.17–7.14 (m, 1H), 7.13–7.10 (m, 2H), 7.06–7.02 (m, 3H), 6.90–6.85 (m, 2H), 2.78 (t, J = 7.8 Hz, 2H), 2.48 (t, J = 7.8 Hz, 2H); **^{13}C NMR** (151 MHz, $CDCl_3$): δ 201.38, 146.70, 141.72, 140.60, 139.49, 130.59, 129.70, 129.60, 129.32, 129.10, 128.38, 127.78, 127.11, 126.54, 125.57 (q, J = 3.7 Hz), δ 124.28 (q, J = 272.1 Hz), 42.93, 28.39; **^{19}F NMR** (565 MHz, $CDCl_3$): δ –62.27; **HRMS** (ESI) m/z $[M-H]^-$ Calcd for $C_{24}H_{18}F_3O$: 379.1315, found: 379.1314.

(E)-4,5-diphenyl-5-(4-(trifluoromethoxy)phenyl)pent-4-enal (4d)

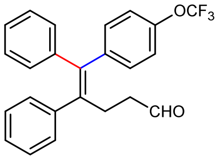

Following the General Procedure C. After concentration and purification by flash chromatography on silica gel (eluent = petroleum ether/ ethyl acetate = 9:1), the product was obtained in 61% yield (48.3 mg, light-yellow oil). **^{1}H NMR** (600 MHz, $CDCl_3$): δ 9.61 (s, 1H), 7.27–7.25 (m, 2H), 7.21–7.16 (m, 4H), 7.15–7.12 (m, 1H), 7.11–7.08 (m, 2H), 7.02 (m, 3H), 6.86 (dd, J = 7.7, 2.0 Hz, 2H), 2.78 (t, J = 7.8 Hz, 2H), 2.46 (t, J = 7.8 Hz, 2H); **^{13}C NMR** (151 MHz, $CDCl_3$): δ 201.52, 148.21, 142.06, 141.58, 140.82, 139.45, 139.15, 130.73, 130.59, 129.63, 128.33, 127.70, 126.98, 126.42, 120.99, 42.99, 28.42; **^{19}F NMR** (565 MHz, $CDCl_3$): δ –57.60; **HRMS** (ESI) m/z $[M+H]^+$ Calcd for $C_{24}H_{20}F_3O_2$: 397.1410, found: 397.1411.

(*E*)-5-(4-chlorophenyl)-5-(4-fluorophenyl)-4-phenylpent-4-enal (4e)

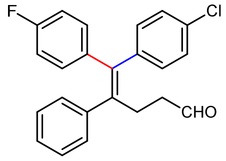

Following the General Procedure C. After concentration and purification by flash chromatography on silica gel (eluent = petroleum ether/ ethyl acetate = 9:1), the product was obtained in 51% yield (37.2 mg, light-yellow oil). **^{1}H NMR** (400 MHz, $CDCl_3$): δ 9.61 (s, 1H), 7.34 (d, *J* = 8.4 Hz, 2H), 7.21–7.14 (m, 5H), 7.10–7.07 (m, 2H), 6.85–6.68 (m, 4H), 2.77 (t, *J* = 7.8 Hz, 2H), 2.47 (t, *J* = 7.7 Hz, 2H); **^{13}C NMR** (101 MHz, $CDCl_3$): δ 201.34, 161.18 (d, *J* = 246.4 Hz), 141.13, 140.63, 139.18, 138.41, 137.96 (d, *J* = 3.5 Hz), 133.07, 132.12 (d, *J* = 7.9 Hz), 130.59, 129.49, 128.79, 128.39, 127.00, 114.57 (d, *J* = 21.3 Hz), 42.88, 28.40; **^{19}F NMR** (376 MHz, $CDCl_3$): δ –115.63; **HRMS** (ESI) *m/z* $[M-H]^-$ Calcd for $C_{23}H_{17}ClFO$: 363.0957, found: 363.0960.

(*Z*)-5-(4-chlorophenyl)-4-phenyl-5-(4-(trifluoromethyl)phenyl)pent-4-enal (4f)

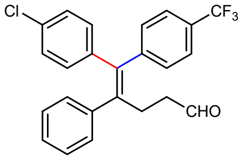

Following the General Procedure C. After concentration and purification by flash chromatography on silica gel (eluent = petroleum ether/ ethyl acetate = 9:1), the product was obtained in 57% yield (47.3 mg, light-yellow oil). **^{1}H NMR** (400 MHz, $CDCl_3$): δ 9.60 (s, 1H), 7.26–7.13 (m, 7H), 7.08 (d, *J* = 6.2 Hz, 2H), 6.99 (d, *J* = 8.5 Hz, 2H), 6.78 (d, *J* = 8.6 Hz, 2H), 2.77 (t, *J* = 7.7 Hz, 2H), 2.45 (t, *J* = 7.7 Hz, 2H); **^{13}C NMR** (101 MHz, $CDCl_3$): δ 201.10, 146.14, 140.20, 140.15, 140.10, 138.13, 132.32, 131.83, 129.63, 129.41, 128.51, 127.94, 127.28, 125.60 (q, *J* = 3.8 Hz), 124.12 (q, *J* = 272.0 Hz), 42.71, 28.36; **^{19}F NMR** (376 MHz, $CDCl_3$): δ –57.72; **HRMS** (ESI) *m/z* $[M+H]^+$ Calcd for $C_{24}H_{19}ClF_3O$: 415.1071, found: 415.1070.

ethyl (*Z*)-4-(1-(4-bromophenyl)-5-oxo-2-phenylpent-1-en-1-yl)benzoate (4g)

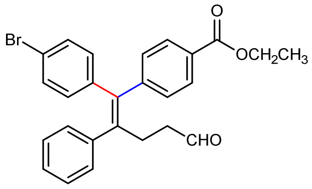

Following the General Procedure C. After concentration and purification by flash chromatography on silica gel (eluent = petroleum ether/ ethyl acetate = 5:1), the product was obtained in 61% yield (56.5 mg, light-yellow oil). **^{1}H NMR** (400 MHz, $CDCl_3$): δ 9.59 (d, *J* = 1.4 Hz, 1H), 8.05 (d, *J* = 8.2 Hz, 2H), 7.29 (d, *J* = 8.2 Hz, 2H), 7.24–7.17 (m, 3H), 7.14 (d, *J* = 8.5 Hz, 2H), 7.09 (dd, *J* = 7.8, 1.8 Hz, 2H), 6.72 (d, *J* = 8.5 Hz, 2H), 4.39 (q, *J* = 7.1 Hz, 2H), 2.74 (t, *J* = 7.8 Hz, 2H), 2.45 (t, *J* = 7.7 Hz, 2H), 1.40 (t, *J* = 7.1 Hz, 3H); **^{13}C NMR** (101 MHz, $CDCl_3$): δ 201.12, 166.32, 147.09, 140.66, 140.28, 139.95, 138.61, 132.20, 130.82, 129.89, 129.44, 129.36, 129.30, 128.50, 127.24, 120.50, 61.08, 42.78, 28.48, 14.39; **HRMS** (ESI) *m/z* $[M+H]^+$ Calcd for $C_{26}H_{24}BrO_3$: 463.0903, found: 463.0903.

(*Z*)-5-(4-nitrophenyl)-4-phenyl-5-(4-(trifluoromethyl)phenyl)pent-4-enal (4h)

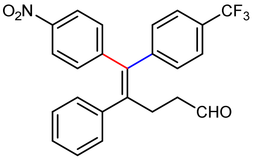

Following the General Procedure C. After concentration and purification by flash chromatography on silica gel (eluent = petroleum ether/ ethyl acetate = 5:1), the product was obtained in 56% yield (47.6 mg, light-yellow oil). **^{1}H NMR** (600 MHz, $CDCl_3$): δ 9.62 (s, 1H), 7.89 (d, *J* = 8.9 Hz, 2H), 7.66 (d, *J* = 8.0 Hz, 2H), 7.37 (d, *J* = 7.9 Hz, 2H), 7.23–7.20 (m, 3H), 7.08 (dd, *J* = 7.3, 2.2 Hz, 2H), 7.02 (d, *J* = 8.9 Hz, 2H), 2.79 (t, *J* = 7.7 Hz, 2H), 2.48 (t, *J* = 7.7 Hz, 2H); **^{13}C NMR** (151 MHz, $CDCl_3$): δ 200.80, 148.71, 146.06, 145.24, 142.86, 139.57, 137.49, 131.39, 129.82, 129.39, 128.82, 127.93, 125.94 (q, *J* = 3.8 Hz), 124.07 (q, *J* = 272.1 Hz), 123.11, 42.54, 28.48; **^{19}F NMR** (565 MHz, $CDCl_3$): δ –62.43; **HRMS** (ESI) *m/z* $[M+Na]^+$ Calcd for $C_{24}H_{18}F_3NNaO_3$: 448.1131, found: 448.1130.

methyl (*E*)-4-(1-(4-fluorophenyl)-5-oxo-2-phenylpent-1-en-1-yl)benzoate (4i)

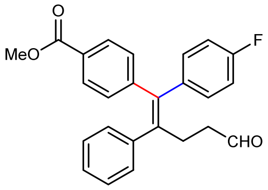

Following the General Procedure C. After concentration and purification by flash chromatography on silica gel (eluent = petroleum ether/ ethyl acetate = 5:1), the product was obtained in 63% yield (48.9 mg, light-yellow oil). ^{1}**H NMR** (400 MHz, $CDCl_3$): δ 9.61 (s, 1H), 7.69 (d, *J* = 8.5 Hz, 2H), 7.18 (m, 5H), 7.10–7.04 (m, 4H), 6.93 (d, *J* = 8.4 Hz, 2H), 3.82 (s, 3H), 2.79 (t, *J* = 7.8 Hz, 2H), 2.46 (t, *J* = 7.8 Hz, 2H); ^{13}**C NMR** (101 MHz, $CDCl_3$): δ 201.25, 166.90, 161.97 (d, *J* = 246.9 Hz), 147.19, 140.40 (d, *J* = 2.5 Hz), 138.79, 138.08 (d, *J* = 3.5 Hz), 130.90 (d, *J* = 7.9 Hz), 130.52, 129.45, 128.89, 128.42, 127.74, 127.21, 115.72, 115.51, 52.01, 42.82, 28.43; ^{19}**F NMR** (376 MHz, $CDCl_3$): δ –114.63; **HRMS** (ESI) *m/z* $[M+Na]^+$ Calcd for $C_{25}H_{21}FNaO_3$: 411.1367, found: 411.1366.

methyl (*Z*)-4-(1-(4-fluorophenyl)-5-oxo-2-phenylpent-1-en-1-yl)benzoate (4j)

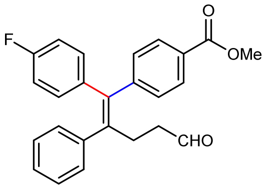

Following the General Procedure C. After concentration and purification by flash chromatography on silica gel (eluent = petroleum ether/ ethyl acetate = 5:1), the product was obtained in 52% yield (40.3 mg, light-yellow oil). ^{1}**H NMR** (400 MHz, $CDCl_3$): δ 9.60 (s, 1H), 8.04 (d, *J* = 8.3 Hz, 2H), 7.30 (d, *J* = 8.2 Hz, 2H), 7.23–7.13 (m, 3H), 7.09 (d, *J* = 7.3 Hz, 2H), 6.85–6.79 (m, 2H), 6.71 (dd, *J* = 9.4, 8.0 Hz, 2H), 3.92 (s, 3H), 2.75 (t, *J* = 7.8 Hz, 2H), 2.46 (t, *J* = 7.4 Hz, 2H); ^{13}**C NMR** (101 MHz, $CDCl_3$): δ 201.19, 166.84, 161.22 (d, *J* = 246.7 Hz), 147.53, 140.46, 139.51, 138.71, 137.67 (d, *J* = 3.4 Hz), 132.13 (d, *J* = 8.0 Hz), 129.89, 129.48, 129.30, 128.91, 128.40, 127.09, 114.63 (d, *J* = 21.3 Hz), 52.20, 42.84, 28.39; ^{19}**F NMR** (376 MHz, $CDCl_3$): δ –115.47; **HRMS** (ESI) *m/z* $[M+Na]^+$ Calcd for $C_{25}H_{21}FNaO_3$: 411.1367, found: 411.1366.

methyl (*E*)-4-(5-oxo-2-phenyl-1-(4-(trifluoromethoxy)phenyl)pent-1-en-1-yl) benzoate (4k)

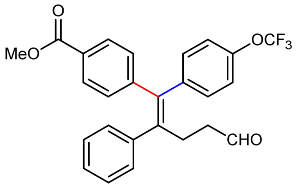

Following the General Procedure C. After concentration and purification by flash chromatography on silica gel (eluent = petroleum ether/ ethyl acetate = 5:1), the product was obtained in 51% yield (46.3 mg, light-yellow oil). **^{1}H NMR** (400 MHz, $CDCl_3$): δ 9.61 (s, 1H), 7.70 (d, *J* = 8.4 Hz, 2H), 7.27–7.15 (m, 7H), 7.07 (dd, *J* = 7.5, 1.9 Hz, 2H), 6.94 (d, *J* = 8.3 Hz, 2H), 3.83 (s, 3H), 2.80 (t, *J* = 7.7 Hz, 2H), 2.47 (t, *J* = 7.7 Hz, 2H); **^{13}C NMR** (101 MHz, $CDCl_3$): δ 201.13, 166.85, 148.33 (q, *J* = 1.9 Hz), 146.90, 140.76, 140.72, 140.18, 138.46, 130.72, 130.52, 129.42, 128.95, 128.44, 127.86, 127.30, 121.04, 120.47 (q, *J* = 257.4 Hz), 52.03, 42.73, 28.36; **^{19}F NMR** (376 MHz, $CDCl_3$): δ –57.72; **HRMS** (ESI) *m/z* $[M+Na]^+$ Calcd for $C_{26}H_{21}F_3NaO_4$: 477.1284, found: 477.1286.

methyl
(*Z*)-4-(5-oxo-2-phenyl-1-(4-(trifluoromethoxy)phenyl)pent-1-en-1-yl)benzoate (4l)

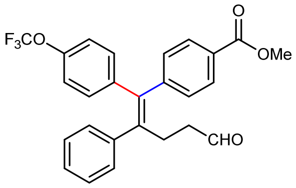

Following the General Procedure C. After concentration and purification by flash chromatography on silica gel (eluent = petroleum ether/ ethyl acetate = 5:1), the product was obtained in 51% yield (46.3 mg, light-yellow oil). **^{1}H NMR** (400 MHz, $CDCl_3$): δ 9.59 (s, 1H), 8.05 (d, *J* = 8.1 Hz, 2H), 7.31 (d, *J* = 8.2 Hz, 2H), 7.23–7.16 (m, 3H), 7.08 (d, *J* = 7.0 Hz, 2H), 6.86 (s, 4H), 3.92 (s, 3H), 2.76 (t, *J* = 7.7 Hz, 2H), 2.46 (t, *J* = 7.6 Hz, 2H); **^{13}C NMR** (101 MHz, $CDCl_3$): δ 201.08, 166.80, 147.49 (d, *J* = 1.9 Hz), 147.19, 140.28, 140.22, 140.19, 138.38, 131.85, 129.95, 129.43, 129.33, 129.03, 128.45, 127.26, 120.34 (q, *J* = 257.0 Hz), 119.98, 52.21, 42.76, 28.41; **^{19}F NMR** (376 MHz, $CDCl_3$): δ –57.85; **HRMS** (ESI) *m/z* $[M+Na]^+$ Calcd for $C_{26}H_{21}F_3NaO_4$: 477.1284, found: 477.1286.

(*E*)-5-(4-chlorophenyl)-4-phenyl-5-(*p*-tolyl)pent-4-enal (4m)

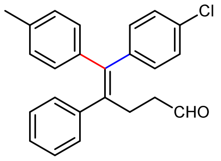

Following the General Procedure C. After concentration and purification by flash chromatography on silica gel (eluent = petroleum ether/ ethyl acetate = 9:1), the product was obtained in 39% yield (28.1 mg, light-yellow oil). **^{1}H NMR** (400 MHz, $CDCl_3$): δ 9.60 (s, 1H), 7.33 (d, *J* = 8.4 Hz, 2H), 7.23–7.06 (m, 8H), 6.83 (d, *J* = 8.0 Hz, 2H), 6.74 (d, *J* = 8.0 Hz, 2H), 2.77 (t, *J* = 7.9 Hz, 2H), 2.44 (t, *J* = 7.8 Hz, 2H), 2.19 (s, 3H); **^{13}C NMR** (101 MHz, $CDCl_3$): δ 201.54, 141.61, 141.02, 139.39, 139.00, 138.29, 135.94, 132.81, 130.61, 130.43, 129.55, 128.66, 128.32, 128.27, 126.78, 43.01, 28.51, 21.10; **HRMS** (ESI) *m/z* $[M-H]^-$ Calcd for $C_{24}H_{20}ClO$: 359.1208, found: 359.1205.

(*Z*)-4-phenyl-5-(*m*-tolyl)-5-(4-(trifluoromethyl)phenyl)pent-4-enal (4n)

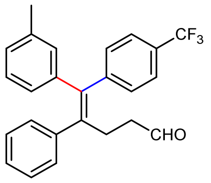

Following the General Procedure C. After concentration and purification by flash chromatography on silica gel (eluent = petroleum ether/ ethyl acetate = 9:1), the product was obtained in 47% yield (37.1 mg, light-yellow oil). **^{1}H NMR** (400 MHz, $CDCl_3$): δ 9.61 (s, 1H), 7.63 (d, *J* = 8.0 Hz, 2H), 7.37 (d, *J* = 8.0 Hz, 2H), 7.22–7.14 (m, 3H), 7.13–7.09 (m, 2H), 6.92 (t, *J* = 7.5 Hz, 1H), 6.84 (d, *J* = 7.6 Hz, 1H), 6.66 (d, *J* = 8.3 Hz, 2H), 2.76 (t, *J* = 7.8 Hz, 2H), 2.47 (t, *J* = 7.7 Hz, 2H), 2.11 (s, 3H); **^{13}C NMR** (101 MHz, $CDCl_3$): δ 201.48, 146.78, 141.57, 140.70, 139.59, 139.25, 137.25, 131.26, 129.65, 129.56, 128.31, 127.71, 127.57, 127.30, 125.55 (q, *J* = 3.8 Hz), 124.31 (q, *J* = 273.8 Hz), 42.96, 28.38, 21.35; **^{19}F NMR** (376 MHz, $CDCl_3$): δ –62.37; **HRMS** (ESI) *m/z* $[M-H]^-$ Calcd for $C_{25}H_{20}F_3O$: 393.1472, found: 393.1473.

(*Z*)-5-(4-bromophenyl)-5-(3-chlorophenyl)-4-phenylpent-4-enal (4o)

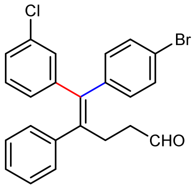

Following the General Procedure C. After concentration and purification by flash chromatography on silica gel (eluent = petroleum ether/ ethyl acetate = 9:1), the product was obtained in 52% yield (44.3 mg, light-yellow oil). **^{1}H NMR** (400 MHz, $CDCl_3$): δ 9.60 (s, 1H), 7.50 (d, *J* = 8.3 Hz, 2H), 7.20 (m, 3H), 7.09 (dd, *J* = 10.2, 7.5 Hz, 4H), 7.03–6.89 (m, 2H), 6.84 (s, 1H), 6.75–6.72 (m, 1H), 2.76 (t, *J* = 7.8 Hz, 2H), 2.45 (m, *J* = 7.5 Hz, 2H); **^{13}C NMR** (101 MHz, $CDCl_3$): δ 201.30, 143.81, 141.13, 140.32, 140.27, 138.25, 133.56, 131.91, 131.01, 130.52, 129.47, 128.90, 128.84, 128.50, 127.32, 126.58, 121.48, 42.85, 28.47; **HRMS** (ESI) *m/z* $[M+H]^+$ Calcd for $C_{23}H_{19}BrClO$: 425.0302, found: 425.0303.

Methyl (*E*)-3-(5-oxo-2-phenyl-1-(4-(trifluoromethyl)phenyl)pent-1-en-1-yl)benzoate (4p)

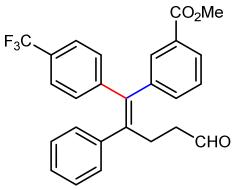

Following the General Procedure C. After concentration and purification by flash chromatography on silica gel (eluent = petroleum ether/ ethyl acetate = 5:1), the product was obtained in 43% yield (37.7 mg, light-yellow oil). **^{1}H NMR** (400 MHz, $CDCl_3$): δ 9.59 (t, *J* = 1.6 Hz, 1H), 7.99 (dd, *J* = 7.6, 1.7 Hz, 1H), 7.92 (d, *J* = 1.8 Hz, 1H), 7.46 (m, 1H), 7.42–7.39 (m, 1H), 7.27–7.25 (m, 2H), 7.19 (m, 3H), 7.10–7.06 (m, 2H), 6.97 (d, *J* = 7.6 Hz, 2H), 3.91 (s, 3H), 2.75 (t, *J* = 7.8 Hz, 2H), 2.47 (t, *J* = 7.4 Hz, 2H); **^{13}C NMR** (101 MHz, $CDCl_3$): δ 201.18, 166.94, 145.56, 142.46, 141.13, 140.16, 138.46, 133.86, 130.85, 130.70, 130.33, 129.51, 128.98, 128.66, 128.60, 127.45, 124.68 (q, *J* = 3.9 Hz), 124.15 (q, *J* = 273.67 Hz), 52.40, 42.83, 28.56; **^{19}F NMR** (376 MHz, $CDCl_3$): δ –62.40; **HRMS** (ESI) *m/z* $[M-H]^-$ Calcd for $C_{26}H_{20}F_3O_3$: 437.1370, found: 437.1364.

(1*R*,2*R*,4*S*)-1,3,3-trimethylbicyclo[2.2.1]heptan-2-yl 4-((*Z*)-1-(4-((((*S*)-2-(3-benzoylphenyl)propanoyl)oxy)methyl)phenyl)-5-oxo-2-phenylpent-1-en-1-yl)benzoate (4q)

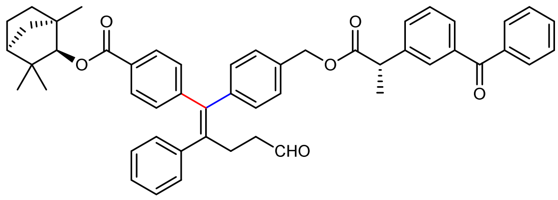

Following the General Procedure C, without using $ArB(OH)_2$. After concentration and purification by flash chromatography on silica gel (eluent = petroleum ether/ ethyl acetate = 4:1), the product was obtained in 41% yield (28.8 mg, light-yellow oil). **^{1}H NMR** (400 MHz, $CDCl_3$): δ 9.60 (s, 1H), 7.80–7.75 (m, 3H), 7.69 (t, *J* = 7.8 Hz, 3H), 7.56 (t, *J* = 6.9 Hz, 2H), 7.46 (t, *J* = 7.7 Hz, 3H), 7.25–7.14 (m, 7H), 7.09 (dd, *J* = 7.8, 1.8 Hz, 2H), 6.93 (d, *J* = 8.3 Hz, 2H), 5.18–5.08 (m, 2H), 5.01 (dt, *J* = 9.7, 2.9 Hz, 1H), 3.88 (q, *J* = 7.2 Hz, 1H), 2.77 (t, *J* = 7.8 Hz, 1H), 2.48–2.37 (m, 3H), 2.02 (ddd, *J* = 13.3, 9.4, 4.3 Hz, 1H), 1.79–1.72 (m, 1H), 1.58 (d, *J* = 7.2 Hz, 3H), 1.37–1.30 (m, 1H), 1.25–1.20 (m, 1H), 1.03 (dd, *J* = 13.8, 3.5 Hz, 1H), 0.93 (s, 3H), 0.88 (s, 3H), 0.85 (s, 3H); **^{13}C NMR** (101 MHz, $CDCl_3$): δ 201.44, 196.60, 173.94, 166.69, 147.02, 142.22, 140.69, 140.42, 140.12, 139.35, 138.33, 137.91, 137.44, 134.80, 132.59, 131.59, 130.52, 130.11, 129.49, 129.38, 129.34, 129.12, 128.83, 128.62, 128.46, 128.35, 128.13, 127.20, 117.92, 80.47, 66.29, 49.02, 47.85, 45.42, 44.94, 42.89, 36.86, 28.52, 28.05, 27.32, 19.72, 18.91, 18.43, 13.61; **HRMS** (ESI) *m/z* $[M+Na]^+$ Calcd for $C_{51}H_{50}NaO_6$: 781.3500, found: 781.3499.

ethyl (*E*)-4-(5-oxo-1-phenyl-2-(4-(trifluoromethyl)phenyl)pent-1-en-1-yl)benzoate (4r)

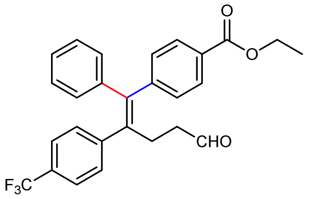

Following the General Procedure C. After concentration and purification by flash chromatography on silica gel (eluent = petroleum ether/ ethyl acetate = 5:1), the product was obtained in 32% yield (28.9 mg, light-yellow oil). **^{1}H NMR** (400 MHz, $CDCl_3$): δ 9.62 (s, 1H), 8.09–8.02 (m, 2H), 7.44 (d, *J* = 8.0 Hz, 2H), 7.34–7.29 (m,

2H), 7.23 (d, *J* = 8.0 Hz, 2H), 7.07–7.01 (m, 3H), 6.88–6.82 (m, 2H), 4.39 (q, *J* = 7.1 Hz, 2H), 2.79 (dd, *J* = 8.8, 6.8 Hz, 2H), 2.49–2.42 (m, 2H), 1.40 (t, *J* = 7.1 Hz, 3H); **^{13}C NMR** (101 MHz, $CDCl_3$): δ 200.80, 166.33, 147.05, 144.69, 141.38, 141.06, 137.71, 130.41, 129.89, 129.86, 129.41, 129.12, 128.79, 127.89, 126.85, 125.23 (q, *J* = 3.8 Hz), δ 124.04 (q, *J* = 272.1 Hz), 61.08, 42.69, 28.12, 14.37; **^{19}F NMR** (376 MHz, $CDCl_3$): δ –62.51; **HRMS** (AP-MALDI) *m/z* $[M]^+$ Calcd for $C_{27}H_{23}O_3F_3$: 452.1594, found: 452.1572.

(*Z*)-4,5-di([1,1'-biphenyl]-4-yl)-5-(4-nitrophenyl)pent-4-enal (4s)

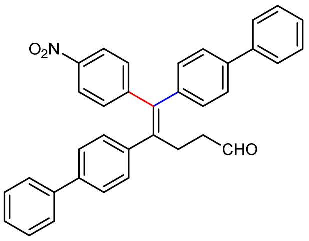

Following the General Procedure C. After concentration and purification by flash chromatography on silica gel (eluent = petroleum ether/ ethyl acetate = 5:1), the product was obtained in 41% yield (41.8 mg, yellow solid). **^{1}H NMR** (400 MHz, $CDCl_3$): δ 9.67 (s, 1H), 7.92 (d, *J* = 8.4 Hz, 2H), 7.66–7.26 (m, 16H), 7.17 (d, *J* = 8.1 Hz, 2H), 7.13 (d, *J* = 8.1 Hz, 2H), 2.92 (t, *J* = 7.7 Hz, 2H), 2.56 (t, *J* = 7.7 Hz, 2H); **^{13}C NMR** (101 MHz, $CDCl_3$): δ 201.19, 149.58, 145.89, 141.38, 140.54, 140.53, 140.38, 140.23, 140.09, 139.01, 138.68, 131.49, 129.95, 129.74, 128.90, 128.86, 127.60, 127.51, 127.21, 127.09, 126.94, 123.02, 42.91, 28.56; **HRMS** (ESI) *m/z* $[M+Na]^+$ Calcd for $C_{35}H_{27}NNaO_3$: 532.1883, found: 532.1883.

(*E*)-4-([1,1'-biphenyl]-4-yl)-5-(4-chlorophenyl)-5-(4-nitrophenyl)pent-4-enal (4t)

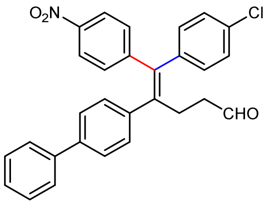

Following the General Procedure C. After concentration and purification by flash chromatography on silica gel (eluent = petroleum ether/ ethyl acetate = 5:1), the product was obtained in 42% yield (39.2 mg, light-yellow oil). **^{1}H NMR** (400 MHz, $CDCl_3$): δ 9.65 (s, 1H), 7.90 (d, *J* = 8.8 Hz, 2H), 7.55 (d, *J* = 7.4 Hz, 2H), 7.47–7.34

(m, 7H), 7.18 (d, J = 8.4 Hz, 2H), 7.14 (d, J = 8.2 Hz, 2H), 7.06 (d, J = 8.8 Hz, 2H), 2.84 (t, J = 8.0 Hz, 2H), 2.52 (t, J = 7.7 Hz, 2H); **^{13}C NMR** (101 MHz, $CDCl_3$): δ 200.90, 149.15, 146.07, 141.95, 140.47, 140.10, 140.07, 138.75, 137.83, 133.82, 131.43, 130.75, 129.91, 129.21, 128.93, 127.71, 127.31, 127.00, 123.12, 42.73, 28.49; **HRMS** (ESI) *m/z* $[M+Na]^+$ Calcd for $C_{29}H_{22}ClNNaO_3$: 490.1180, found: 490.1185.

methyl (*E*)-4-(1-(4-chlorophenyl)-5-methoxy-5-oxo-2-phenylpent-1-en-1-yl)benzoate (4u)

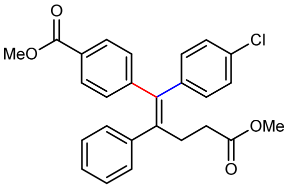

Following the General Procedure C. After concentration and purification by flash chromatography on silica gel (eluent = petroleum ether/ ethyl acetate = 5:1), the product was obtained in 47% yield (40.8 mg, light-yellow oil). **^{1}H NMR** (400 MHz, $CDCl_3$): δ 7.69 (d, J = 8.3 Hz, 2H), 7.35 (d, J = 8.4 Hz, 2H), 7.20–7.05 (m, 7H), 6.92 (d, J = 8.3 Hz, 2H), 3.82 (s, 3H), 3.56 (s, 3H), 2.80 (t, J = 7.7 Hz, 2H), 2.31 (t, J = 7.7 Hz, 2H); **^{13}C NMR** (101 MHz, $CDCl_3$): δ 173.20, 166.90, 147.07, 140.81, 140.63, 140.26, 138.59, 133.19, 130.70, 130.59, 129.48, 128.89, 128.80, 128.32, 127.75, 127.16, 52.02, 51.63, 33.01, 31.10; **HRMS** (ESI) *m/z* $[M+Na]^+$ Calcd for $C_{26}H_{23}ClNaO_4$: 457.1177, found: 457.1178.

(*E*)-5,6-diphenyl-6-(4-(trifluoromethyl)phenyl)hex-5-en-2-one (4v)

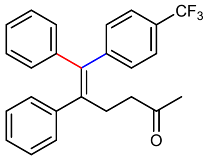

Following the General Procedure C. After concentration and purification by flash chromatography on silica gel (eluent = petroleum ether/ ethyl acetate = 9:1), the product was obtained in 53% yield (41.6 mg, white solid). **^{1}H NMR** (400 MHz, $CDCl_3$): δ 7.61 (d, J = 8.0 Hz, 2H), 7.37 (d, J = 7.9 Hz, 2H), 7.16 (m, 3H), 7.10 (dd, J = 7.9, 1.7 Hz, 2H), 7.05–6.81 (m, 5H), 2.71 (t, J = 7.9 Hz, 2H), 2.44 (t, J = 7.9 Hz, 2H), 2.00 (s, 3H); **^{13}C NMR** (101 MHz, $CDCl_3$): δ 207.90, 146.74, 141.82, 140.77, 139.92, 139.05, 130.56, 129.64, 129.53, 128.19, 127.65, 126.87, 126.35, 125.41 (q, J

= 3.7 Hz), 124.22 (q, J = 271.9 Hz), 42.42, 29.92, 29.73; **^{19}F NMR** (376 MHz, $CDCl_3$): δ –62.38; **HRMS** (ESI) *m/z* $[M+Na]^+$ Calcd for $C_{25}H_{21}F_3NaO$: 417.1437, found: 417.1439.

methyl (*E*)-4-(5-oxo-1,2-diphenylpent-1-en-1-yl)benzoate (4w)

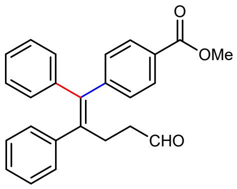

Following the General Procedure C. After concentration and purification by flash chromatography on silica gel (eluent = petroleum ether/ ethyl acetate = 5:1), the product was obtained in 54% yield (39.9 mg, light-yellow oil). **^{1}H NMR** (400 MHz, $CDCl_3$): δ 9.60 (s, 1H), 8.04 (d, J = 8.3 Hz, 2H), 7.32 (d, J = 6.6 Hz, 2H), 7.20–7.13 (m, 3H), 7.10 (dd, J = 8.0, 1.7 Hz, 2H), 7.05–6.80 (m, 5H), 3.92 (s, 3H), 2.76 (t, J = 7.7 Hz, 2H), 2.46 (t, J = 7.7 Hz, 2H); **^{13}C NMR** (101 MHz, $CDCl_3$): δ 201.32, 166.91, 147.79, 141.68, 140.64, 139.83, 139.18, 130.53, 129.82, 129.54, 129.33, 128.76, 128.26, 127.64, 126.96, 126.39, 52.18, 42.92, 28.40; **HRMS** (ESI) *m/z* $[M+Na]^+$ Calcd for $C_{25}H_{22}NaO_3$: 393.1461, found: 393.1465.

2.1.4. Synthetic Procedures and Product Characterization of By-Products

dimethyl 4,4'-(2-phenylpent-1-ene-1,3-diyl)dibenzoate (3a'')

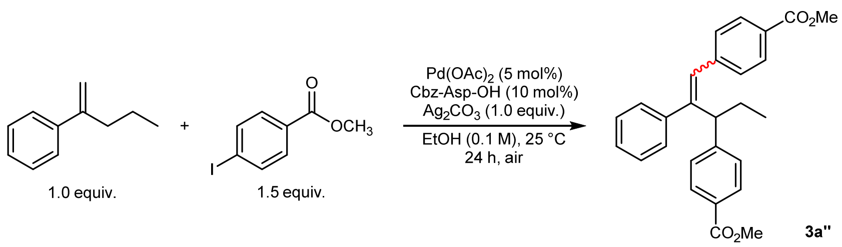

Following the General Procedure A. After concentration and purification by flash chromatography on silica gel (eluent = petroleum ether/ ethyl acetate = 10:1), the product was obtained in 20% yield (16.5 mg, colorless oil). **^{1}H NMR** (400 MHz, $CDCl_3$): δ 7.93 (d, *J* = 8.3 Hz, 2H), 7.72 (d, *J* = 8.4 Hz, 2H), 7.24–7.16 (m, 5H), 6.90 (d, *J* = 8.4 Hz, 2H), 6.78 (dd, *J* = 7.8, 1.8 Hz, 2H), 6.60 (s, 1H), 3.90 (s, 3H), 3.83 (s, 3H), 3.64 (t, *J* = 7.6 Hz, 1H), 2.01 (dt, *J* = 14.1, 7.1 Hz, 1H), 1.87 (dt, *J* = 14.4, 7.4 Hz, 1H), 0.96 (t, *J* = 7.3 Hz, 3H); **^{13}C NMR** (101 MHz, $CDCl_3$): δ 167.14, 166.92, 147.97, 147.81, 141.82, 139.91, 129.56, 129.14, 128.98, 128.77, 128.51, 128.44, 128.35, 127.78, 127.27, 126.57, 57.17, 52.06, 51.98, 26.14, 12.56; **HRMS** (ESI) *m/z* $[M+Na]^+$ Calcd for $C_{27}H_{26}NaO_4$: 437.1723, found: 437.1724.

methyl 4-((1*E*,3*E*)-5-oxo-2-phenylpenta-1,3-dien-1-yl)benzoate (6a)

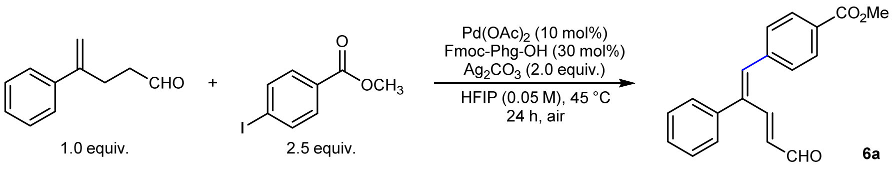

An oven-dried 35 mL sealed tube with previously placed magnetic stir-bar was charged with methyl 4-iodobenzoate (131 mg, 0.5 mmol, 2.5 equiv), $Pd(OAc)_2$ (4.5 mg, 0.02 mmol, 10 mol%), Fmoc-Phg-OH (22.4 mg, 0.06 mmol, 30 mol%), Ag_2CO_3 (110 mg, 0.4 mmol, 2 equiv.), followed by addition of HFIP (4 mL) and 4-phenylpent-4-enal (32.04 mg, 0.2 mmol). The reaction mixture was stirred vigorously on a hotplate at 45 °C for 24 hours. After completion of the reaction, the resultant solution was filtered through a short pad of 1:1 mixture of Celite and silica gel, and the column was washed with ethyl acetate. After concentration and purification by flash chromatography on silica gel (eluent: petroleum ether/ ethyl acetate = 10:1), the product was obtained in 8% yield (4.7 mg, white solid). **^{1}H NMR**

(600 MHz, $CDCl_3$): δ 9.66 (dd, J = 7.8, 1.5 Hz, 1H), 8.12–8.08 (m, 2H), 7.73 (dd, J = 15.7, 1.1 Hz, 1H), 7.45–7.39 (m, 5H), 7.35–7.32 (m, 2H), 7.25 (d, J = 1.5 Hz, 1H), 7.01 (s, 1H), 6.20 (ddd, J = 15.7, 7.8, 1.5 Hz, 1H), 3.95 (s, 3H); **^{13}C NMR** (151 MHz, $CDCl_3$): δ 194.07, 166.69, 149.01, 141.06, 140.59, 139.62, 137.86, 134.18, 129.94, 129.80, 128.99, 128.76, 128.61, 52.43; **HRMS** (ESI) *m/z* $[M+Na]^+$ Calcd for $C_{19}H_{16}NaO_3$: 315.0992, found: 315.0990.

2.2. Control Experiments for Mechanistic Study

2.2.1. Parallel Model Reactions Employing a Smaller Amount of Aryl Iodide 2a

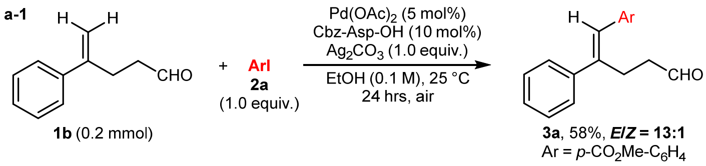

Control Experiment a-1: To an oven-dried 35 mL Schlenk tube with previously placed magnetic stir-bar were added methyl 4-iodobenzoate **2a** (52.4 mg, 0.2 mmol, 1.0 equiv.), $Pd(OAc)_2$ (2.2 mg, 0.01 mmol, 5 mol%), Cbz-Asp-OH (5.3 mg, 0.02 mmol, 10 mol%), Ag_2CO_3 (55 mg, 0.2 mmol, 1 equiv.), followed by addition of EtOH (2 mL) and 4-phenylpent-4-enal **1b** (32 mg, 0.2 mmol). The tube was sealed with a screw cap and the reaction mixture was stirred vigorously at room temperature (25 °C). After stirring for 24 hours, the resultant solution was filtered through a short pad of 1:1 mixture of Celite and silica gel, and the column was washed with ethyl acetate (15 mL). The combined organic solutions were concentrated under reduced pressure to afford the crude reaction mixture; the yield and *E*/*Z* ratio were calculated based on ^{1}H NMR analysis using 1,1,2,2-tetrachloroethane as the internal standard.

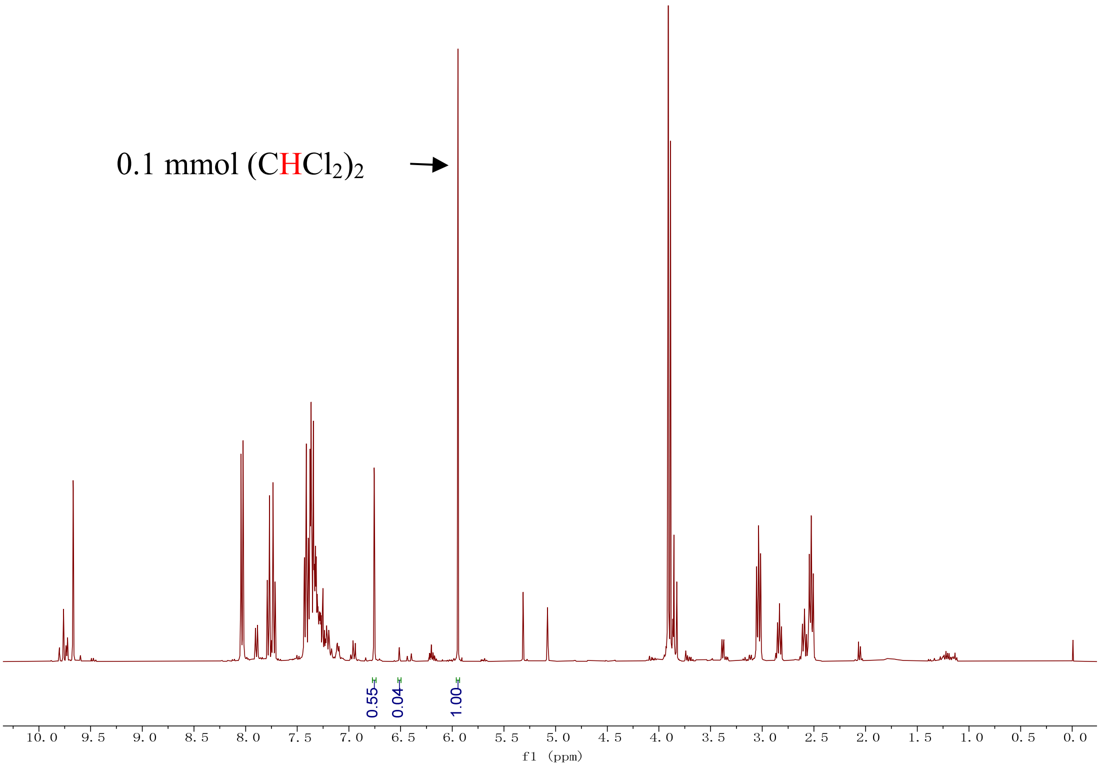

Supplementary Figure 5. ^{1}H NMR (400 MHz, $CDCl_3$) of control experiment a-1

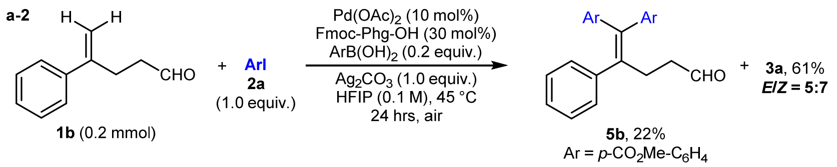

Control Experiment a-2: To an oven-dried 35 mL Schlenk tube with previously placed magnetic stir-bar were added methyl 4-iodobenzoate **2a** (52.4 mg, 0.2 mmol, 1.0 equiv.), 4-methoxybenzoborate (7.2 mg, 0.04 mmol, 0.2 equiv.), $Pd(OAc)_2$ (4.5 mg, 0.02 mmol, 10 mol%), Fmoc-Phg-OH (22.4 mg, 0.06 mmol, 30 mol%), Ag_2CO_3 (55 mg, 0.2 mmol, 1.0 equiv.), followed by addition of HFIP (2 mL) and 4-phenylpent-4-enal **1b** (32 mg, 0.2 mmol). The tube was sealed with a screw cap and the reaction mixture was stirred vigorously on a hotplate at 45 °C for 24 hours. After completion of the reaction, the resultant solution was filtered through a short pad of 1:1 mixture of Celite and silica gel, and the column was washed with ethyl acetate (15 mL). The combined organic solutions were concentrated under reduced pressure to afford the crude reaction mixture; the yield and *E*/*Z* ratio were calculated based on ^1H NMR analysis using 1,1,2,2-tetrachloroethane as the internal standard.

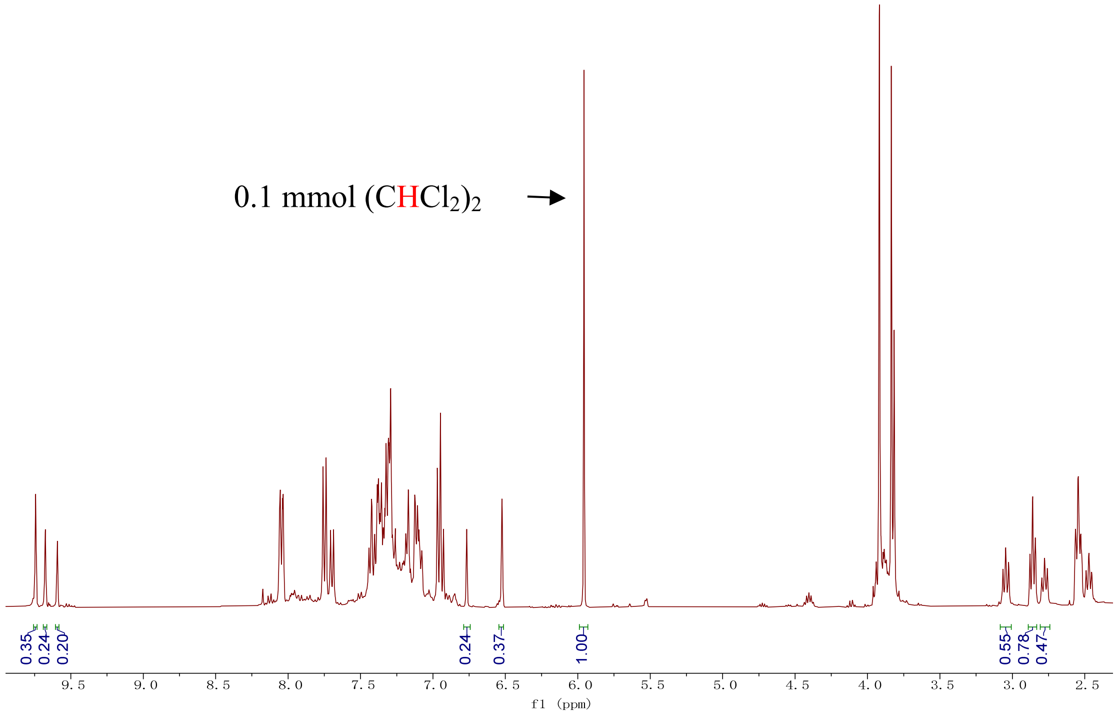

Supplementary Figure 6. ^1H NMR (400 MHz, $CDCl_3$) of control experiment a-2

2.2.2. *E*/*Z* Isomerization of the Internal Alkene 3b

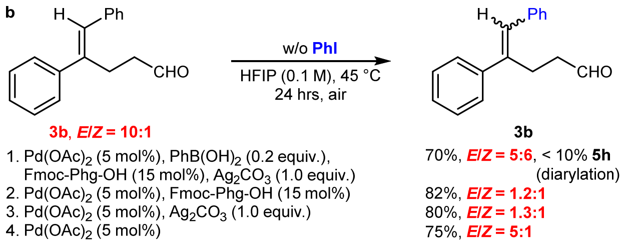

Control Experiment b: To an oven-dried 35 mL Schlenk tube with previously placed magnetic stir-bar were added **1**. $Pd(OAc)_2$ (2.2 mg, 0.01 mmol, 5 mol%), phenylboronic acid (4.9 mg, 0.04 mmol, 0.2 equiv.), Fmoc-Phg-OH (11.2 mg, 0.03 mmol, 15 mol%), Ag_2CO_3 (55 mg, 0.2 mmol, 1 equiv.); **2.** $Pd(OAc)_2$ (2.2 mg, 0.01 mmol, 5 mol%), Fmoc-Phg-OH (11.2 mg, 0.03 mmol, 15 mol%); **3.** $Pd(OAc)_2$ (2.2 mg, 0.01 mmol, 5 mol%), Ag_2CO_3 (55 mg, 0.2 mmol, 1 equiv.); **4**. $Pd(OAc)_2$ (2.2 mg, 0.01 mmol, 5 mol%); followed by addition of **3b** (47 mg, 0.2 mmol, obtained from the **General Procedure A**) which dissolved in 2 ml HFIP. The tube was sealed with a screw cap and the reaction mixture was stirred vigorously on a hotplate at 45 °C for 24 hours. After completion of the reaction, the resultant solution was filtered through a short pad of 1:1 mixture of Celite and silica gel, and the column was washed with ethyl acetate (15 mL). The combined organic solutions were concentrated under reduced pressure to afford the crude reaction mixture; the yield and *E*/*Z* ratio were calculated based on ^{1}H NMR analysis of the crude reaction mixture using 1,1,2,2-tetrachloroethane as the internal standard.

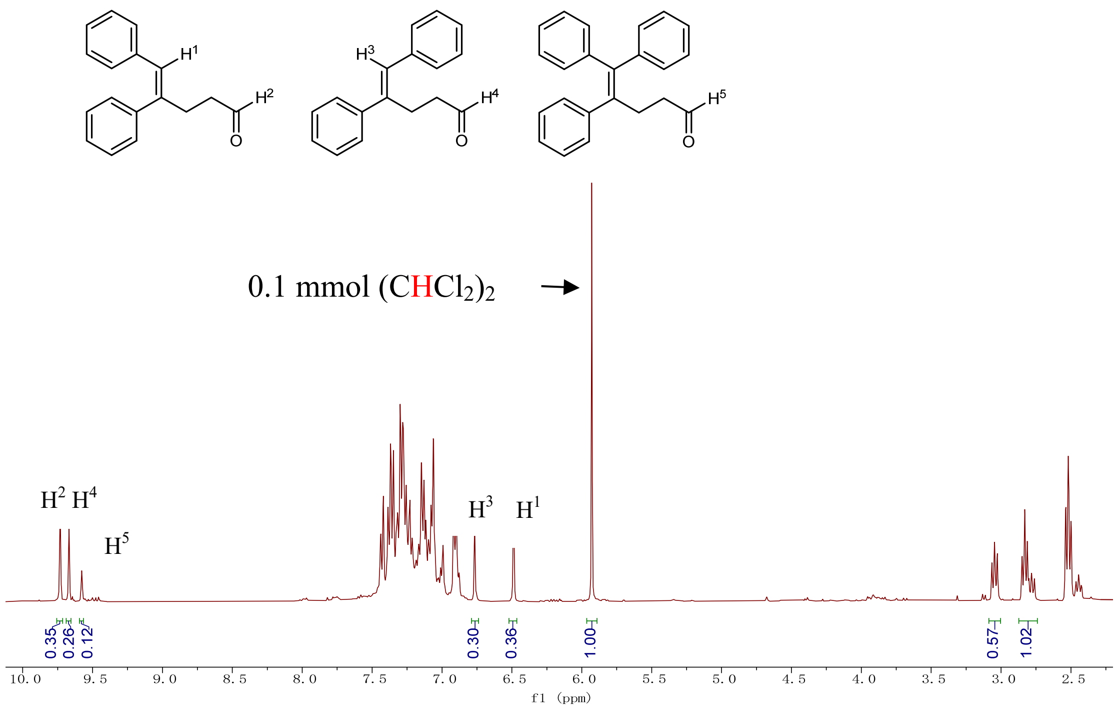

Supplementary Figure 7. ^{1}H NMR (400 MHz, $CDCl_3$) of control experiment b-1

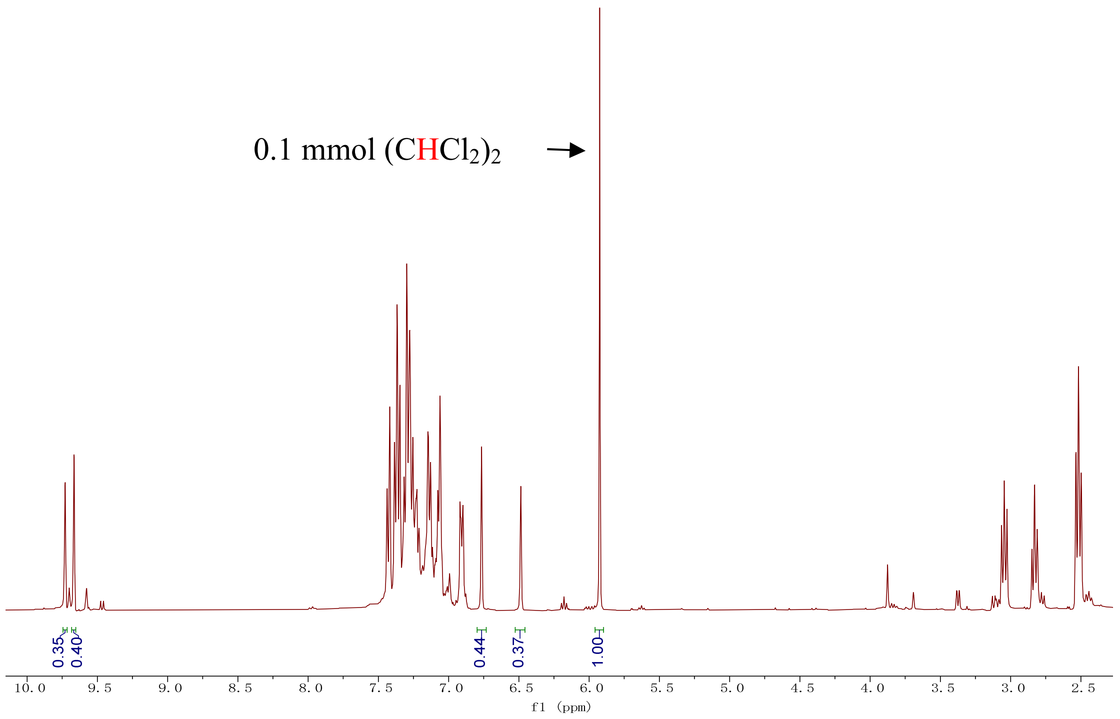

Supplementary Figure 8. ^{1}H NMR (400 MHz, $CDCl_3$) of control experiment b-2

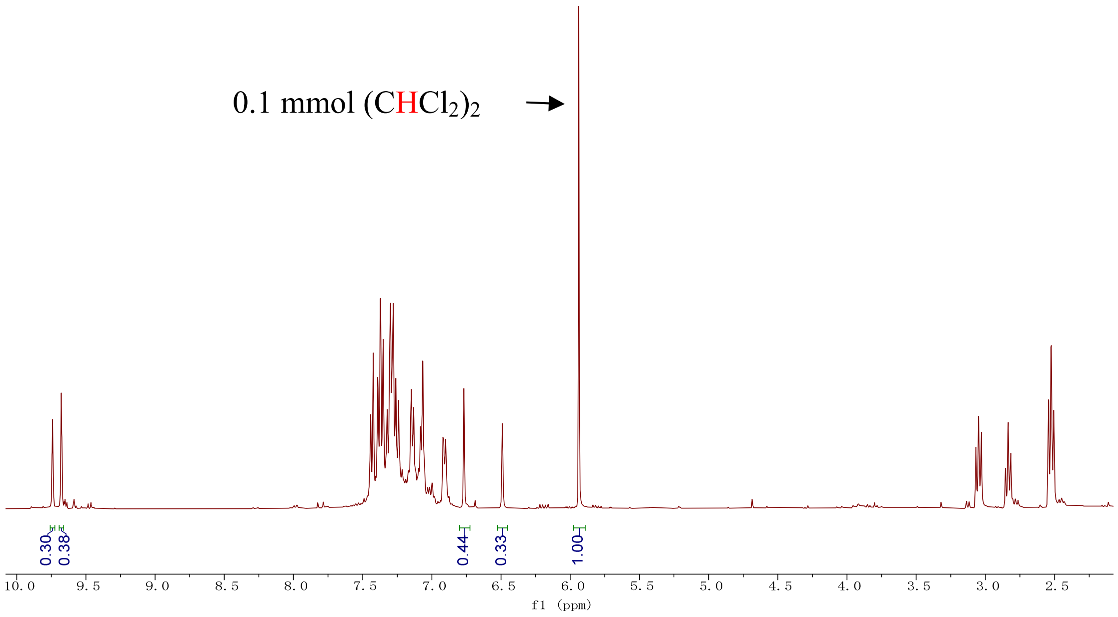

Supplementary Figure 9. ^{1}H NMR (400 MHz, $CDCl_3$) of control experiment b-3

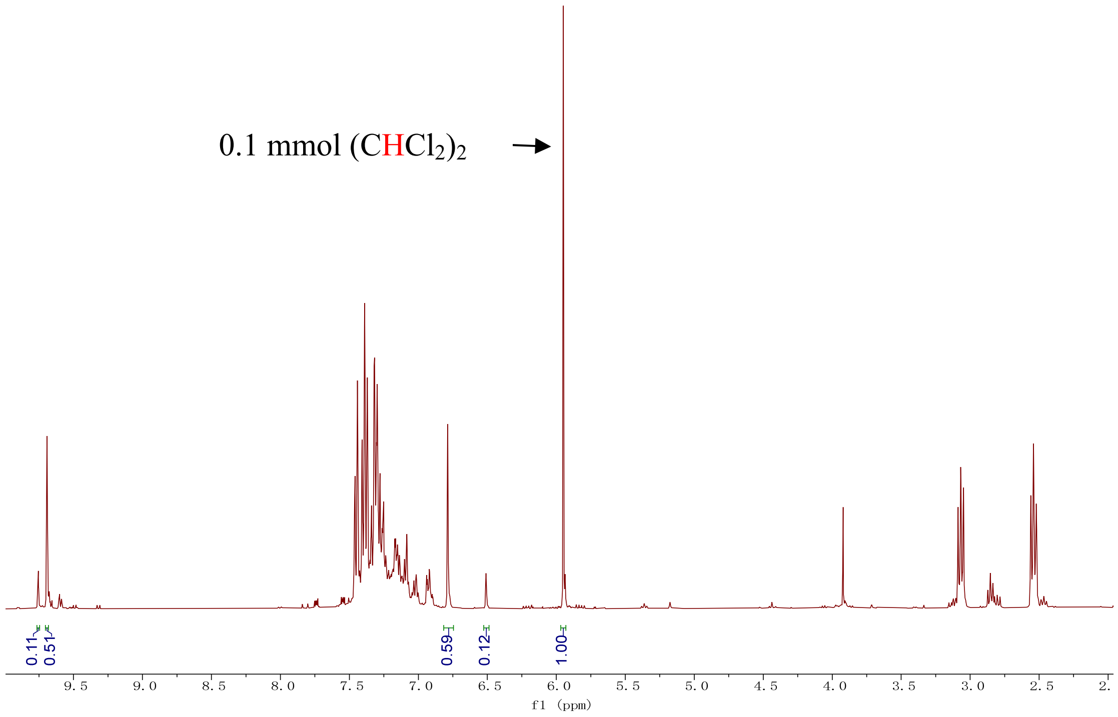

Supplementary Figure 10. ^{1}H NMR (400 MHz, $CDCl_3$) of control experiment b-4

2.2.3. The Second Arylation of the Mixed Isomers 3b

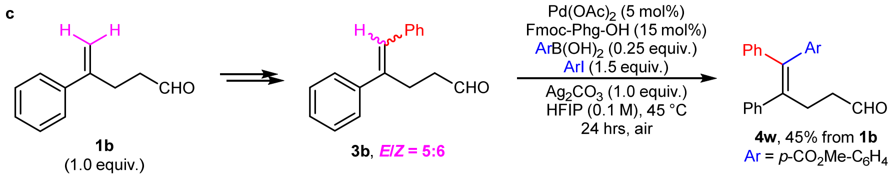

Control Experiment c: To an oven-dried 35 mL Schlenk tube with previously placed magnetic stir-bar were added methyl 4-iodobenzoate **2a** (78.6 mg, 0.3 mmol, 1.5 equiv.), 4-methoxybenzoborate (9.0 mg, 0.05 mmol, 0.25 equiv.), $Pd(OAc)_2$ (2.2 mg, 0.01 mmol, 5 mol%), Fmoc-Phg-OH (11.2 mg, 0.03 mmol, 15 mol%), Ag_2CO_3 (55 mg, 0.2 mmol, 1.0 equiv.), followed by addition of **3b** (47 mg, 0.2 mmol, obtained from the **Control Experiment b-1**) which dissolved in 2ml HFIP. The tube was sealed with a screw cap and the reaction mixture was stirred vigorously on a hotplate at 45 °C for 24 hours. After completion of the reaction, the resultant solution was filtered through a short pad of 1:1 mixture of Celite and silica gel, and the column was washed with ethyl acetate (15 mL). The combined organic solutions were concentrated under reduced pressure to afford the crude reaction mixture; the yield was calculated based on ^{1}H NMR analysis of the crude reaction mixture using 1,1,2,2-tetrachloroethane as the internal standard.

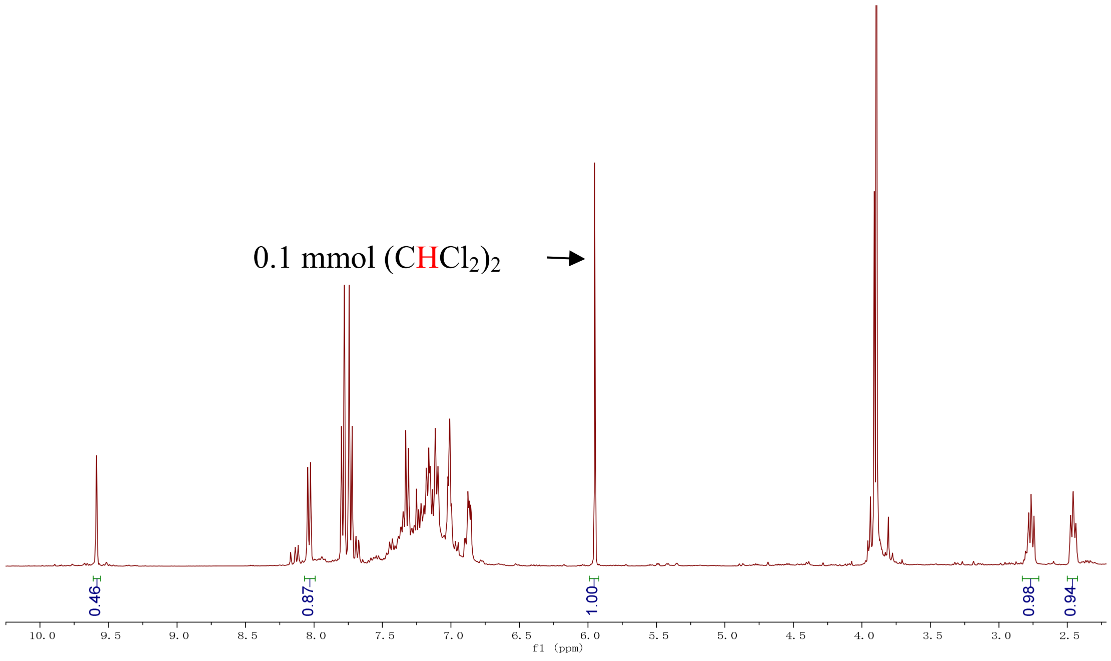

Supplementary Figure 11. ^{1}H NMR (400 MHz, $CDCl_3$) of control experiment c

2.2.4. Deuterium KIE Studies

Synthesis of methyl 4-phenylpent-4-enoate-2,2,5,5-*d*4 ([D4]-1a)

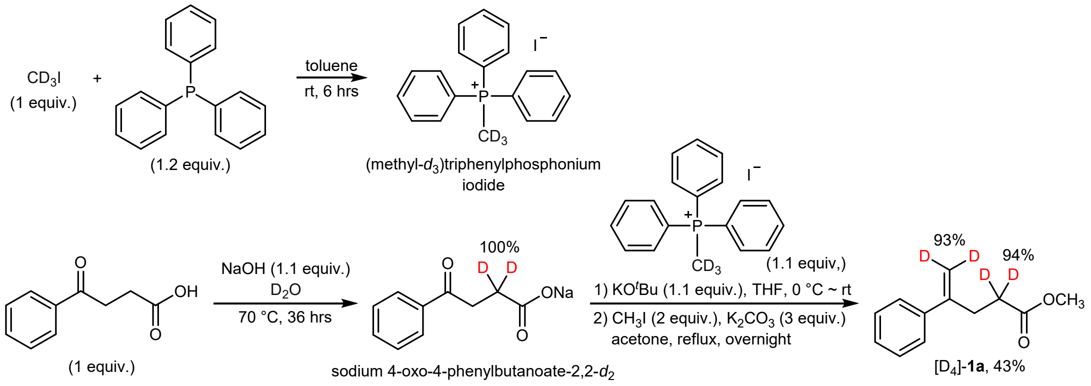

(Methyl-*d*3)triphenylphosphonium iodide was prepared according to previously reported literature procedure[14]. An oven-dried 50 mL round bottom flask was equipped with a condenser. CD_3I (1.0 g, 6.9 mmol, 1.0 equiv., ≥99.5 atom% D) was added to a solution of PPh_3 (2.17 g, 8.27 mmol, 1.2 equiv.) in dry PhMe (15 mL) at ambient temperature. The resulting mixture was vigorously stirred for 6 hours. The precipitate was collected by filtration, washed with PhMe (3 × 5 mL) and hexane (3 × 5 mL) and dried in vacuo to provide (methyl-*d*3)triphenylphosphonium iodide (2.86 g, 99 %) as a white powder. The deuterium content was confirmed by ^{1}H NMR spectroscopic analysis. **^{1}H NMR** (400 MHz, CD_3Cl): δ 7.98–7.57 (m, 15H). **^{13}C NMR** (101 MHz, CD_3Cl): δ 135.29, 135.26, 133.43, 133.32, 130.62, 130.50, 119.31, 118.42.

Sodium 4-oxo-4-phenylbutanoate-2,2-*d*2

An oven-dried 50 mL round bottom flask was equipped with a condenser. The flask was charged with 4-oxo-4-phenylbutanoic acid (3 g, 16.83 mmol), NaOH (727 mg, 18.18 mmol), protected with nitrogen before 10 mL D_2O was added via syringe. The reaction mixture was stirred at 70 °C for 36 hours and allowed to cool to room temperature. Solvent was removed in vacuo (protected with nitrogen atmosphere) to provide the crude product (3.3 g, 98%) as a white solid in more than 99% deuterated. **^{1}H NMR** (500 MHz, D_2O): δ 7.95 (dd, J = 8.3, 1.3 Hz, 2H), 7.65 (t, J = 7.5 Hz, 1H), 7.52 (t, J = 7.5 Hz, 2H), 2.51 (s, 2H). **^{13}C NMR** (126 MHz, D_2O): δ 204.38, 181.65, 136.24, 133.82, 128.76, 128.10, 34.39 (m), 31.40.

Methyl 4-phenylpent-4-enoate-2,2,5,5-*d*4 ([D_4]-1a)

To a suspension of (Methyl-*d*3)triphenylphosphonium iodide (4.48 g, 11.0 mmol) in THF (100 mL) was added potassium *tert*-butoxide (1.23 g, 11.0 mmol) at 0 °C under nitrogen atmosphere. The mixture was stirred at room temperature for 2 hours. Sodium 4-oxo-4-phenylbutanoate-2,2-*d*2 (2 g, 10.0 mmol) was added at 0°C and the reaction was stirred at room temperature for 5 hours. Solvent was removed in vacuo and the residue was diluted with DCM (50 mL) and aqueous NaOH solution (1 M, 100 mL). The aqueous layer was separated, washed with dichloromethane (50 × 2 mL), acidified to pH = 1 with concentrated HCl and extracted with DCM (50 mL × 3). The combined organic layers were dried over anhydrous Na_2SO_4 and filtered, concentrated under reduced pressure to give the crude product as a white solid. The crude product and K_2CO_3 (4.14 g, 30.0 mmol) were placed in a 250 mL round bottom flask. Acetone (100 mL) and iodomethane (2.83 g, 1.24 mL, 20 mmol) were added dropwise, and the mixture was heated at 70 °C overnight. After cooling to room temperature, solvent was removed in vacuo and 50 mL water was added. The mixture was extracted with EtOAc (50 mL × 3), the combined organic layers were dried over anhydrous Na_2SO_4 and filtered, concentrated under reduced pressure. The residue was purified by flash chromatography on a silica gel (PE:EA = 10:1) to provide methyl 4-phenylpent-4-enoate-2,2,5,5-*d*4 ([D_4]-**1a**) (0.83 g, 4.3 mmol, 43%) as a colorless oil. **^{1}H NMR** (400 MHz, $CDCl_3$): δ 7.31 (d, J = 7.8 Hz, 2H), 7.27–7.21 (m, 2H), 7.20–7.15 (m, 1H), 3.56 (s, 3H), 2.38 (s, 2H). **^{13}C NMR** (101 MHz, $CDCl_3$): δ 173.54, 146.64, 140.48, 128.41, 127.62, 126.09, 112.17 (m), 51.57, 32.93, 29.69 (m).

KIE experiments in HFIP with completely parallel settings:

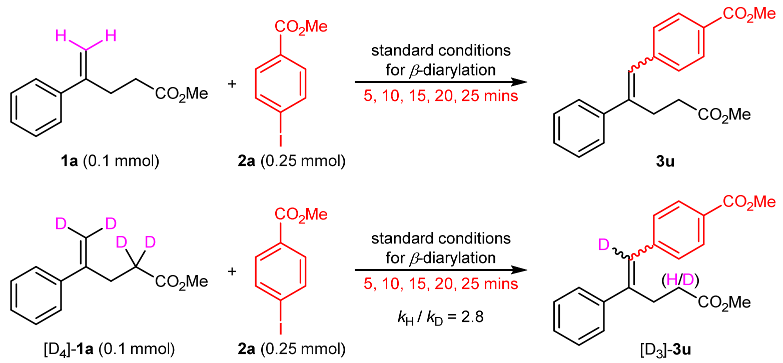

Following the General Procedure B, the reaction mixture was heated at 45 °C for 5 to 25 min. After immediately cooling in a dry ice/acetone bath, the mixture was filtered

through a short pad of 1:1 mixture of Celite and silica gel, washed with EtOAc and concentrated, the yield of mono-arylated product was determined by crude ^{1}H NMR using 1,1,2,2-tetrachloroethane as the internal standard. The obtained average yields for three trials were plotted as yield (product) vs. time (min) (Supplementary Fig. 5).

t/min \ yield	5	10	15	20	25
**3u**	1.6%	3%	4%	5.3%	6.3%
[D_3]-**3u**	1.2%	1.5%	2.3%	2.5%	2.8%

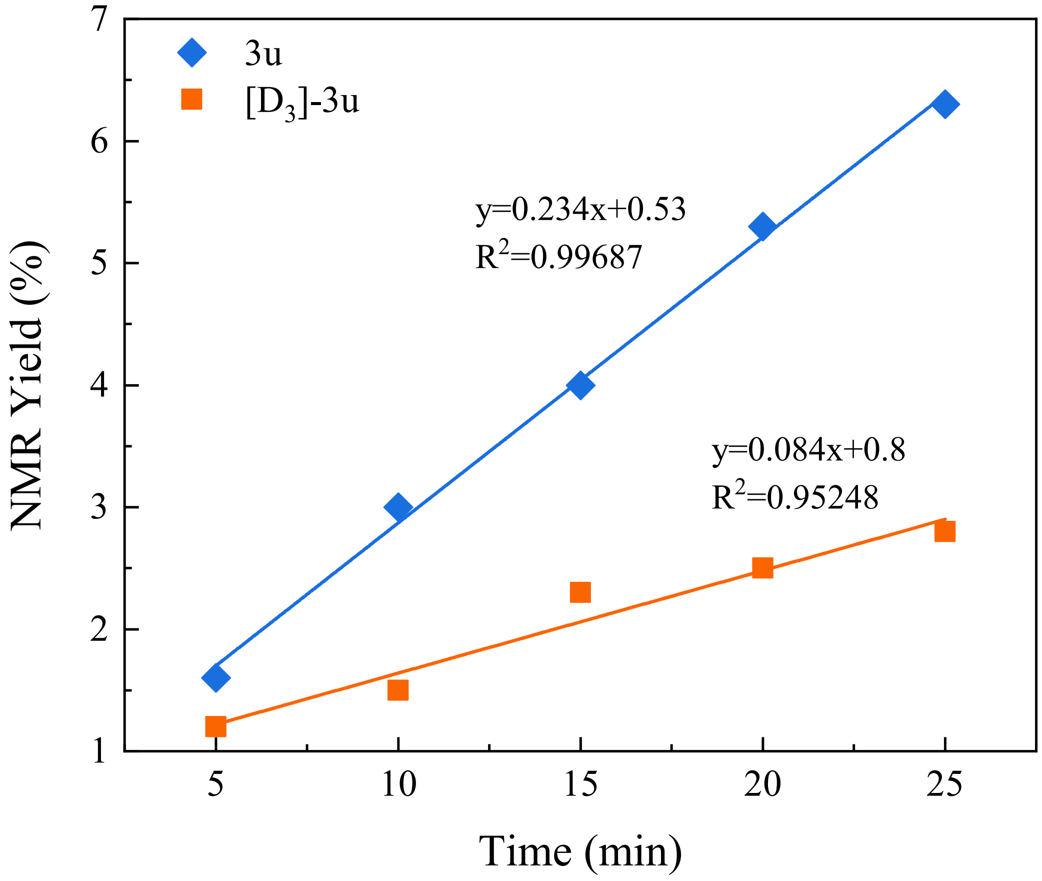

Supplementary Figure 12. The reaction of **1a** and deuterated-**1a** with **2a**

The KIE value was determined to be 2.8, this data shown that the C–H cleavage step is possible the rate-determined step for the arylation reaction occurred in HFIP.

Determination of intermolecular isotope effect in EtOH:

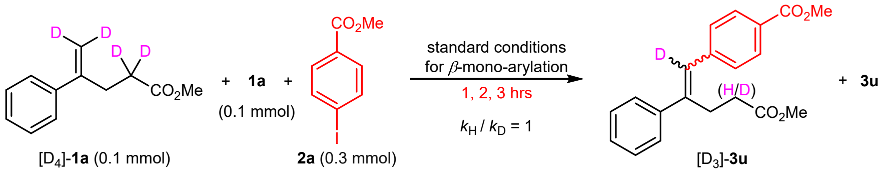

Following the General Procedure A, the reaction mixture of methyl 4-iodobenzoate **2a** (79 mg, 0.3 mmol, 1.5 equiv.), $Pd(OAc)_2$ (2.2 mg, 0.01 mmol, 5 mol%), Cbz-Asp-OH (5.3 mg, 0.02 mmol, 10 mol%), Ag_2CO_3 (55 mg, 0.2 mmol, 1 equiv.), a solution of [D_4]-**1a** (19.4 mg, 0.1 mmol, 0.5 equiv.) and **1a** (19 mg, 0.1 mmol, 0.5 equiv.) in EtOH (2 mL) was stirred vigorously at 30 °C for 1 to 3 hours. After immediately cooling in a dry ice/acetone bath, the mixture was filtered through a short pad of 1:1 mixture of Celite and silica gel, washed with EtOAc and concentrated, the yield of mono-arylated product was determined by crude ^{1}H NMR using 1,1,2,2-tetrachloroethane as the internal standard. At each time node, the amount of mono-arylated product [D_3]-**3u** formed comes from the deuterated substrate [D_4]-**1a** is approximately equal to the amount of **3u** derived from **1a**, indicating a value of k_H/k_D ≈ 1.

t/hrs / yield	1	2	3
**3u**	1.1%	5.0%	11%
[D_3]-**3u**	1.2%	5.0%	10%

methyl (*E*)-4-(5-methoxy-5-oxo-2-phenylpent-1-en-1-yl)benzoate (3u)

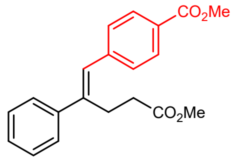

Following the General Procedure A. After concentration and purification by flash chromatography on silica gel (eluent = petroleum ether/ ethyl acetate = 5:1), the product (**3u**, 82% yield, determined by crude NMR) was obtained as a mixture with double-bond-migrated product (**3u'**, 12% yield, determined by crude NMR). **^{1}H NMR** (400 MHz, $CDCl_3$): δ 8.05 (d, J = 8.1 Hz, 2H), 7.45 (d, J = 7.2 Hz, 2H), 7.38 (m, 4H), 7.33 (d, J = 7.2 Hz, 1H), 6.75 (s, 1H), 3.92 (s, 3H), 3.59 (s, 3H), 3.06 (t, J = 7.6 Hz, 2H), 2.40 (t, J = 7.6 Hz, 2H); **^{13}C NMR** (101 MHz, $CDCl_3$): δ 173.12, 166.89,

142.87, 142.43, 141.51, 129.75, 128.68, 128.64, 128.54, 127.87, 126.68, 52.10, 51.63, 33.00, 25.77; **HRMS** (ESI) *m/z* $[M+H]^+$ Calcd for $C_{20}H_{21}O_4$: 325.1434, found: 325.1437.

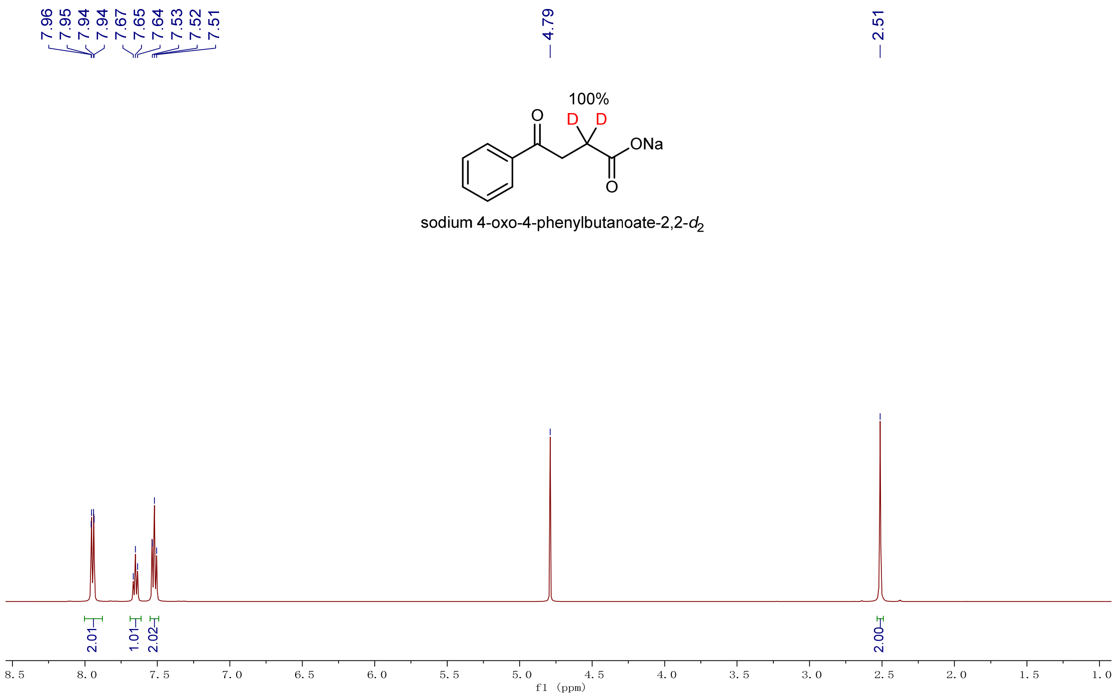

Supplementary Figure 13. ^{1}H NMR (500 MHz, D_2O) of Sodium 4-oxo-4-phenylbutanoate-2,2-*d*2

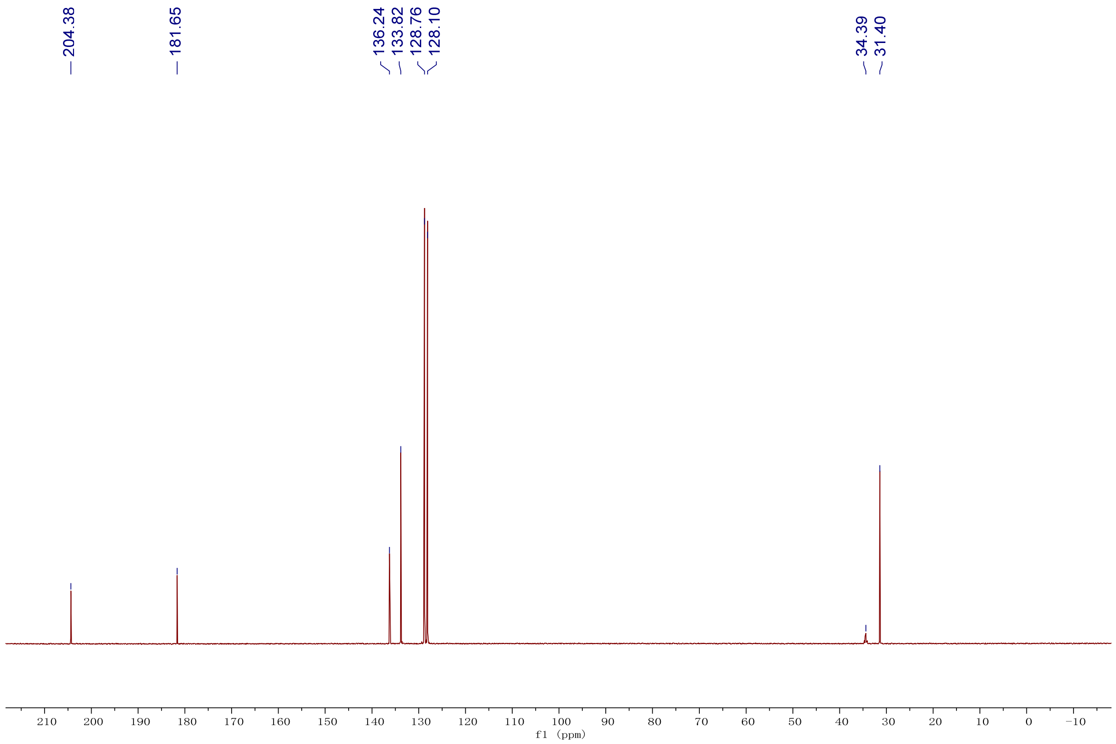

Supplementary Figure 14. ^{13}C NMR (126 MHz, D_2O) of Sodium 4-oxo-4-phenylbutanoate-2,2-*d*2

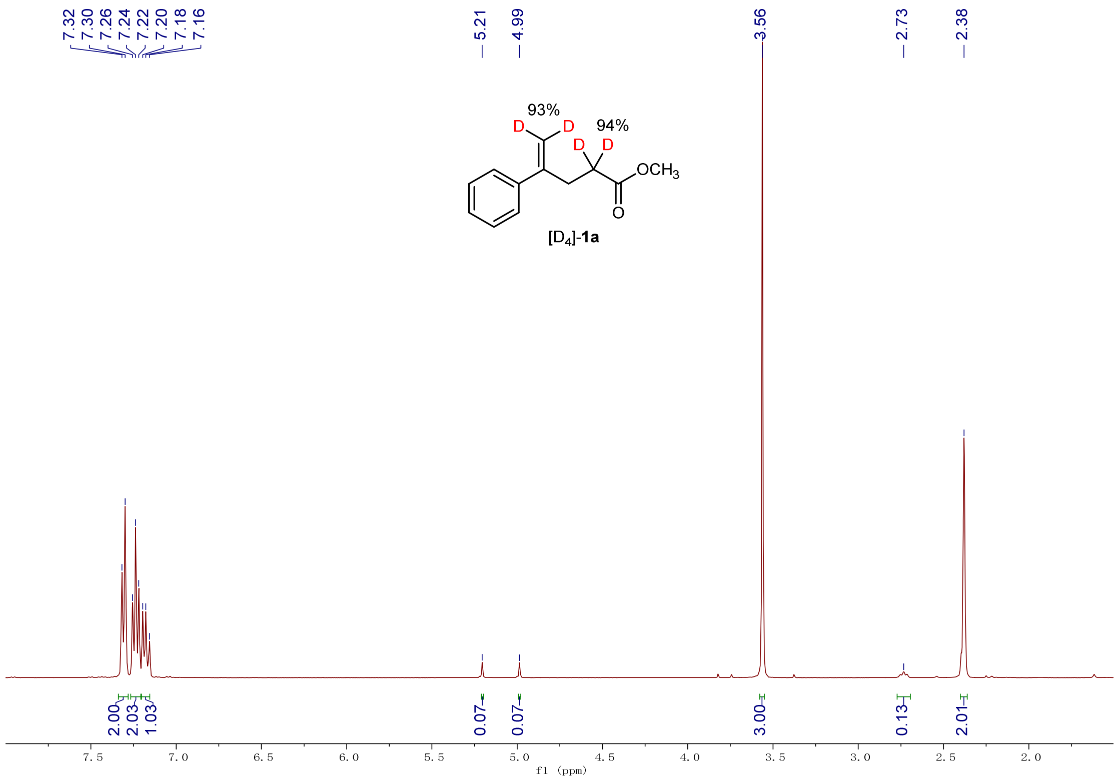

Supplementary Figure 15. ^{1}H NMR (400 MHz, $CDCl_3$) of **[D$_4$]-1a**

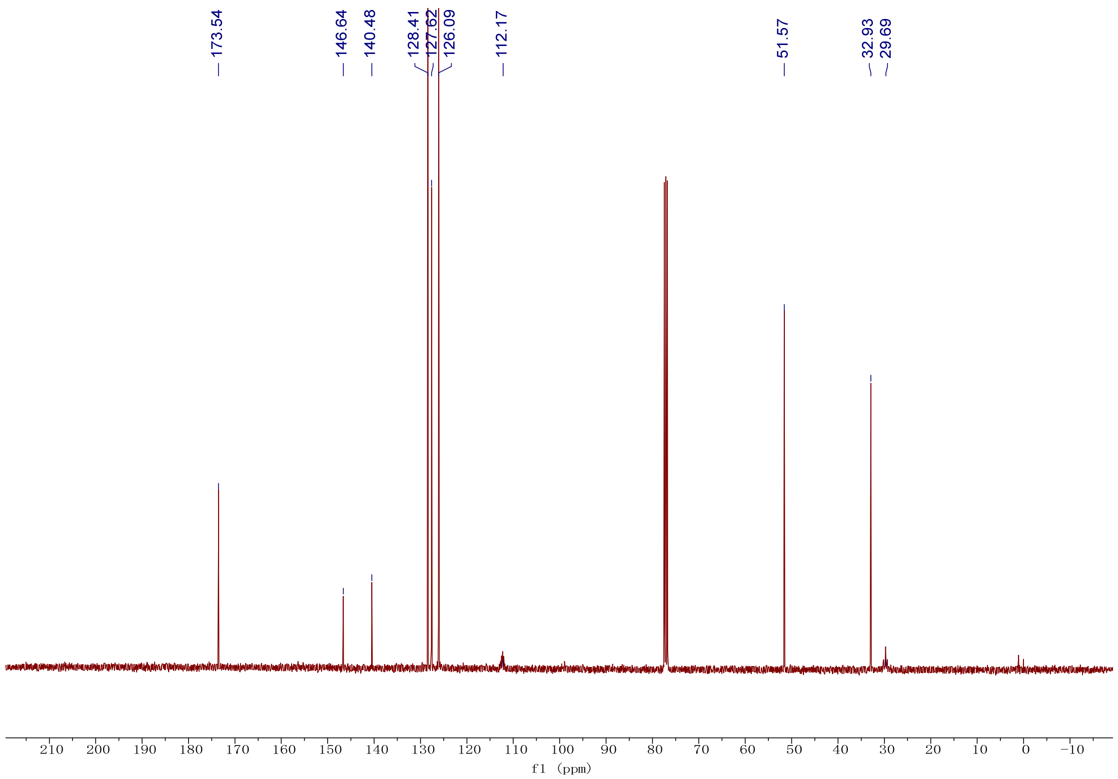

Supplementary Figure 16. ^{13}C NMR (101 MHz, $CDCl_3$) of **[D$_4$]-1a**

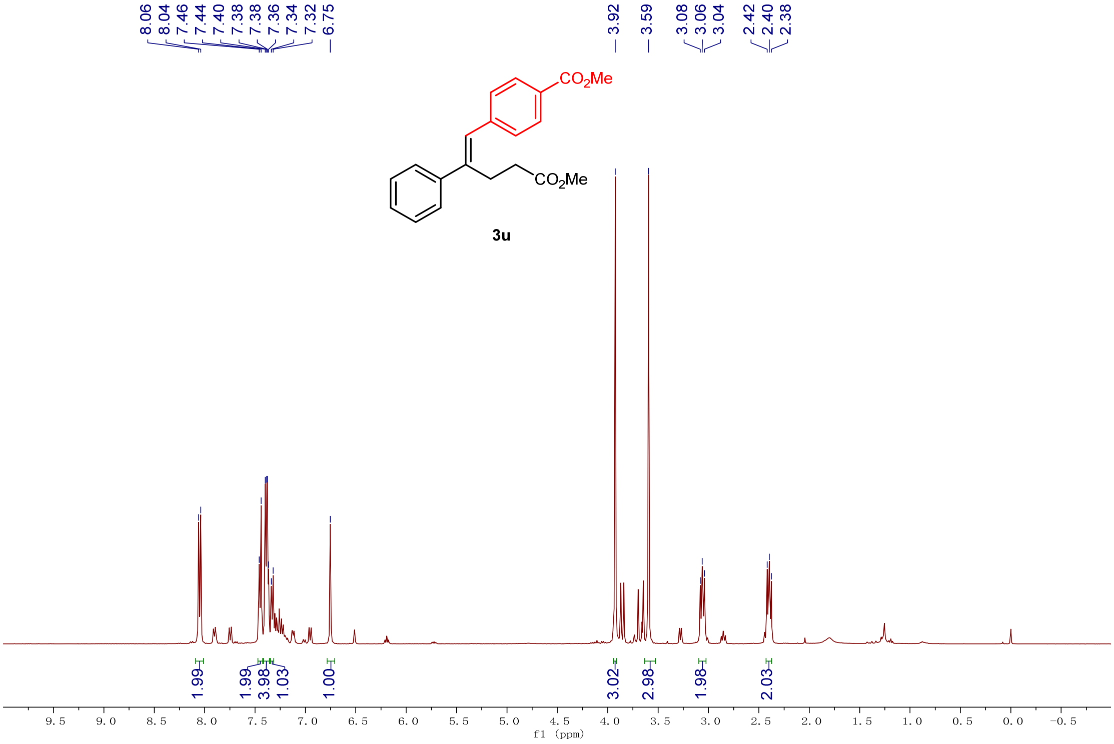

Supplementary Figure 17. ^{1}H NMR (400 MHz, $CDCl_3$) of **3u**, containing an inseparable byproduct

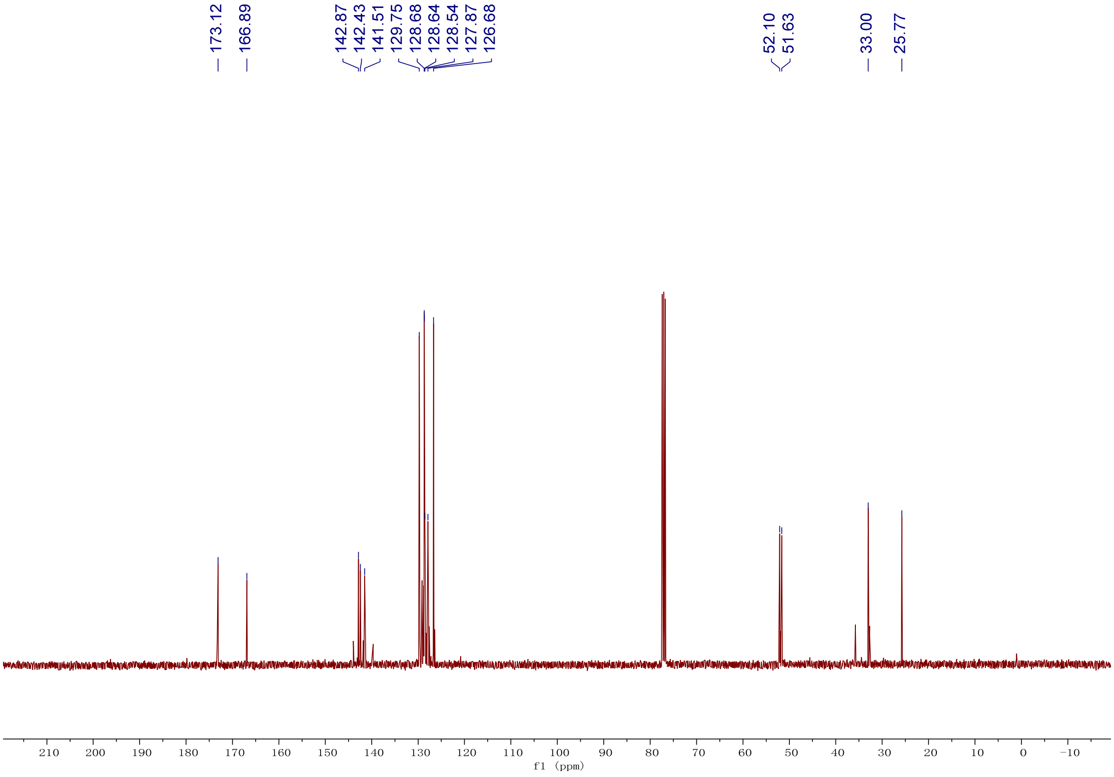

Supplementary Figure 18. ^{13}C NMR (101 MHz, $CDCl_3$) of **3u**, containing an inseparable byproduct

2.3. Synthetic Applications

2.3.1. Gram-Scale Experiments

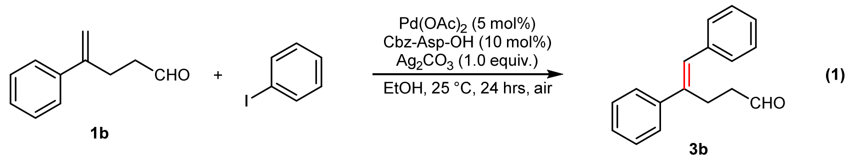

Gram-Scale Experiment (1): To an oven-dried 100 mL round bottom flask with previously placed magnetic stir-bar were added iodobenzene (1.05 mL, 9.36 mmol, 1.5 equiv.), $Pd(OAc)_2$ (70 mg, 0.312 mmol, 5 mol%), Cbz-Asp-OH (167 mg, 0.624 mmol, 10 mol%), Ag_2CO_3 (1.72 g, 6.24 mmol, 1.0 equiv.), followed by addition of EtOH (50 mL) and 4-phenylpent-4-enal **1b** (1 g, 6.24 mmol). The reaction mixture was stirred vigorously at room temperature (25 °C) for 24 hours. After completion of the reaction, the resultant solution was concentrated under reduced pressure, diluted with ethyl acetate (30 mL) and filtered through a short pad of 1:1 mixture of Celite and silica gel, the column was washed with ethyl acetate (50 mL). The combined organic solutions were concentrated under reduced pressure, and the residue was purified by flash column chromatography on silica gel (PE/EA = 9:1) to afford the desired product **3b** in 73% yield (1.07 g, light-yellow oil).

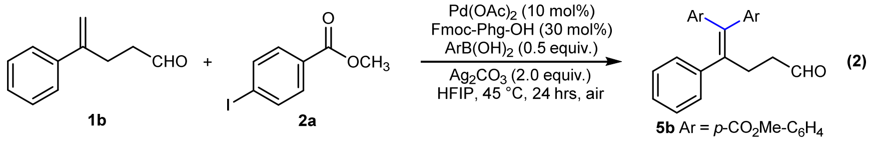

Gram-Scale Experiment (2): To an oven-dried 250 mL round bottom flask with previously placed magnetic stir-bar were added methyl 4-iodobenzoate **2a** (4.09 g, 15.6 mmol, 2.5 equiv.), 4-methoxybenzoborate (562 mg, 3.12 mmol, 0.5 equiv.), $Pd(OAc)_2$ (141 mg, 0.624 mmol, 10 mol%), Fmoc-Phg-OH (699 mg, 1.872 mmol, 30 mol%), Ag_2CO_3 (3.44 g, 12.48 mmol, 2.0 equiv.), followed by addition of HFIP (70 mL) and 4-phenylpent-4-enal **1b** (1 g, 6.24 mmol). The reaction mixture was stirred vigorously on a hotplate at 45 °C for 24 hours. After completion of the reaction, the resultant solution was concentrated under reduced pressure, diluted with ethyl acetate (50 mL) and filtered through a short pad of 1:1 mixture of Celite and silica gel, the column was washed with ethyl acetate (100 mL). The combined organic solutions were concentrated under reduced pressure, and the residue was purified by flash

column chromatography on silica gel (PE/EA = 4:1) to afford the desired product **5b** in 64% yield (1.71 g, light-yellow oil).

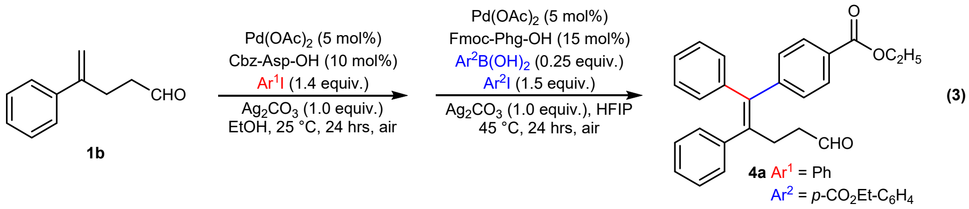

1b

Gram-Scale Experiment (3): To an oven-dried 100 mL round bottom flask with previously placed magnetic stir-bar were added iodobenzene (1.0 mL, 8.736 mmol, 1.4 equiv.), $Pd(OAc)_2$ (70 mg, 0.312 mmol, 5 mol%), Cbz-Asp-OH (167 mg, 0.624 mmol, 10 mol%), Ag_2CO_3 (1.72 g, 6.24 mmol, 1.0 equiv.), followed by addition of EtOH (50 mL) and 4-phenylpent-4-enal **1b** (1 g, 6.24 mmol). The reaction mixture was stirred vigorously at room temperature (25 °C) for 24 hours. After completion of the reaction, the resultant solution was concentrated under reduced pressure, diluted with ethyl acetate (30 mL) and filtered through a short pad of 1:1 mixture of Celite and silica gel, the column was washed with ethyl acetate (50 mL). The combined organic solutions were concentrated under reduced pressure to afford the mono-arylated product **3b** which was used in the next step without further purification. To another oven-dried 250 mL round bottom flask with previously placed magnetic stir-bar were added ethyl 4-iodobenzoate (2.58 g, 9.36 mmol, 1.5 equiv.), 4-ethoxycarbonylphenylboronic acid (303 mg, 1.56 mmol, 0.25 equiv.), $Pd(OAc)_2$ (70 mg, 0.312 mmol, 5 mol%), Fmoc-Phg-OH (350 mg, 0.936 mmol, 15 mol%), Ag_2CO_3 (1.72 g, 6.24 mmol, 1.0 equiv.), followed by the addition of the crude mono-arylated product **3b** dissolved in 70 mL HFIP. The reaction mixture was stirred vigorously on a hotplate at 45 °C for 24 hours. After completion of the reaction, the resultant solution was concentrated under reduced pressure, diluted with ethyl acetate (50 mL) and filtered through a short pad of 1:1 mixture of Celite and silica gel, the column was washed with ethyl acetate (100 mL). The combined organic solutions were concentrated under reduced pressure, and the residue was purified by flash column chromatography on silica gel (PE/EA = 5:1) to afford the desired double arylated product **4a** in 54% yield (1.29 g, light-yellow oil).

2.3.2. Photophysical Properties of 4s

The UV-Vis absorption spectra were measured on a Perkin-Elmer Lambda 35 UV-Vis spectrophotometer using a 10 mm path quartz cell. The emission and excitation spectra were measured on Edinburgh FLS-1000 fluorescence spectrometer. The emission spectra and quantum yields were measured upon excitation at 460 nm. All optical properties were measured at room temperature.

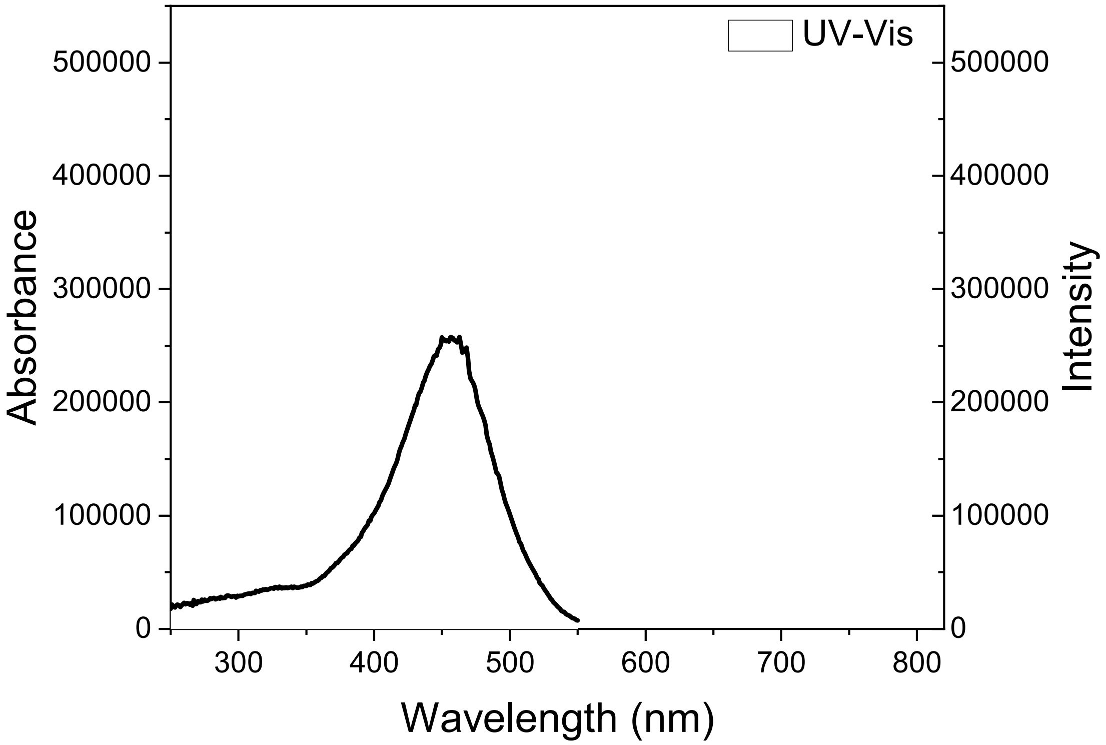

Supplementary Figure 19. Solid-state UV/Vis absorption spectrum of **4s**

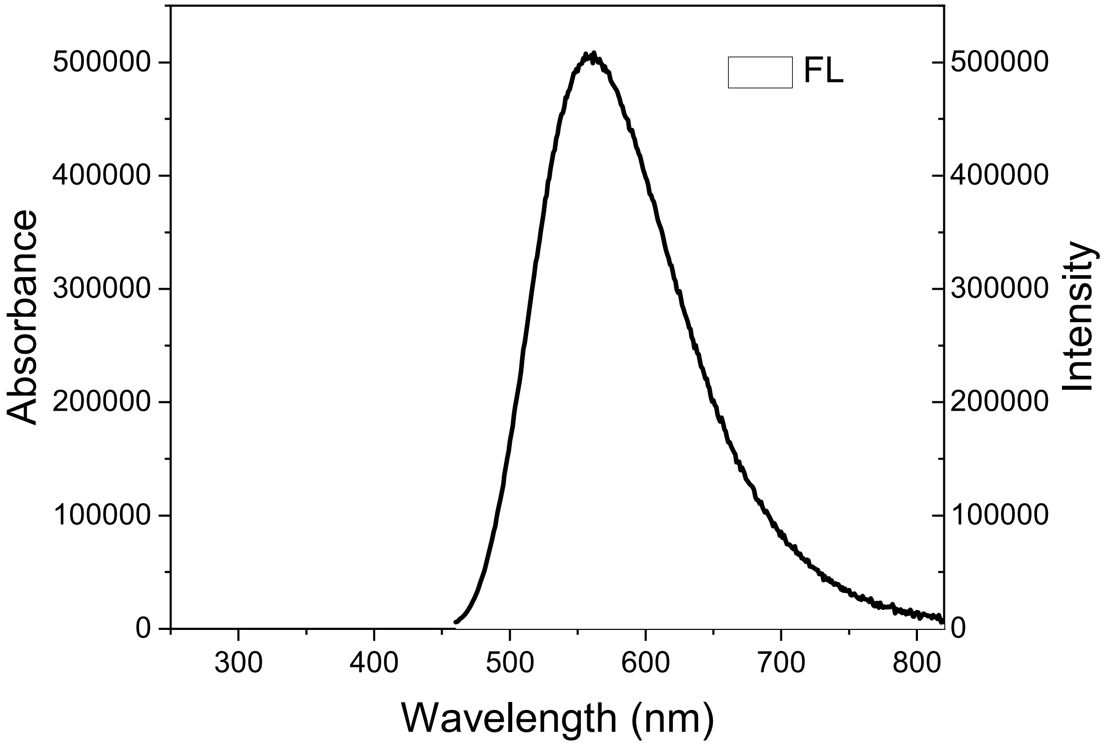

Supplementary Figure 20. Solid-state fluorescence emission spectrum of **4s**

3. Supplementary Tables and Figures

3.1. X-ray Data

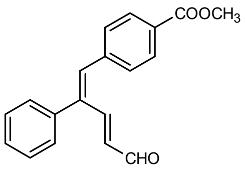

6a

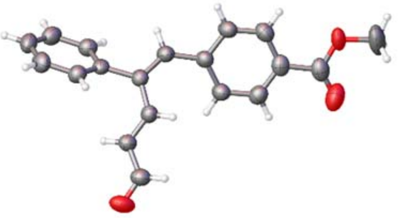

Supplementary Table 17. Crystal data and structure refinement for **6a**

Identification code	2252784	
Empirical formula	$C_{19}H_{16}O_3$	
Formula weight	164.76	
Temperature (K)	293(2)	
Wavelength (Å)	1.54184	
Crystal system	monoclinic	
Space group	$P2_1/c$	
Unit cell dimensions (Å, °)	a = 15.0734(5)	a = 90
	b = 13.1381(4)	b = 90.519(4)
	c = 7.8048(3)	g = 90
Volume (Å)	1545.57(9)	
Z	7	
Calculated density (g cm^{-3})	1.239	
Absorption coefficient (mm^{-1})	0.625	
F_{000}	608	
Crystal size (mm^3)	0.1 x 0.04 x 0.03	
q range for data collection (°)	2.932 to 75.899	
Miller index ranges	$-18 \leq h \leq 17$, $-16 \leq k \leq 7$, $-9 \leq l \leq 9$	
Reflections collected	5393	
Independent reflections	2585 [R_{int} = 0.0283]	
Completeness to q_{max} (%)	0.804	
Max. and min. transmission	0.90877 and 1.00000	
Refinement method	Full-matrix least-squares on F^2	
Data / restraints / parameters	2585 / 1 / 200	
Goodness-of-fit on F^2	1.116	

Final *R* indices [*I* > 2s(*I*)]	*R*1 = 0.0933, *wR*2 = 0.2727
R indices (all data)	*R*1 = 0.1031, *wR*2 = 0.2796
Largest diff. peak and hole (e Å^{-3})	0.575 and -0.321

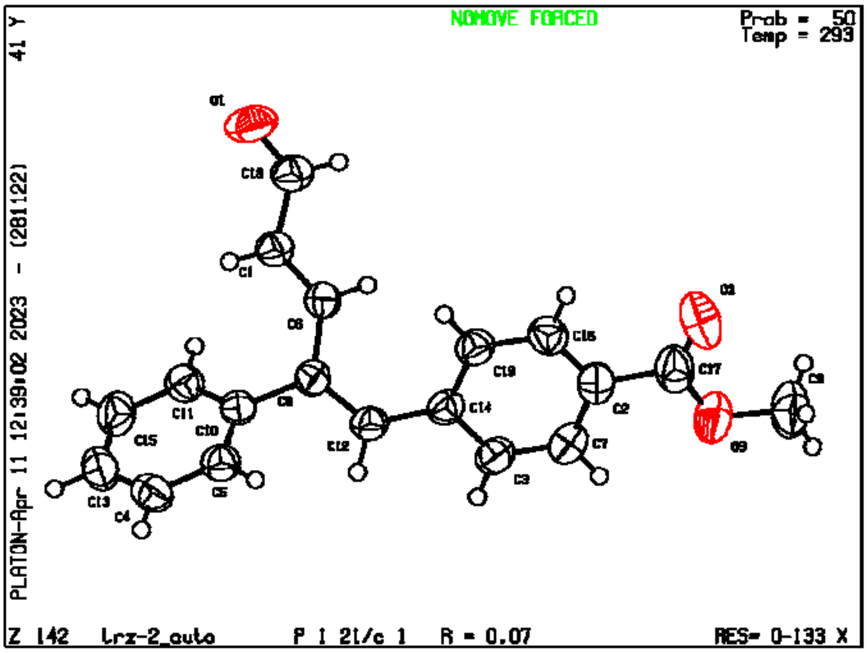

Supplementary Figure 21. Crystal structure diagram of **6a**

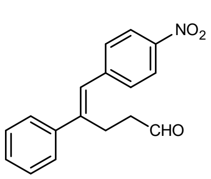

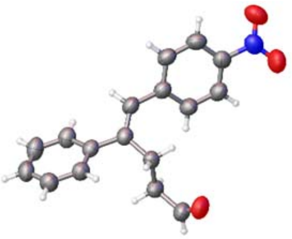

3f

Supplementary Table 18. Crystal data and structure refinement for **3f**

Identification code	2252796	
Empirical formula	$C_{17}H_{15}NO_3$	
Formula weight	186.20	
Temperature (K)	293(2)	
Wavelength (Å)	1.54184	
Crystal system	orthorhombic	
Space group	*P*$2_12_12_1$	
Unit cell dimensions (Å, °)	*a* = 8.6155(3)	a = 90
	b = 9.4679(3)	b = 90
	c = 17.6023(4)	g = 90

Volume (Å)	1435.83(8)
Z	6
Calculated density (g cm^{-3})	1.292
Absorption coefficient (mm^{-1})	0.708
F_{000}	588
Crystal size (mm^3)	0.1 x 0.08 x 0.07
q range for data collection (°)	5.025 to 75.583
Miller index ranges	$-10 \le h \le 10$, $-11 \le k \le 11$, $-11 \le l \le 22$
Reflections collected	5004
Independent reflections	2583 [R_{int} = 0.0297]
Completeness to q_{max} (%)	0.979
Max. and min. transmission	0.90841 and 1.00000
Refinement method	Full-matrix least-squares on F^2
Data / restraints / parameters	2583 / 0 / 190
Goodness-of-fit on F^2	1.045
Final R indices [$I > 2s(I)$]	$R1$ = 0.0463, $wR2$ = 0.1317
R indices (all data)	$R1$ = 0.0526, $wR2$ = 0.1390
Largest diff. peak and hole (e Å^{-3})	0.225 and -0.185
Absolute structure parameter	0.1(2)

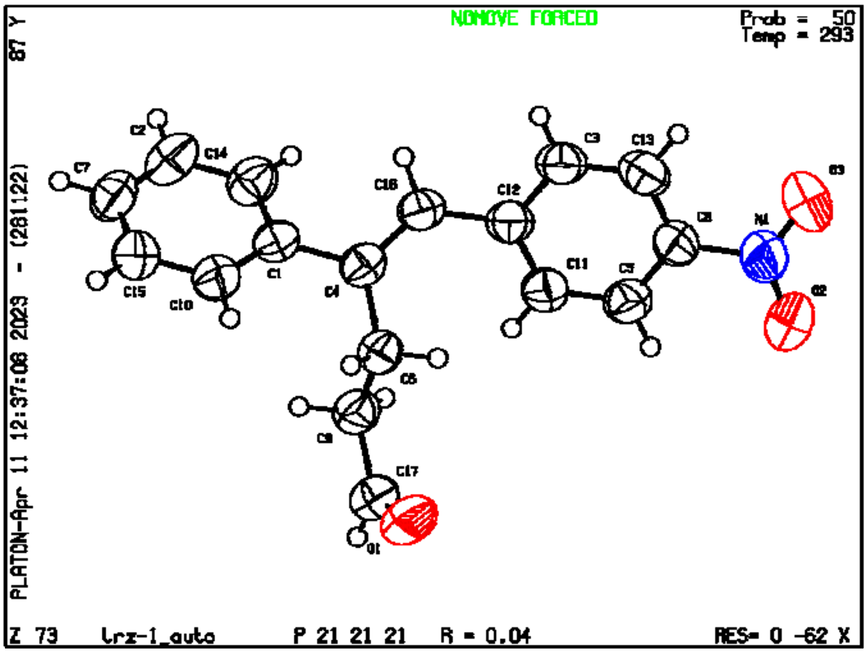

Supplementary Figure 22. Crystal structure diagram of **3f**

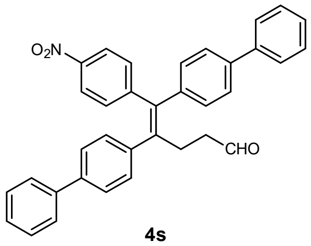

4s

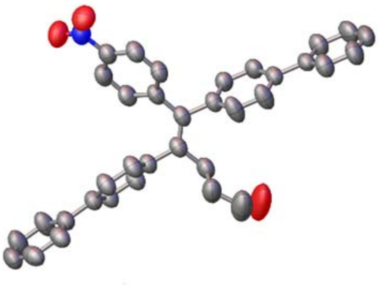

Supplementary Table 19. Crystal data and structure refinement for **4s**.

Identification code	2253116	
Empirical formula	$C_{35}H_{27}NO_3{\cdot}O$	
Formula weight	525.57	
Temperature (K)	293(2)	
Wavelength (Å)	1.54184	
Crystal system	monoclinic	
Space group	*C*2/*c*	
Unit cell dimensions (Å, °)	*a* = 27.0694(7)	a = 90
	b = 8.7808(3)	b = 90.923(2)
	c = 25.0765(6)	g = 90
Volume (Å)	5959.7(3)	
Z	8	
Calculated density (g cm^{-3})	1.172	
Absorption coefficient (mm^{-1})	0.611	
F_{000}	2208	
Crystal size (mm^3)	0.1x0.08 x0.08	
q range for data collection (°)	3.266 to 75.637	
Miller index ranges	$-33 \leq h \leq 32$, $-10 \leq k \leq 4$, $-29 \leq l \leq 31$	
Reflections collected	18793	
Independent reflections	5936 [R_{int} = 0.0245]	
Completeness to q_{max} (%)	0.960	
Max. and min. transmission	0.64021 and 1.00000	
Refinement method	Full-matrix least-squares on F^2	
Data / restraints / parameters	5936 / 54 / 388	
Goodness-of-fit on F^2	1.185	
Final *R* indices [$I > 2s(I)$]	*R*1 = 0.0976, *wR*2 = 0.2879	

R indices (all data)	*R*1 = 0.1244, *wR*2 = 0.3214
Largest diff. peak and hole (e Å^{-3})	0.806 and -0.224

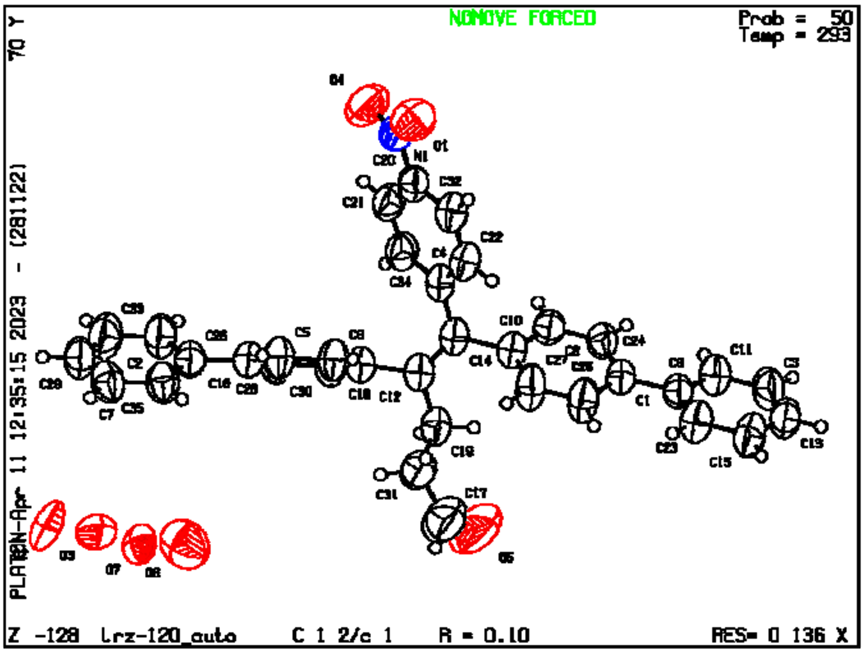

Supplementary Figure 23. Crystal structure diagram of **4s**

3.2. NMR Spectra

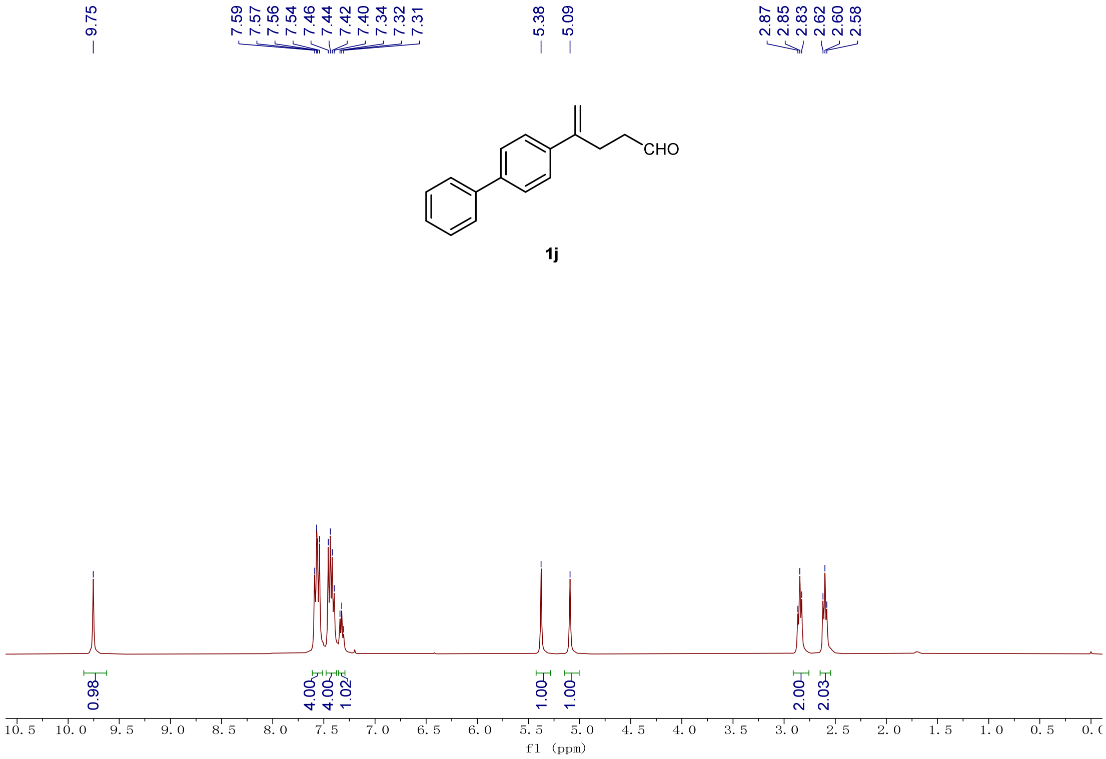

Supplementary Figure 24. ^{1}H NMR (400 MHz, $CDCl_3$) of **1j**

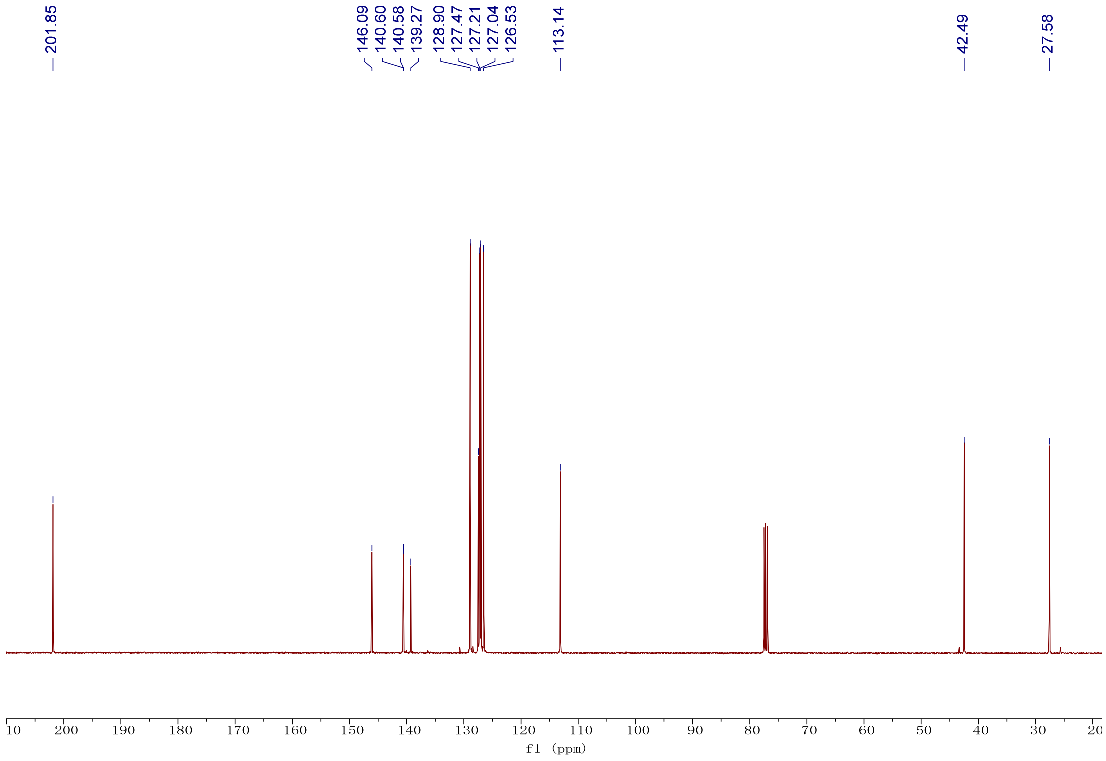

Supplementary Figure 25. ^{13}C NMR (101 MHz, $CDCl_3$) of **1j**

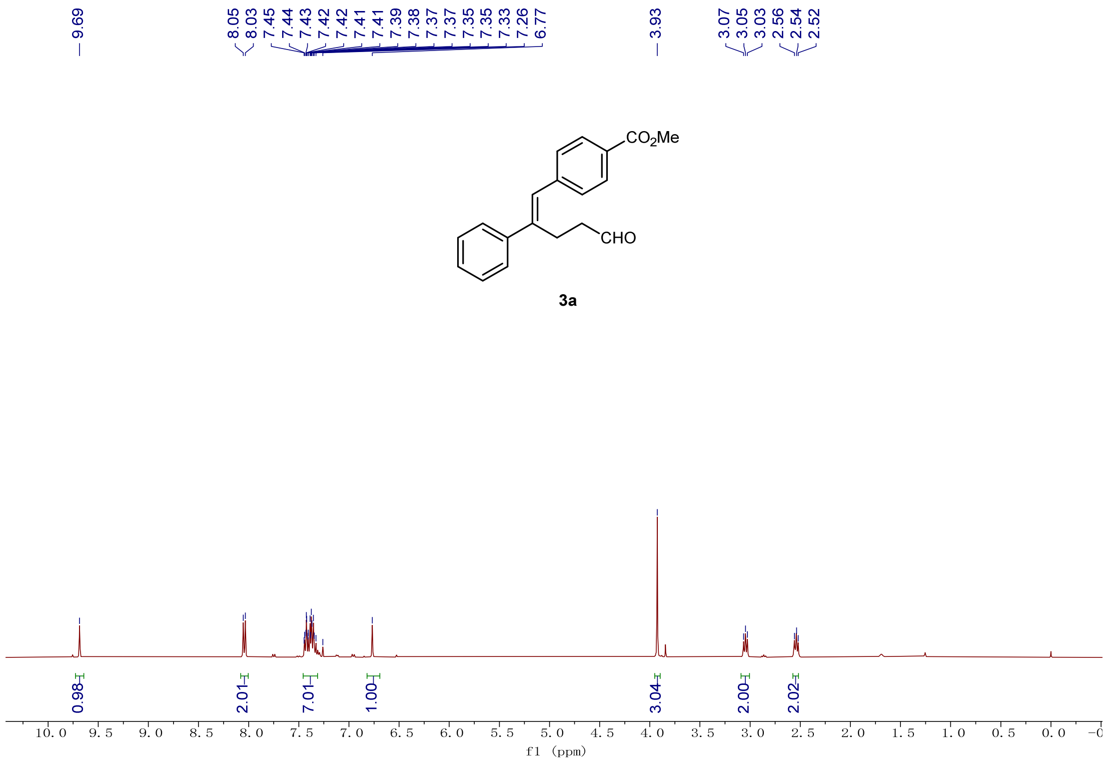

Supplementary Figure 26. ^{1}H NMR (400 MHz, $CDCl_3$) of **3a**

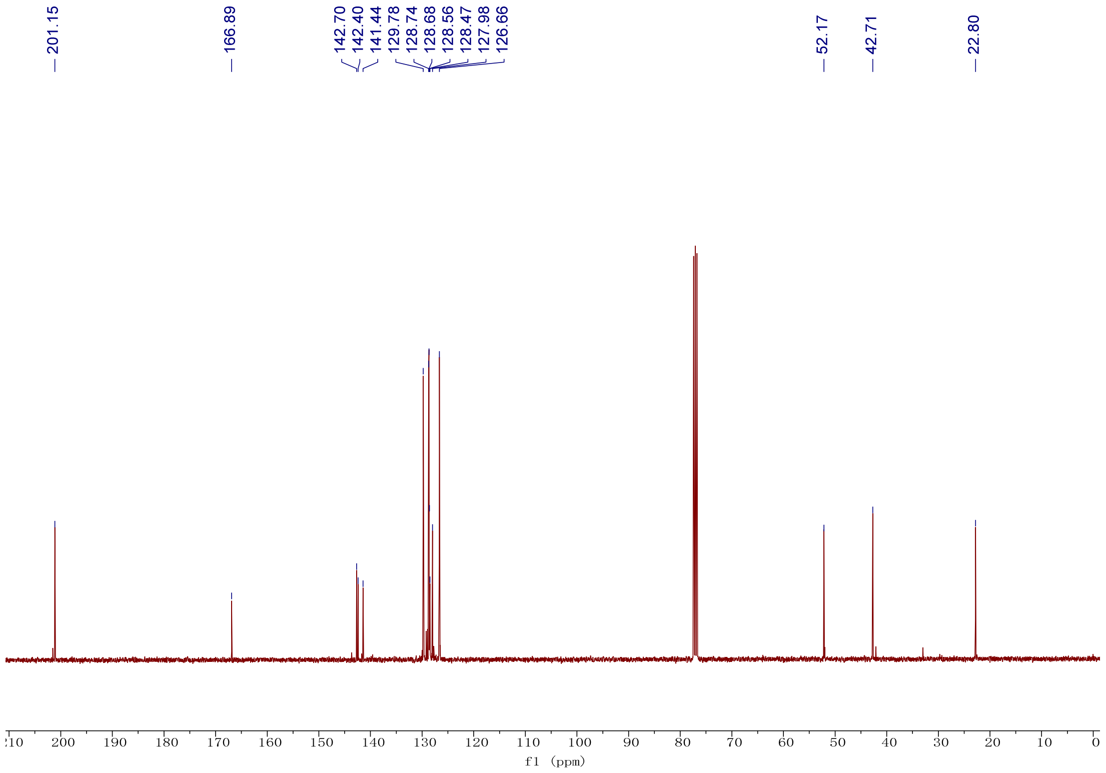

Supplementary Figure 27. ^{13}C NMR (101 MHz, $CDCl_3$) of **3a**

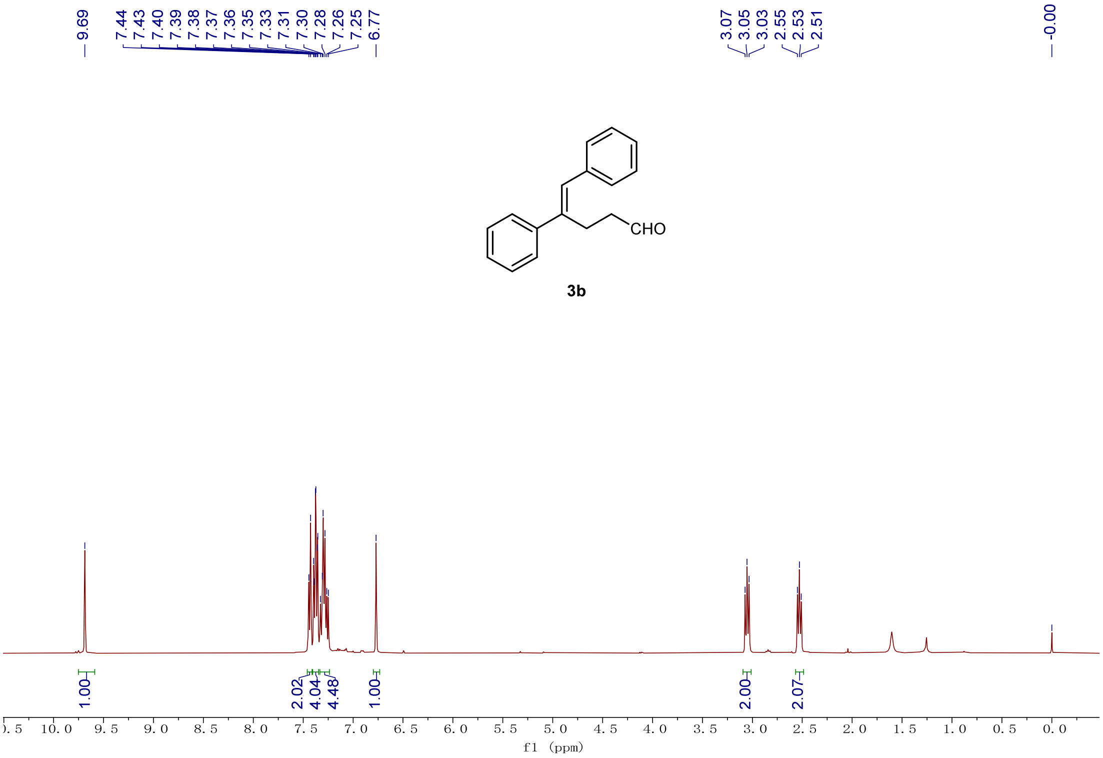

Supplementary Figure 28. ^{1}H NMR (400 MHz, $CDCl_3$) of **3b**

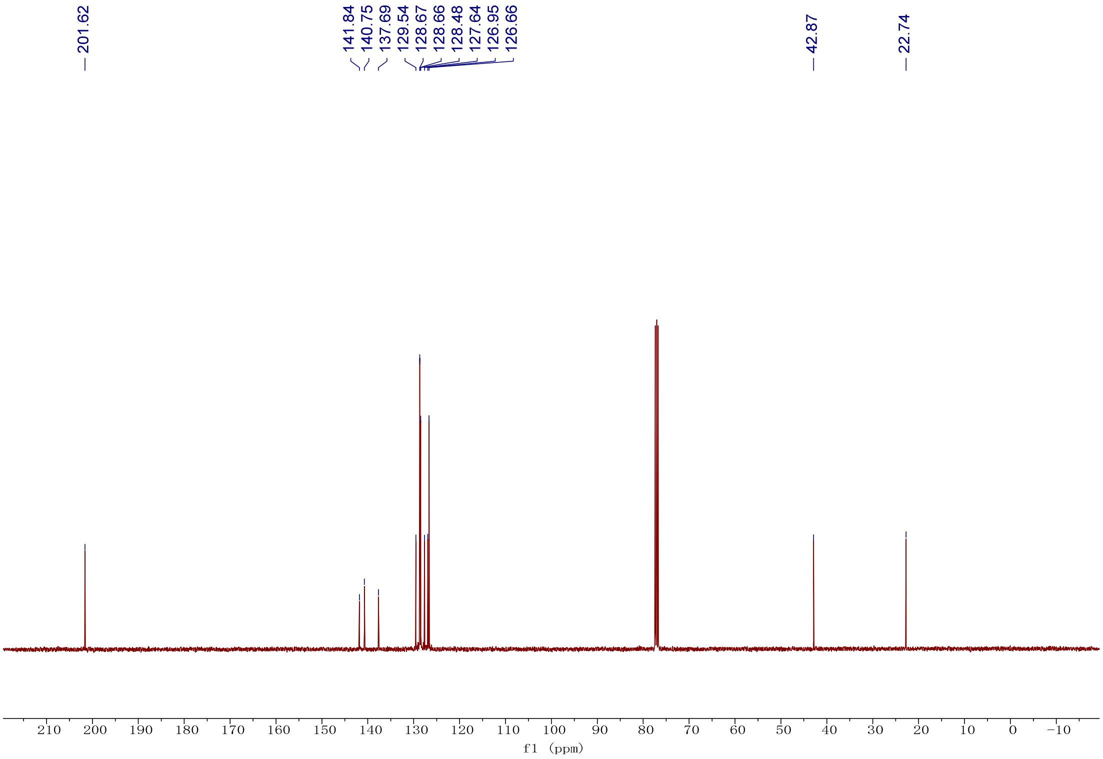

Supplementary Figure 29. ^{13}C NMR (101 MHz, $CDCl_3$) of **3b**

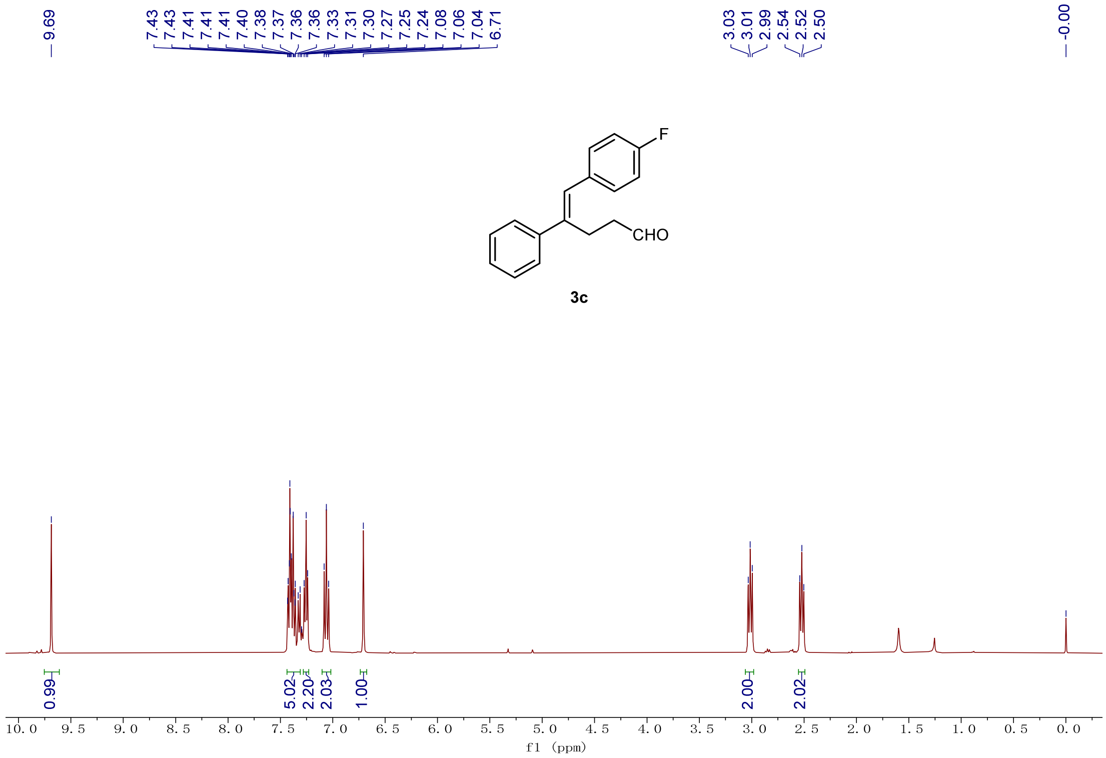

Supplementary Figure 30. ^{1}H NMR (400 MHz, $CDCl_3$) of **3c**

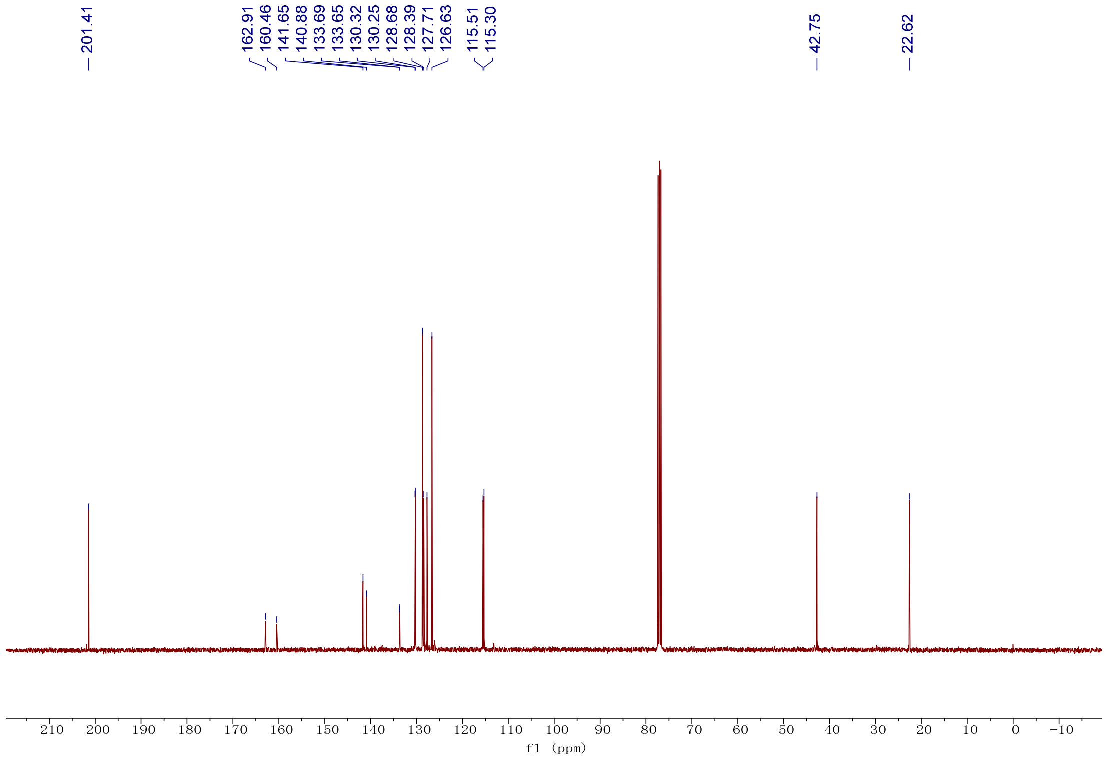

Supplementary Figure 31. ^{13}C NMR (101 MHz, $CDCl_3$) of **3c**

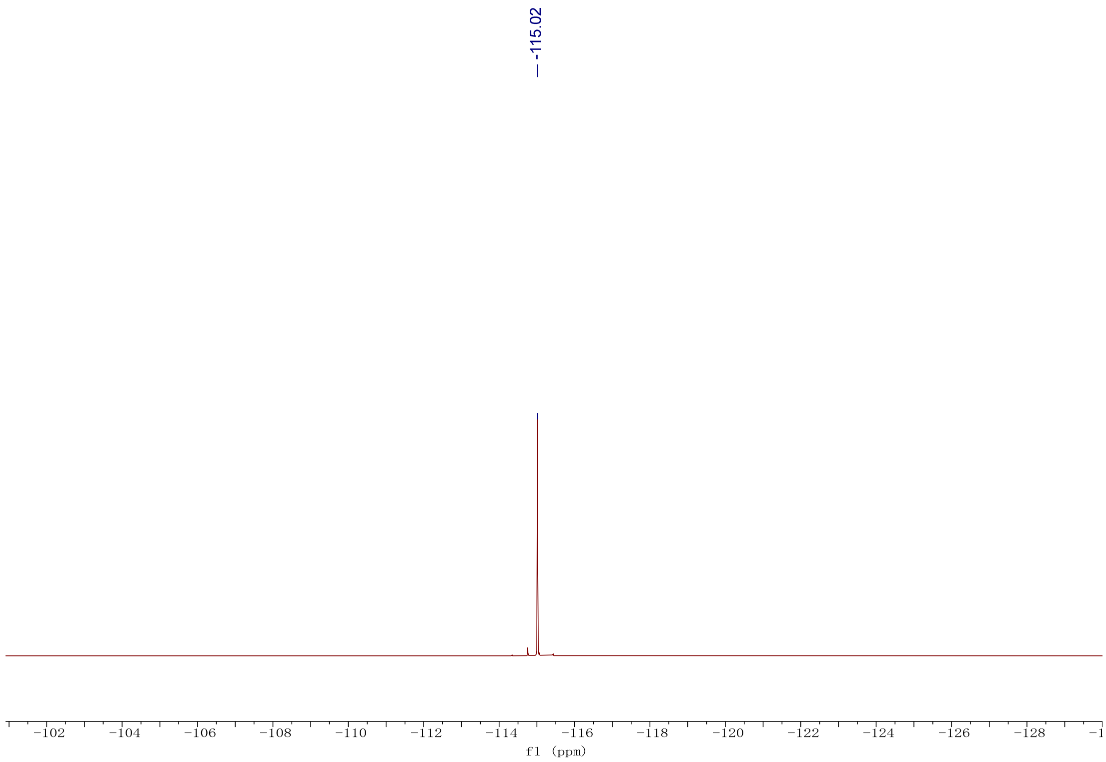

Supplementary Figure 32. ^{19}F NMR (376 MHz, $CDCl_3$) of **3c**

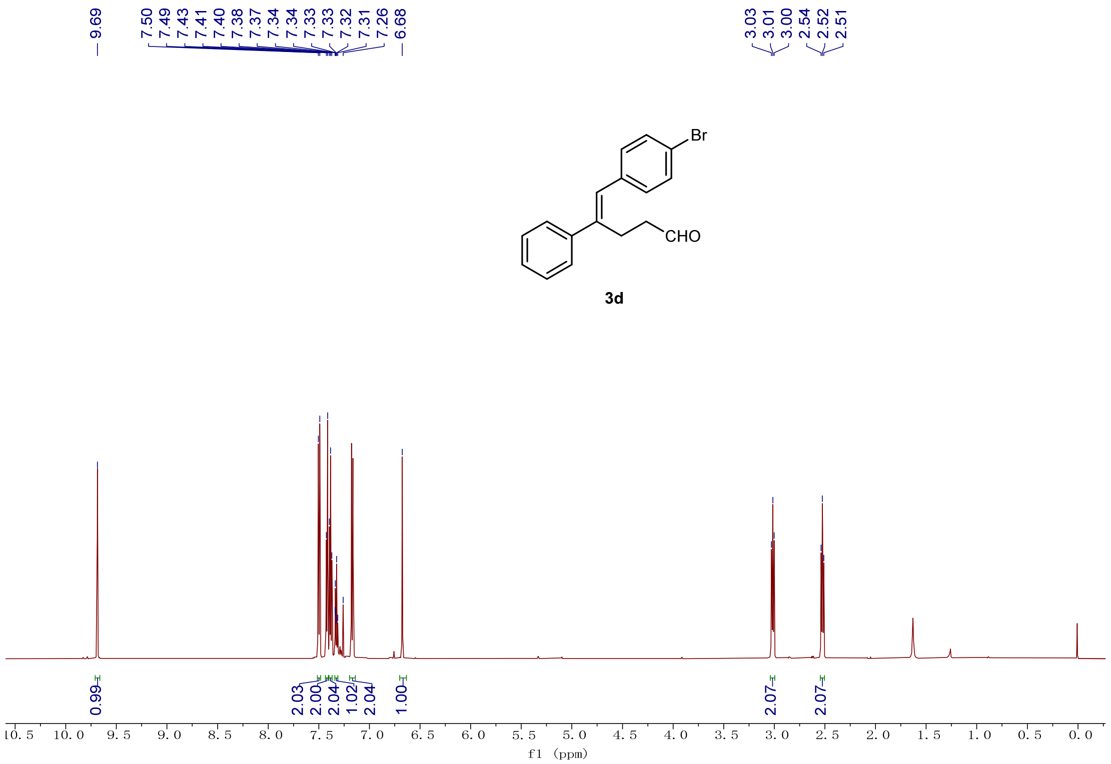

Supplementary Figure 33. ^{1}H NMR (600 MHz, $CDCl_3$) of **3d**

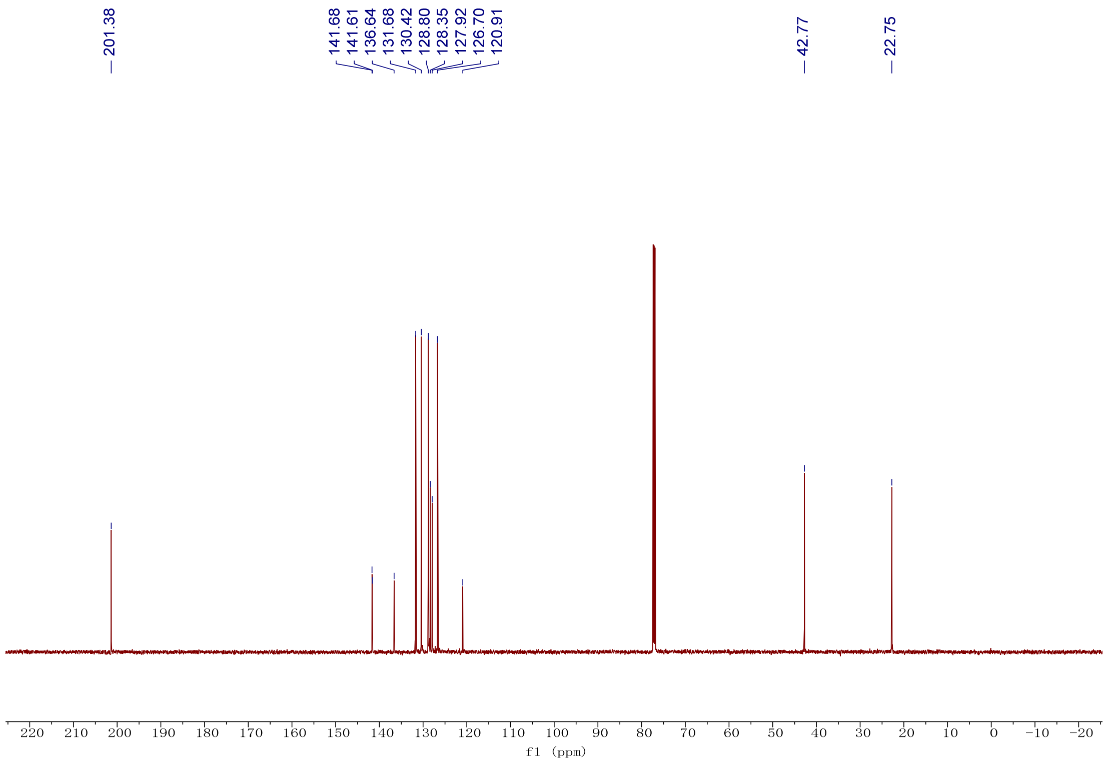

Supplementary Figure 34. ^{13}C NMR (151 MHz, $CDCl_3$) of **3d**

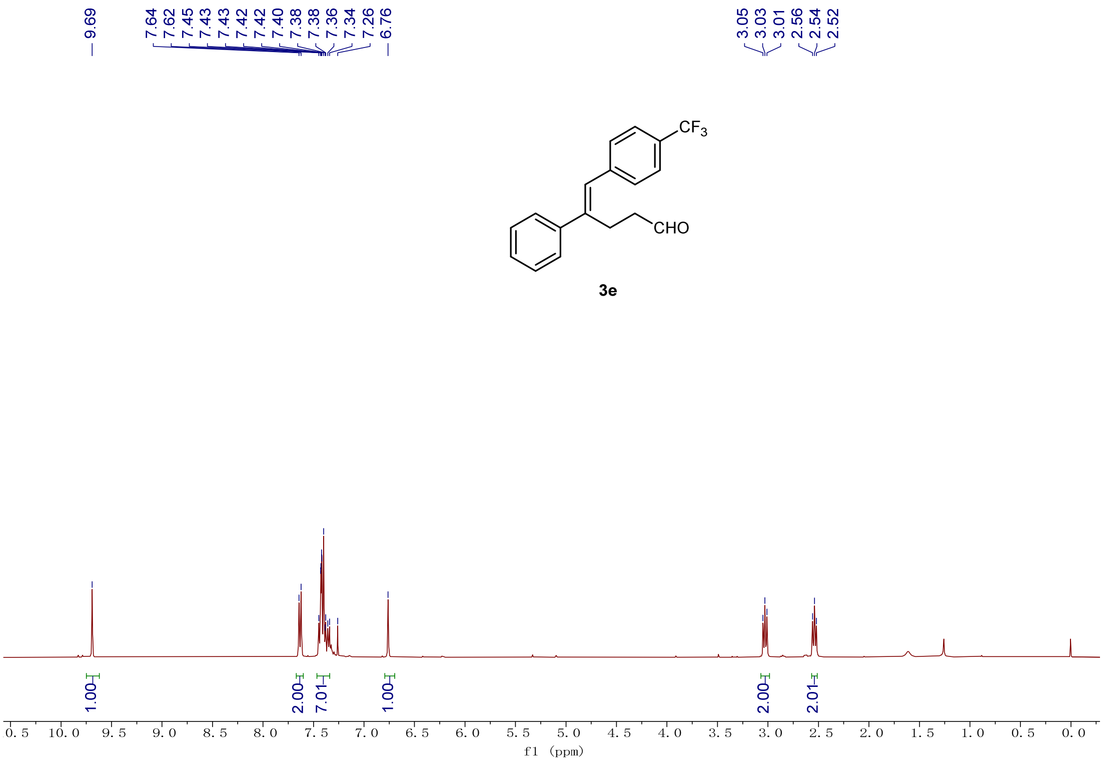

Supplementary Figure 35. ^{1}H NMR (400 MHz, $CDCl_3$) of **3e**

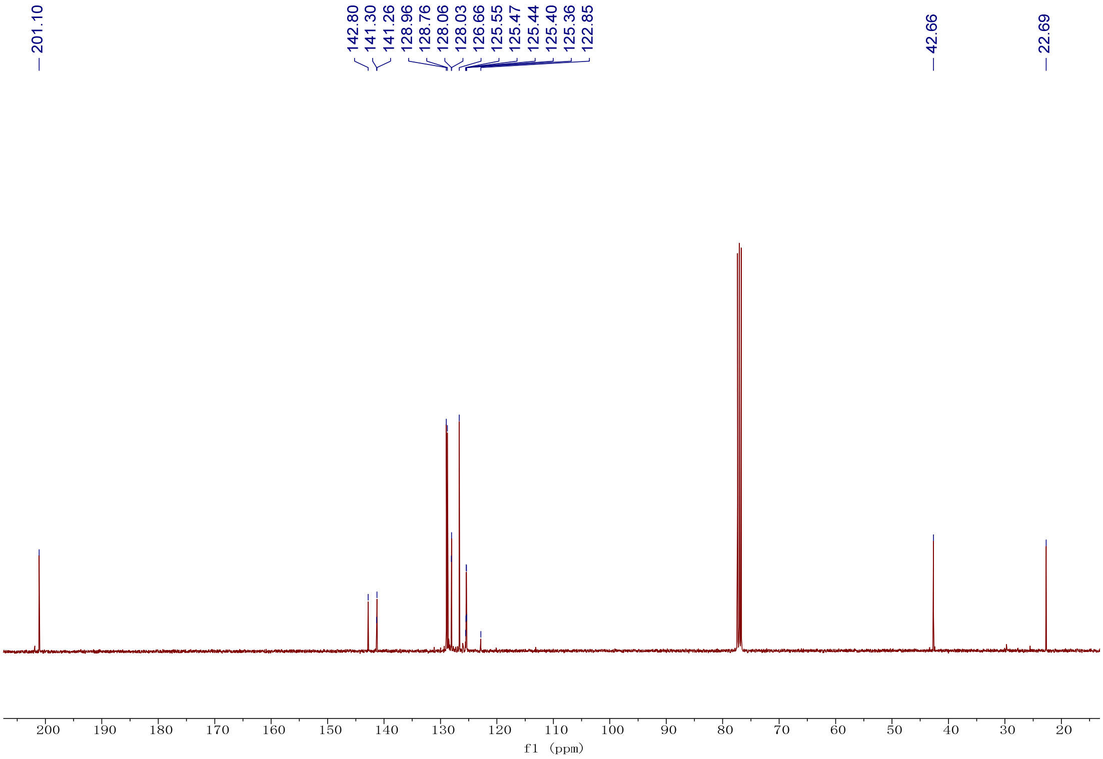

Supplementary Figure 36. ^{13}C NMR (101 MHz, $CDCl_3$) of **3e**

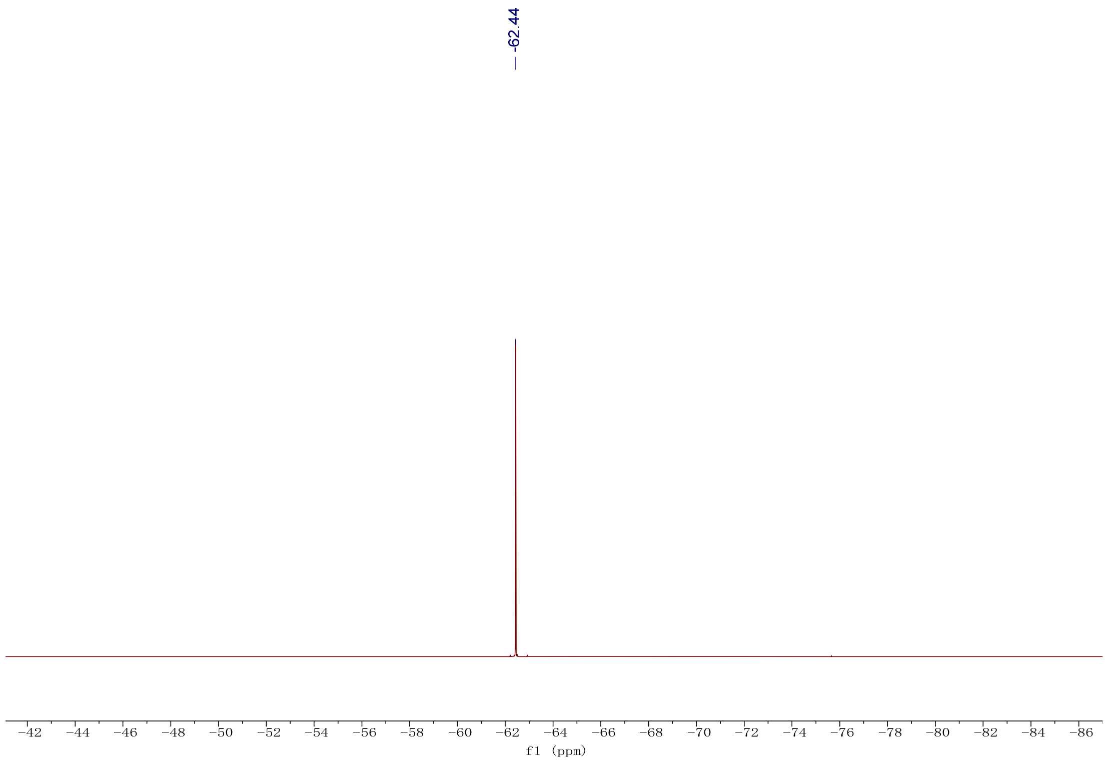

Supplementary Figure 37. ^{19}F NMR (376 MHz, $CDCl_3$) of **3e**

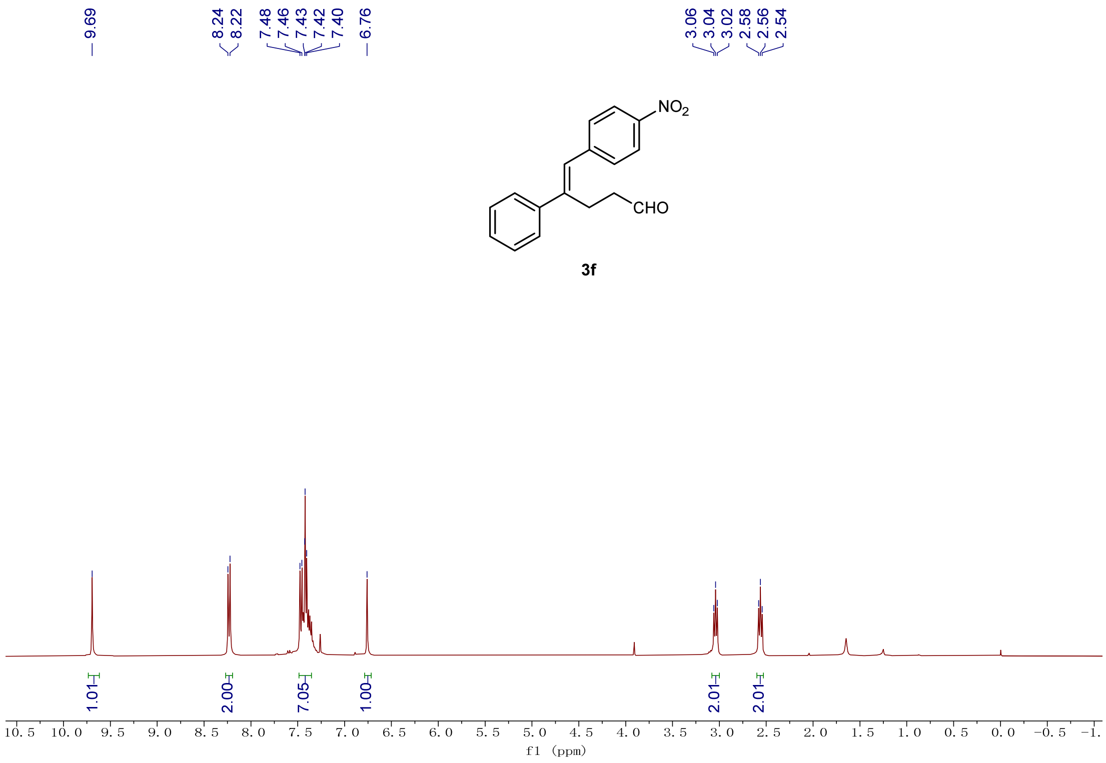

Supplementary Figure 38. ^{1}H NMR (400 MHz, $CDCl_3$) of **3f**

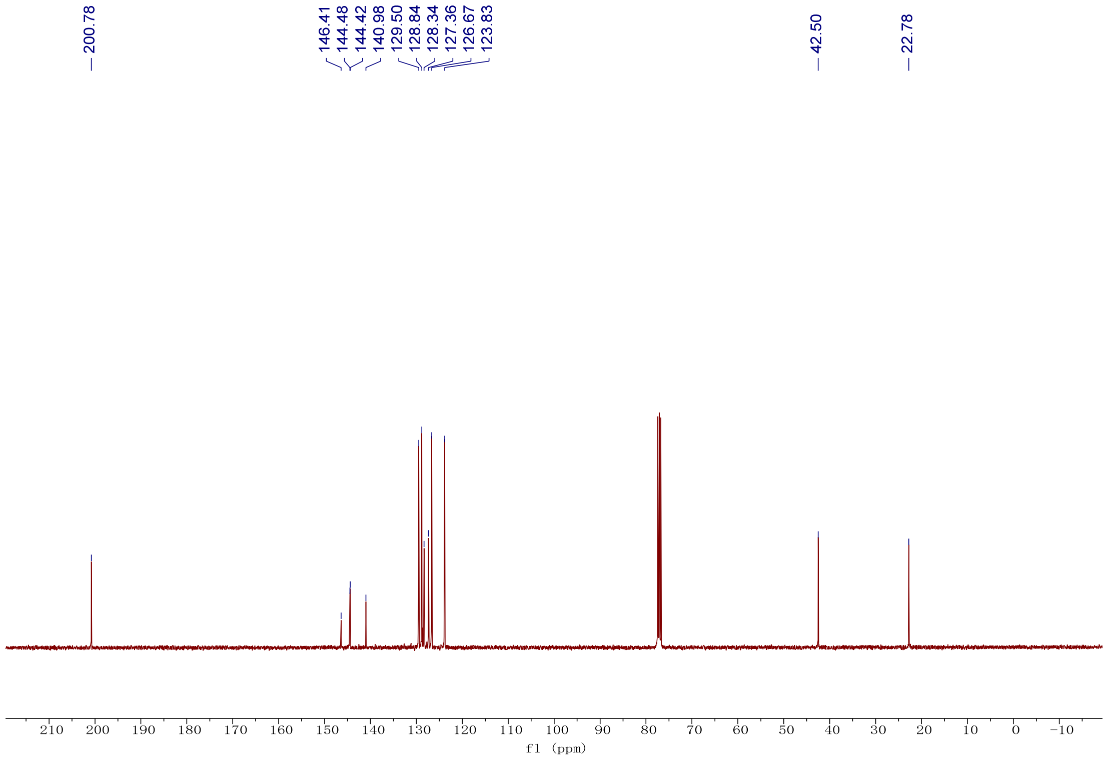

Supplementary Figure 39. ^{13}C NMR (101 MHz, $CDCl_3$) of **3f**

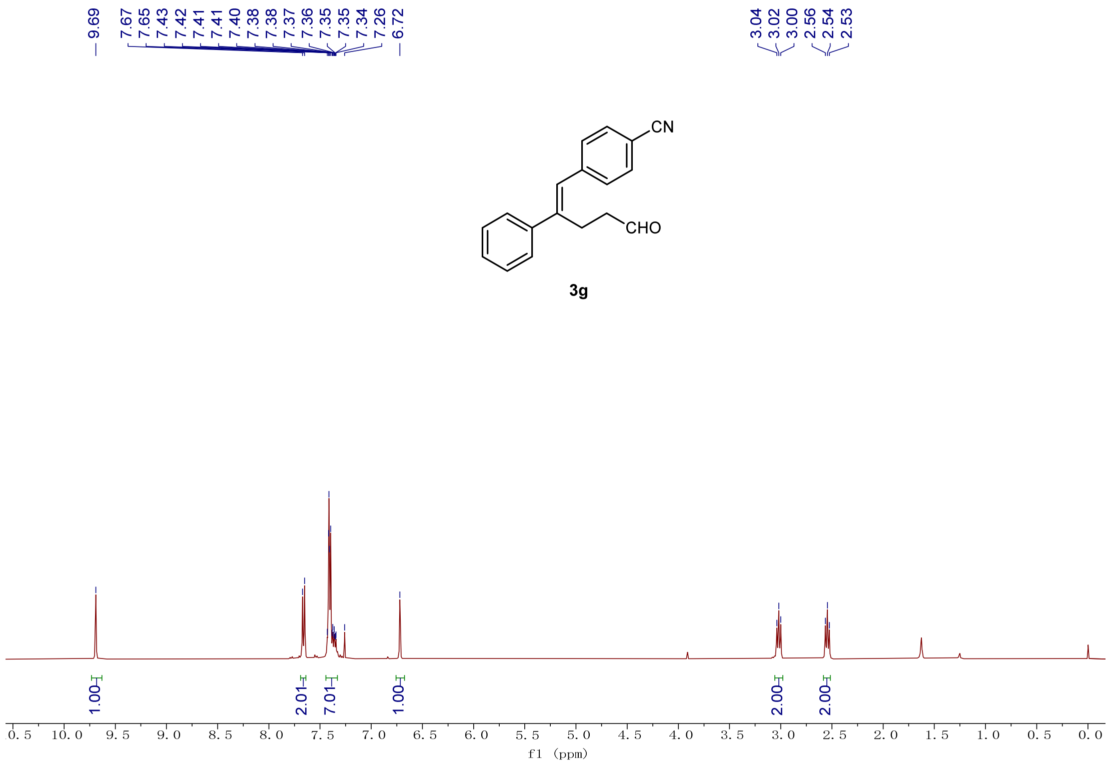

Supplementary Figure 40. ^{1}H NMR (400 MHz, $CDCl_3$) of **3g**

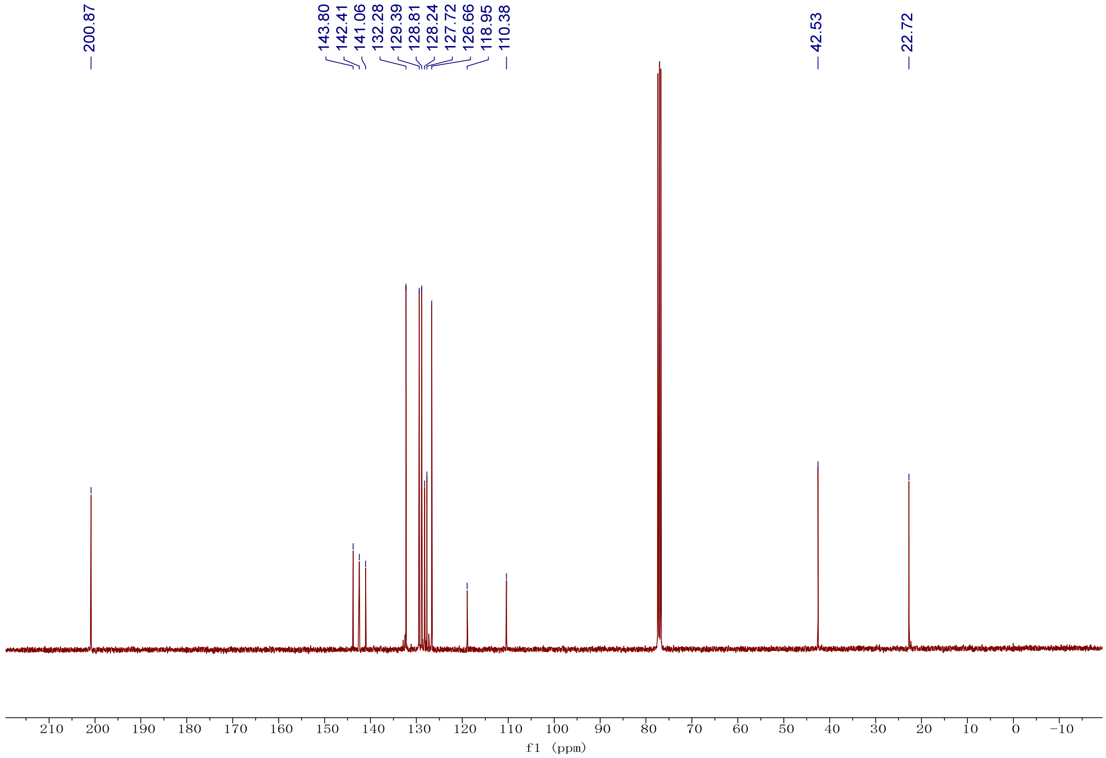

Supplementary Figure 41. ^{13}C NMR (101 MHz, $CDCl_3$) of **3g**

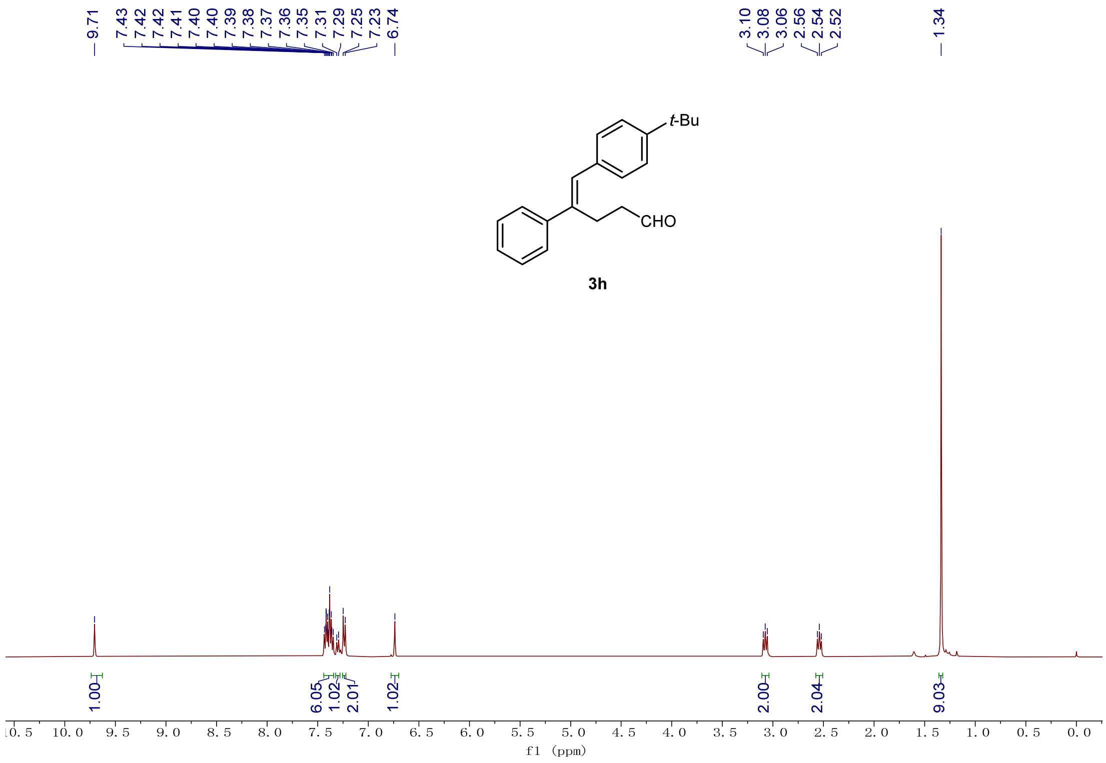

Supplementary Figure 42. ^{1}H NMR (400 MHz, $CDCl_3$) of **3h**

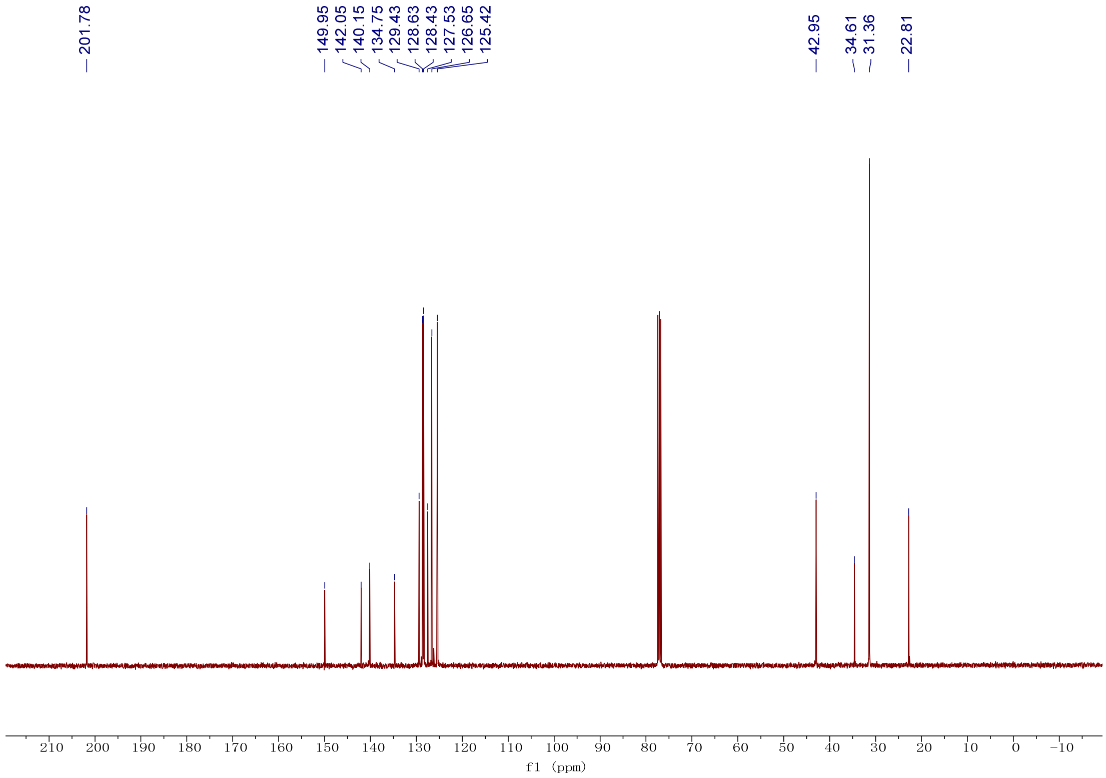

Supplementary Figure 43. ^{13}C NMR (101 MHz, $CDCl_3$) of **3h**

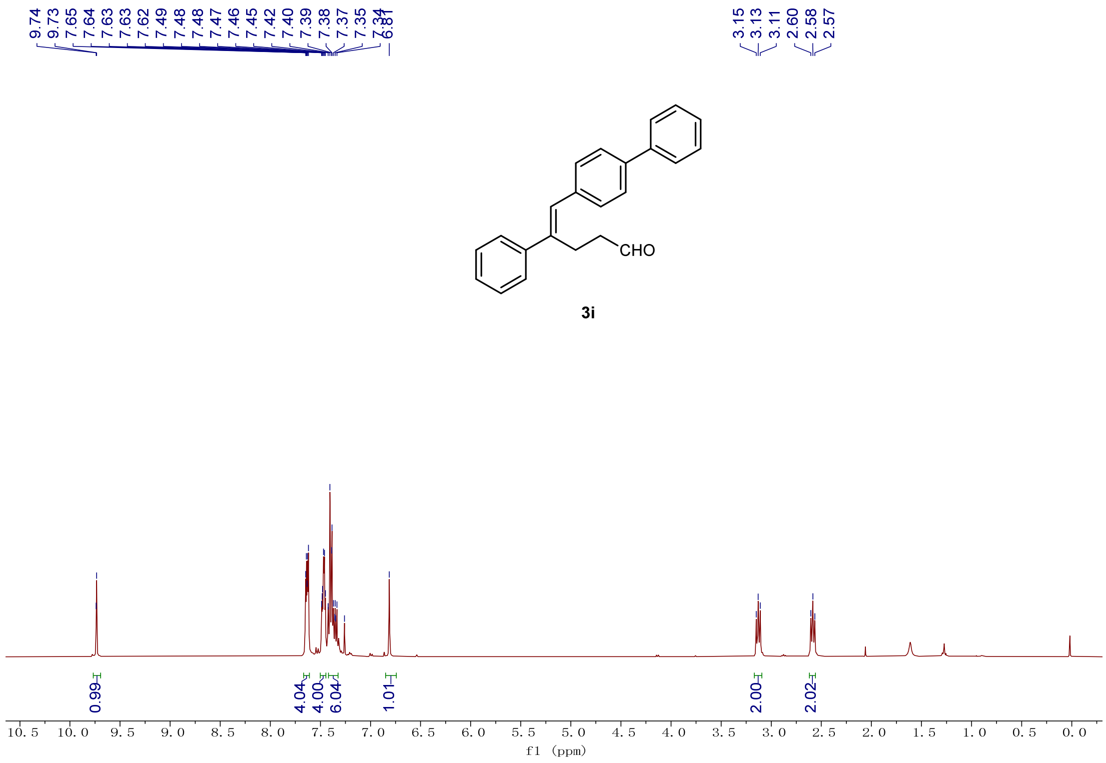

Supplementary Figure 44. ^{1}H NMR (400 MHz, $CDCl_3$) of **3i**

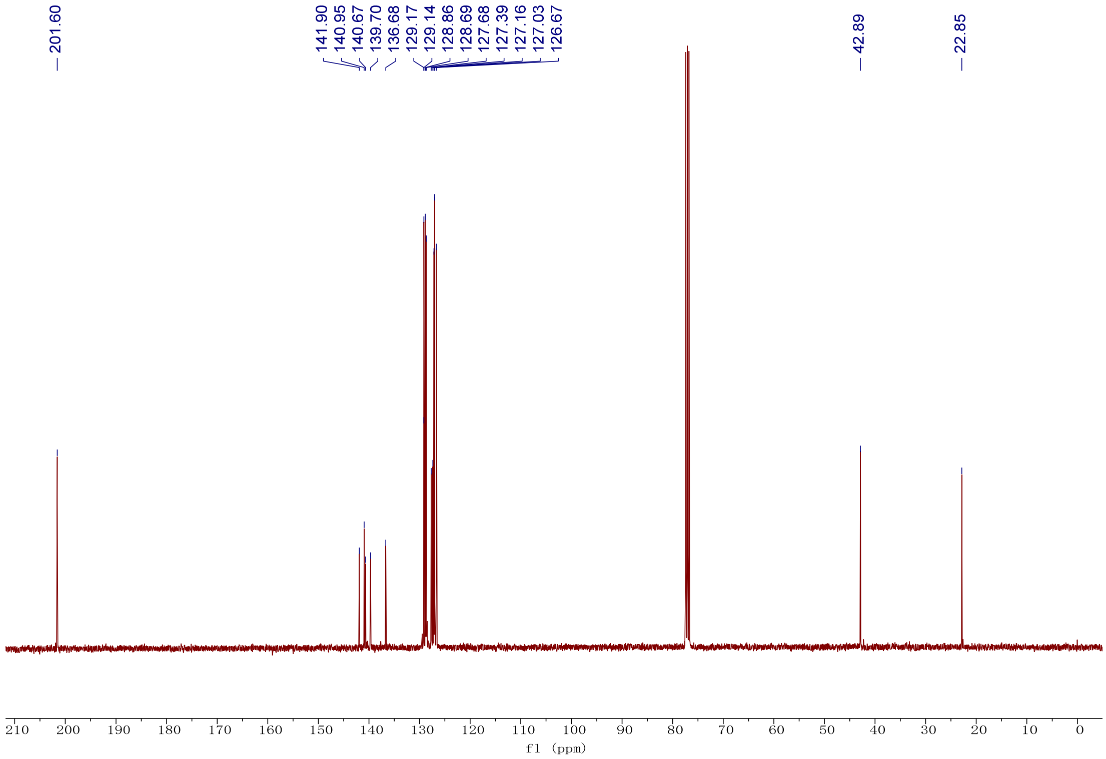

Supplementary Figure 45. ^{13}C NMR (101 MHz, $CDCl_3$) of **3i**

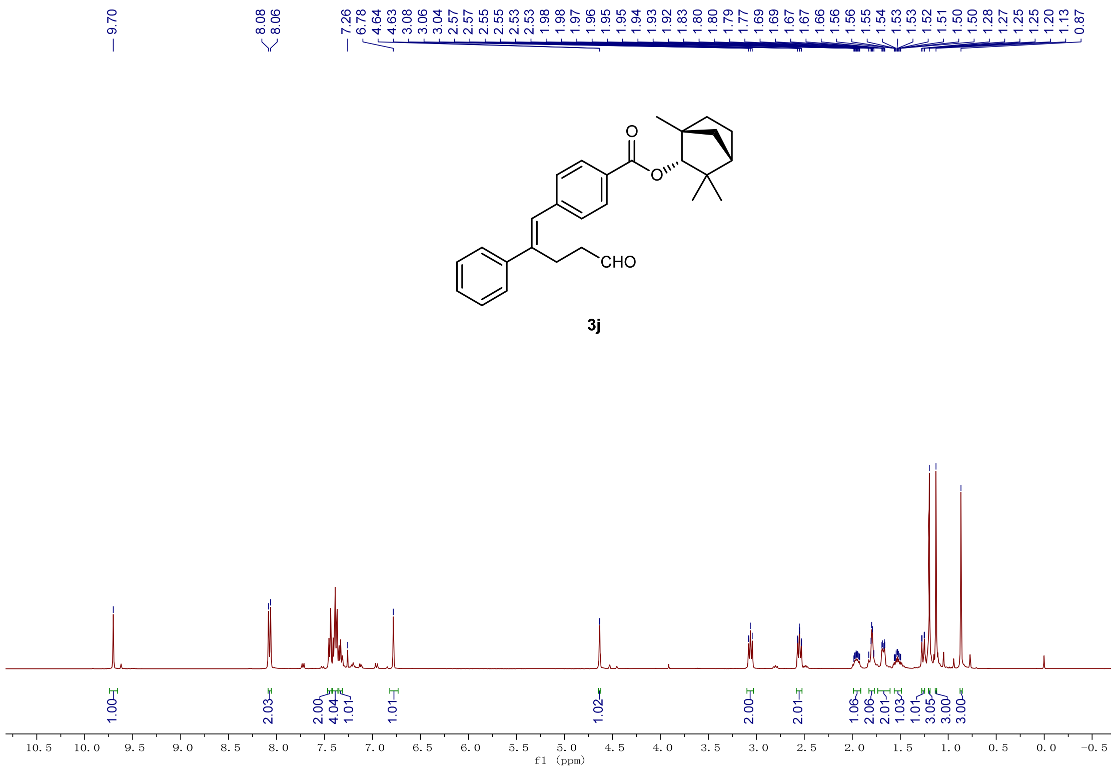

Supplementary Figure 46. ^{1}H NMR (400 MHz, $CDCl_3$) of **3j**

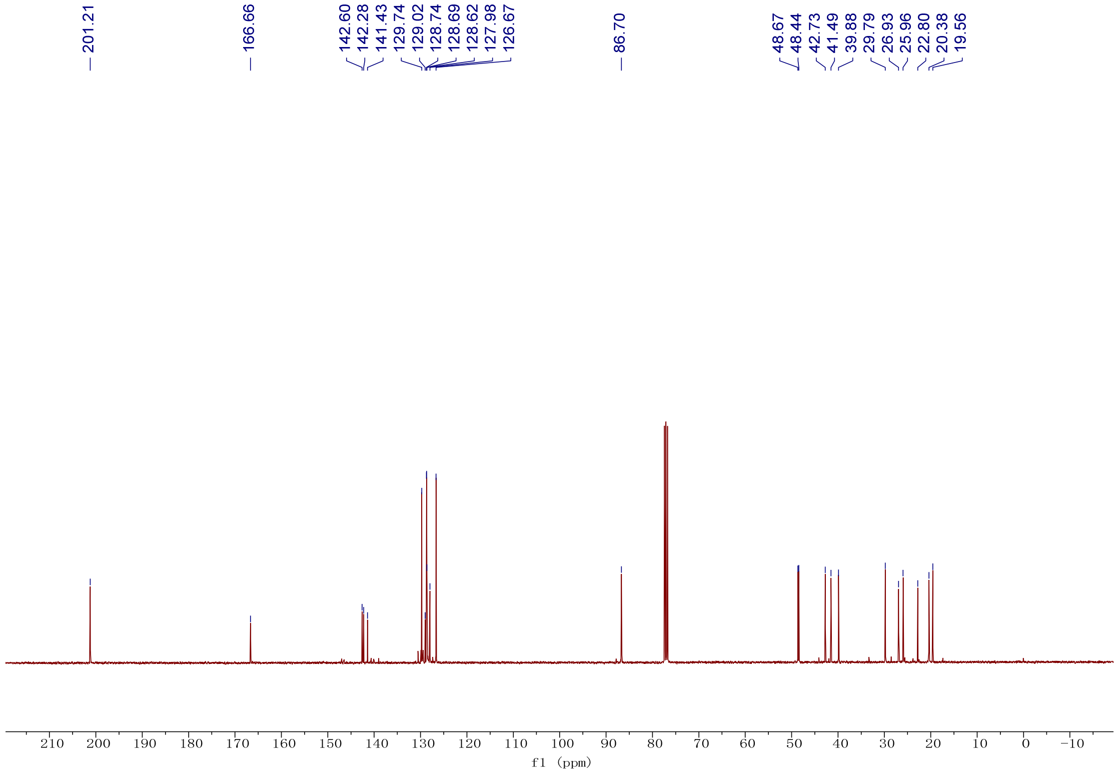

Supplementary Figure 47. ^{13}C NMR (101 MHz, $CDCl_3$) of **3j**

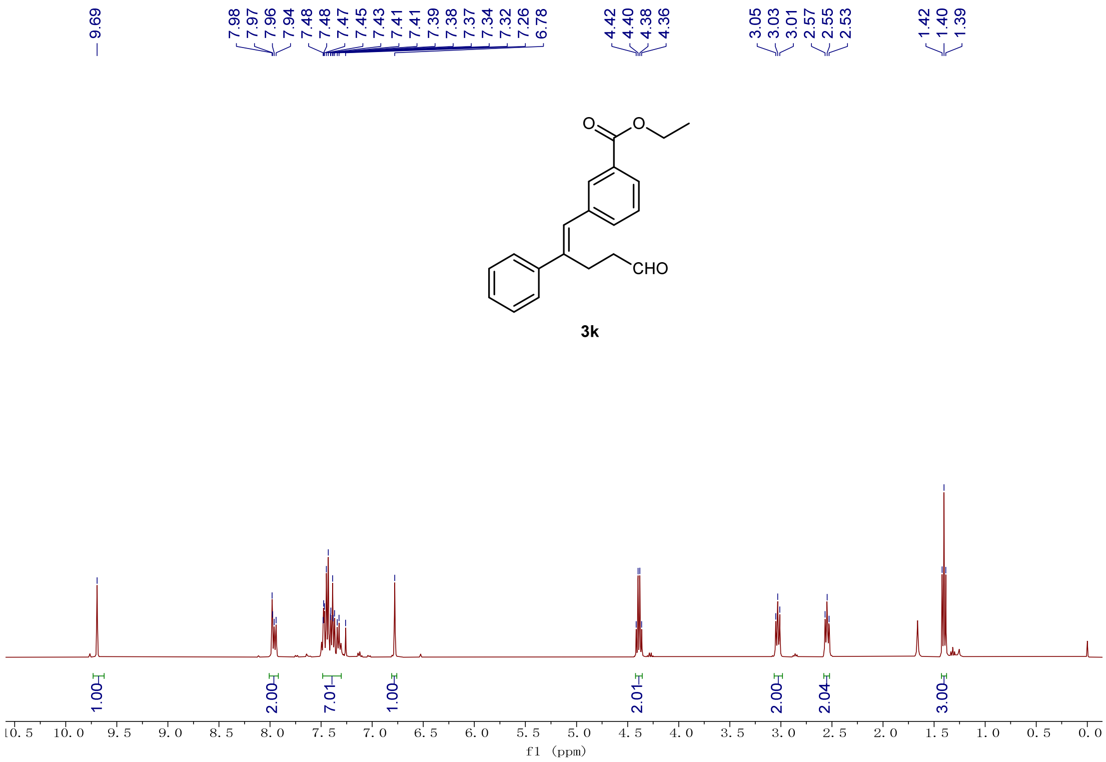

Supplementary Figure 48. ^{1}H NMR (400 MHz, $CDCl_3$) of **3k**

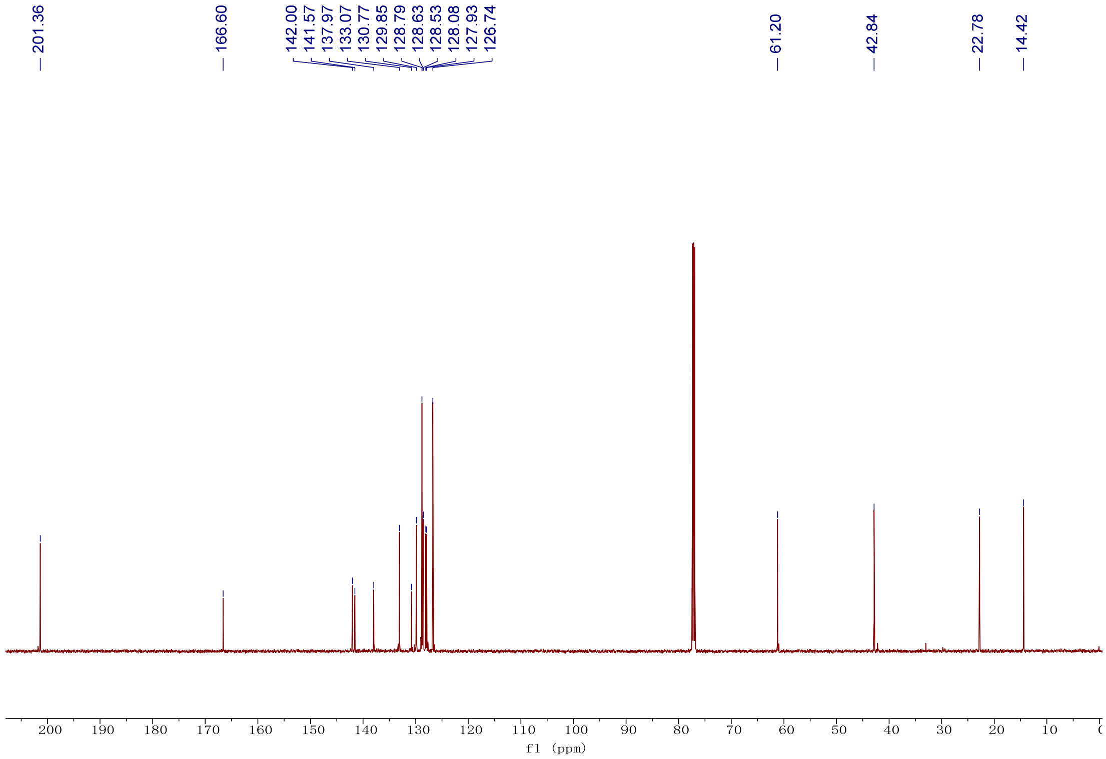

Supplementary Figure 49. ^{13}C NMR (101 MHz, $CDCl_3$) of **3k**

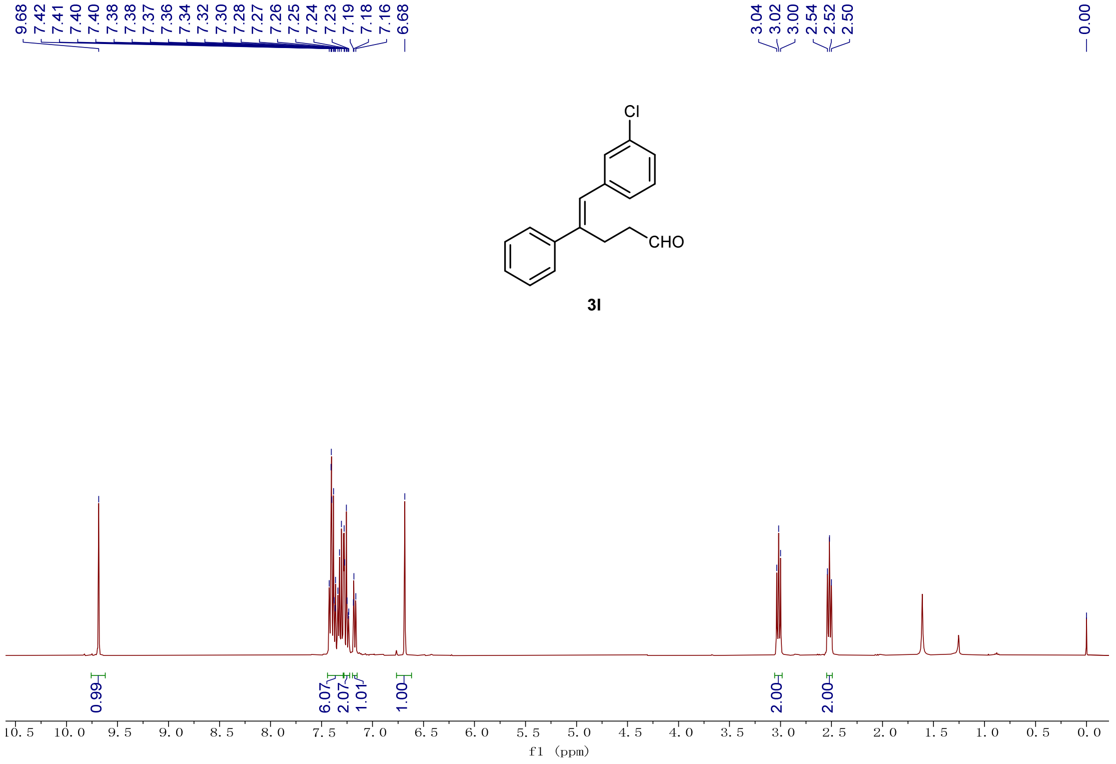

Supplementary Figure 50. ^{1}H NMR (400 MHz, $CDCl_3$) of **3l**

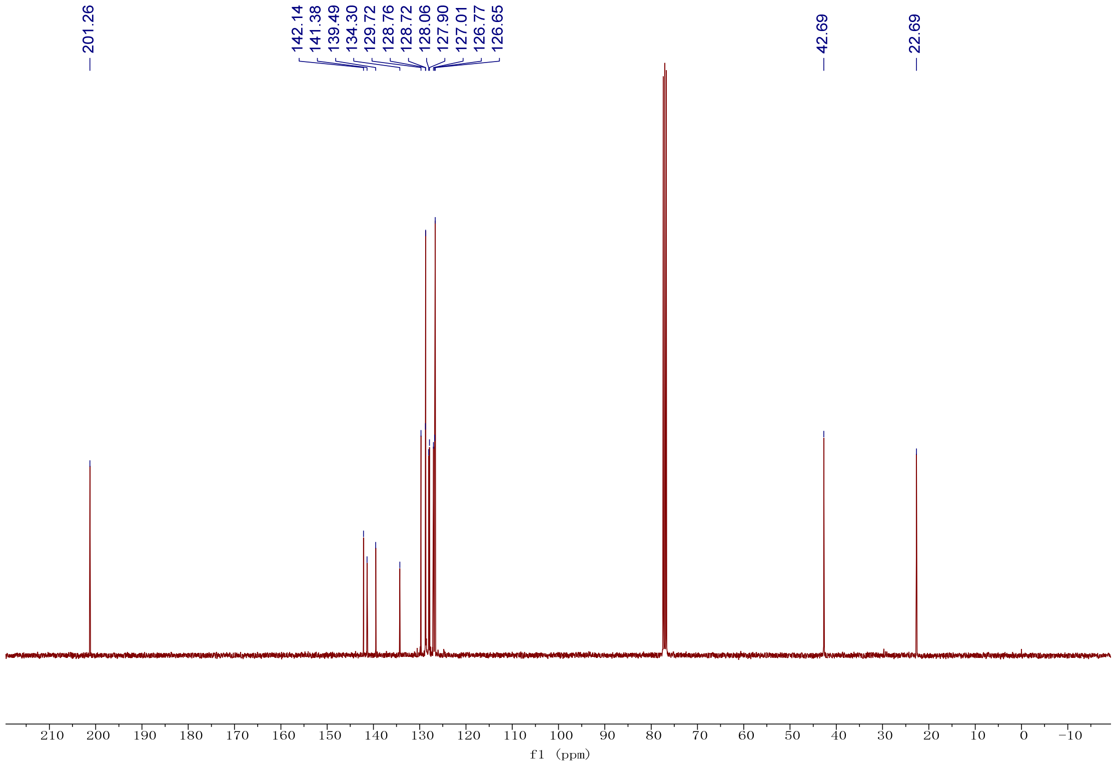

Supplementary Figure 51. ^{13}C NMR (101 MHz, $CDCl_3$) of **3l**

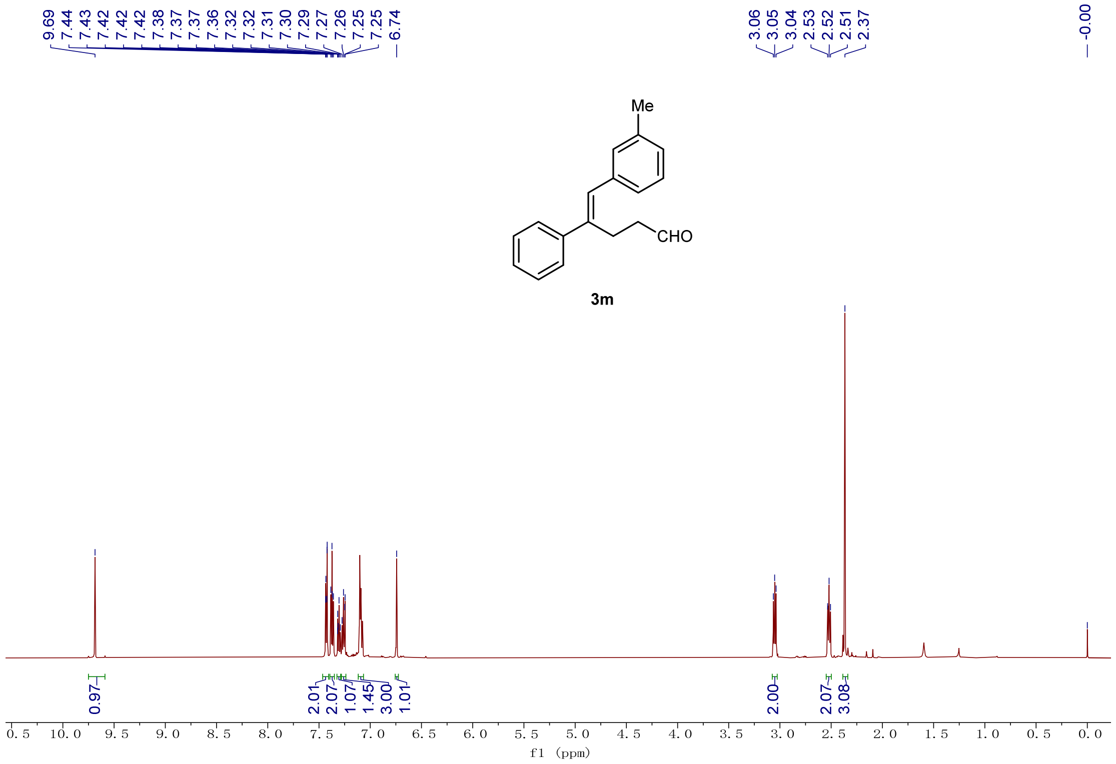

Supplementary Figure 52. ^{1}H NMR (600 MHz, $CDCl_3$) of **3m**

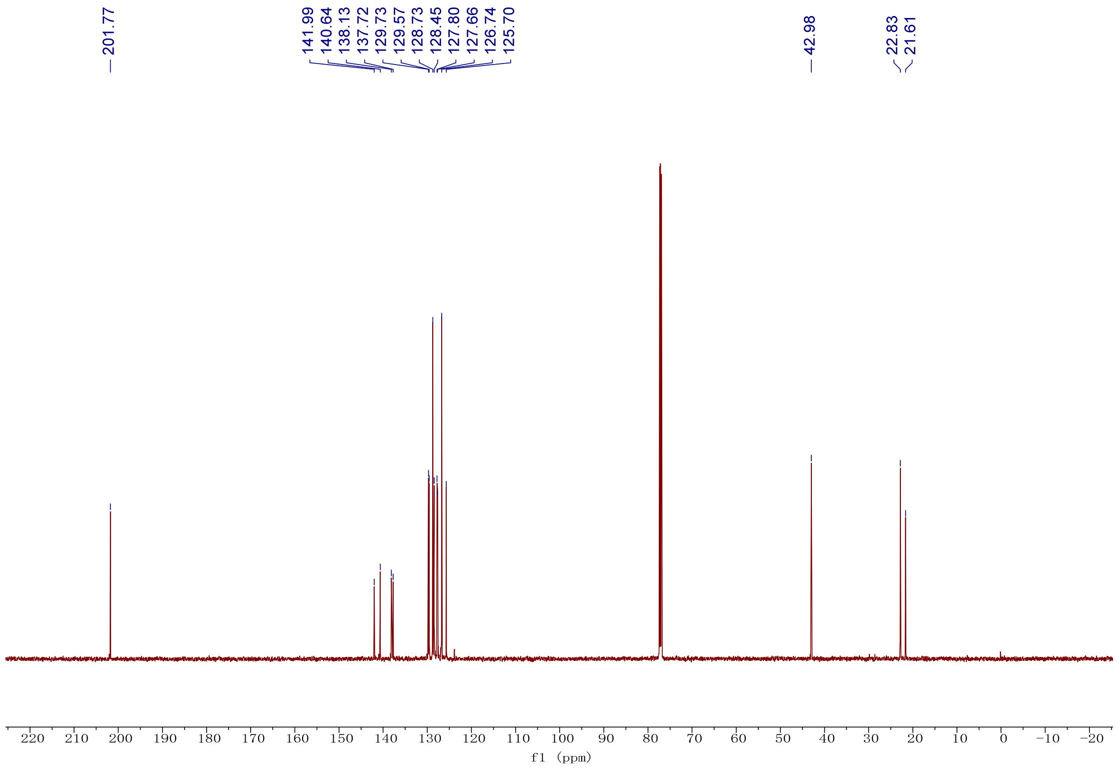

Supplementary Figure 53. ^{13}C NMR (151 MHz, $CDCl_3$) of **3m**

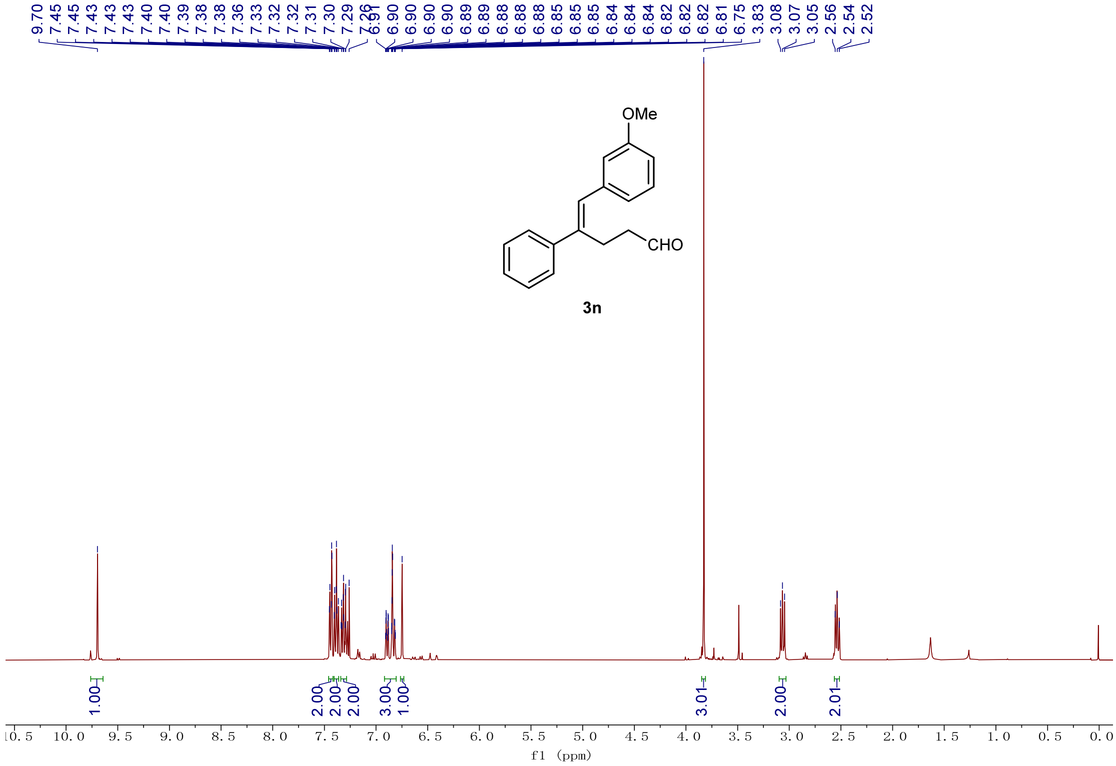

Supplementary Figure 54. ^{1}H NMR (400 MHz, $CDCl_3$) of **3n**

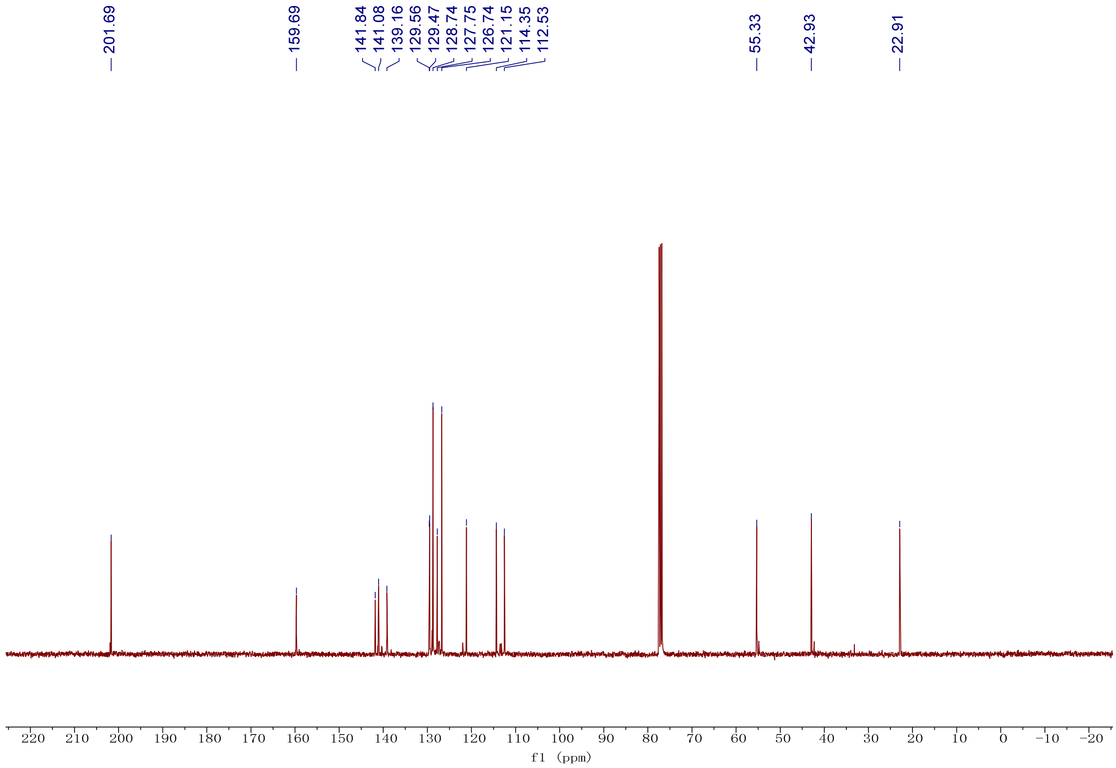

Supplementary Figure 55. ^{13}C NMR (101 MHz, $CDCl_3$) of **3n**

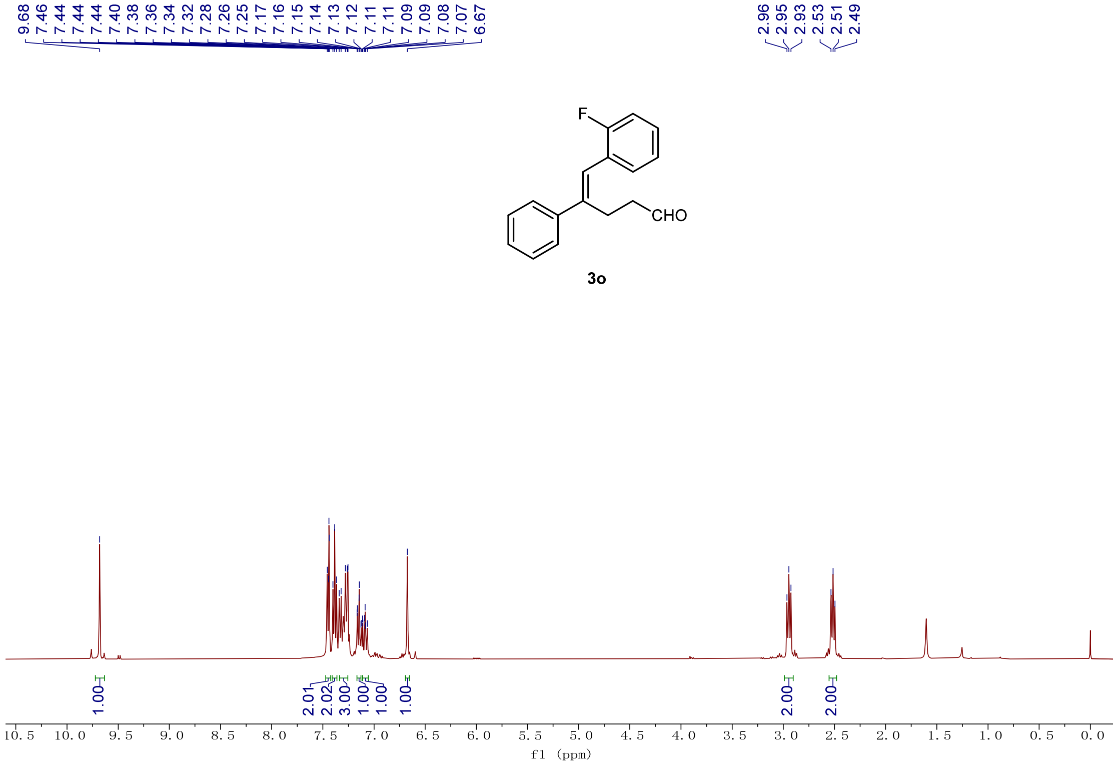

Supplementary Figure 56. ^{1}H NMR (400 MHz, $CDCl_3$) of **3o**

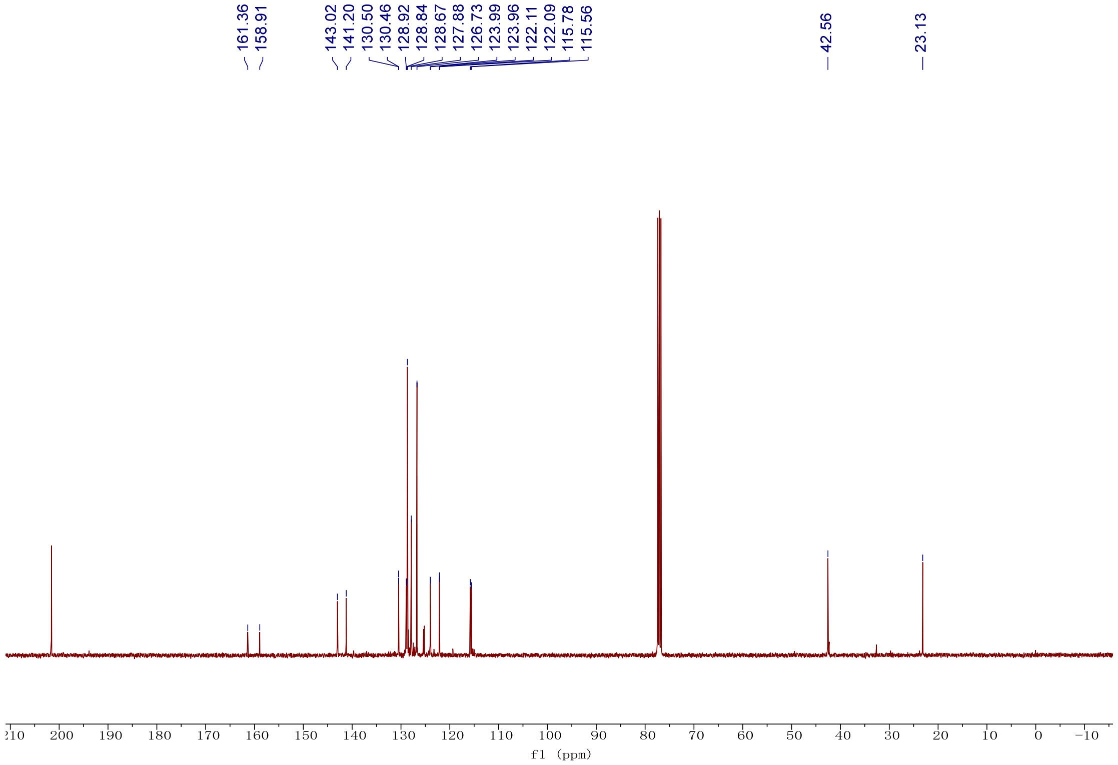

Supplementary Figure 57. ^{13}C NMR (101 MHz, $CDCl_3$) of **3o**

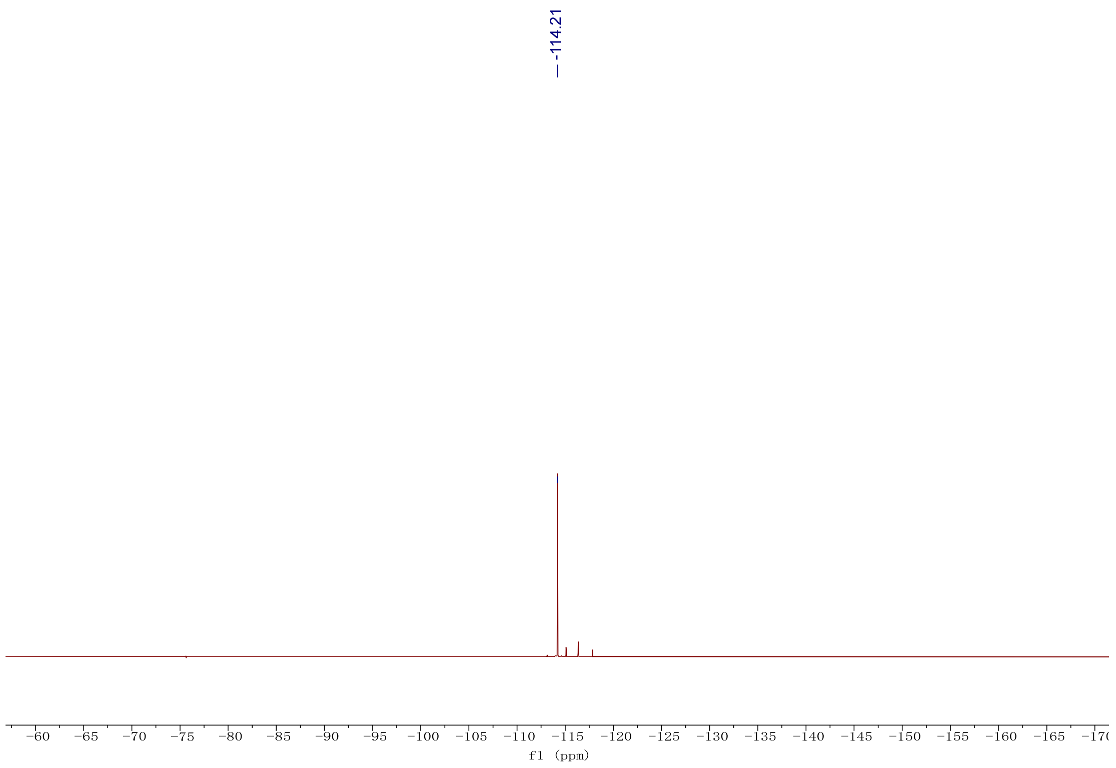

Supplementary Figure 58. ^{19}F NMR (376 MHz, $CDCl_3$) of **3o**

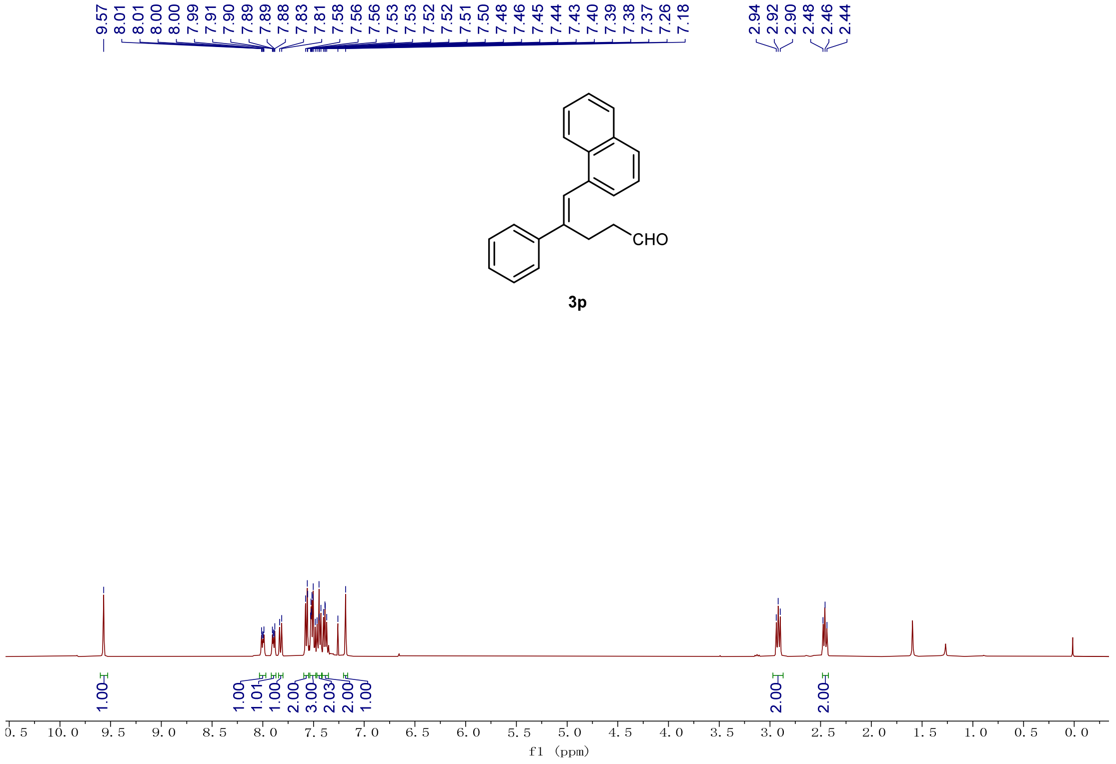

Supplementary Figure 59. ^{1}H NMR (400 MHz, $CDCl_3$) of **3p**

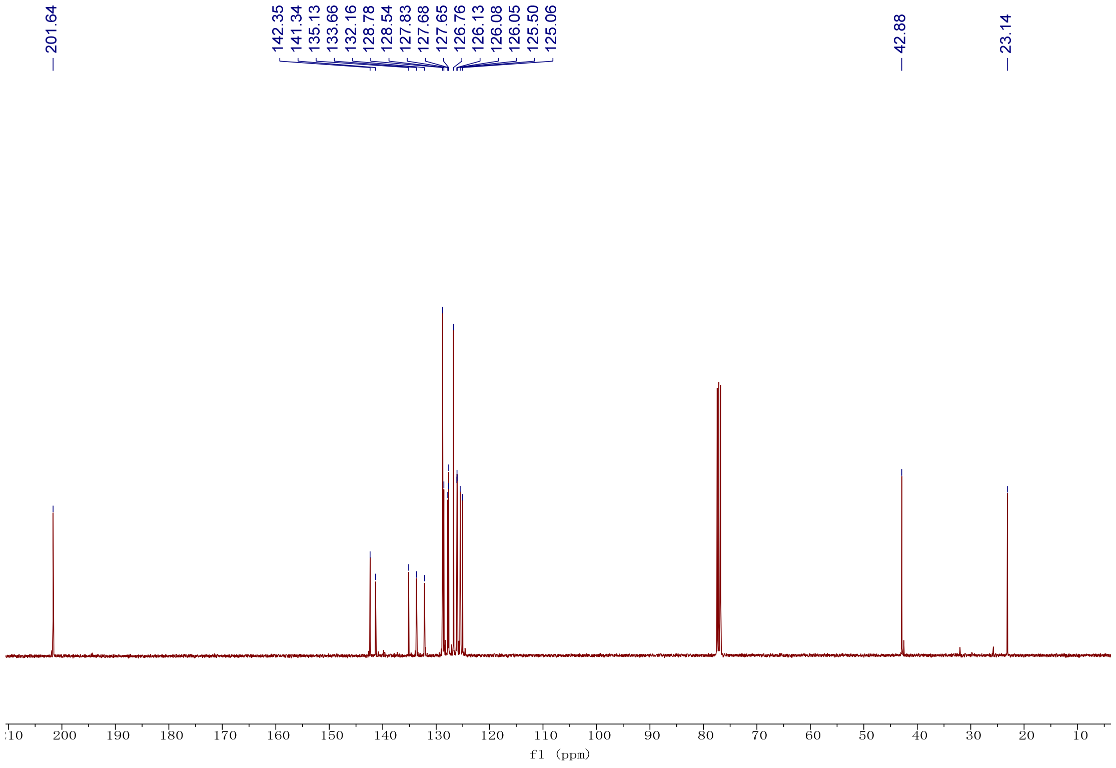

Supplementary Figure 60. ^{13}C NMR (101 MHz, $CDCl_3$) of **3p**

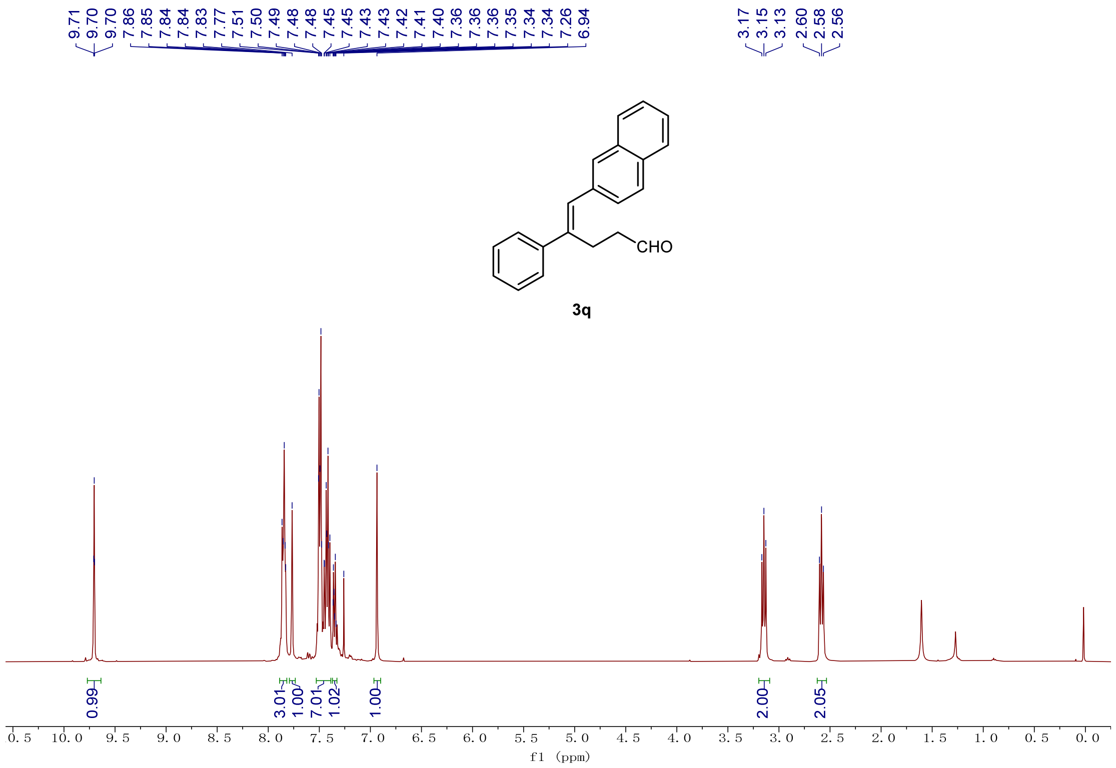

Supplementary Figure 61. ^{1}H NMR (400 MHz, $CDCl_3$) of **3q**

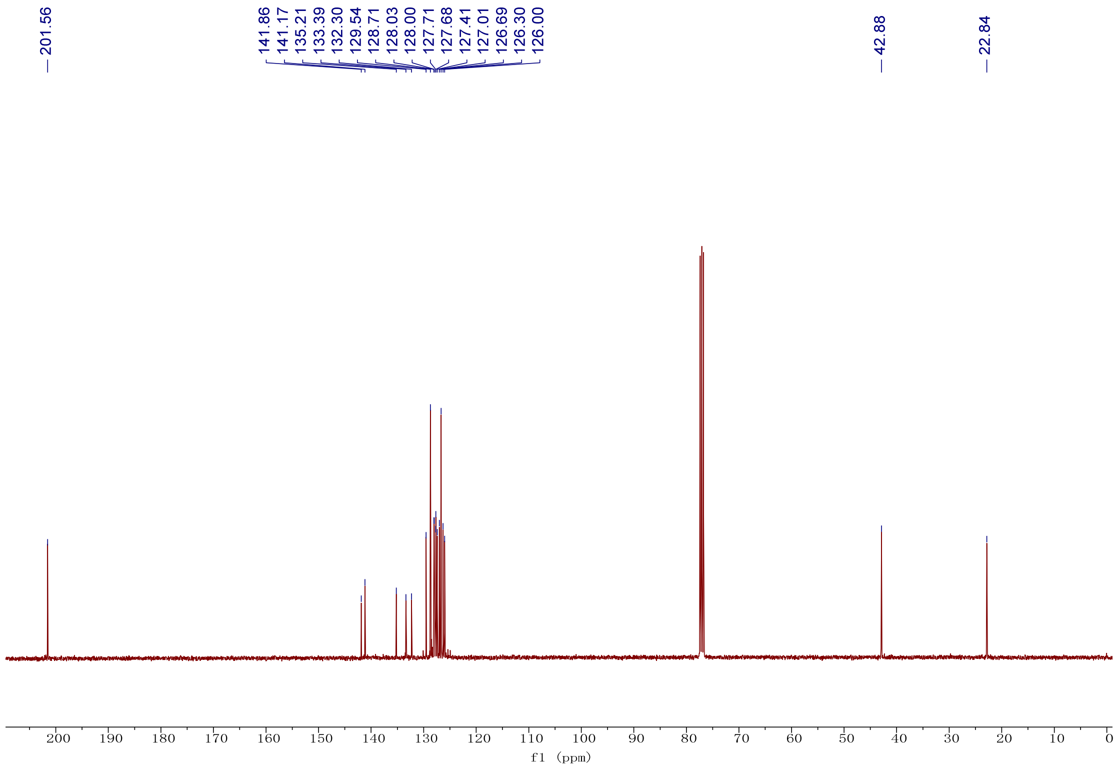

Supplementary Figure 62. ^{13}C NMR (101 MHz, $CDCl_3$) of **3q**

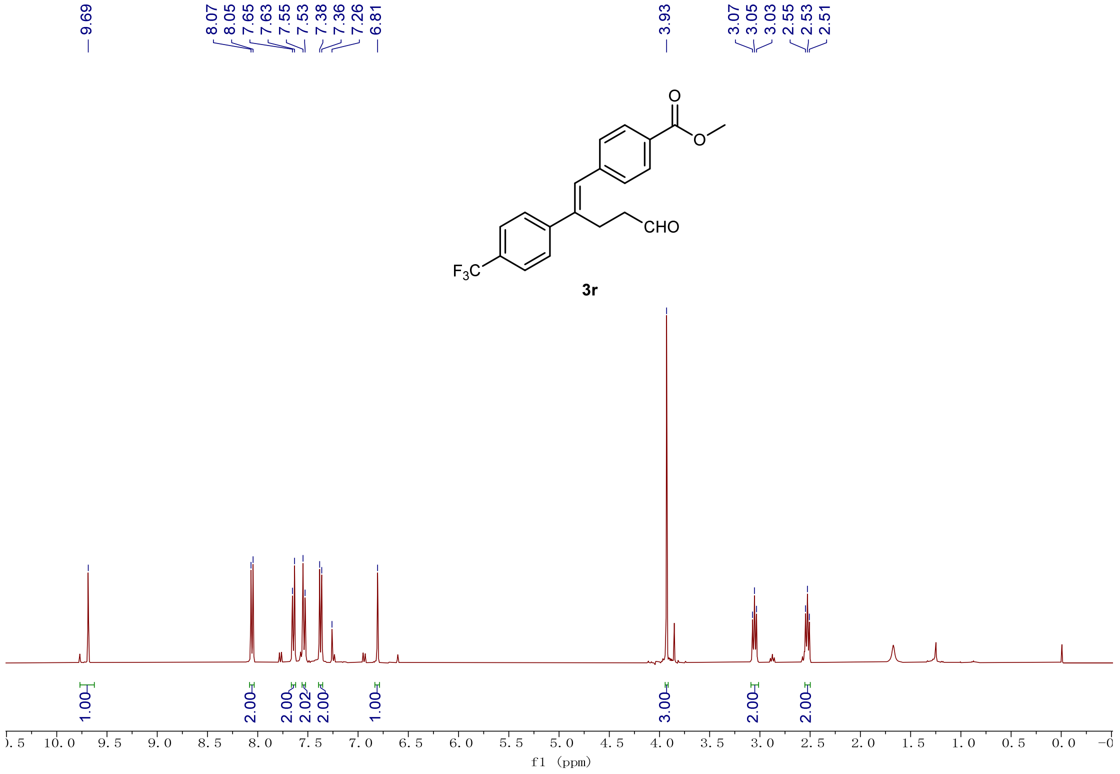

Supplementary Figure 63. ^{1}H NMR (400 MHz, $CDCl_3$) of **3r**

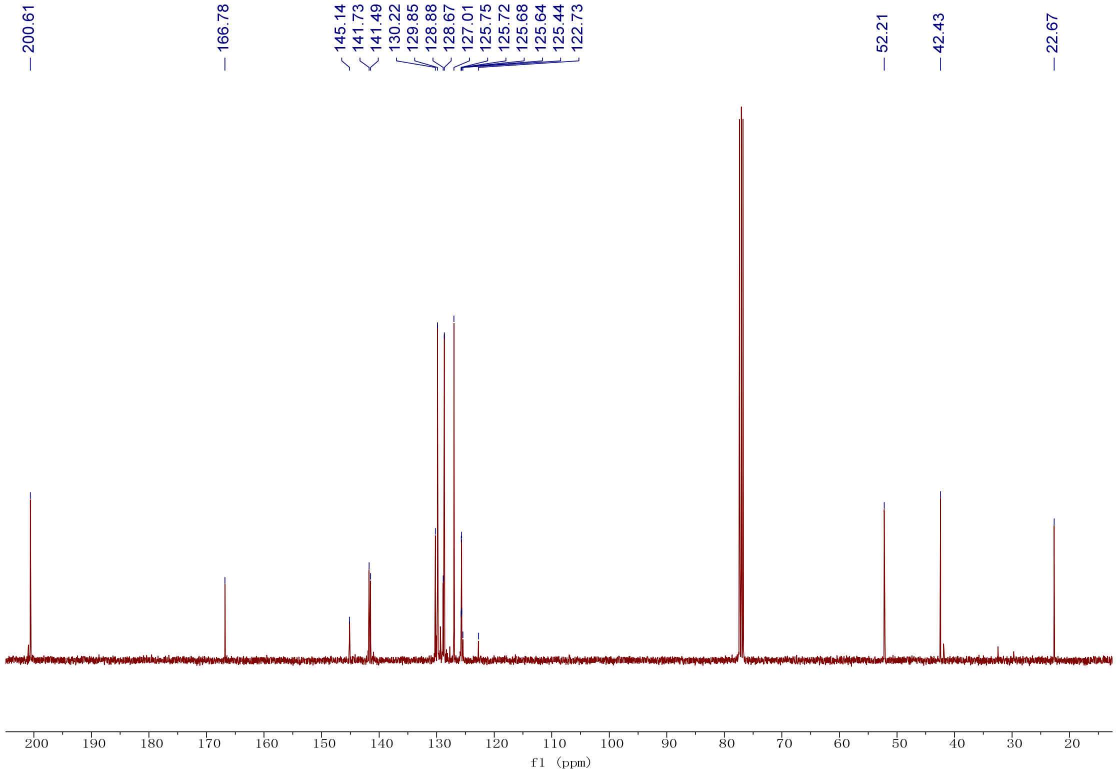

Supplementary Figure 64. ^{13}C NMR (101 MHz, $CDCl_3$) of **3r**

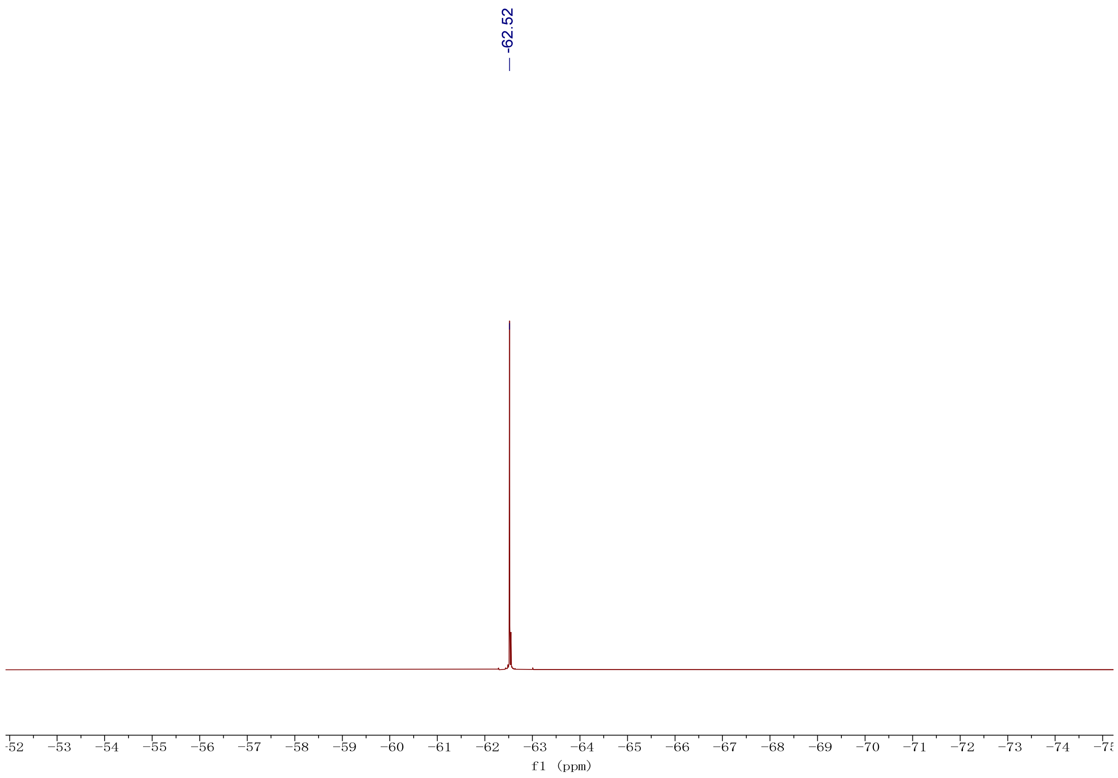

Supplementary Figure 65. ^{19}F NMR (376 MHz, $CDCl_3$) of **3r**

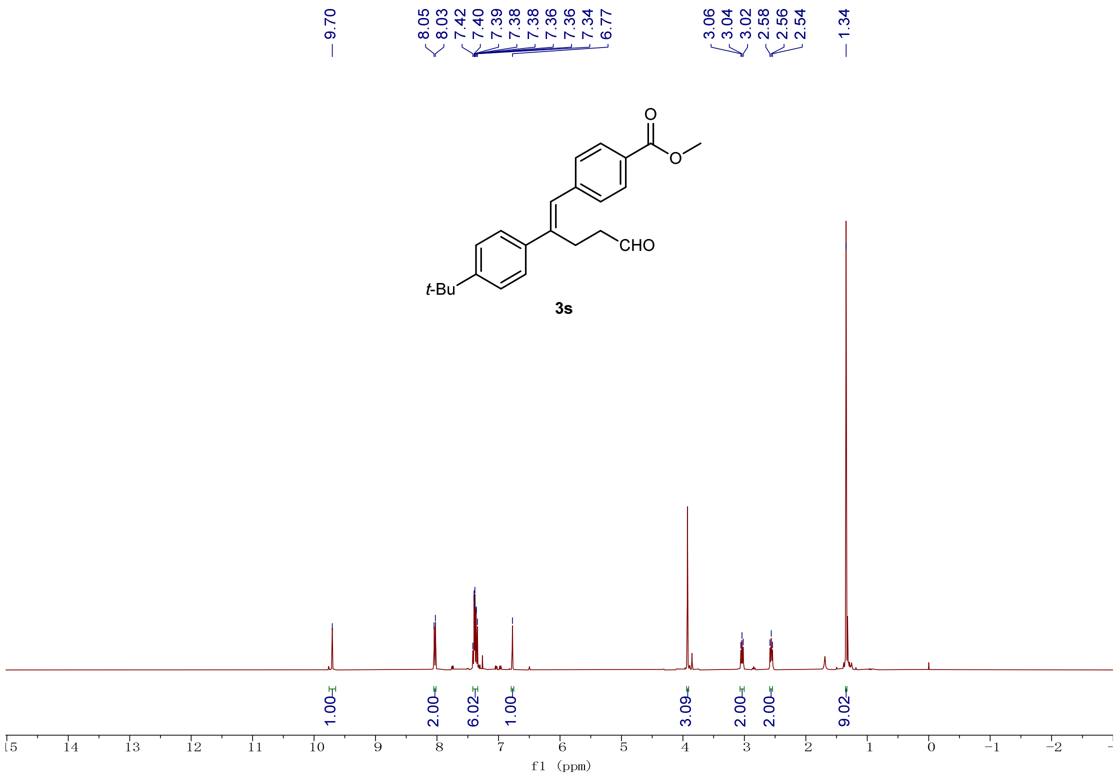

Supplementary Figure 66. ^{1}H NMR (400 MHz, $CDCl_3$) of **3s**

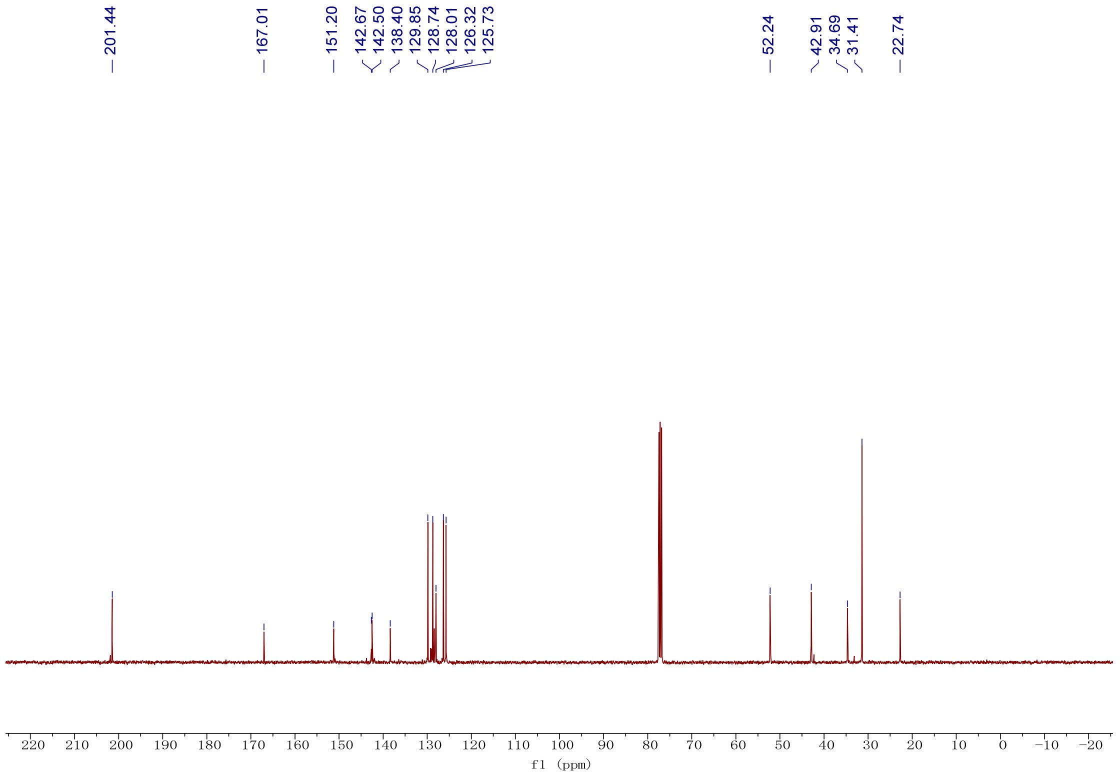

Supplementary Figure 67. ^{13}C NMR (101 MHz, $CDCl_3$) of **3s**

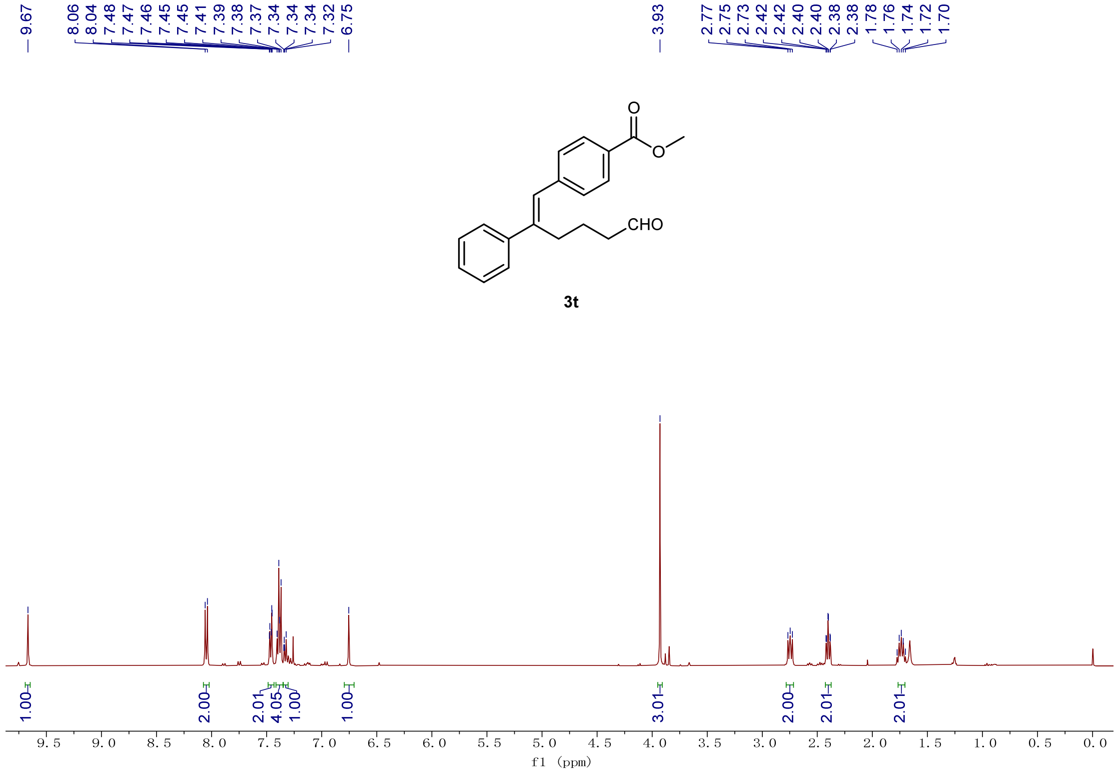

Supplementary Figure 68. ^{1}H NMR (400 MHz, $CDCl_3$) of **3t**

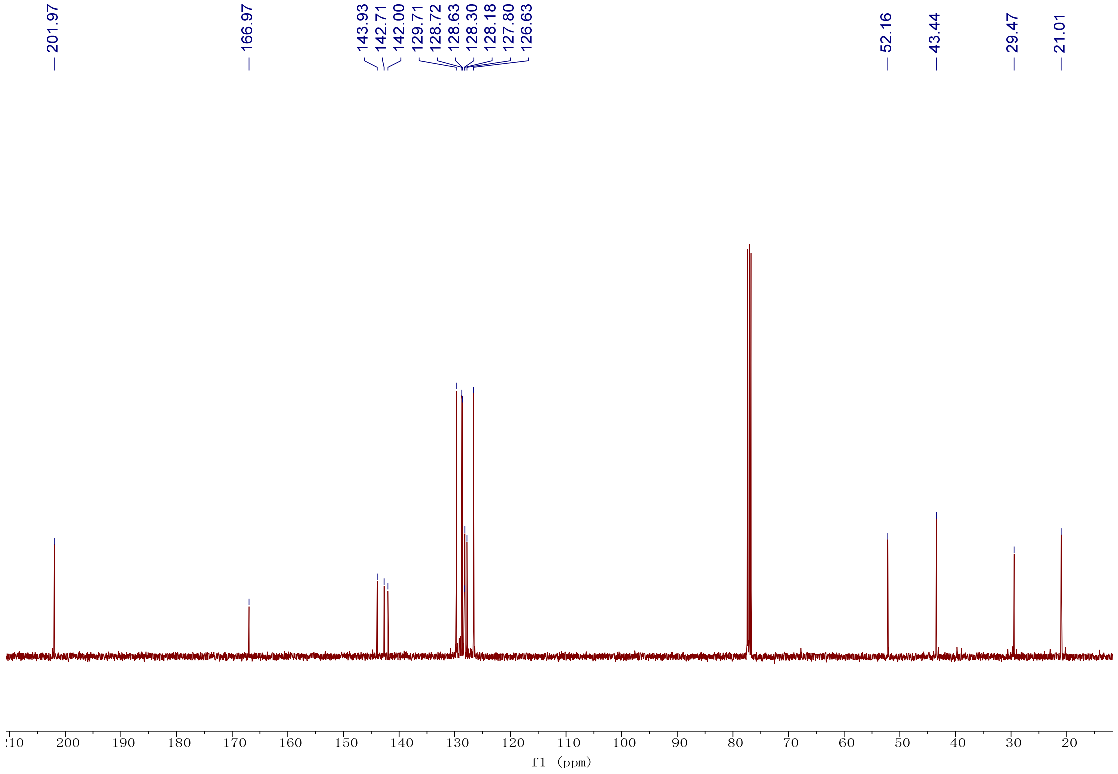

Supplementary Figure 69. ^{13}C NMR (101 MHz, $CDCl_3$) of **3t**

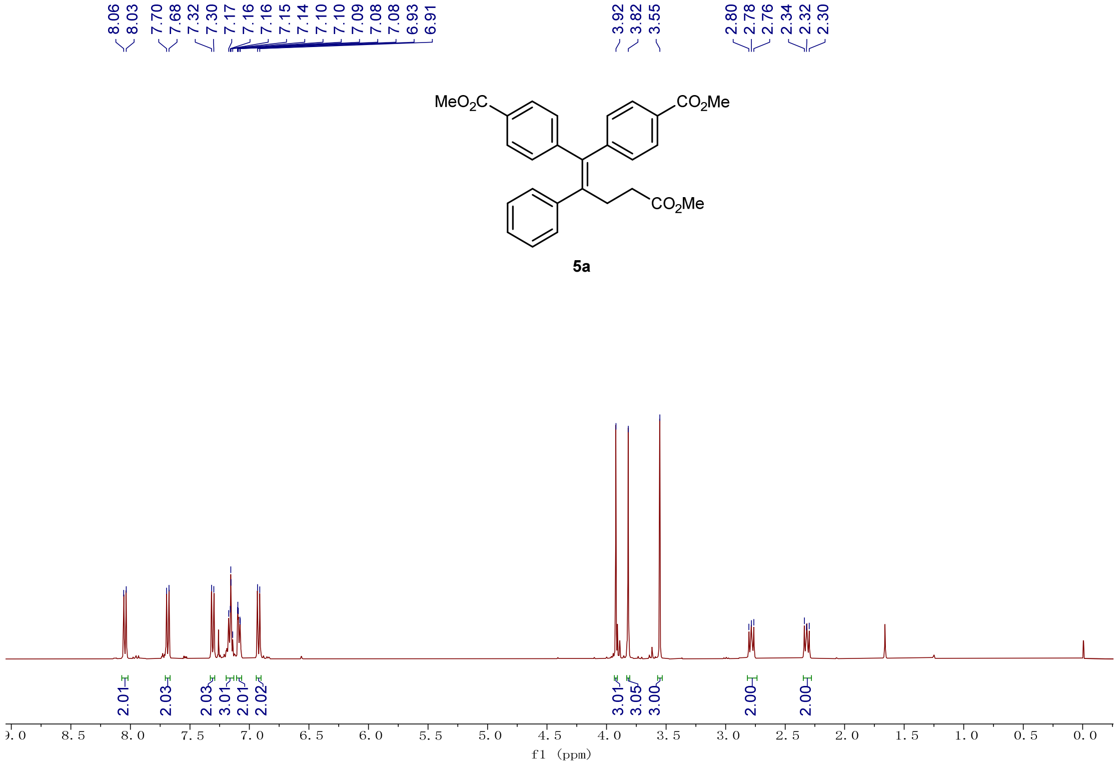

Supplementary Figure 70. ^{1}H NMR (400 MHz, $CDCl_3$) of **5a**

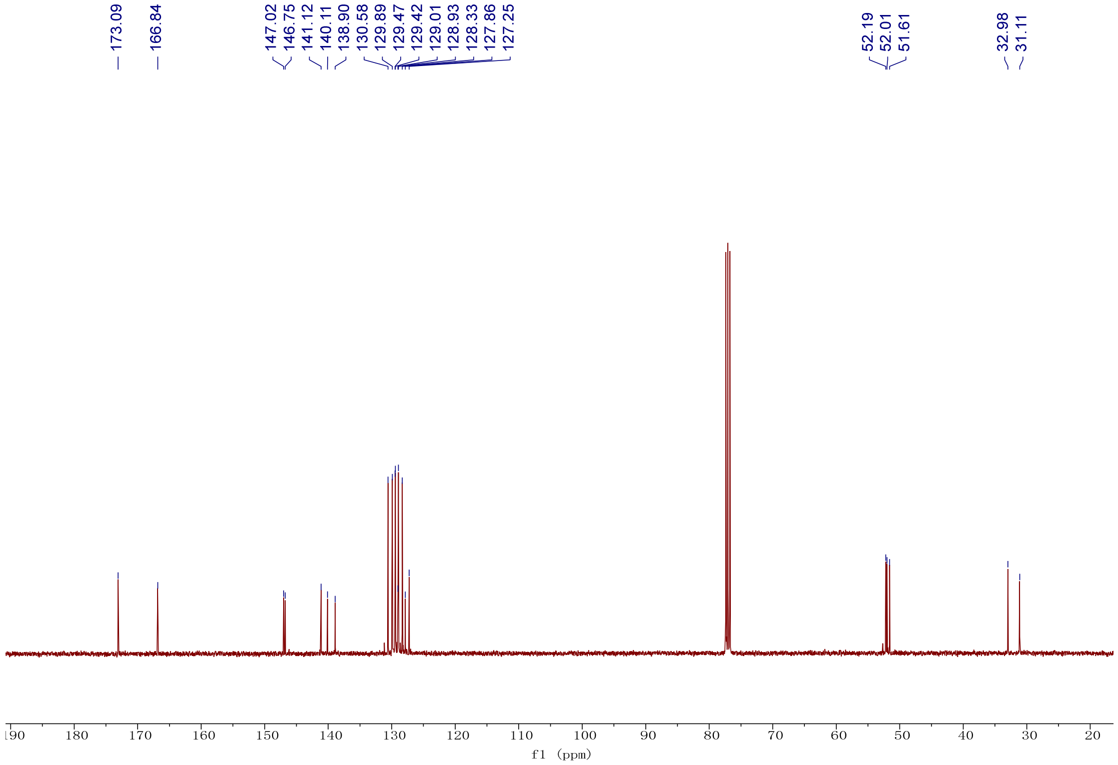

Supplementary Figure 71. ^{13}C NMR (101 MHz, $CDCl_3$) of **5a**

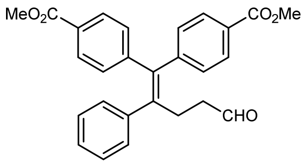

5b

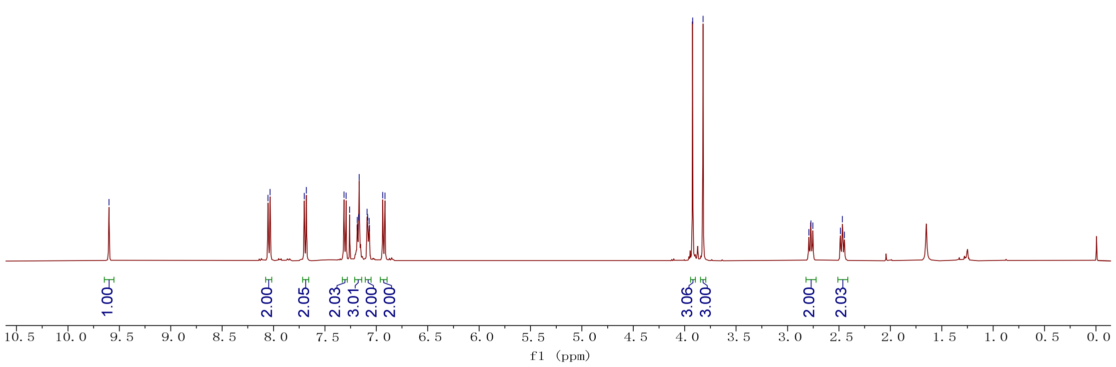

Supplementary Figure 72. ^{1}H NMR (400 MHz, $CDCl_3$) of **5b**

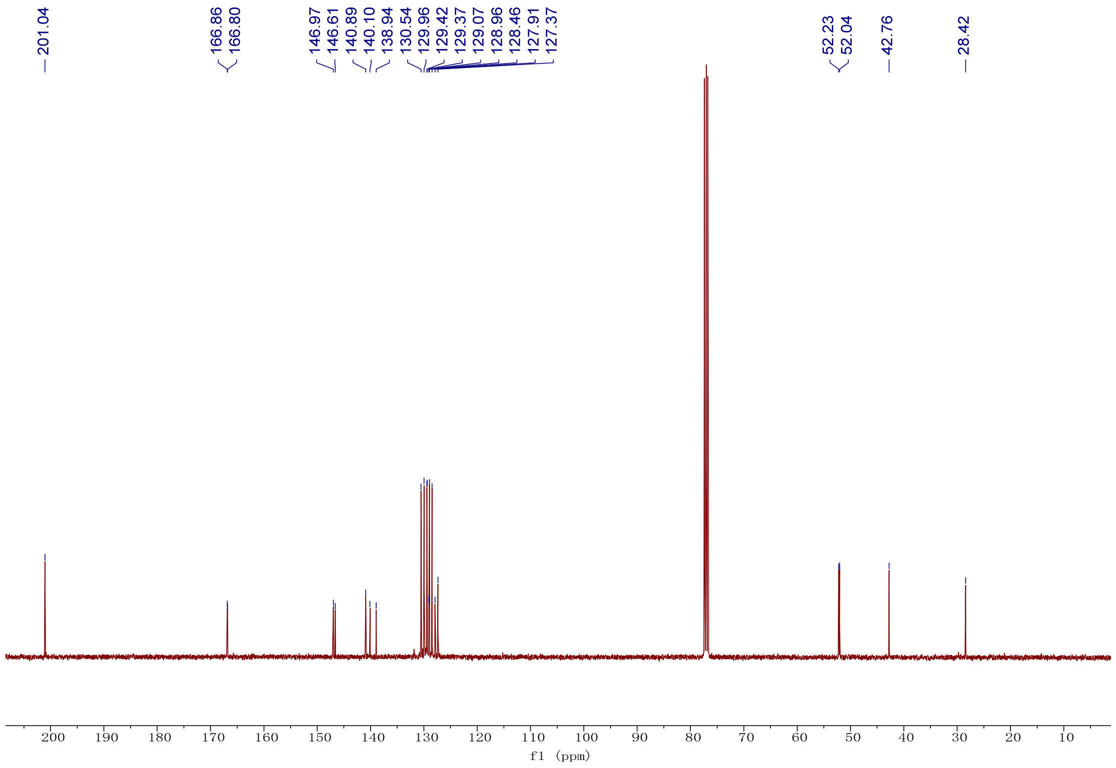

Supplementary Figure 73. ^{13}C NMR (101 MHz, $CDCl_3$) of **5b**

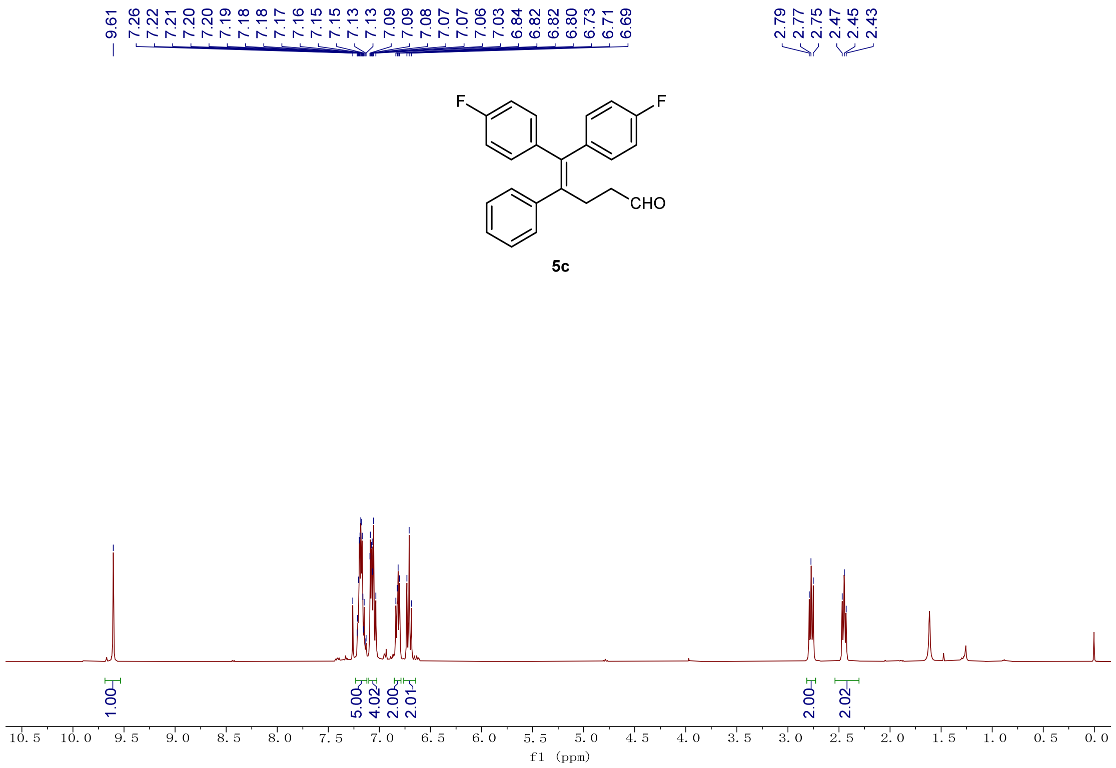

Supplementary Figure 74. ^{1}H NMR (400 MHz, $CDCl_3$) of **5c**

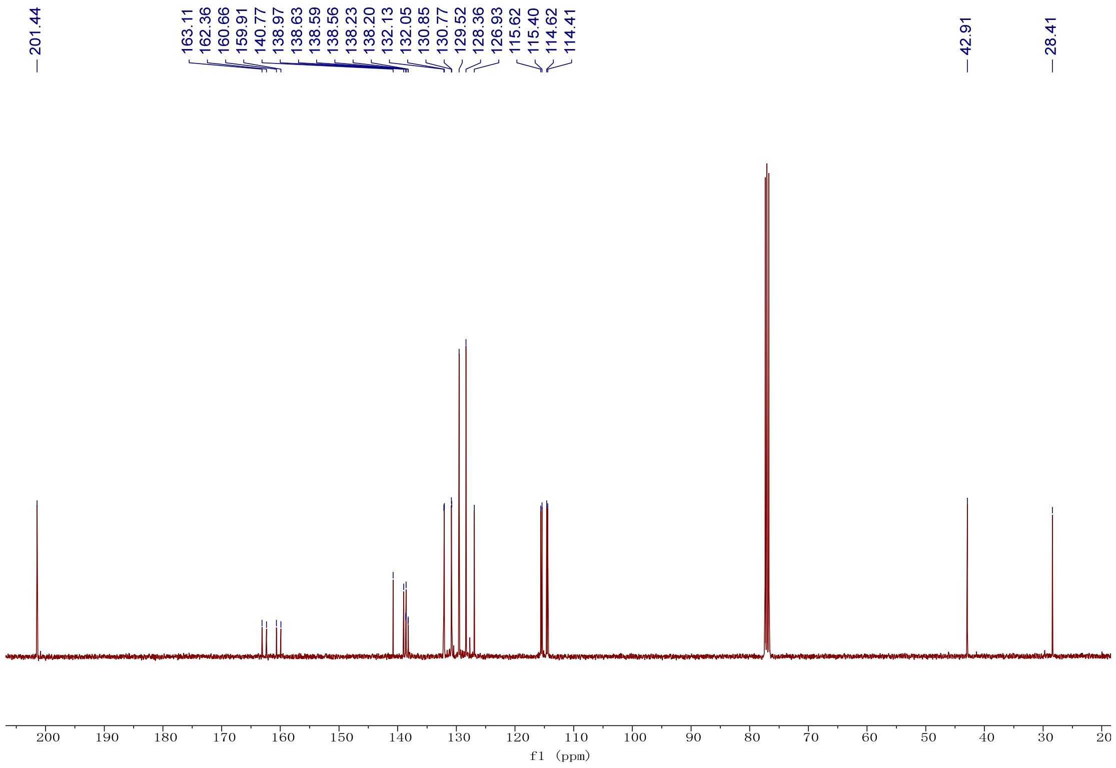

Supplementary Figure 75. ^{13}C NMR (101 MHz, $CDCl_3$) of **5c**

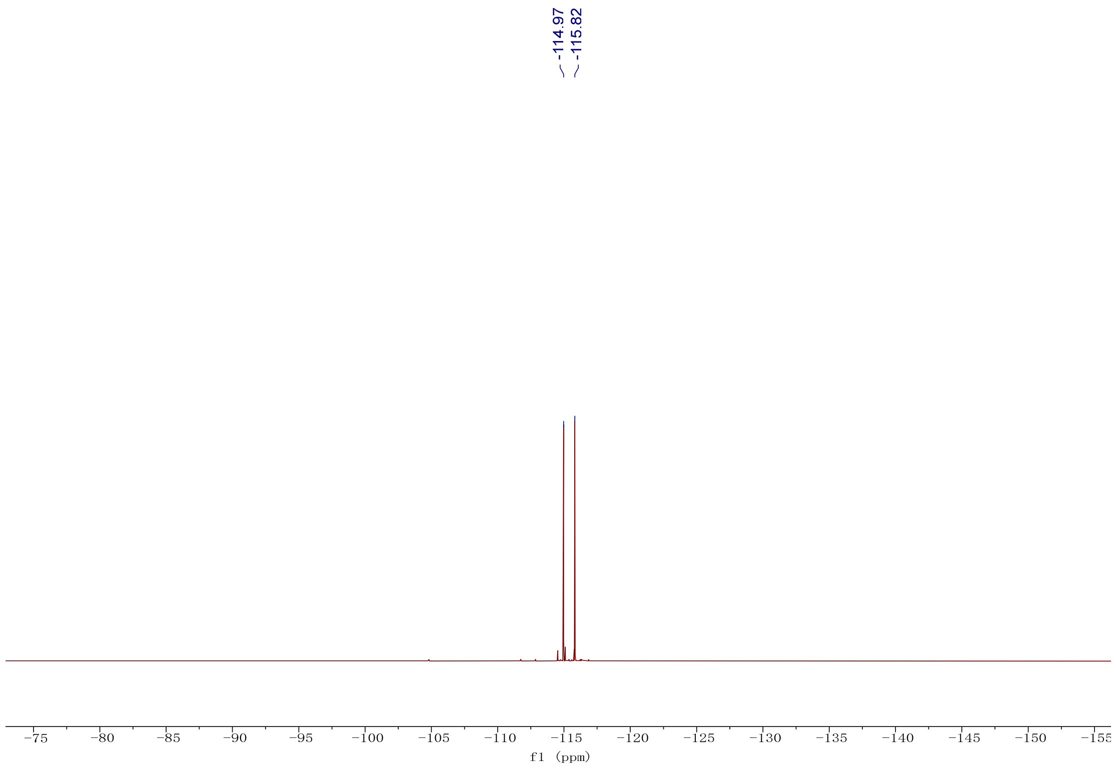

Supplementary Figure 76. ^{19}F NMR (376 MHz, $CDCl_3$) of **5c**

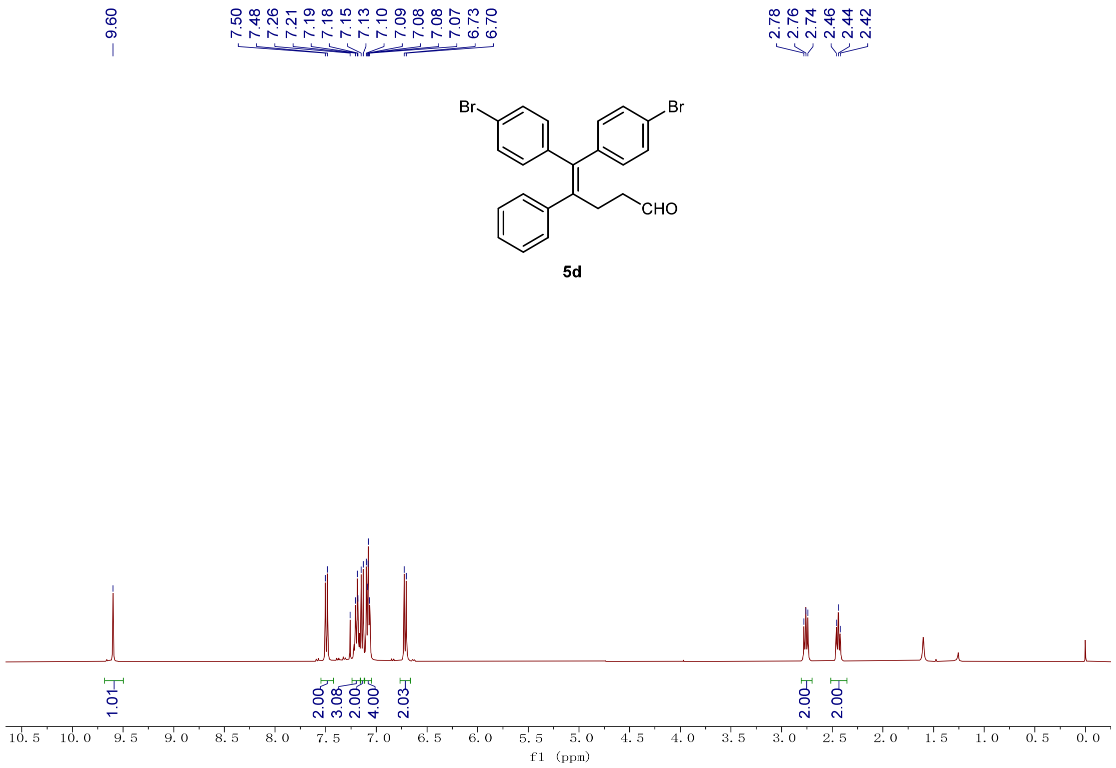

Supplementary Figure 77. ^{1}H NMR (400 MHz, $CDCl_3$) of **5d**

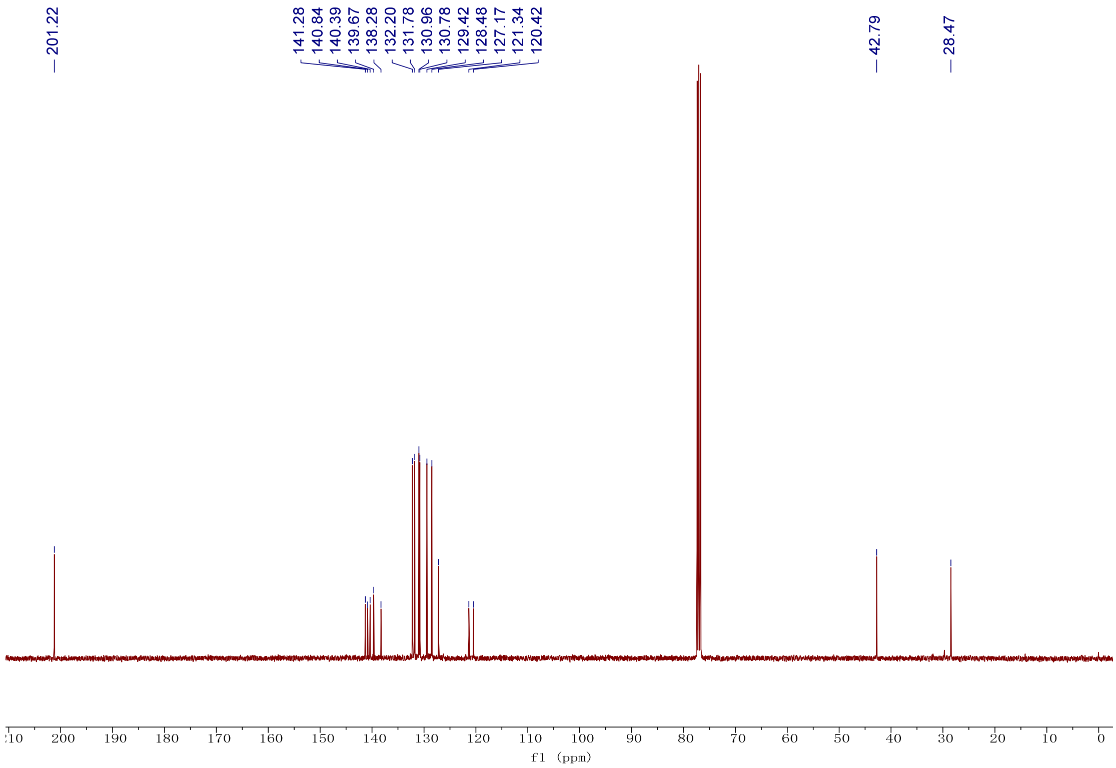

Supplementary Figure 78. ^{13}C NMR (101 MHz, $CDCl_3$) of **5d**

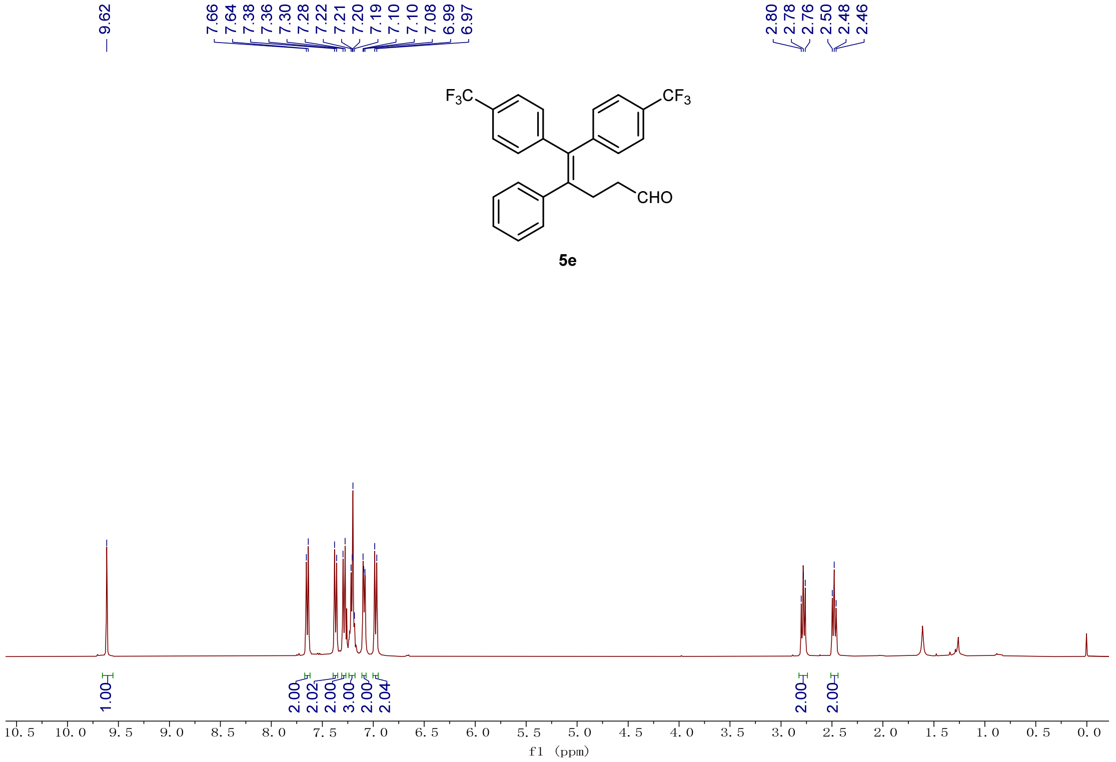

Supplementary Figure 79. ^{1}H NMR (400 MHz, $CDCl_3$) of **5e**

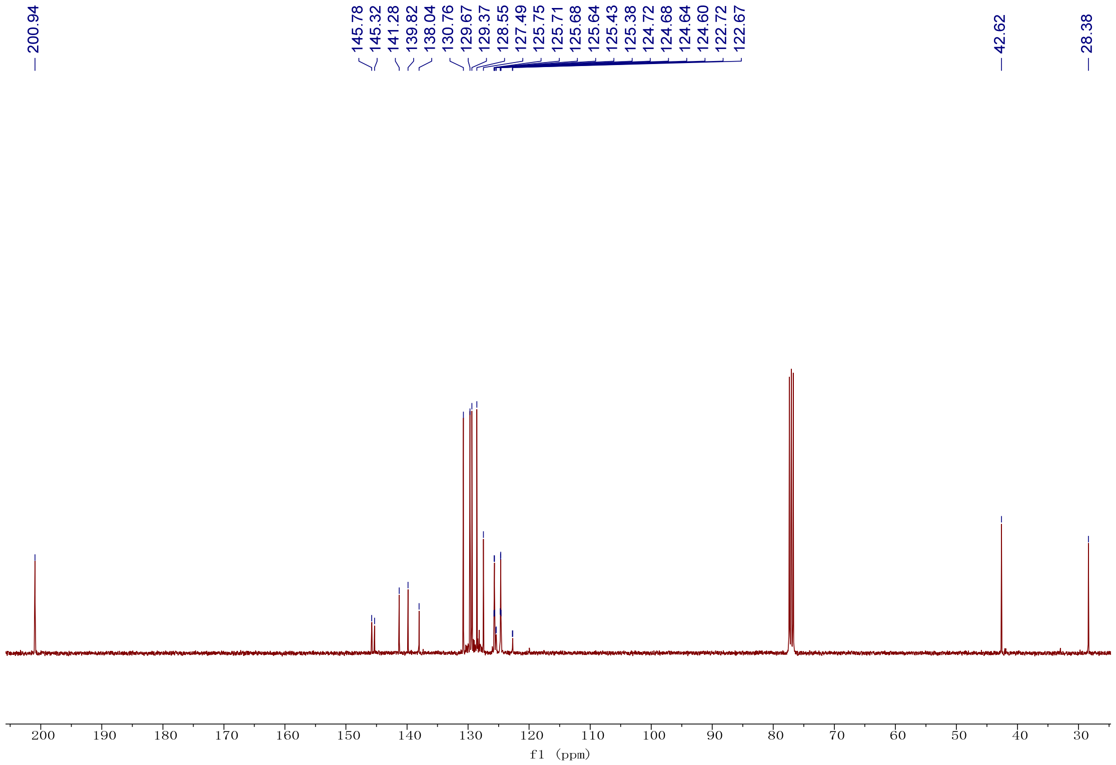

Supplementary Figure 80. ^{13}C NMR (101 MHz, $CDCl_3$) of **5e**

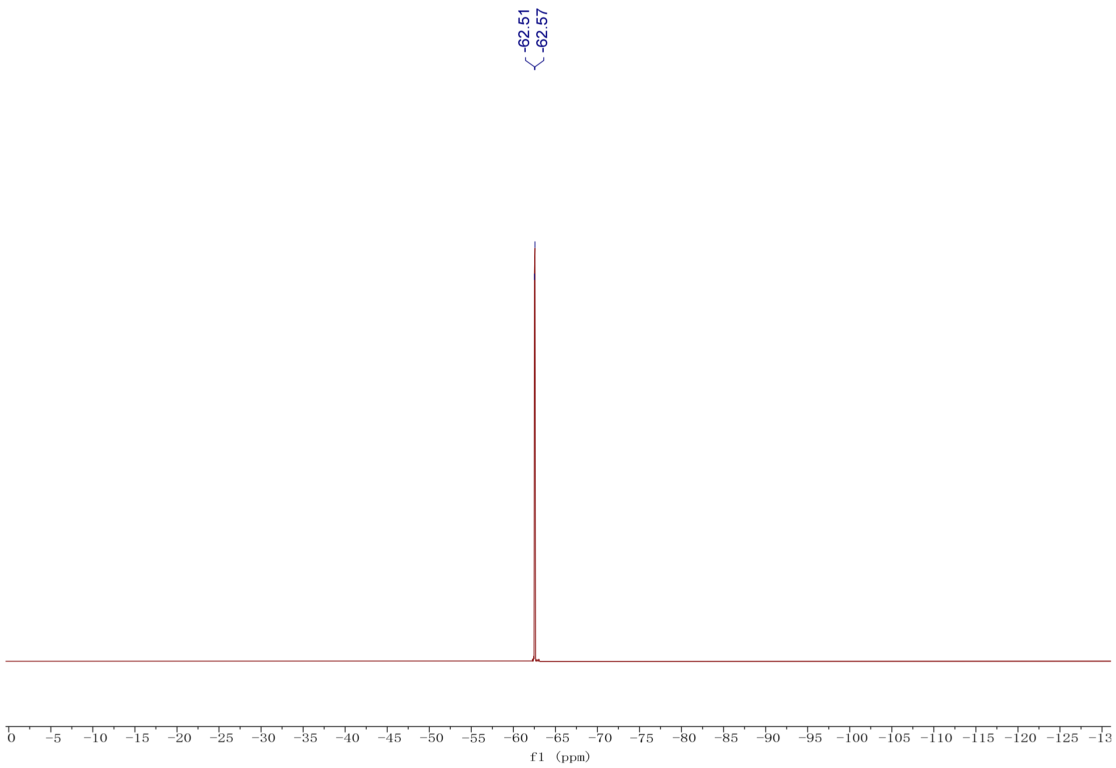

Supplementary Figure 81. ^{19}F NMR (376 MHz, $CDCl_3$) of **5e**

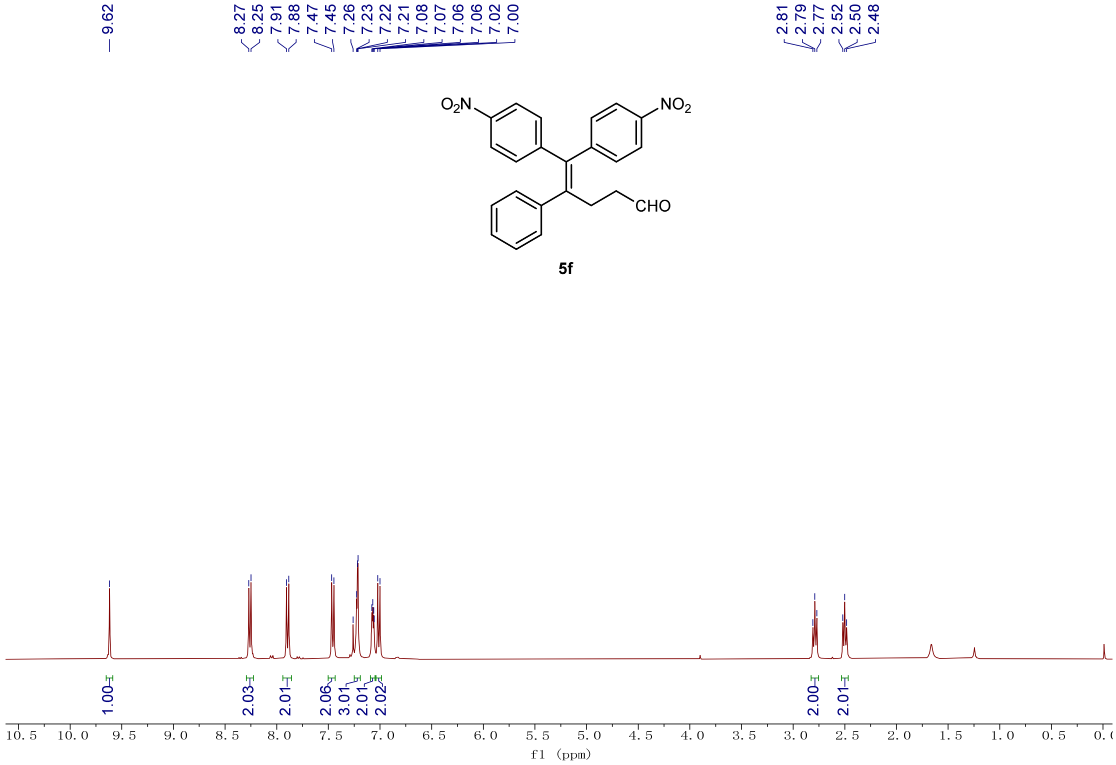

Supplementary Figure 82. ^{1}H NMR (400 MHz, $CDCl_3$) of **5f**

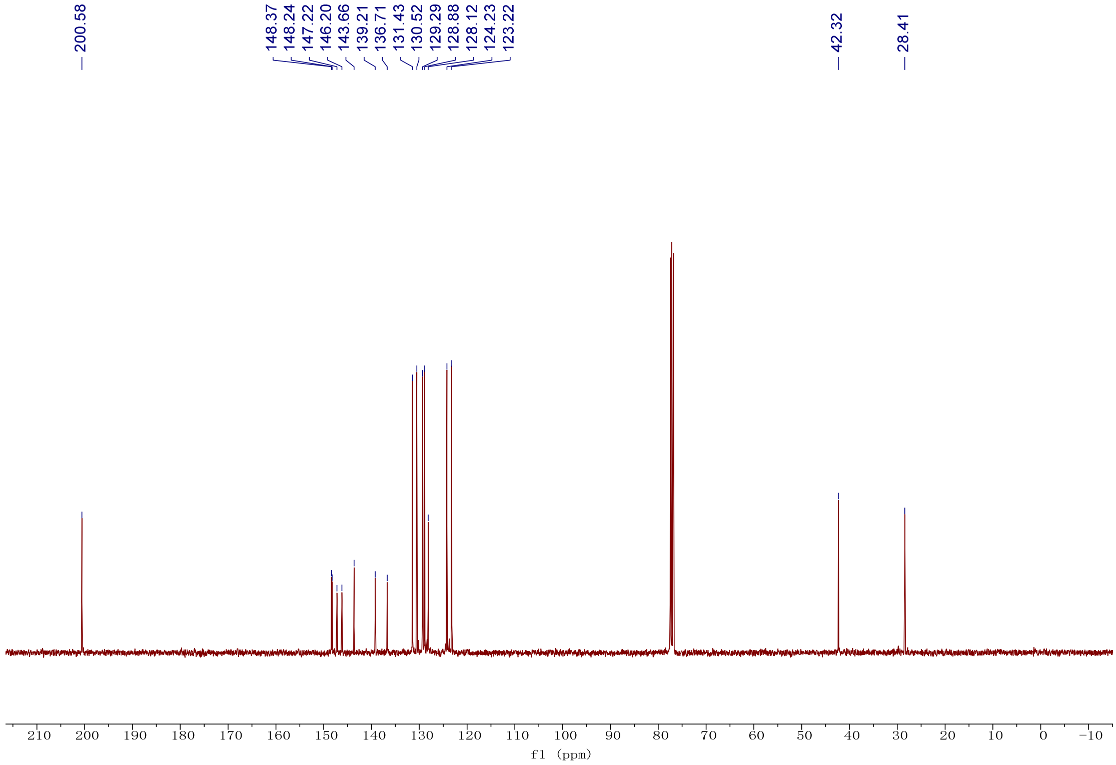

Supplementary Figure 83. ^{13}C NMR (101 MHz, $CDCl_3$) of **5f**

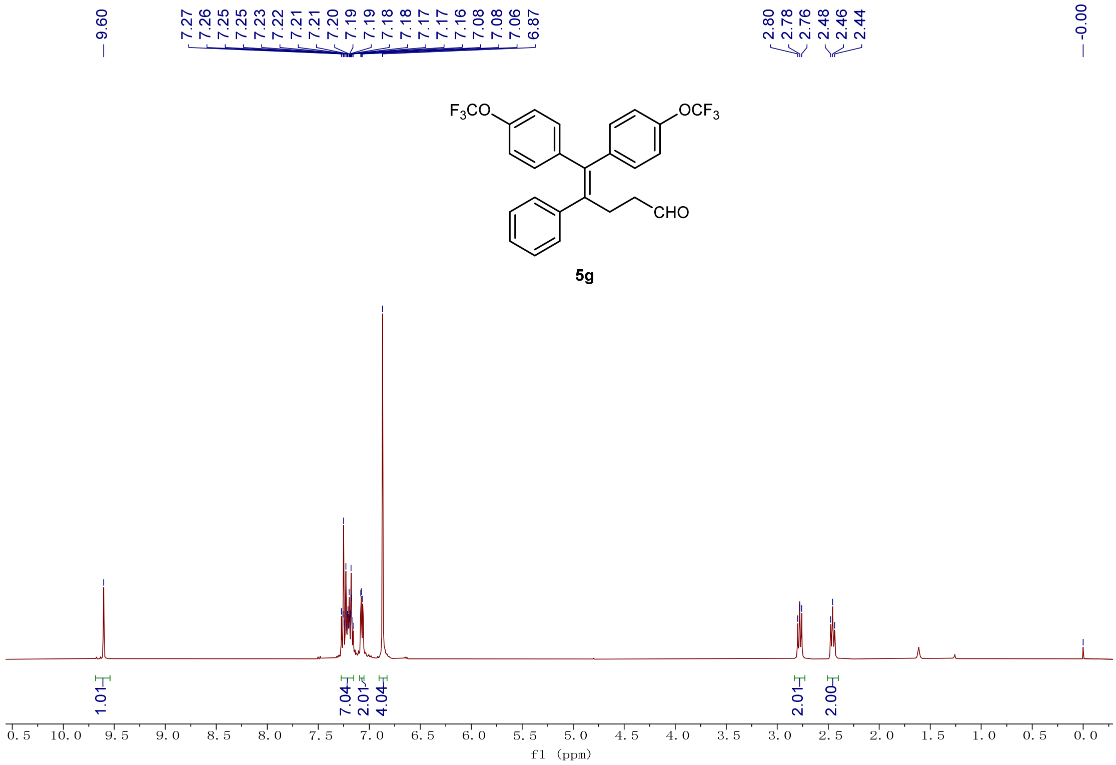

Supplementary Figure 84. ¹H NMR (400 MHz, $CDCl_3$) of **5g**

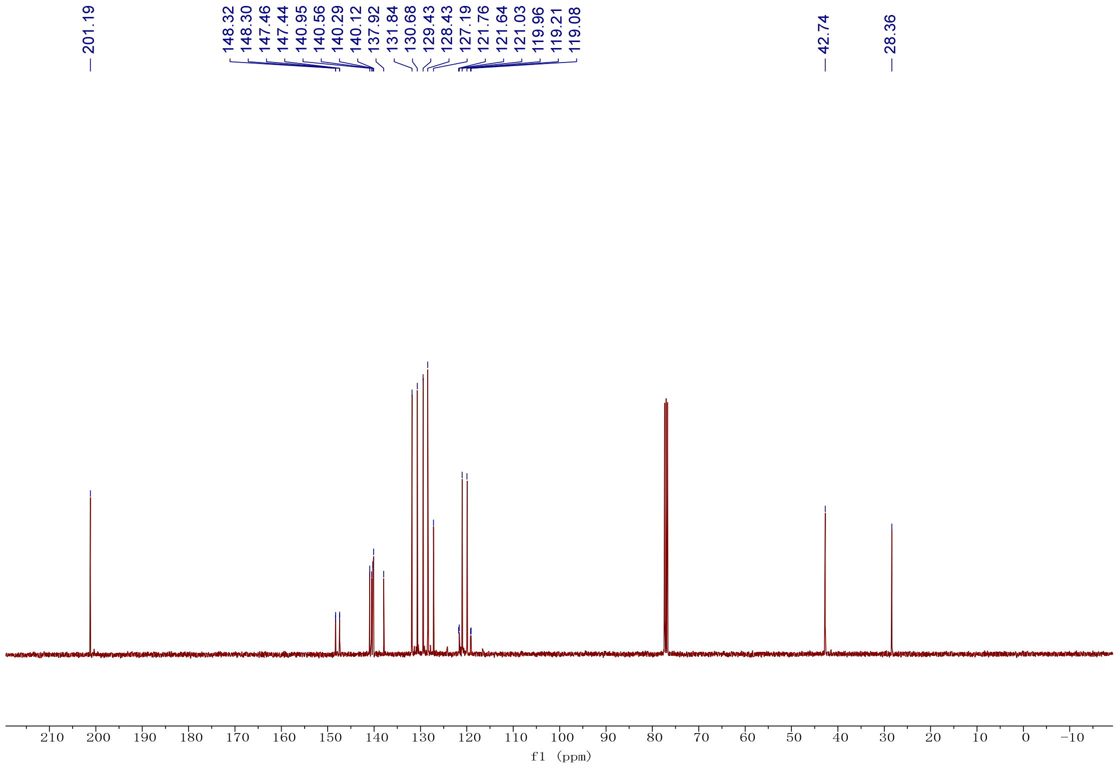

Supplementary Figure 85. ^{13}C NMR (101 MHz, $CDCl_3$) of **5g**

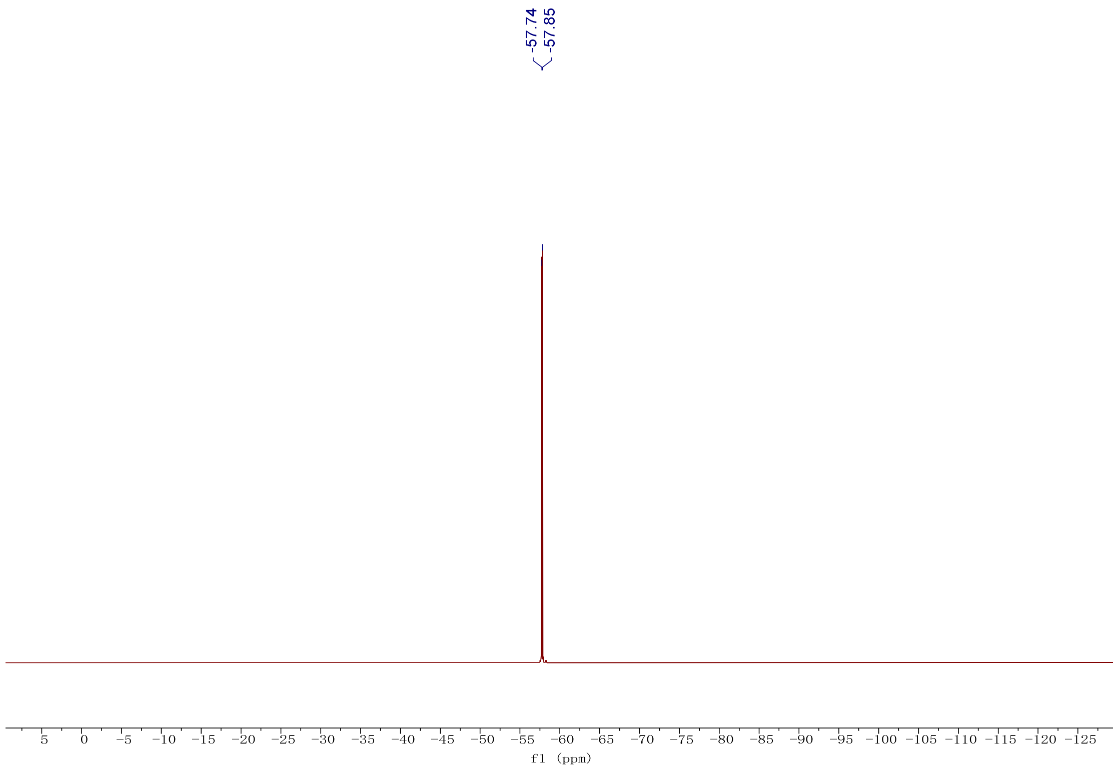

Supplementary Figure 86. ^{19}F NMR (376 MHz, $CDCl_3$) of **5g**

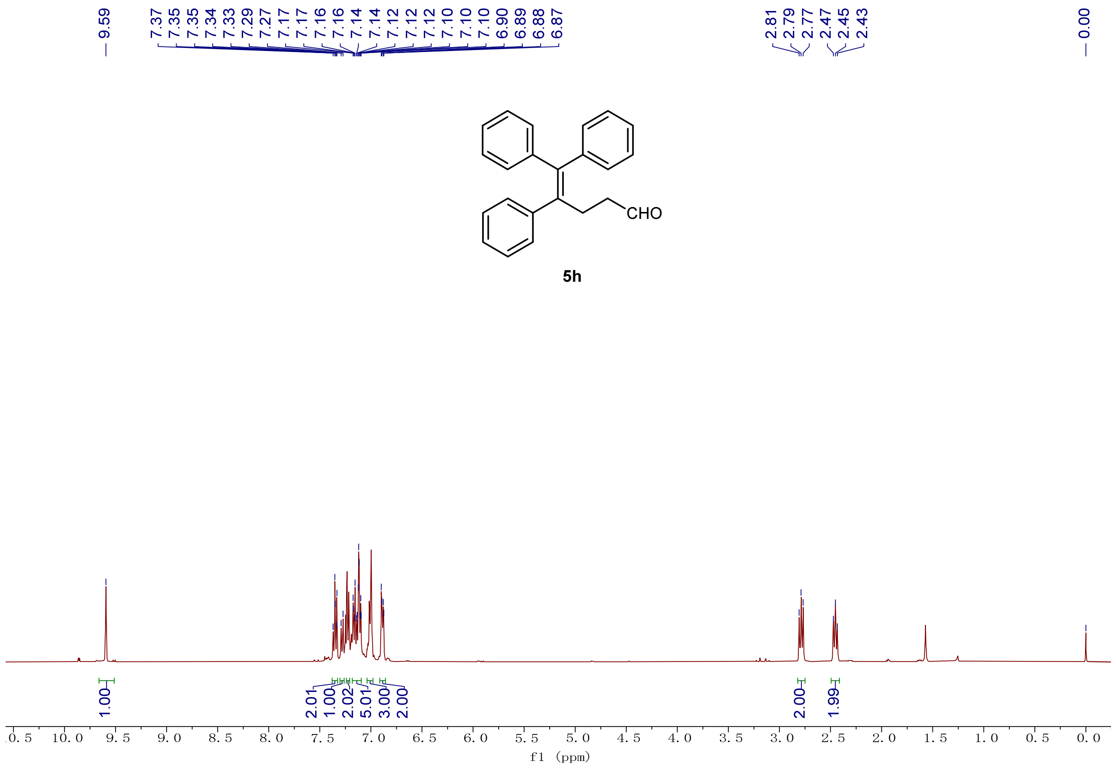

Supplementary Figure 87. ^{1}H NMR (400 MHz, $CDCl_3$) of **5h**

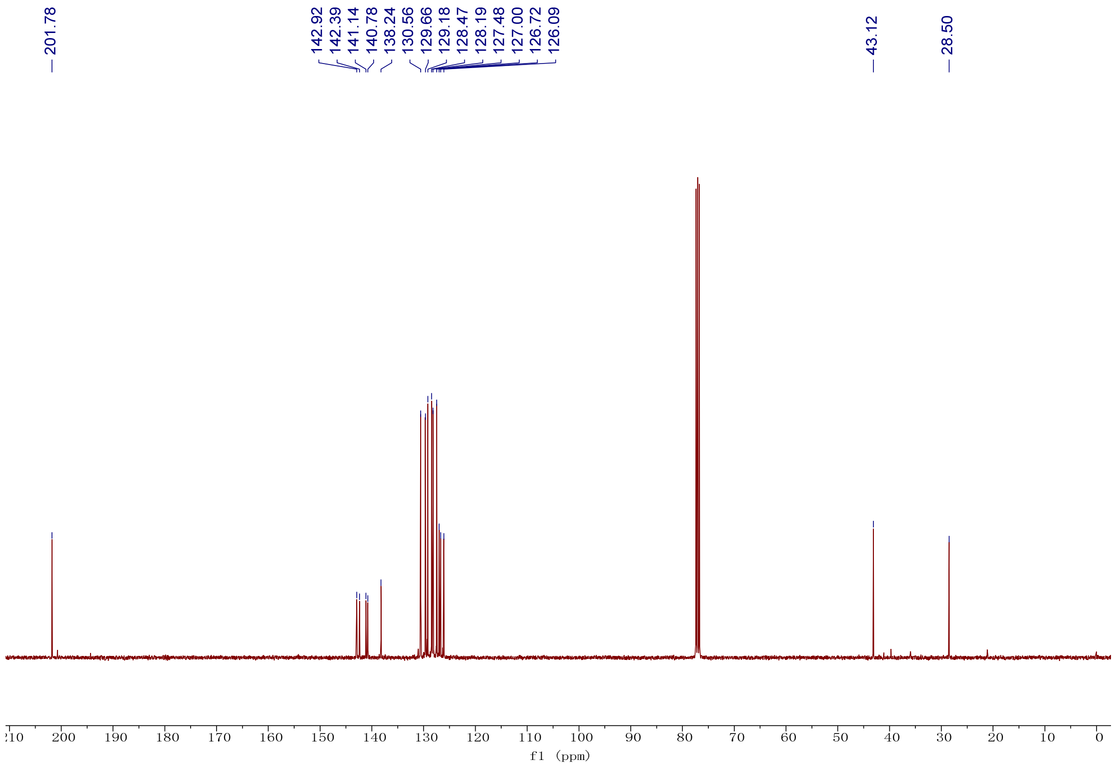

Supplementary Figure 88. ^{13}C NMR (101 MHz, $CDCl_3$) of **5h**

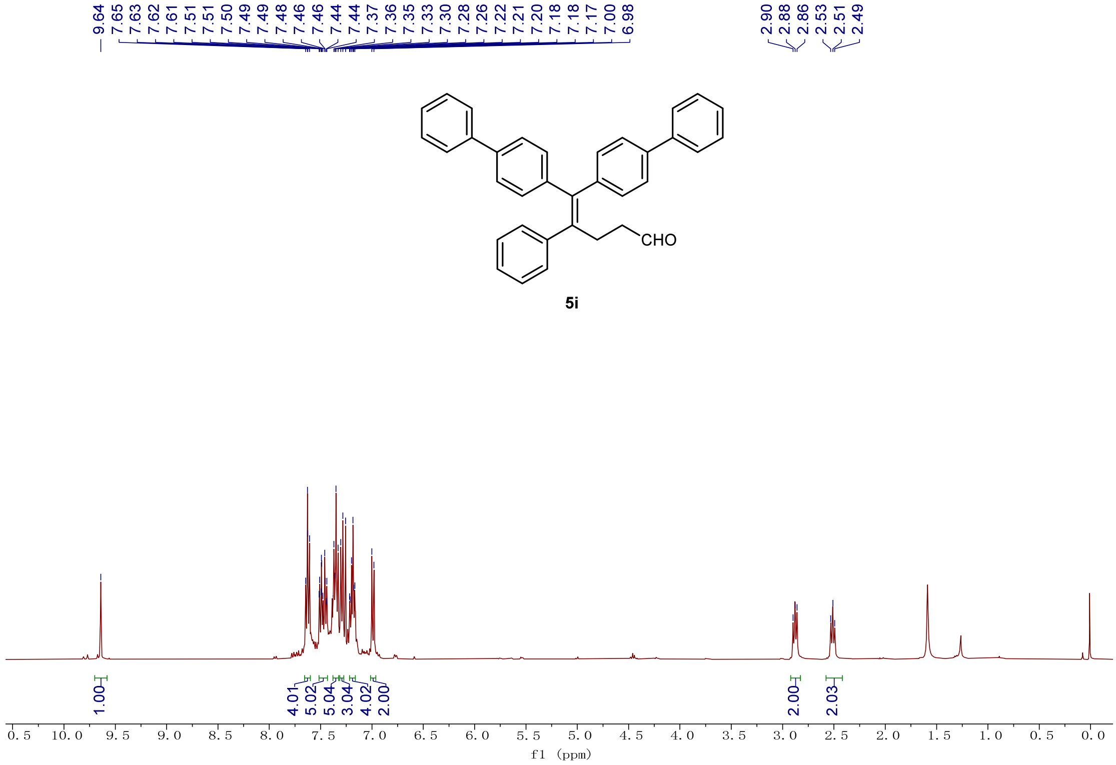

Supplementary Figure 89. ^{1}H NMR (400 MHz, CDCl$_3$) of **5i**

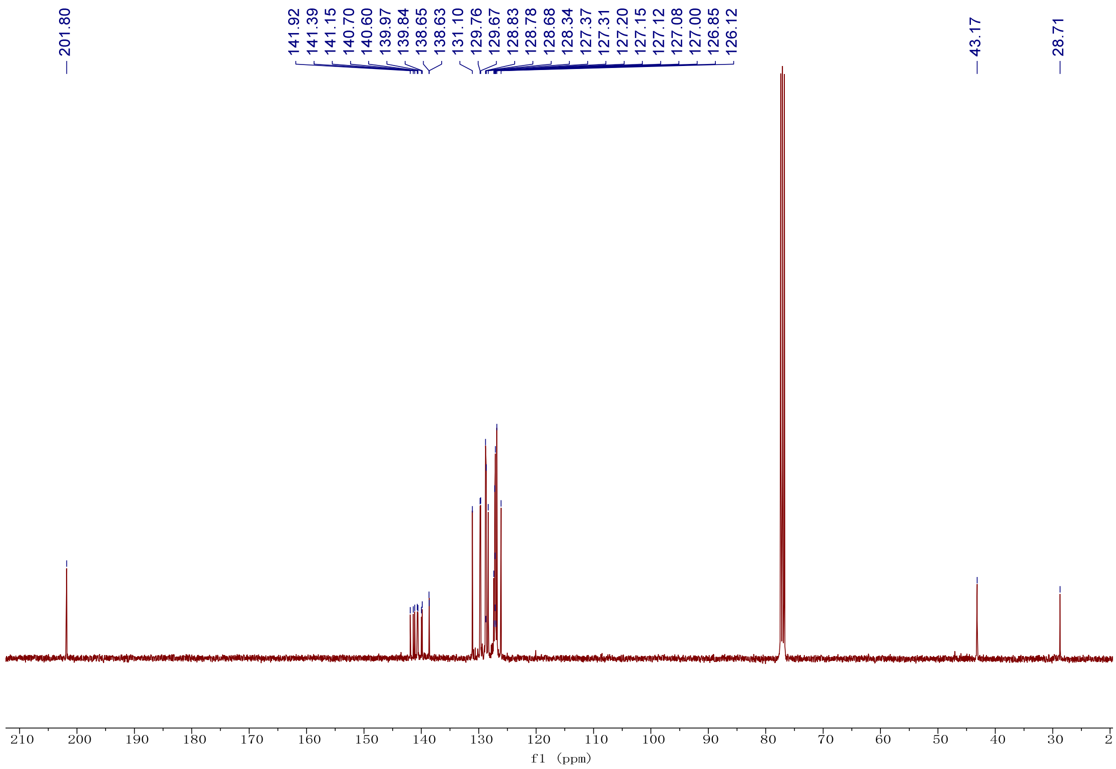

Supplementary Figure 90. ^{13}C NMR (101 MHz, CDCl$_3$) of **5i**

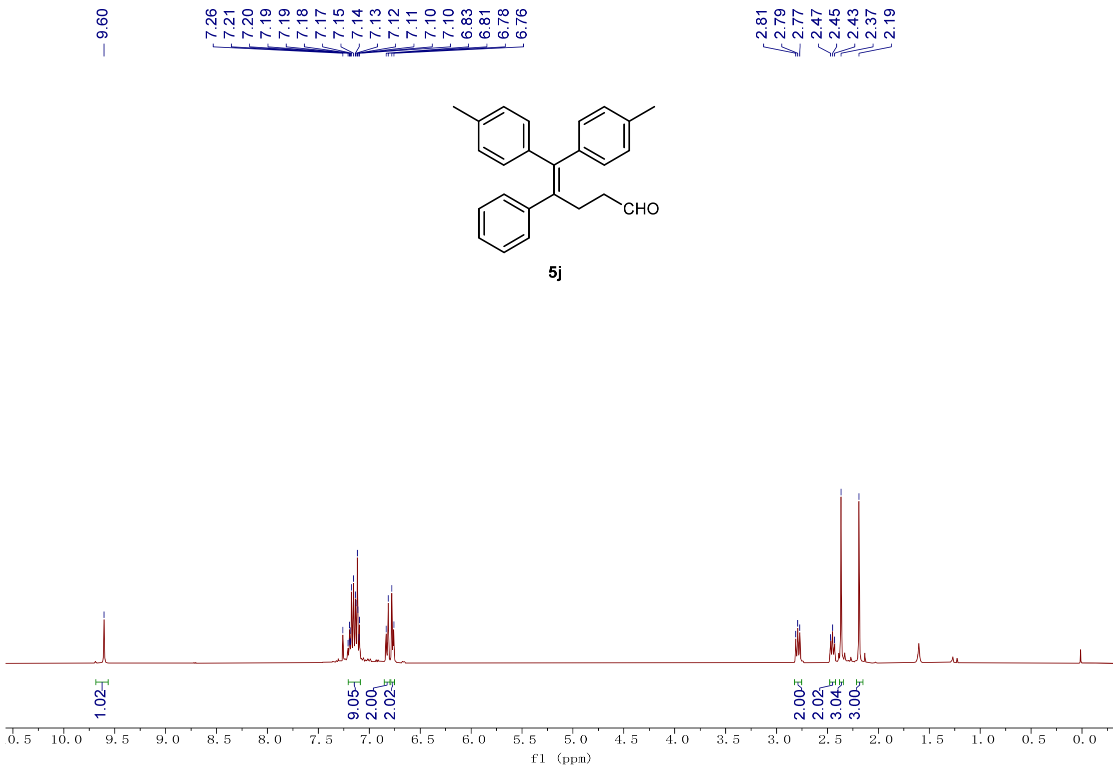

Supplementary Figure 91. ^{1}H NMR (400 MHz, $CDCl_3$) of **5j**

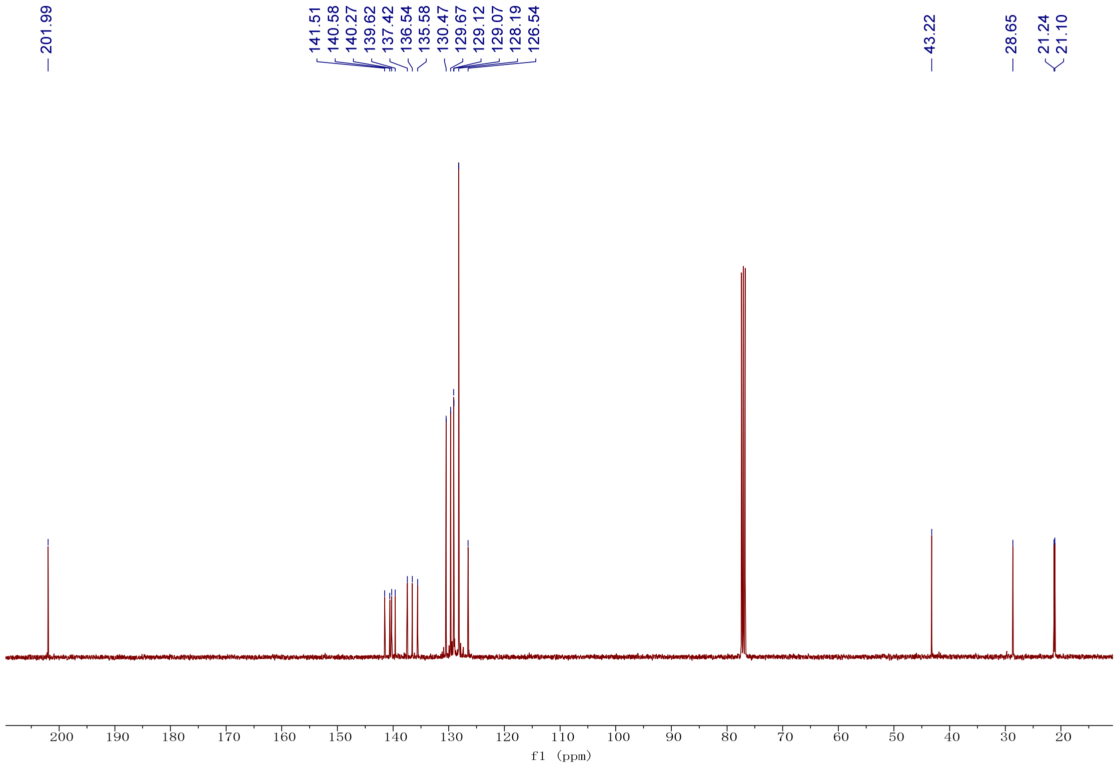

Supplementary Figure 92. ^{13}C NMR (101 MHz, $CDCl_3$) of **5j**

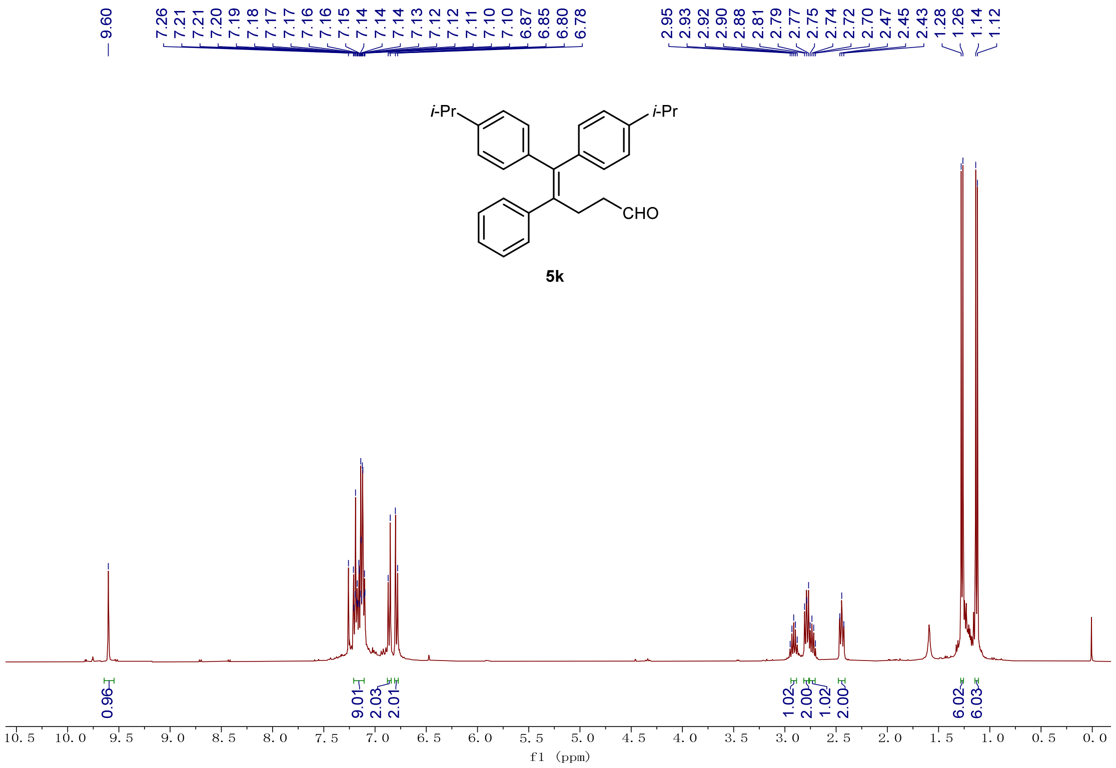

Supplementary Figure 93. ^{1}H NMR (400 MHz, $CDCl_3$) of **5k**

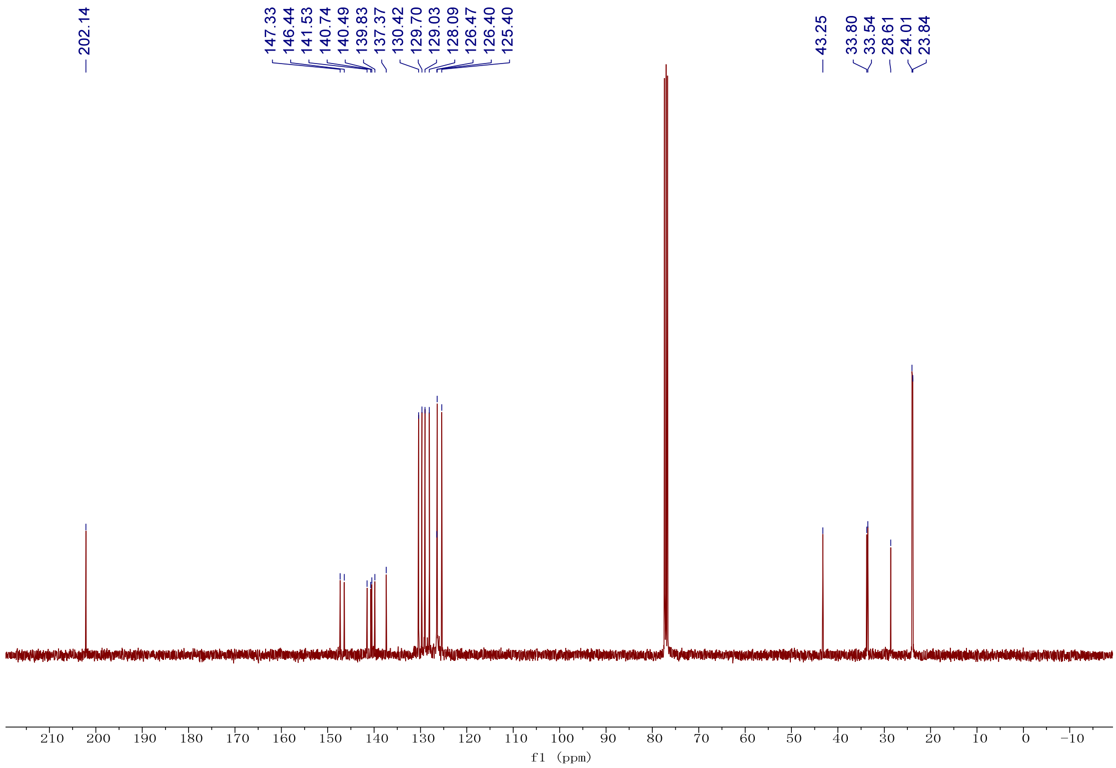

Supplementary Figure 94. ^{13}C NMR (101 MHz, $CDCl_3$) of **5k**

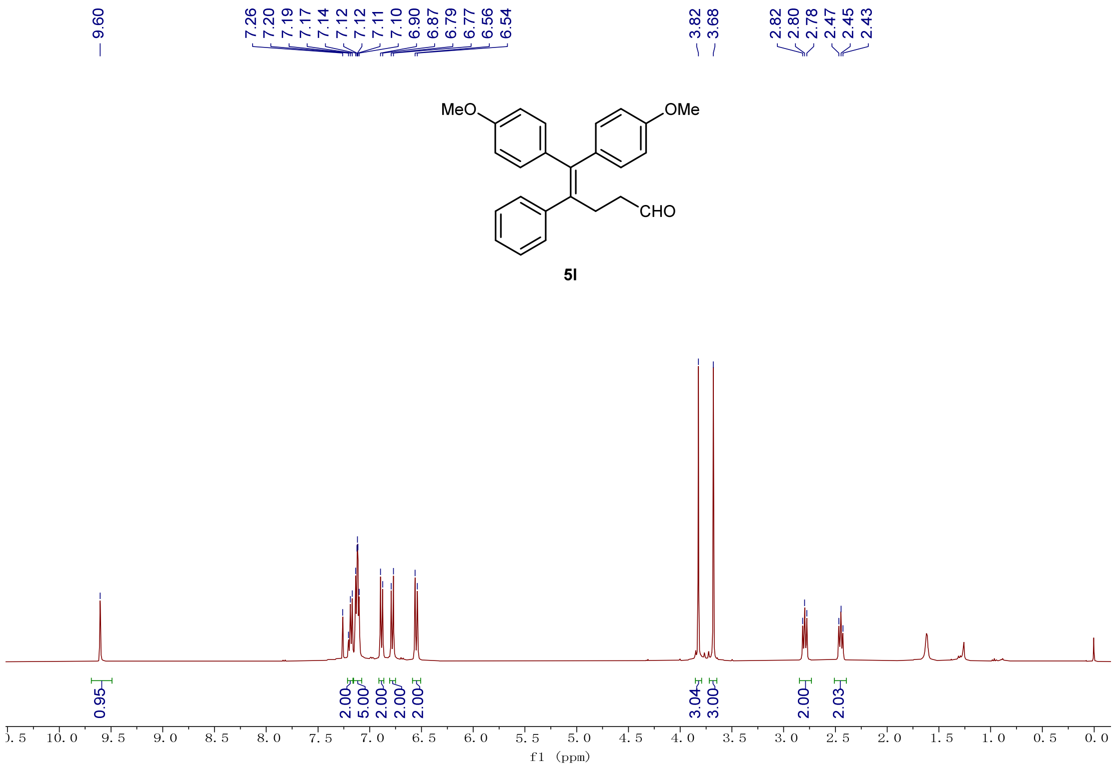

Supplementary Figure 95. ^{1}H NMR (400 MHz, $CDCl_3$) of **5l**

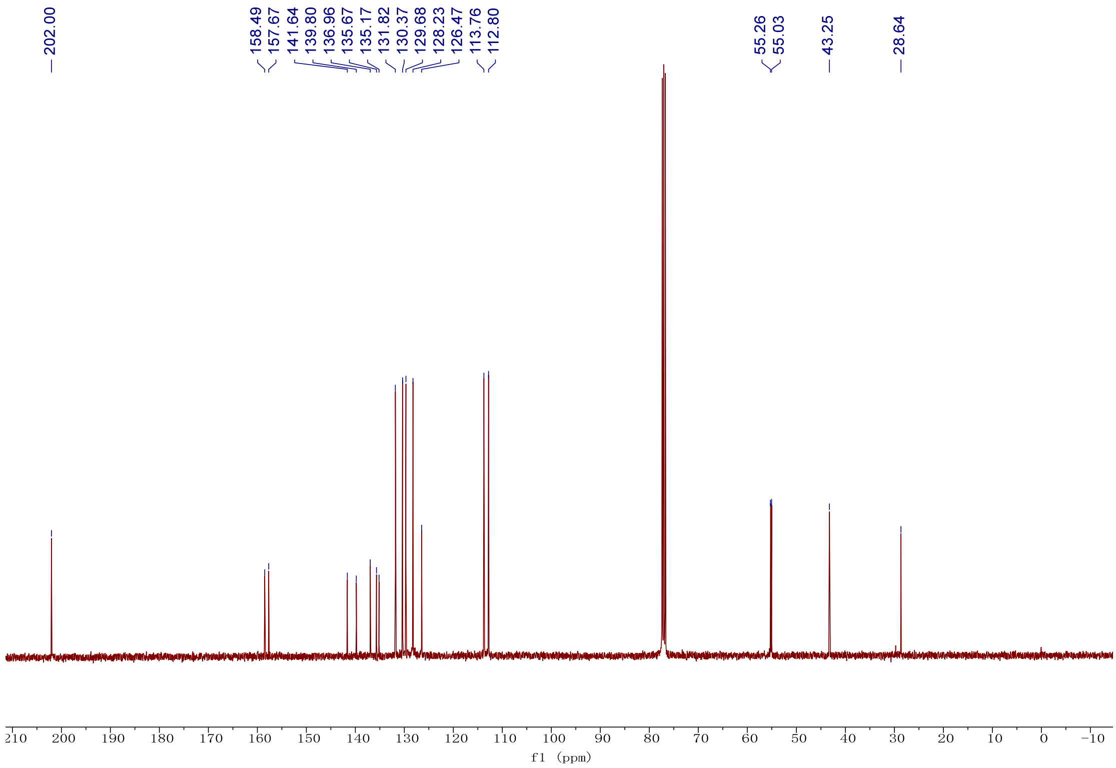

Supplementary Figure 96. ^{13}C NMR (101 MHz, $CDCl_3$) of **5l**

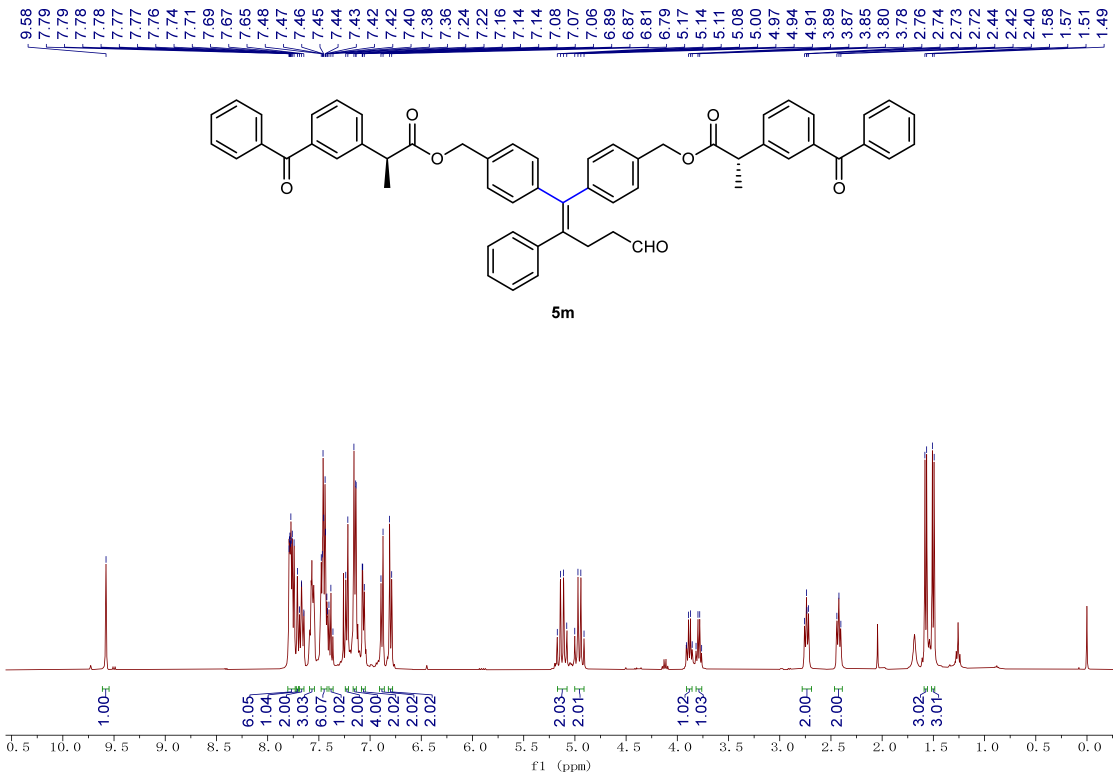

Supplementary Figure 97. ^{1}H NMR (400 MHz, $CDCl_3$) of **5m**

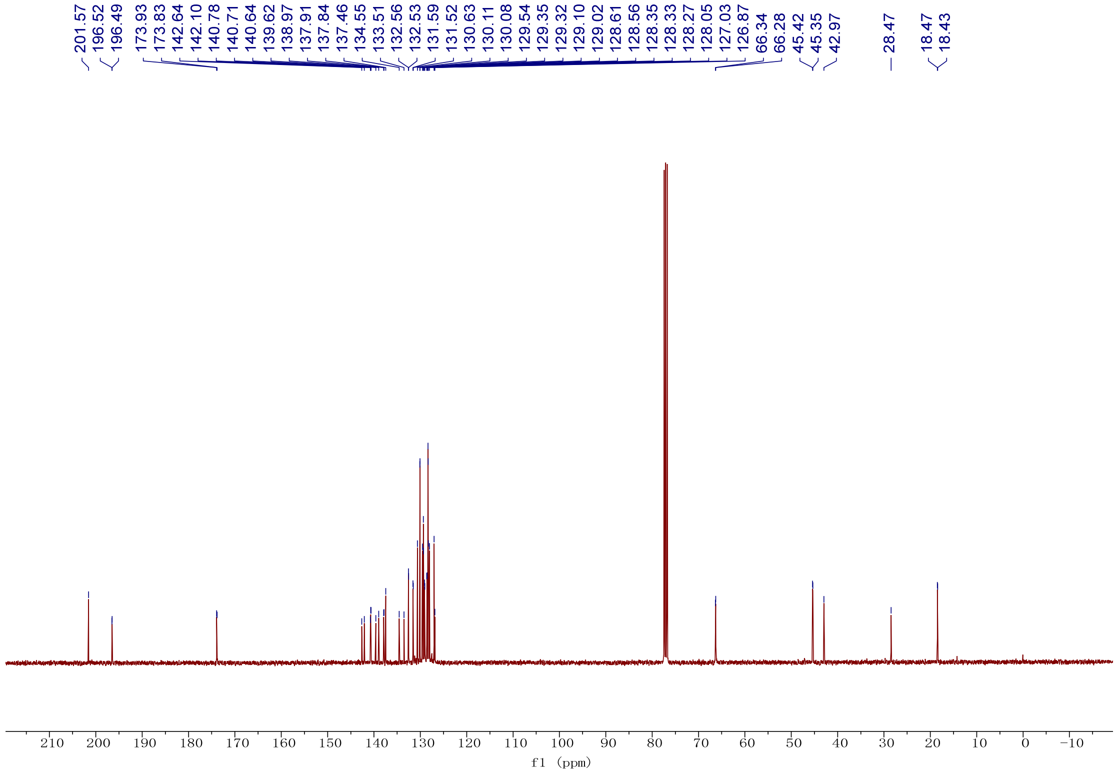

Supplementary Figure 98. ^{13}C NMR (101 MHz, $CDCl_3$) of **5m**

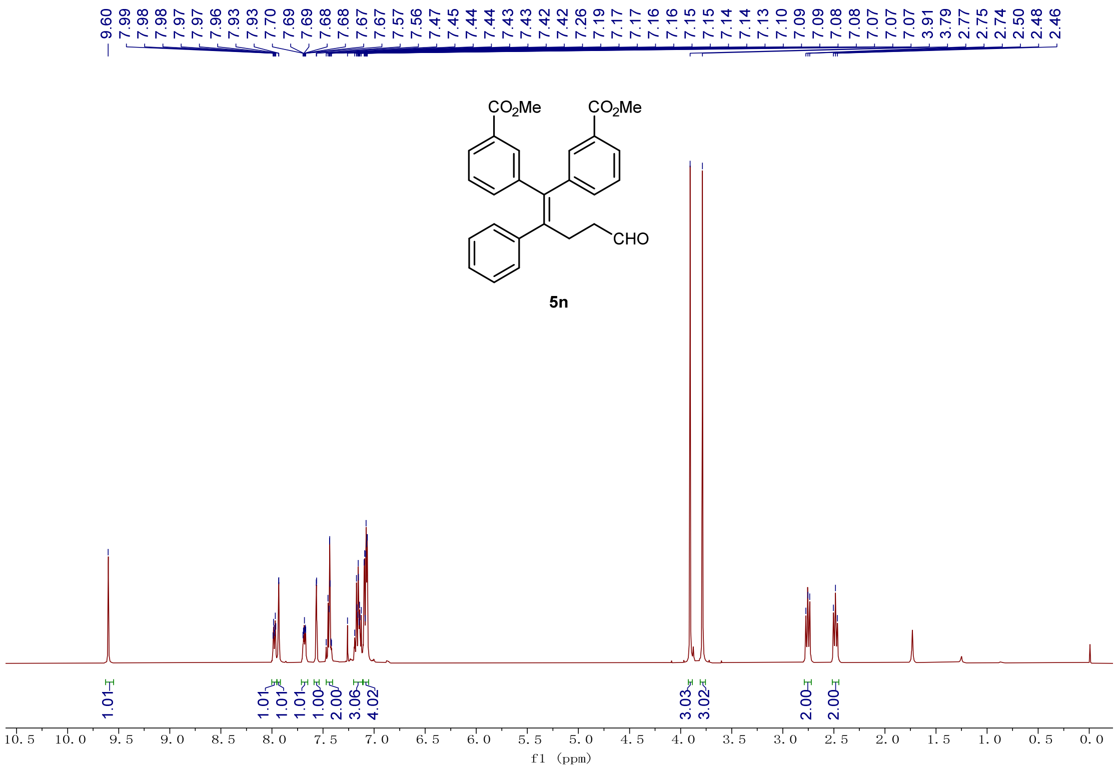

Supplementary Figure 99. ^{1}H NMR (400 MHz, $CDCl_3$) of **5n**

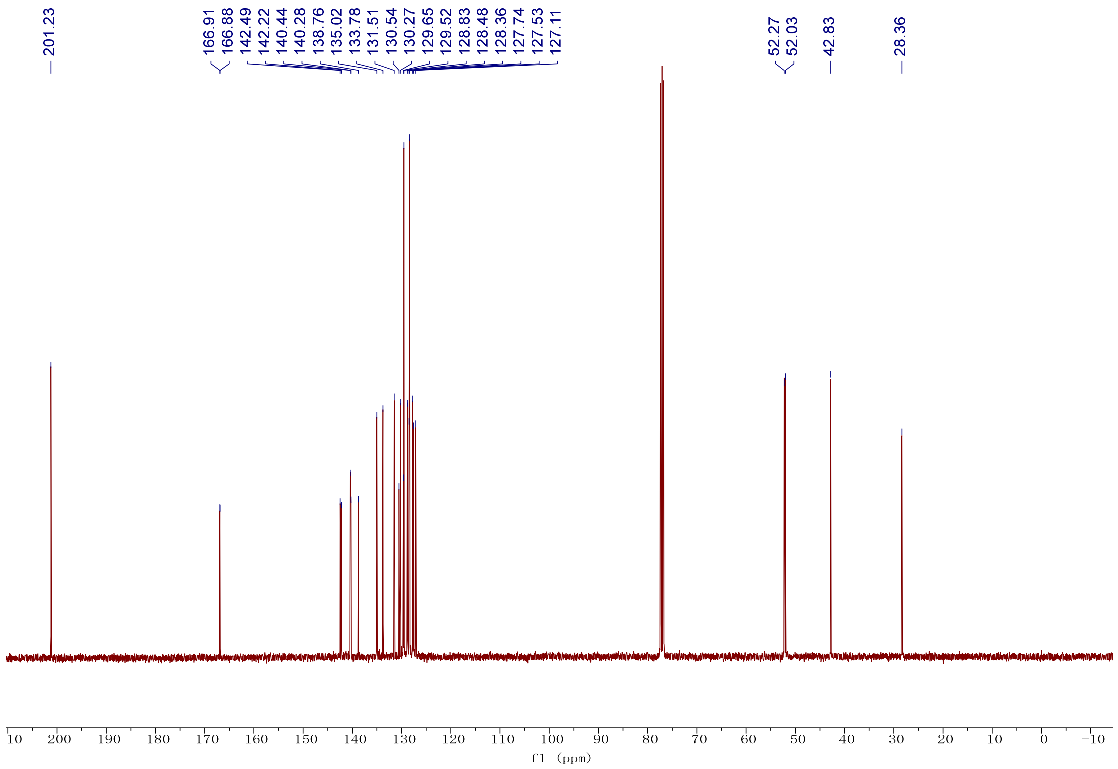

Supplementary Figure 100. ^{13}C NMR (101 MHz, $CDCl_3$) of **5n**

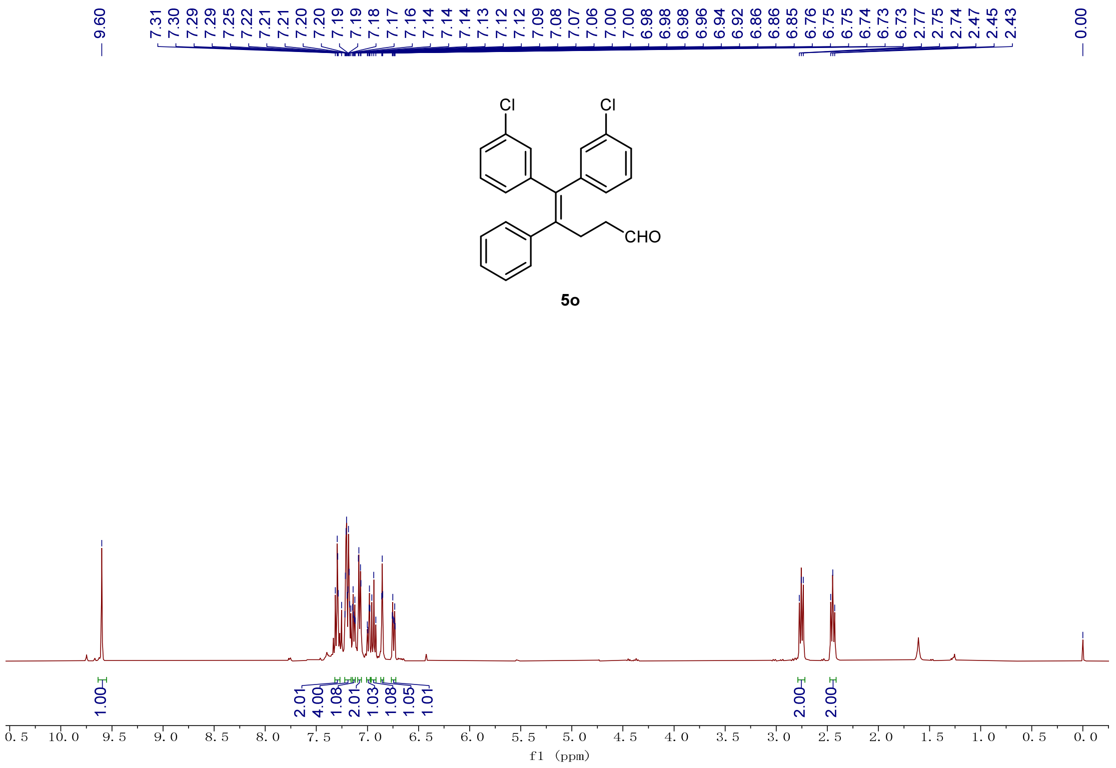

Supplementary Figure 101. ^{1}H NMR (400 MHz, $CDCl_3$) of **5o**

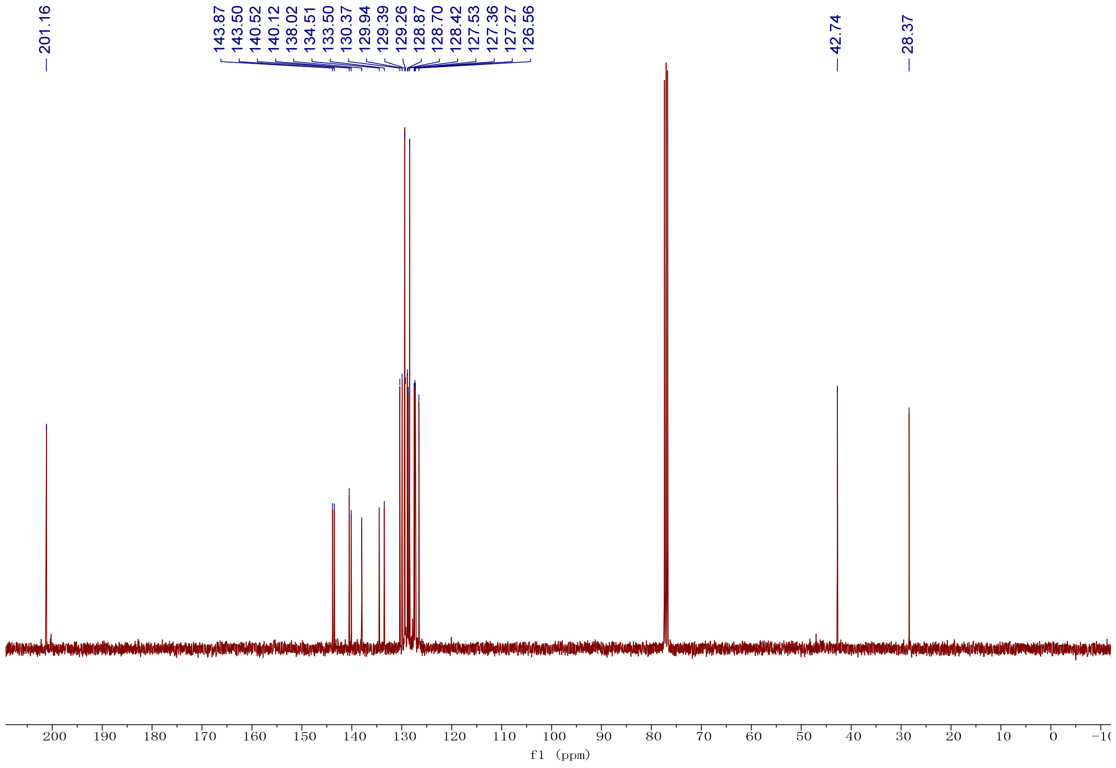

Supplementary Figure 102. ^{13}C NMR (101 MHz, $CDCl_3$) of **5o**

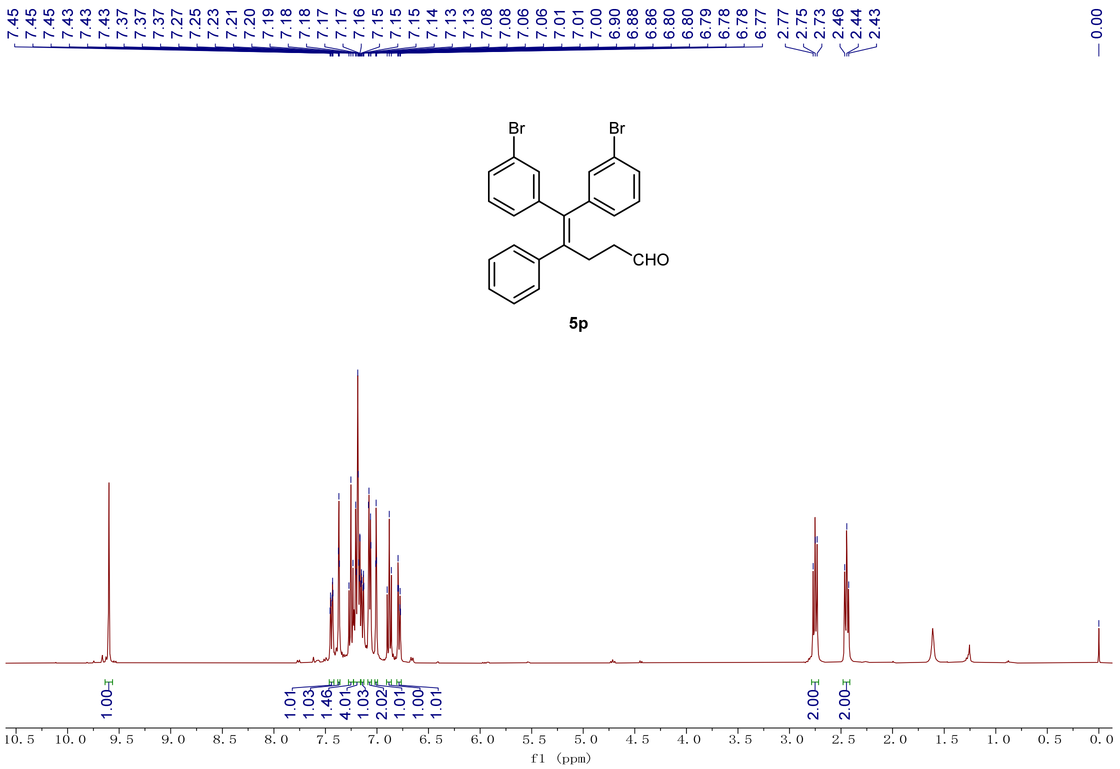

Supplementary Figure 103. ^{1}H NMR (400 MHz, $CDCl_3$) of **5p**

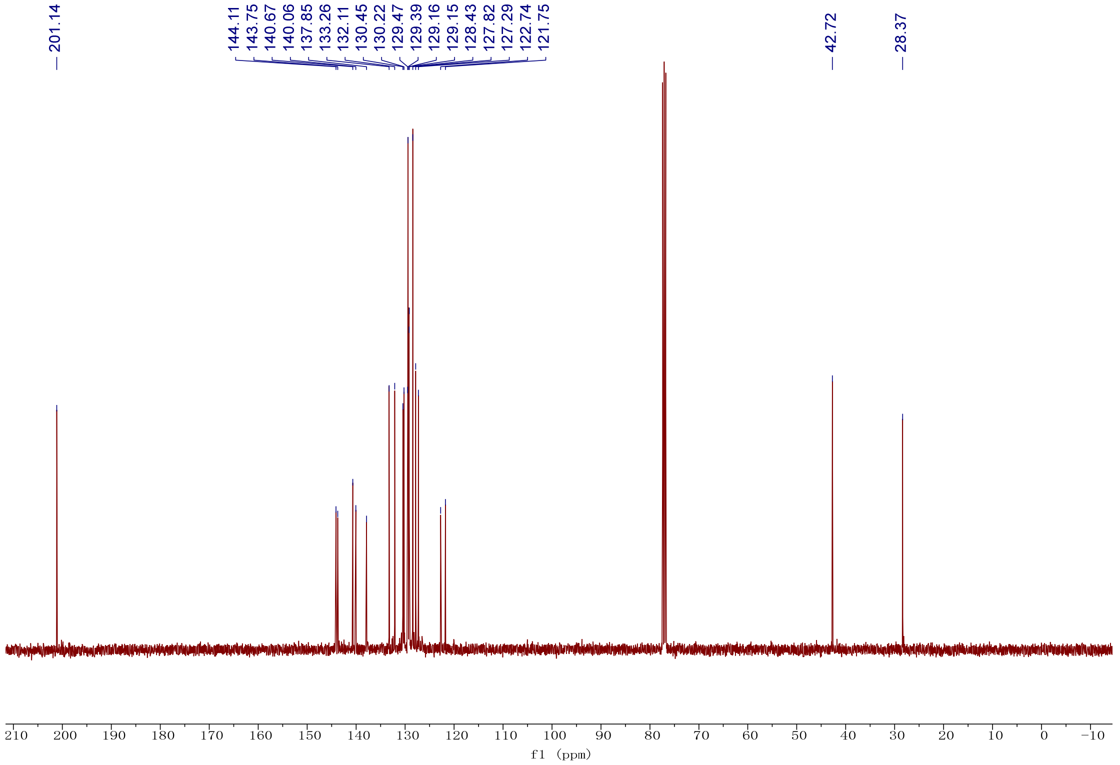

Supplementary Figure 104. ^{13}C NMR (101 MHz, $CDCl_3$) of **5p**

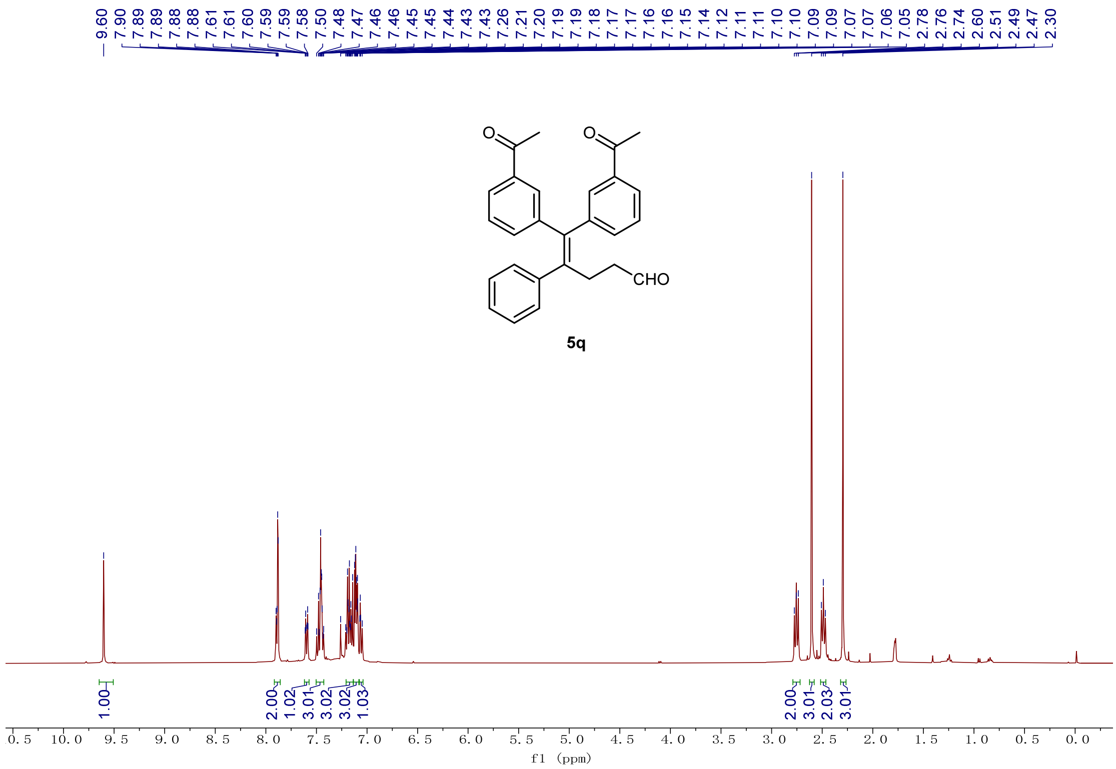

Supplementary Figure 105. ^{1}H NMR (400 MHz, $CDCl_3$) of **5q**

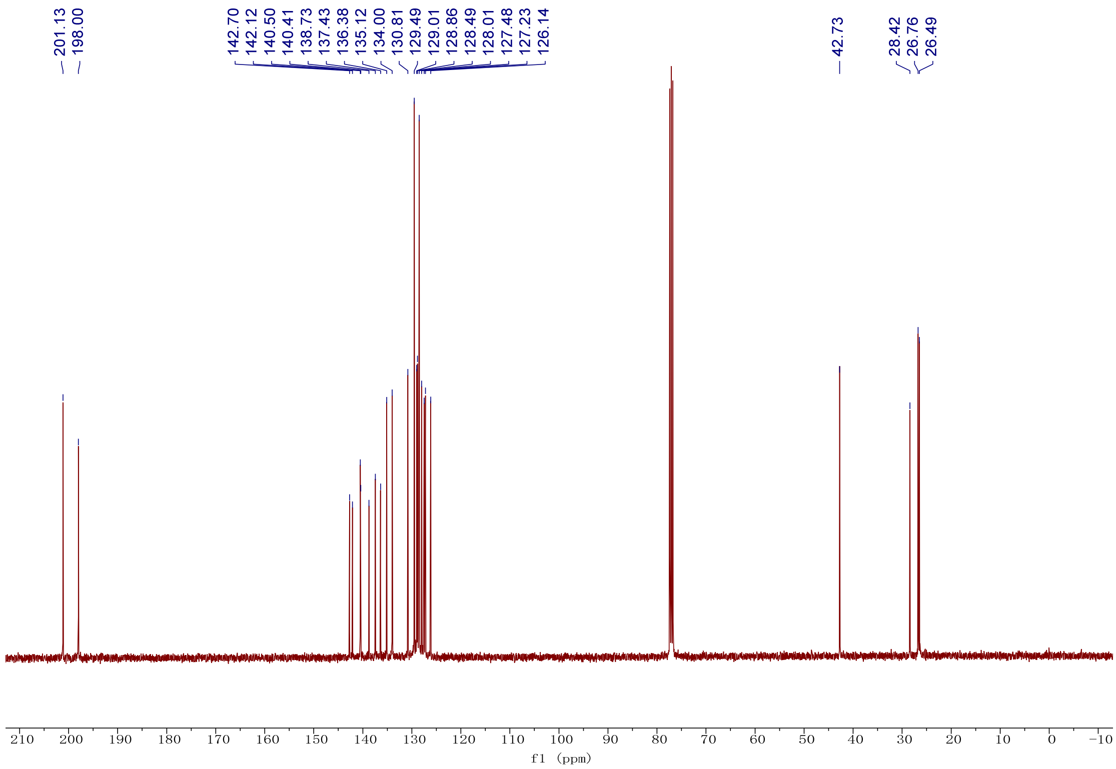

Supplementary Figure 106. ^{13}C NMR (101 MHz, $CDCl_3$) of **5q**

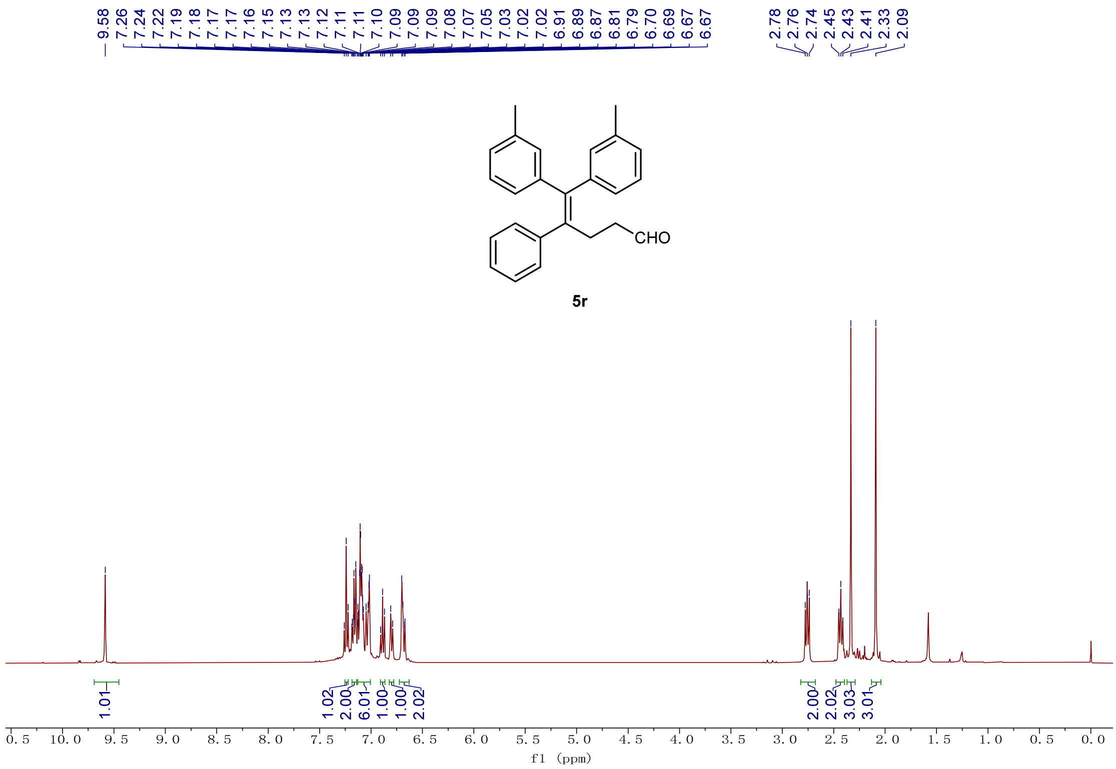

Supplementary Figure 107. ^{1}H NMR (400 MHz, $CDCl_3$) of **5r**

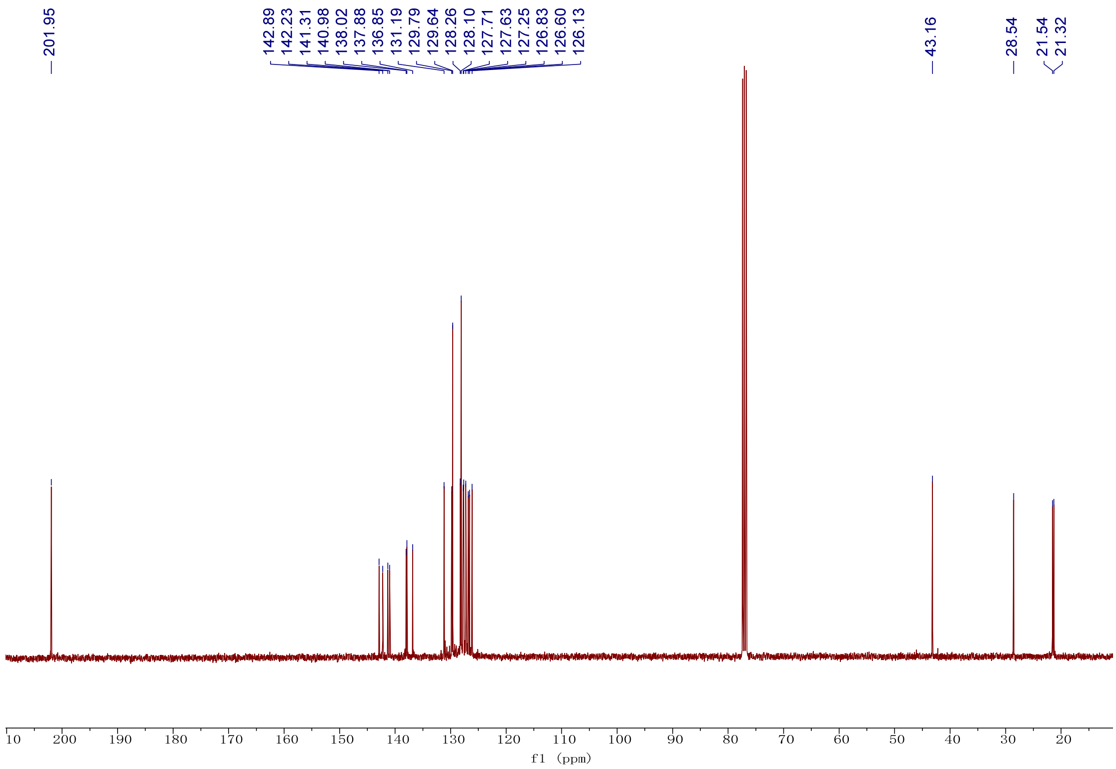

Supplementary Figure 108. ^{13}C NMR (101 MHz, $CDCl_3$) of **5r**

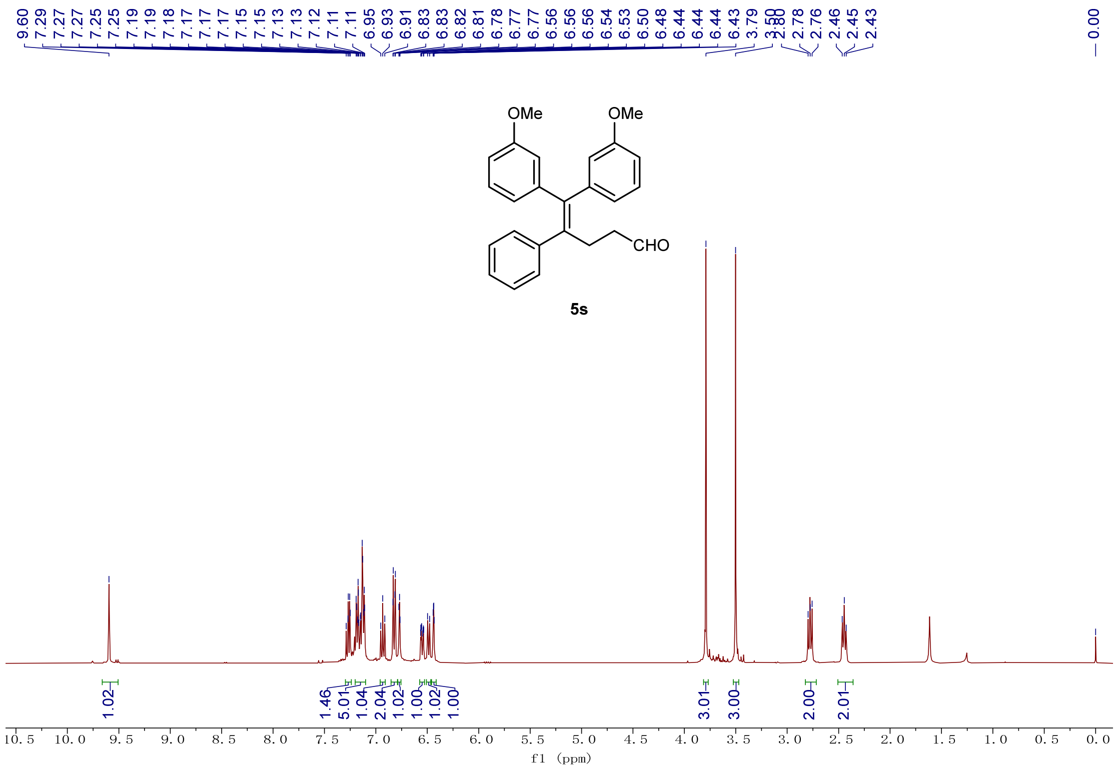

Supplementary Figure 109. ^{1}H NMR (400 MHz, $CDCl_3$) of **5s**

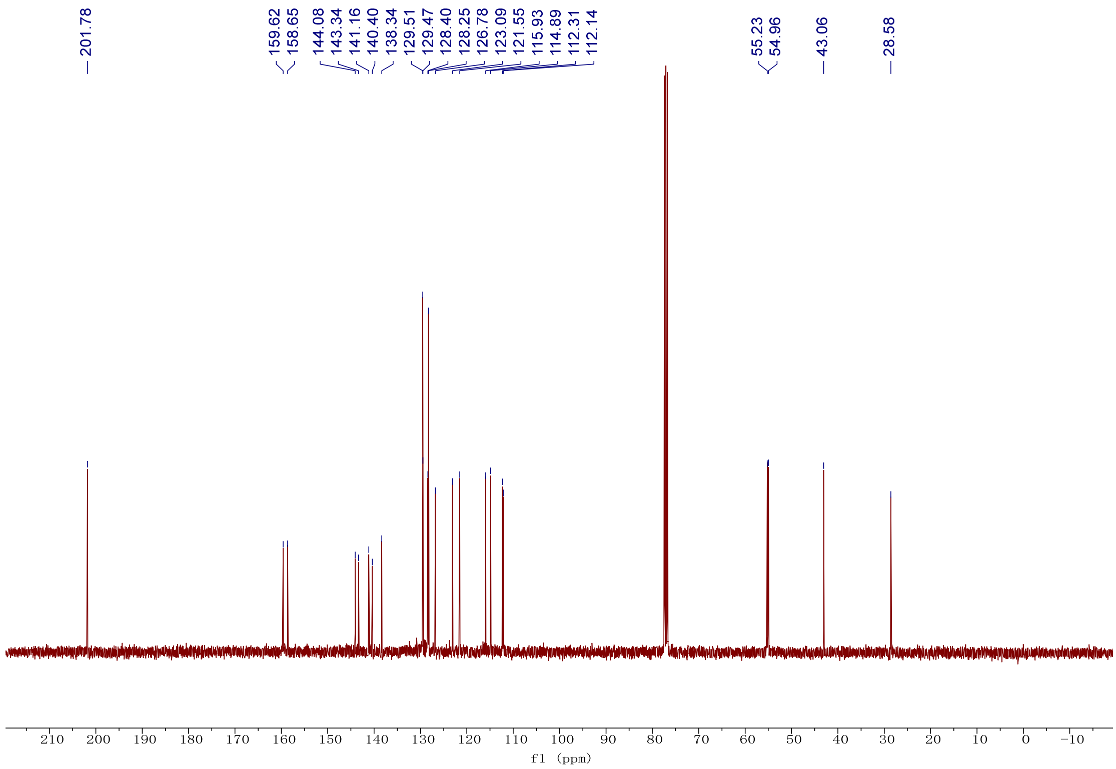

Supplementary Figure 110. ^{13}C NMR (101 MHz, $CDCl_3$) of **5s**

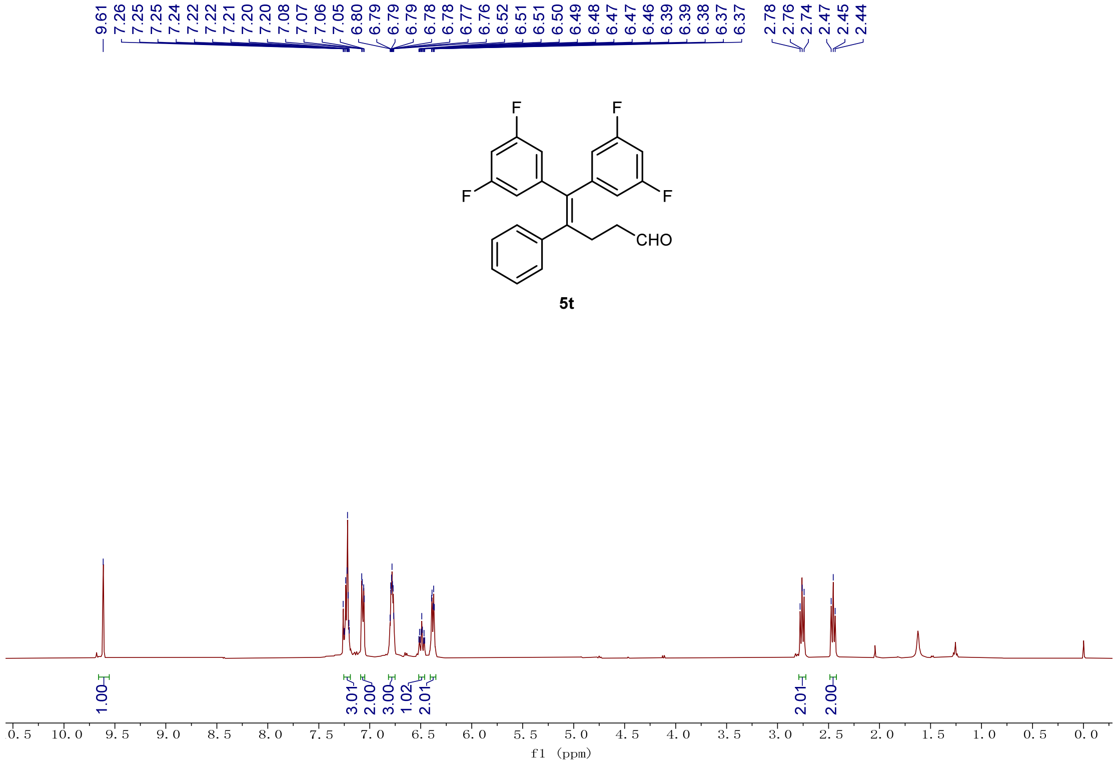

Supplementary Figure 111. ^{1}H NMR (400 MHz, $CDCl_3$) of **5t**

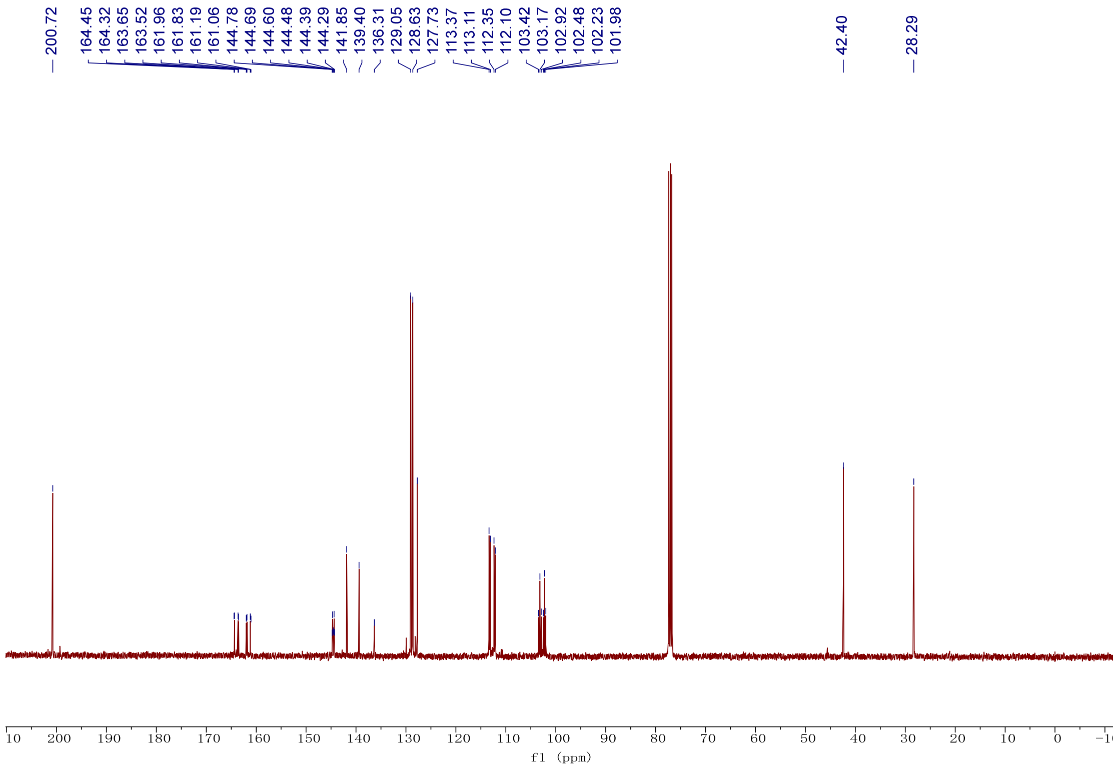

Supplementary Figure 112. ^{13}C NMR (101 MHz, $CDCl_3$) of **5t**

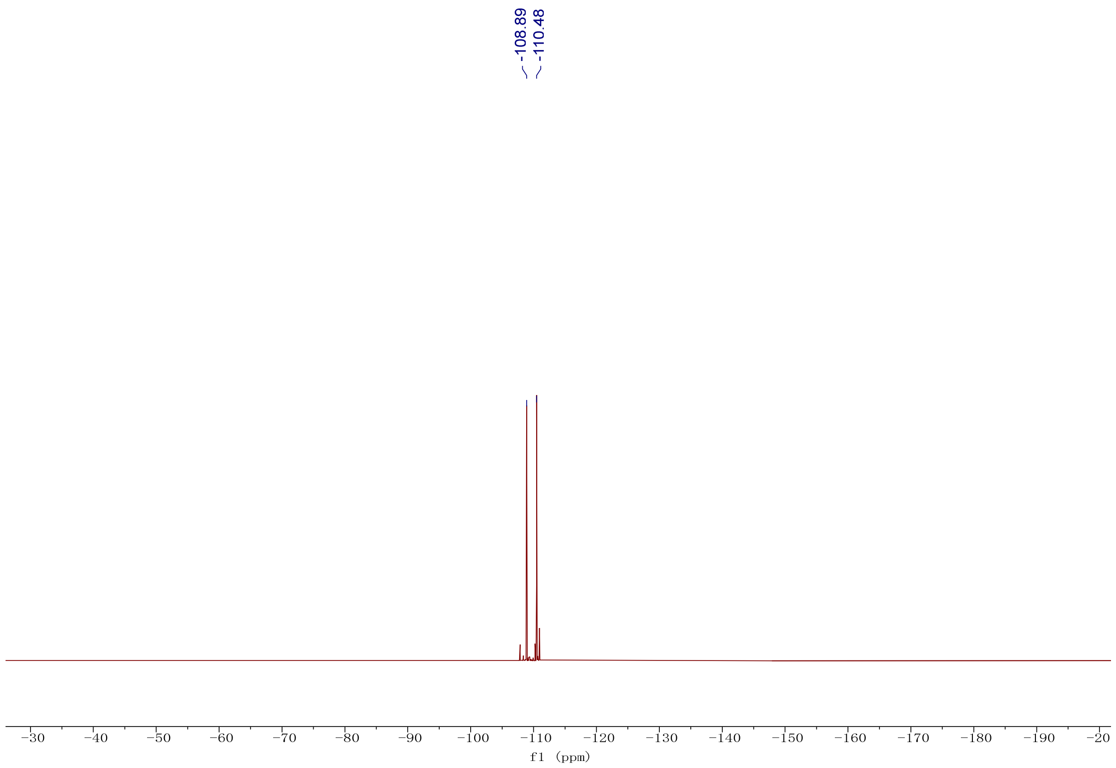

Supplementary Figure 113. ^{19}F NMR (376 MHz, $CDCl_3$) of **5t**

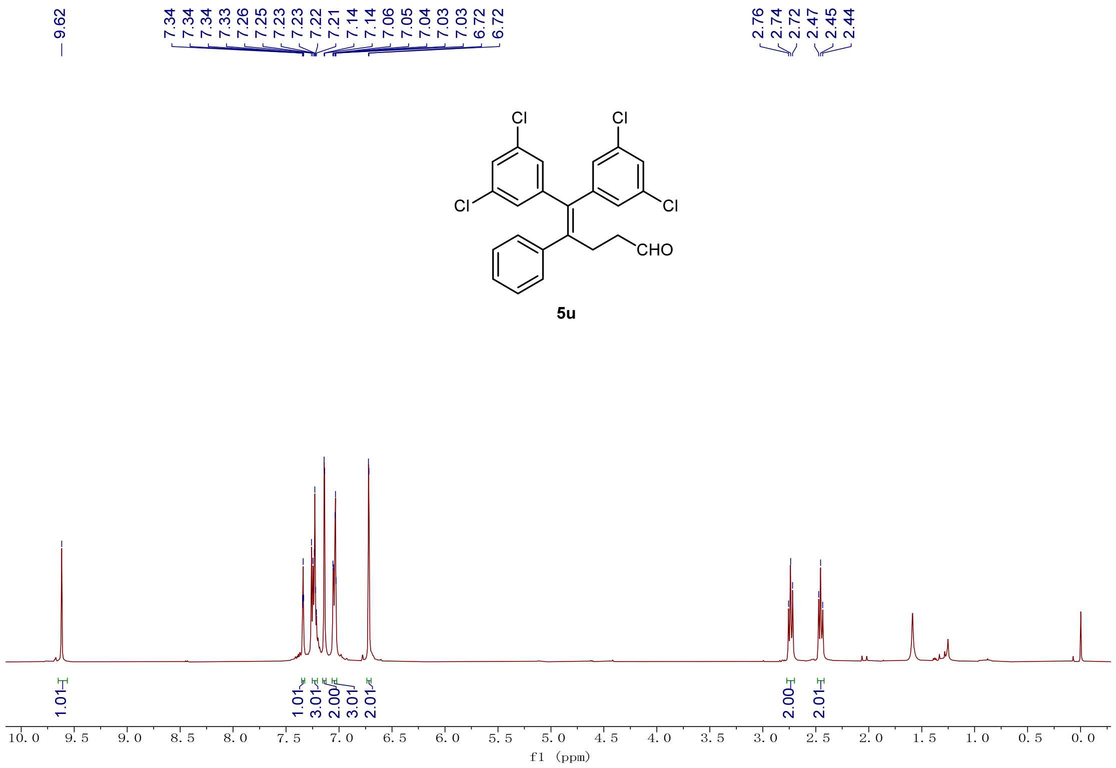

Supplementary Figure 114. ^{1}H NMR (400 MHz, $CDCl_3$) of **5u**

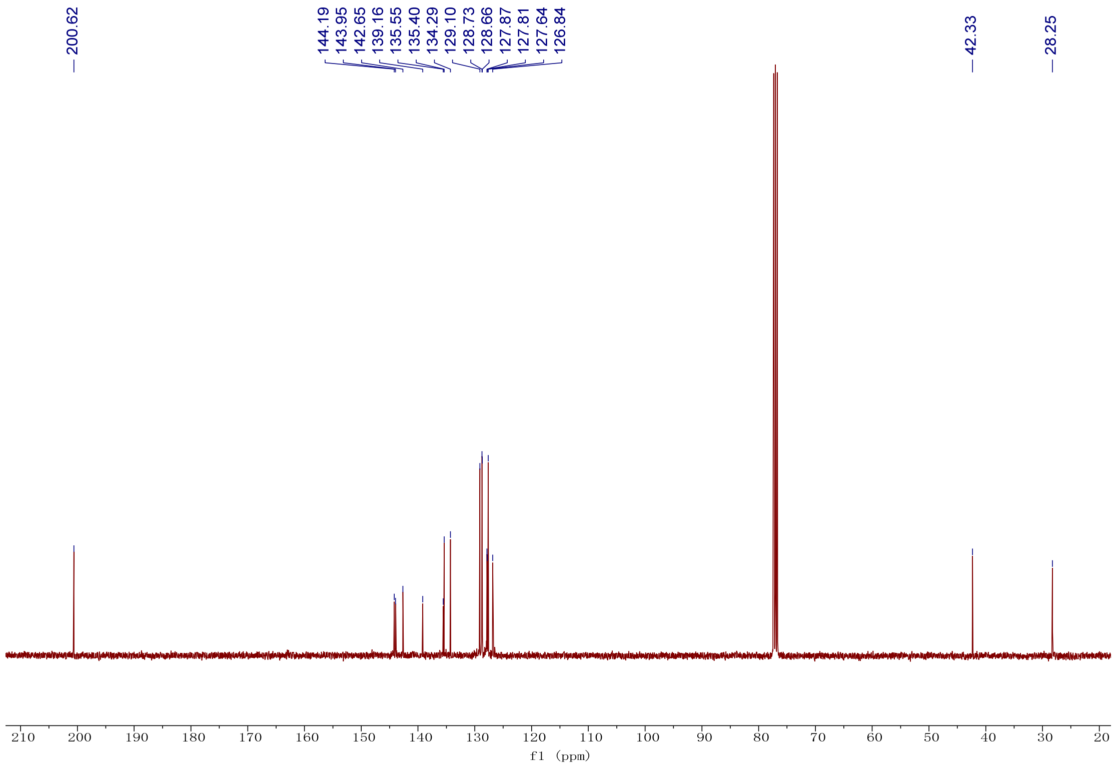

Supplementary Figure 115. ^{13}C NMR (101 MHz, $CDCl_3$) of **5u**

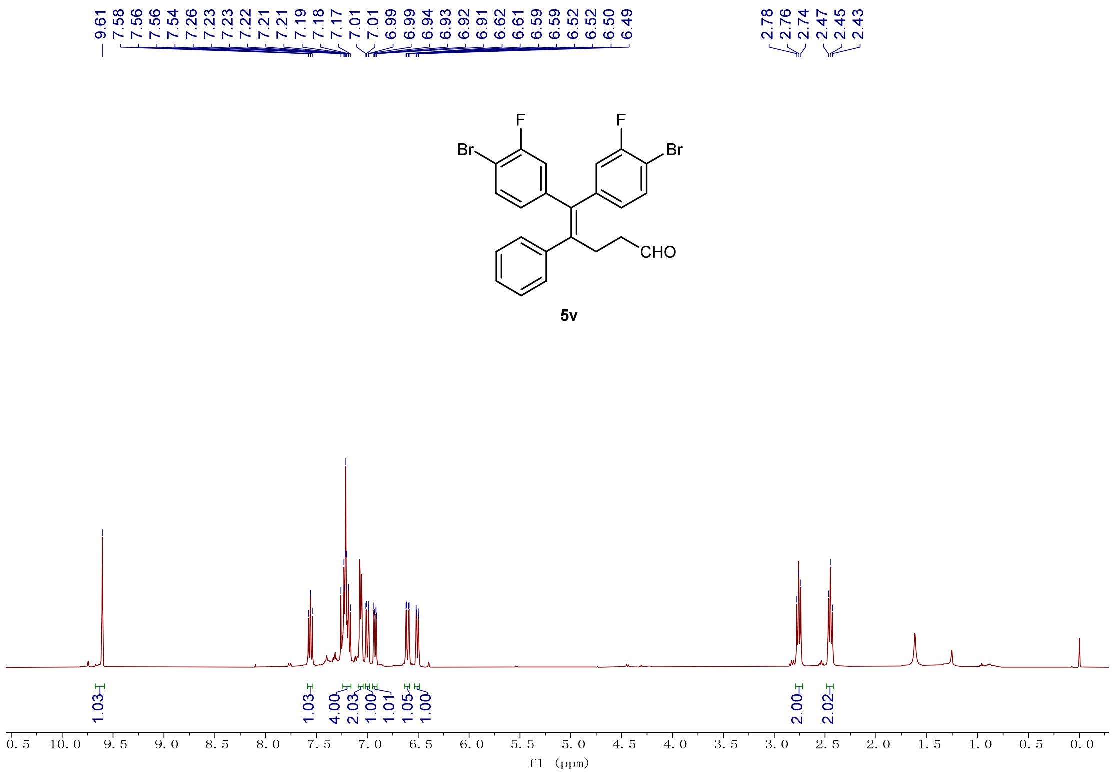

Supplementary Figure 116. ^{1}H NMR (400 MHz, $CDCl_3$) of **5v**

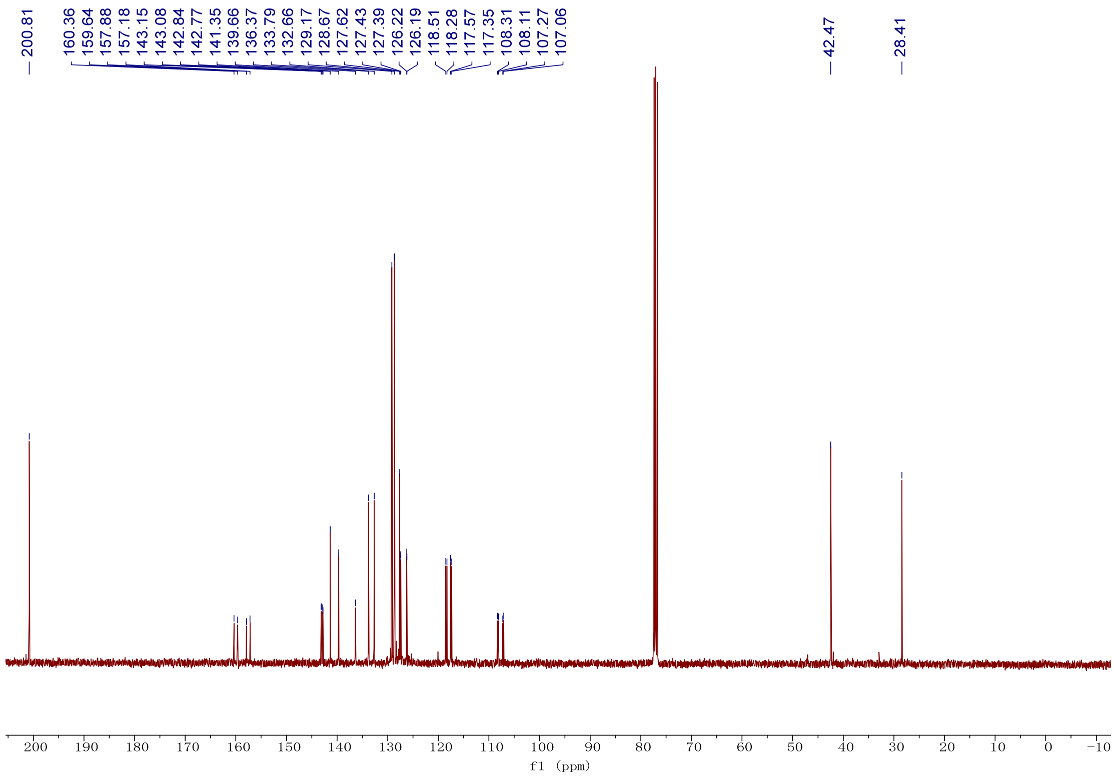

Supplementary Figure 117. ^{13}C NMR (101 MHz, $CDCl_3$) of **5v**

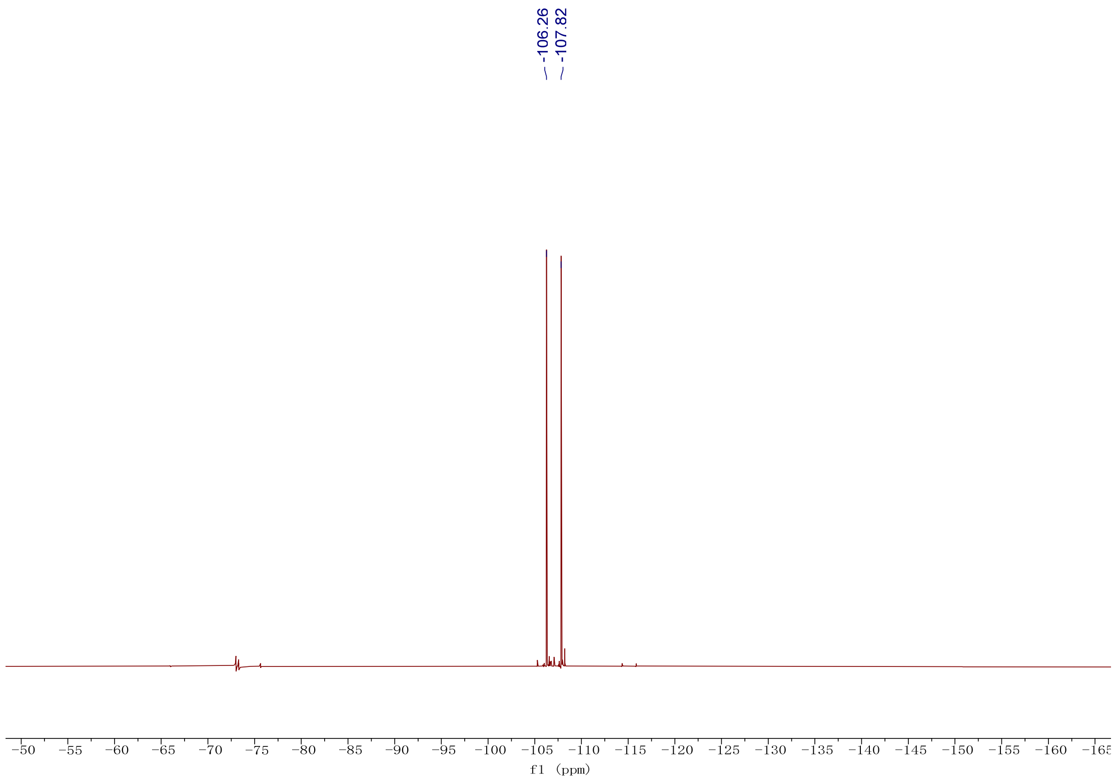

Supplementary Figure 118. ^{19}F NMR (376 MHz, $CDCl_3$) of **5v**

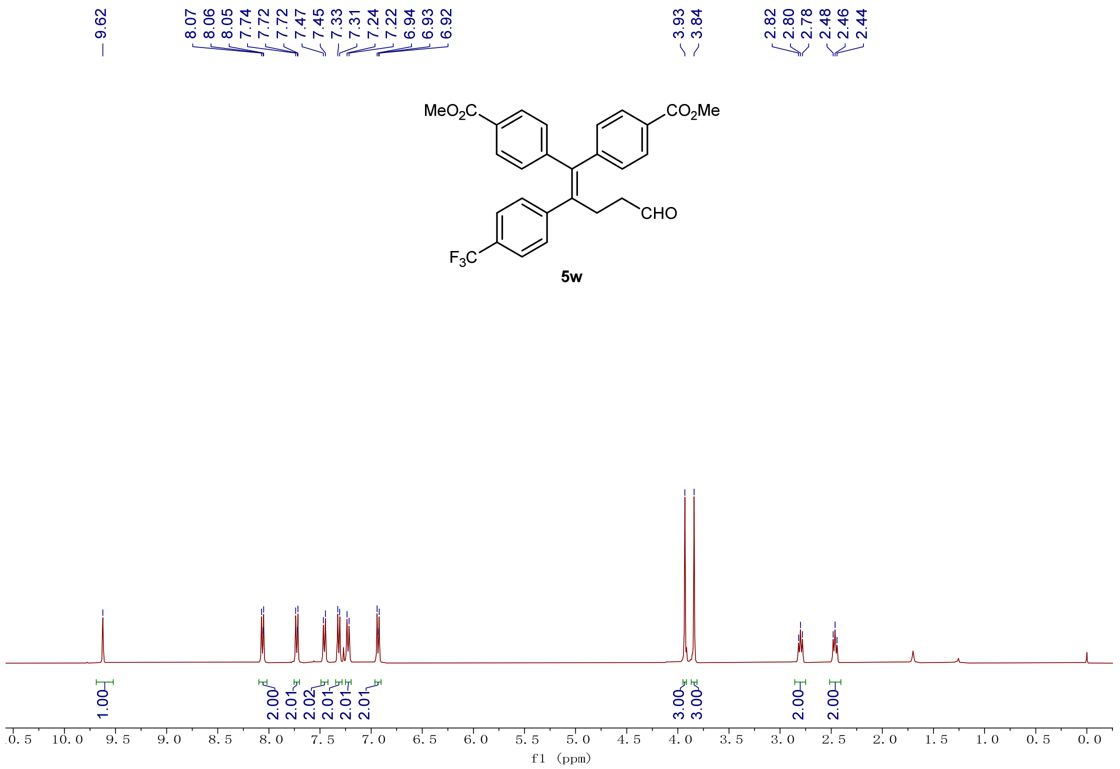

Supplementary Figure 119. ^{1}H NMR (400 MHz, $CDCl_3$) of **5w**

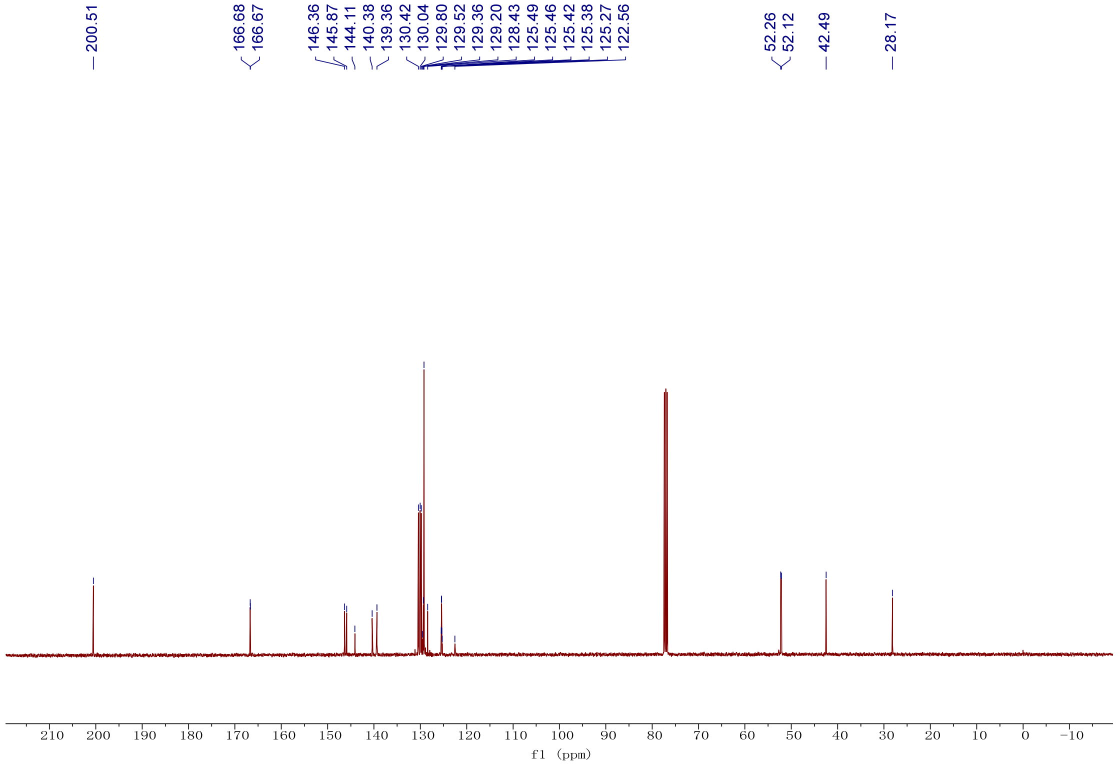

Supplementary Figure 120. ^{13}C NMR (101 MHz, $CDCl_3$) of **5w**

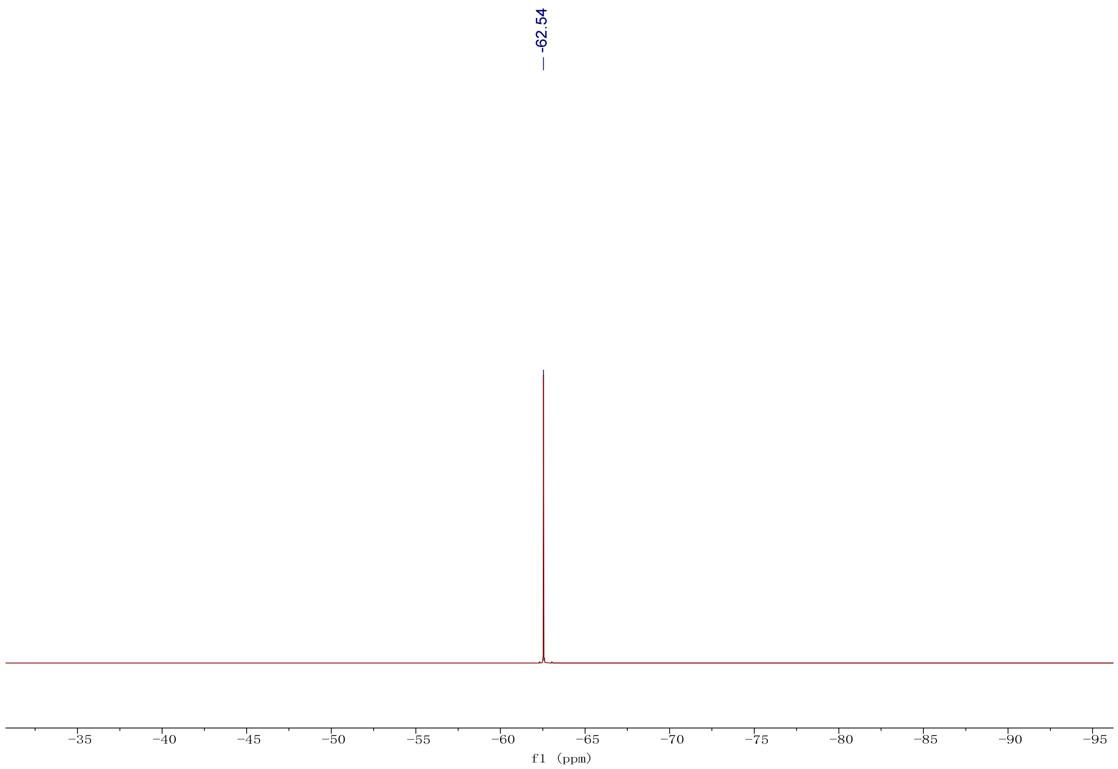

Supplementary Figure 121. ^{19}F NMR (376 MHz, $CDCl_3$) of **5w**

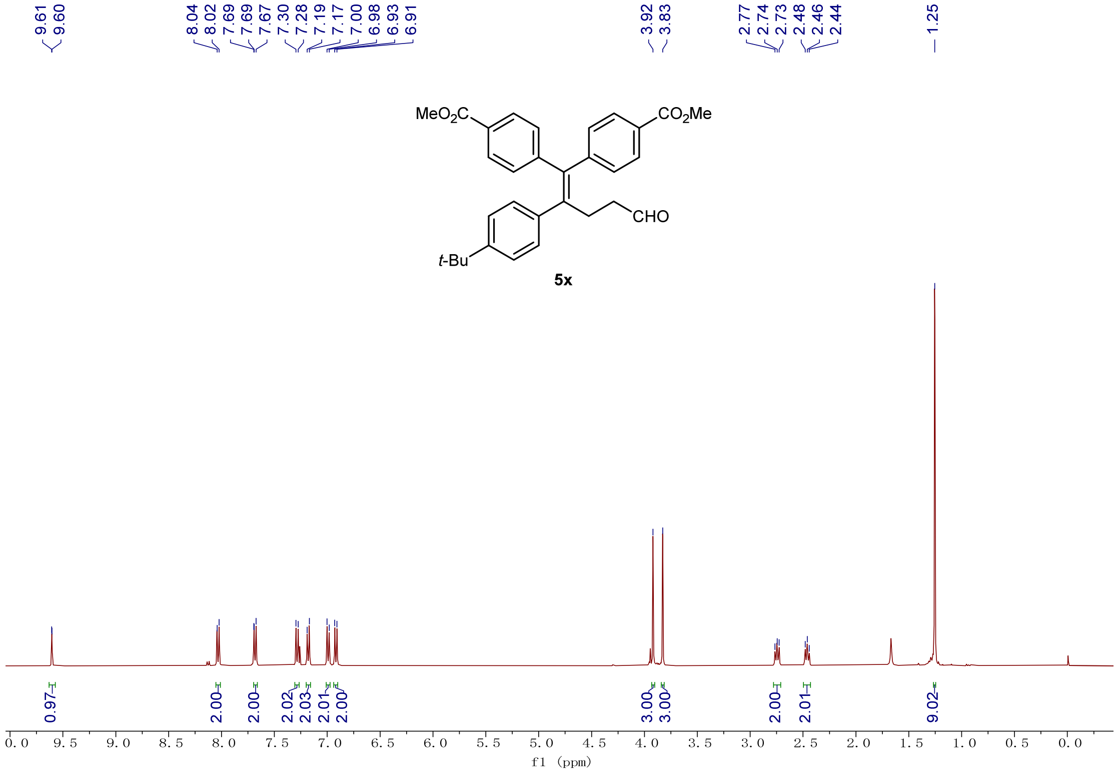

Supplementary Figure 122. ^{1}H NMR (400 MHz, $CDCl_3$) of **5x**

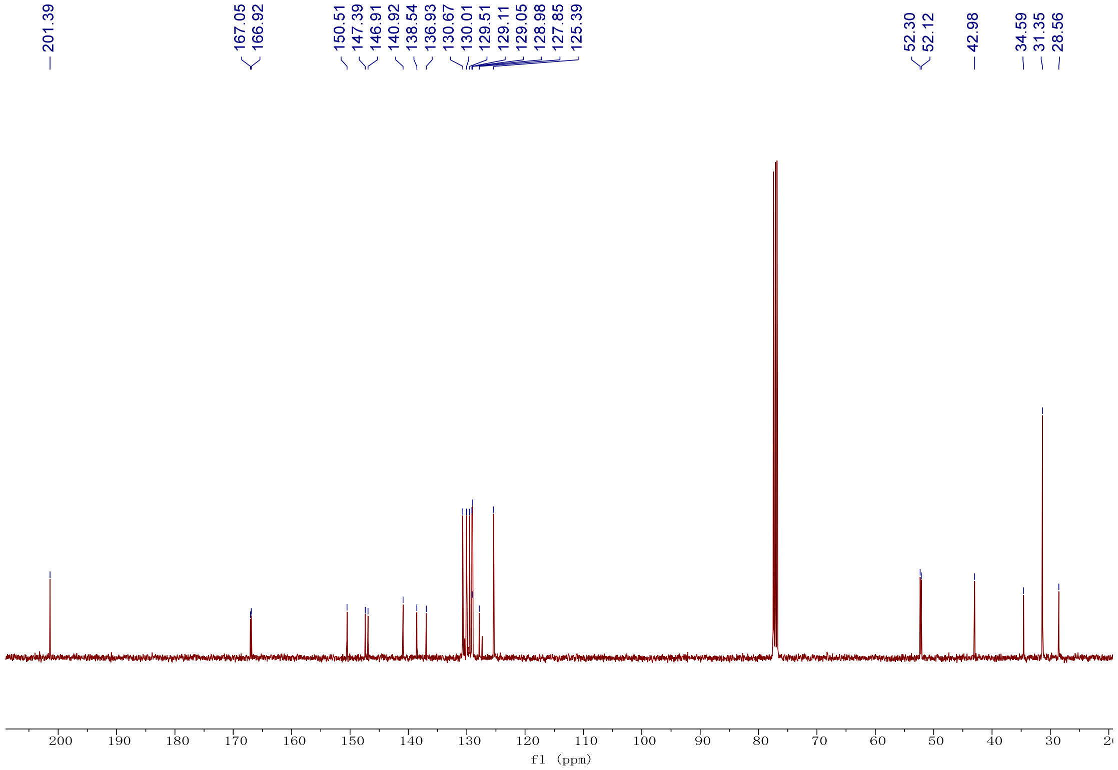

Supplementary Figure 123. ^{13}C NMR (101 MHz, $CDCl_3$) of **5x**

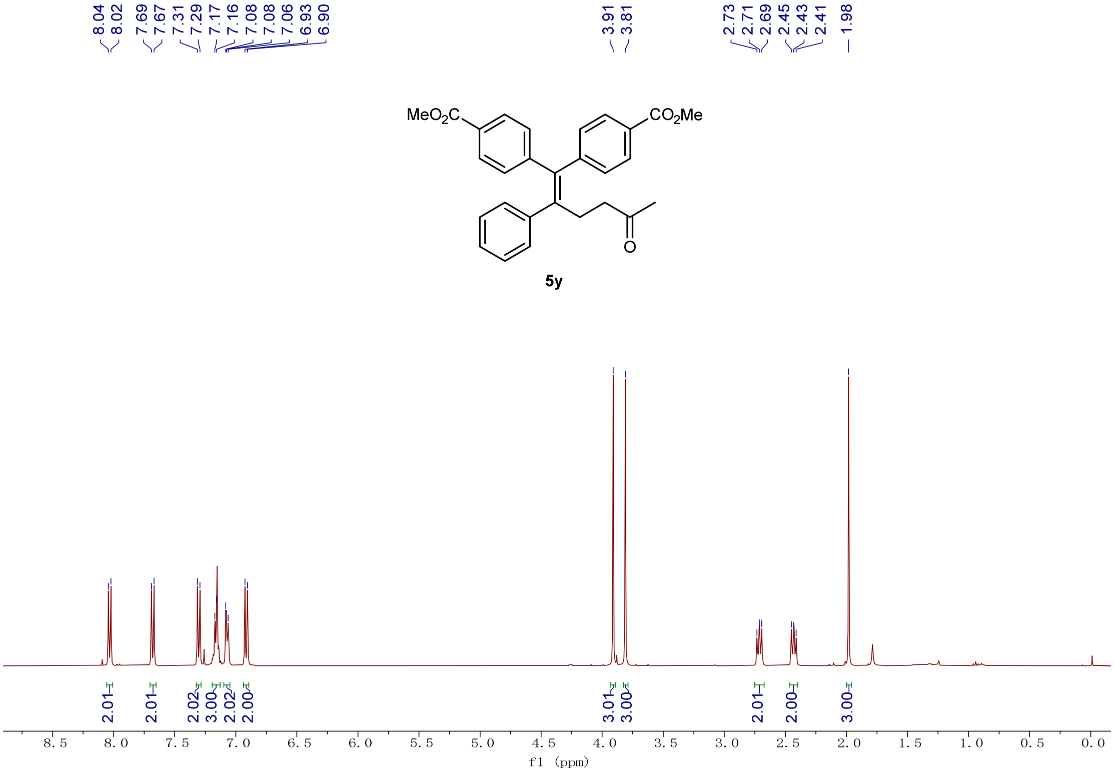

Supplementary Figure 124. ^{1}H NMR (400 MHz, $CDCl_3$) of **5y**

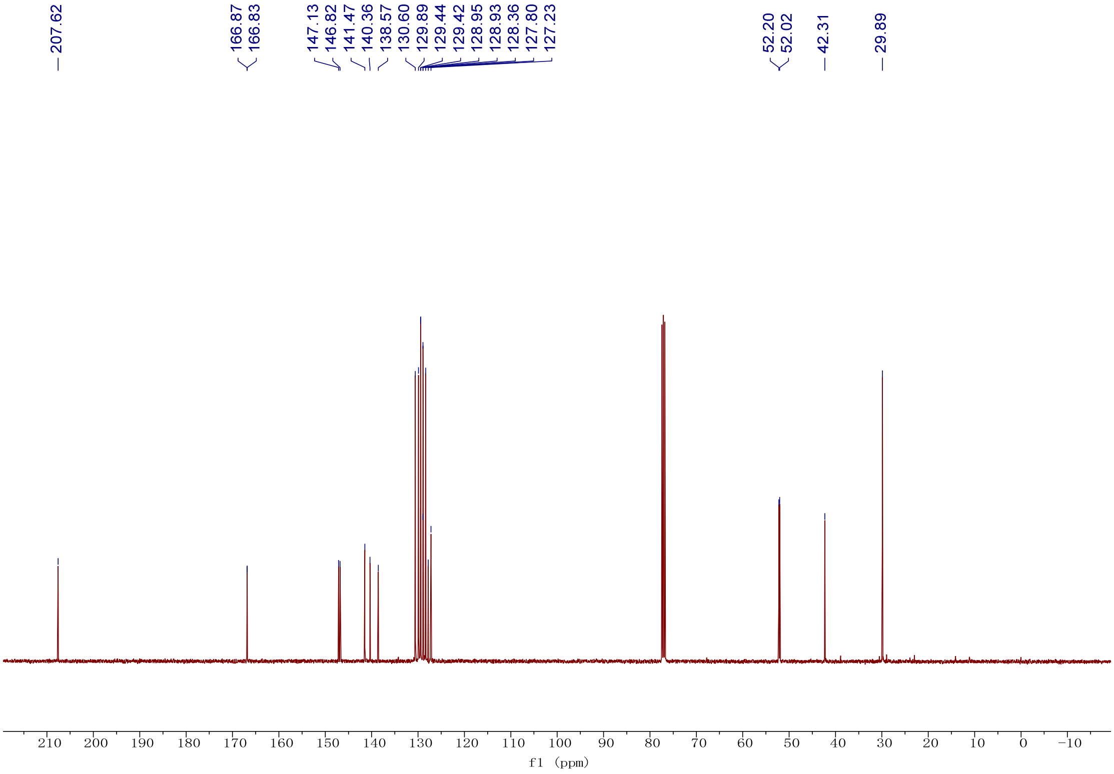

Supplementary Figure 125. ^{13}C NMR (101 MHz, $CDCl_3$) of **5y**

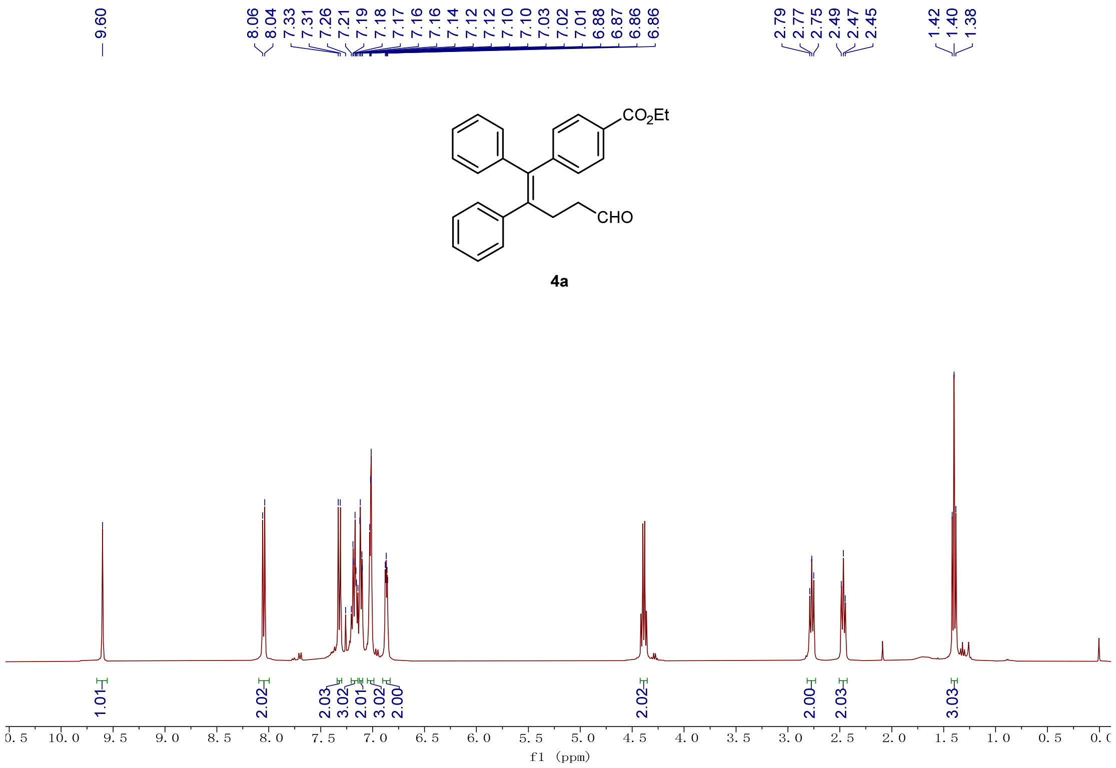

Supplementary Figure 126. ^{1}H NMR (400 MHz, $CDCl_3$) of **4a**

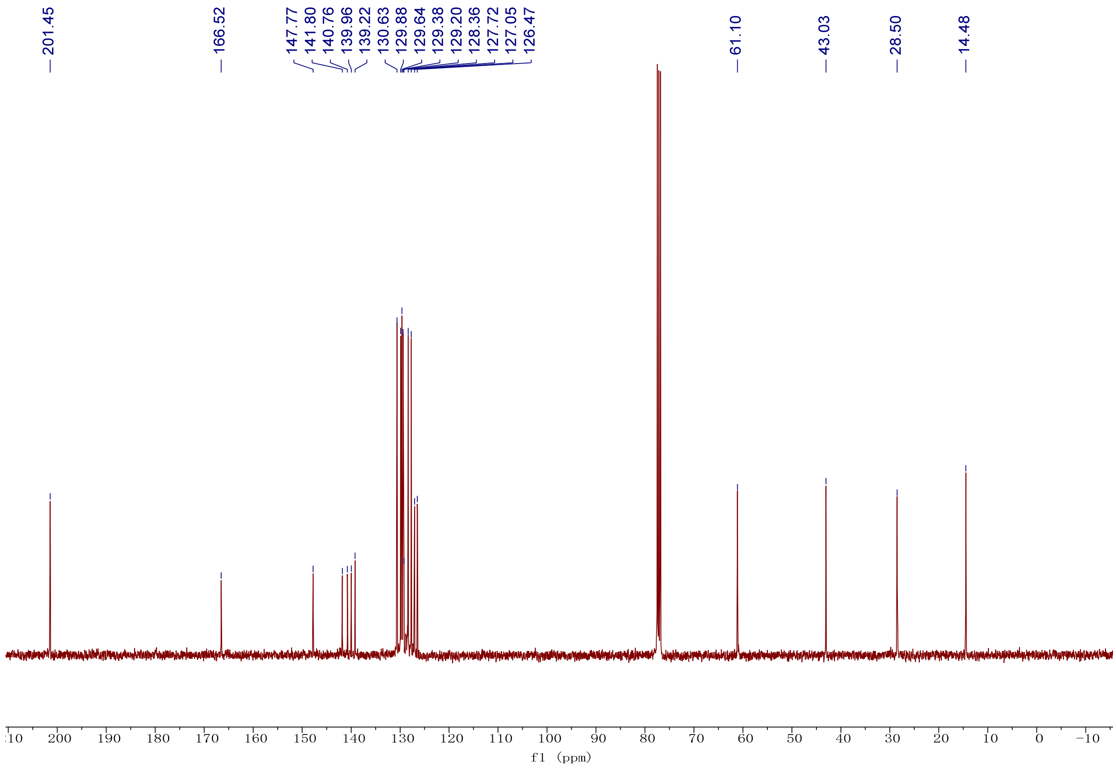

Supplementary Figure 127. ^{13}C NMR (101 MHz, $CDCl_3$) of **4a**

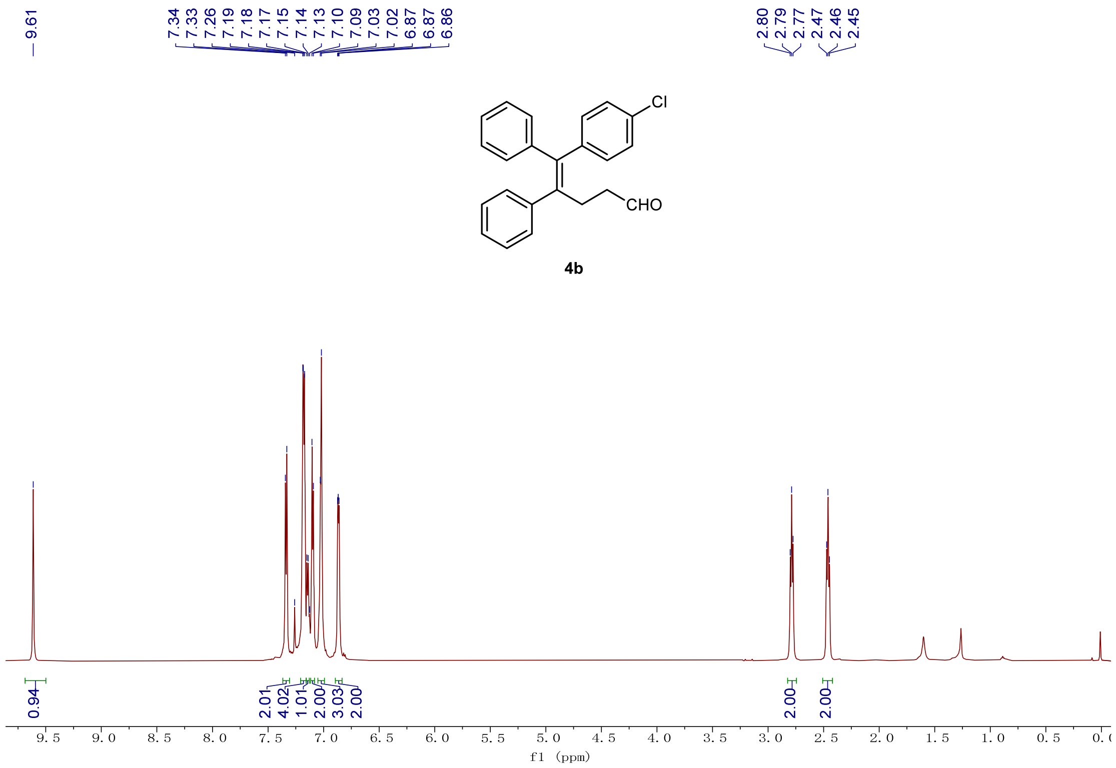

Supplementary Figure 128. ^{1}H NMR (600 MHz, $CDCl_3$) of **4b**

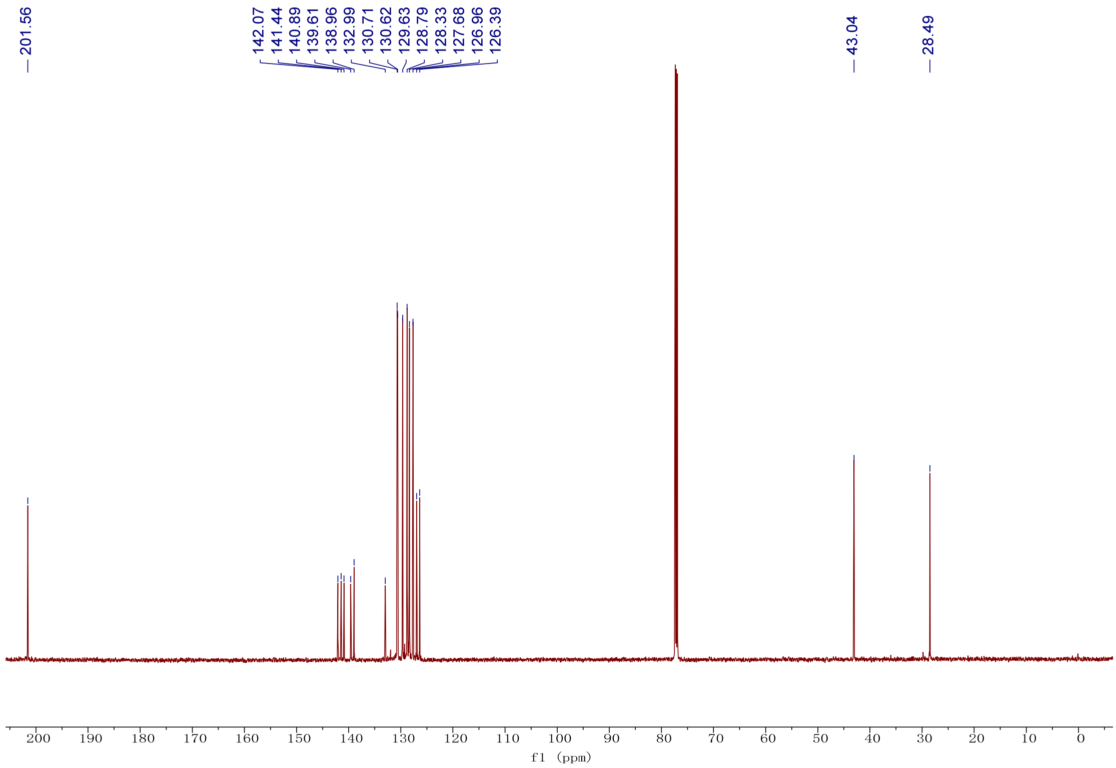

Supplementary Figure 129. ^{13}C NMR (151 MHz, $CDCl_3$) of **4b**

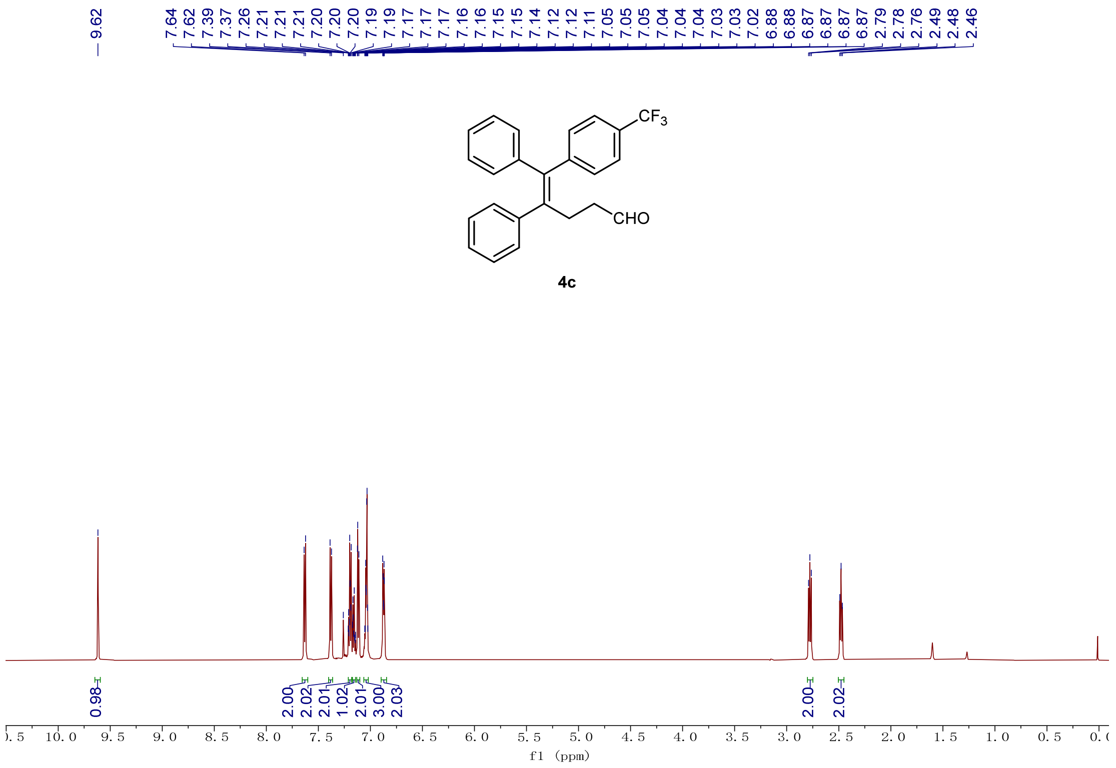

Supplementary Figure 130. ^{1}H NMR (600 MHz, $CDCl_3$) of **4c**

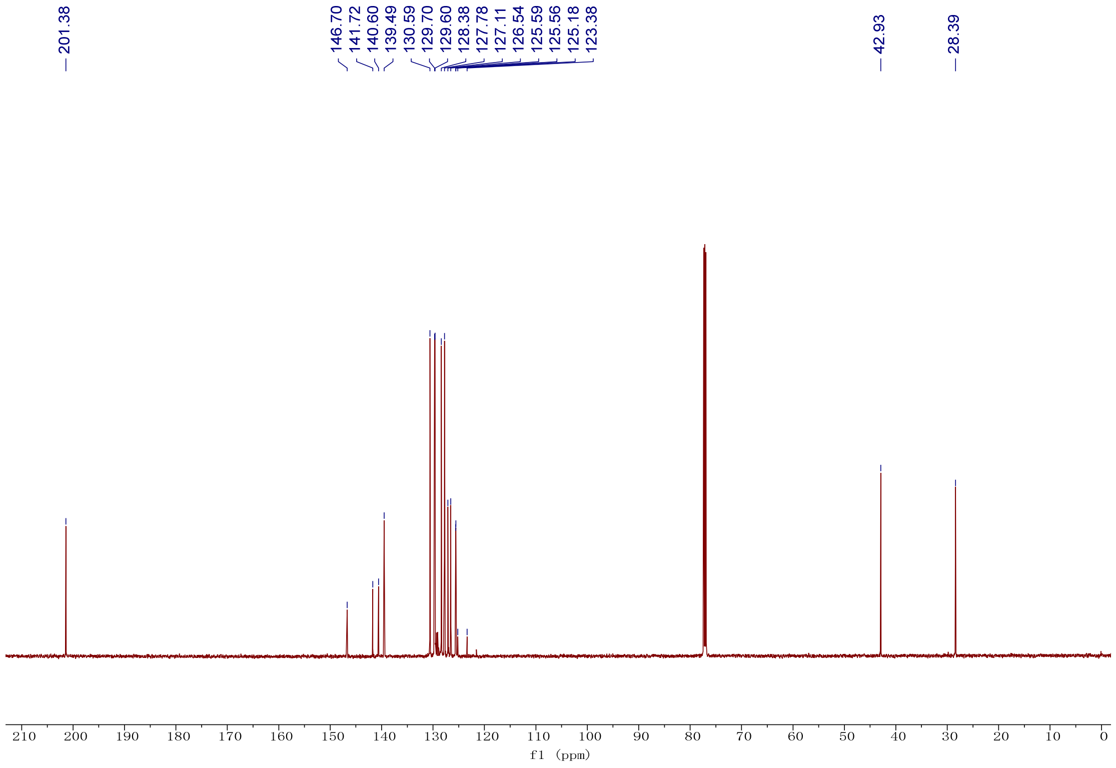

Supplementary Figure 131. ^{13}C NMR (151 MHz, $CDCl_3$) of **4c**

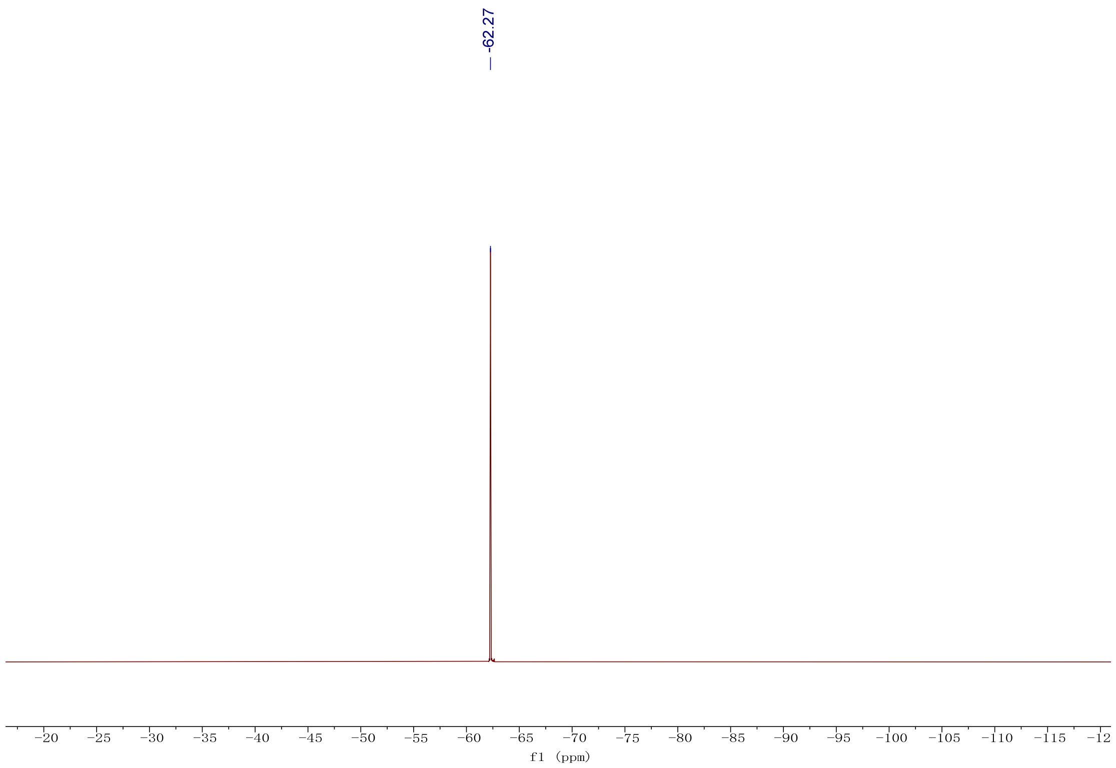

Supplementary Figure 132. ^{19}F NMR (565 MHz, $CDCl_3$) of **4c**

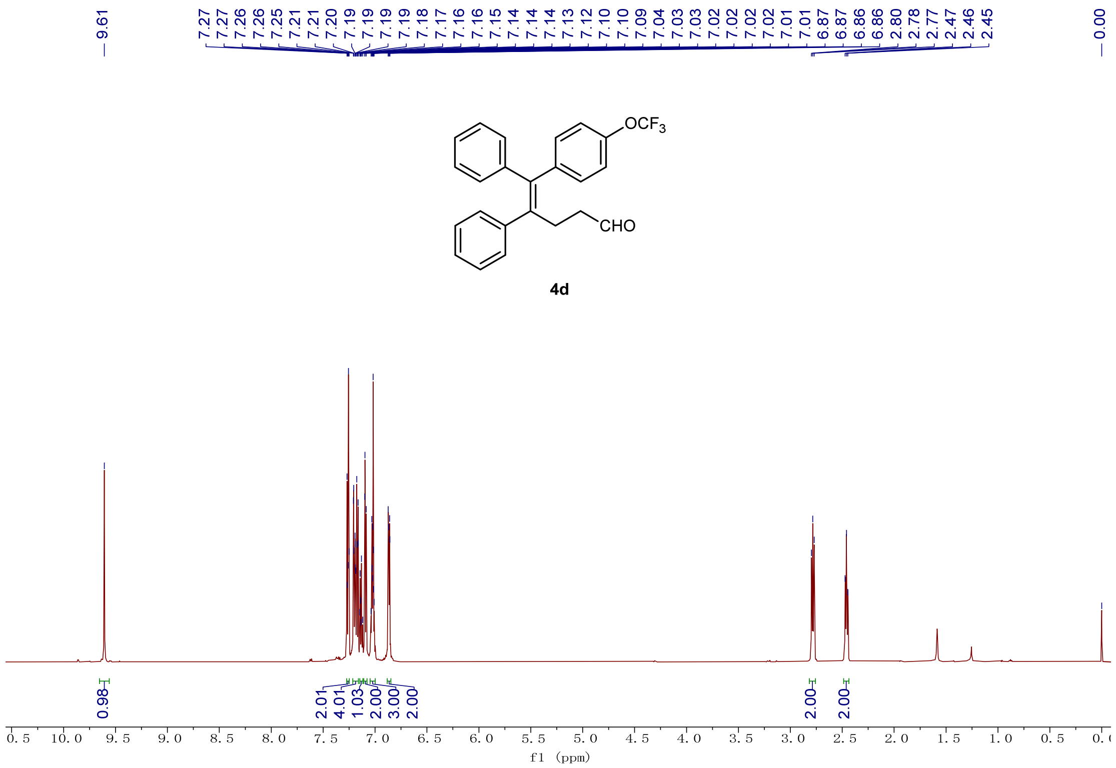

Supplementary Figure 133. ^{1}H NMR (600 MHz, $CDCl_3$) of **4d**

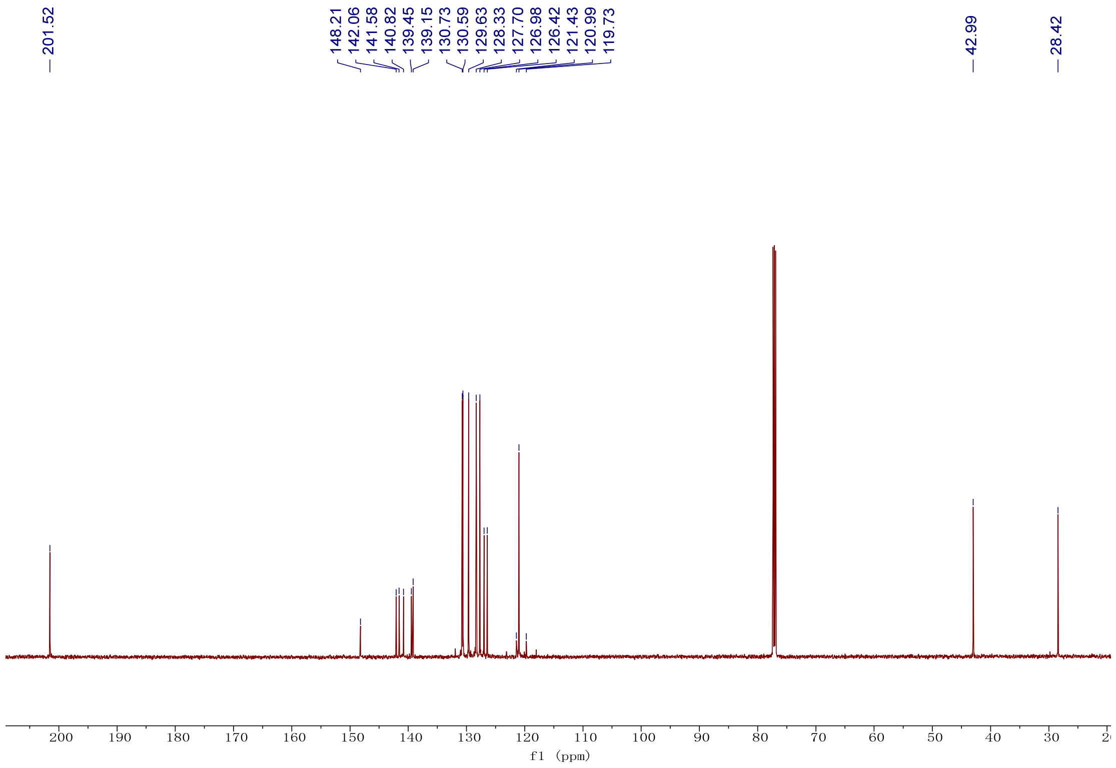

Supplementary Figure 134. ^{13}C NMR (151 MHz, $CDCl_3$) of **4d**

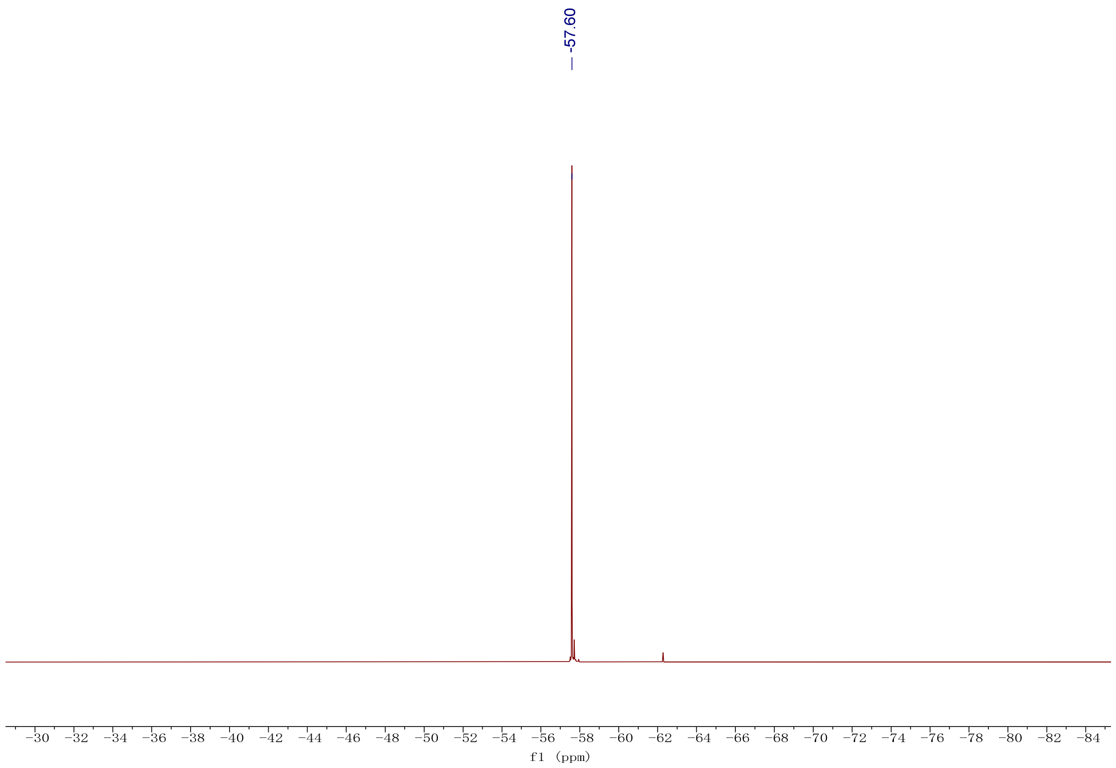

Supplementary Figure 135. ^{19}F NMR (565 MHz, $CDCl_3$) of **4d**

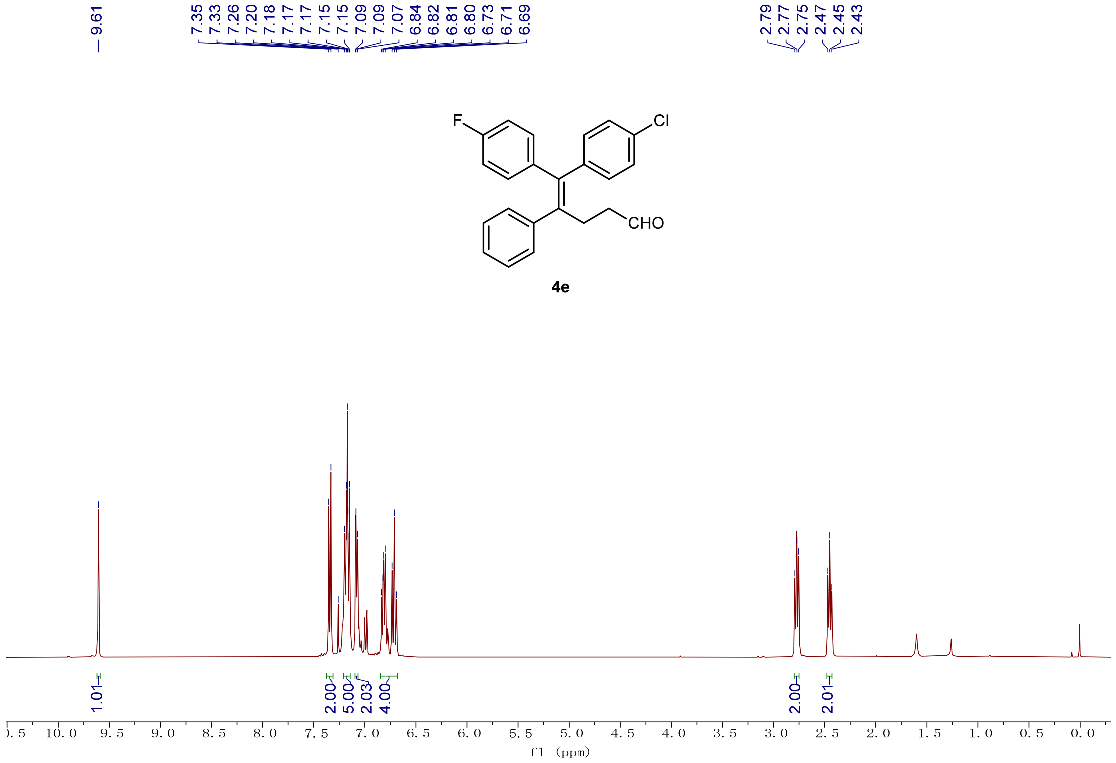

Supplementary Figure 136. ^{1}H NMR (400 MHz, $CDCl_3$) of **4e**

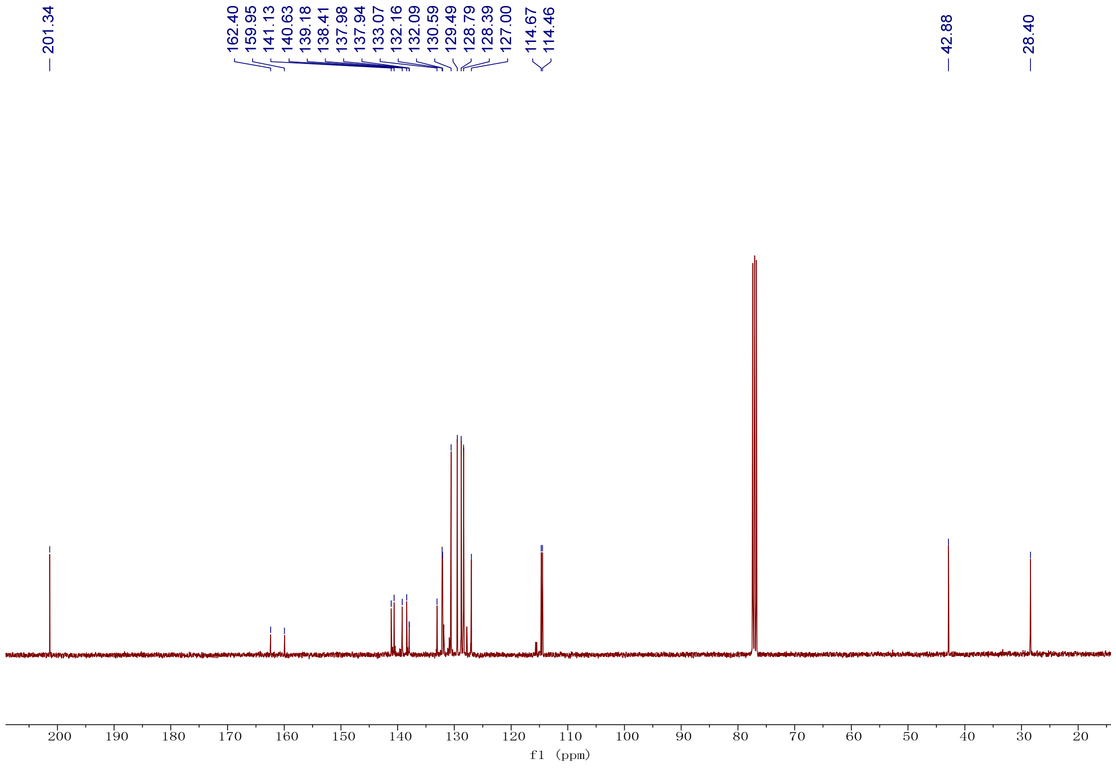

Supplementary Figure 137. ^{13}C NMR (101 MHz, $CDCl_3$) of **4e**

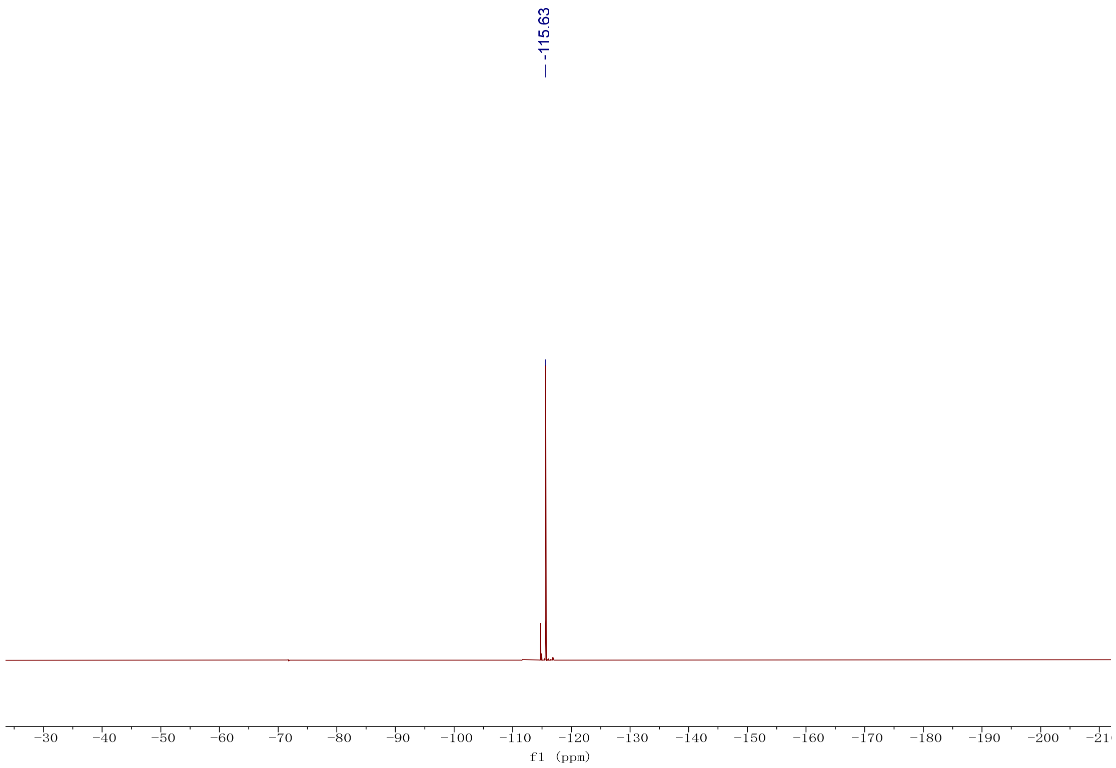

Supplementary Figure 138. ^{19}F NMR (376 MHz, $CDCl_3$) of **4e**

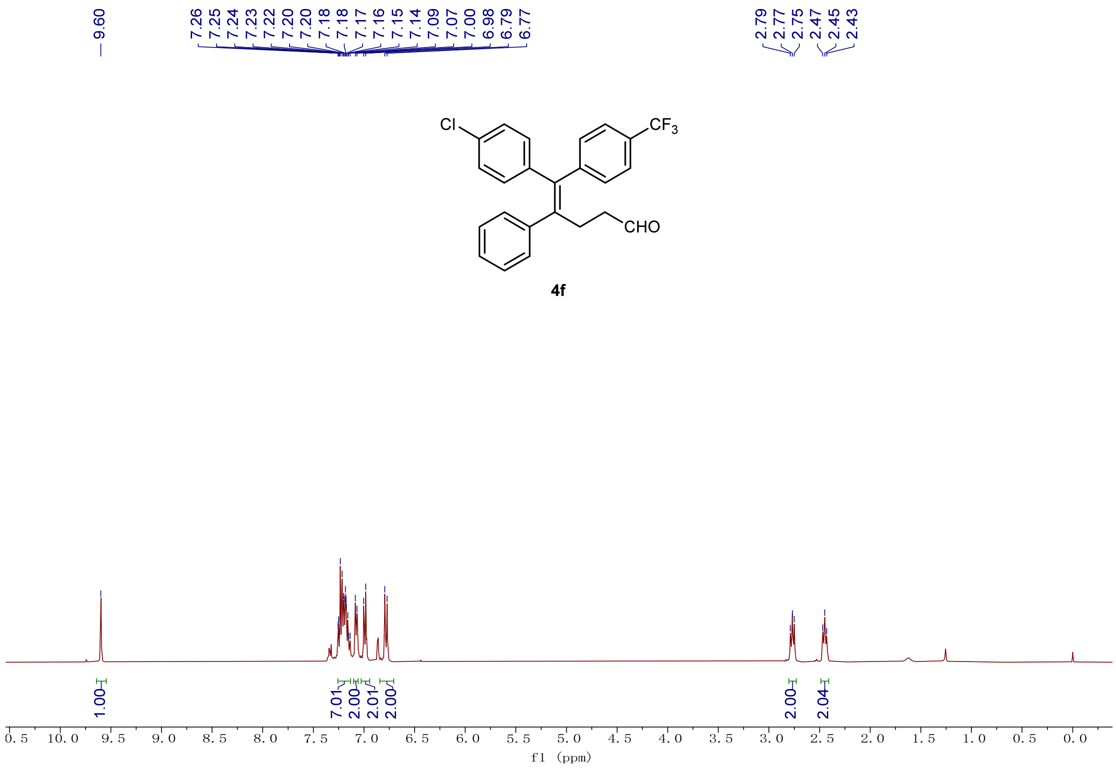

Supplementary Figure 139. ^{1}H NMR (400 MHz, $CDCl_3$) of **4f**

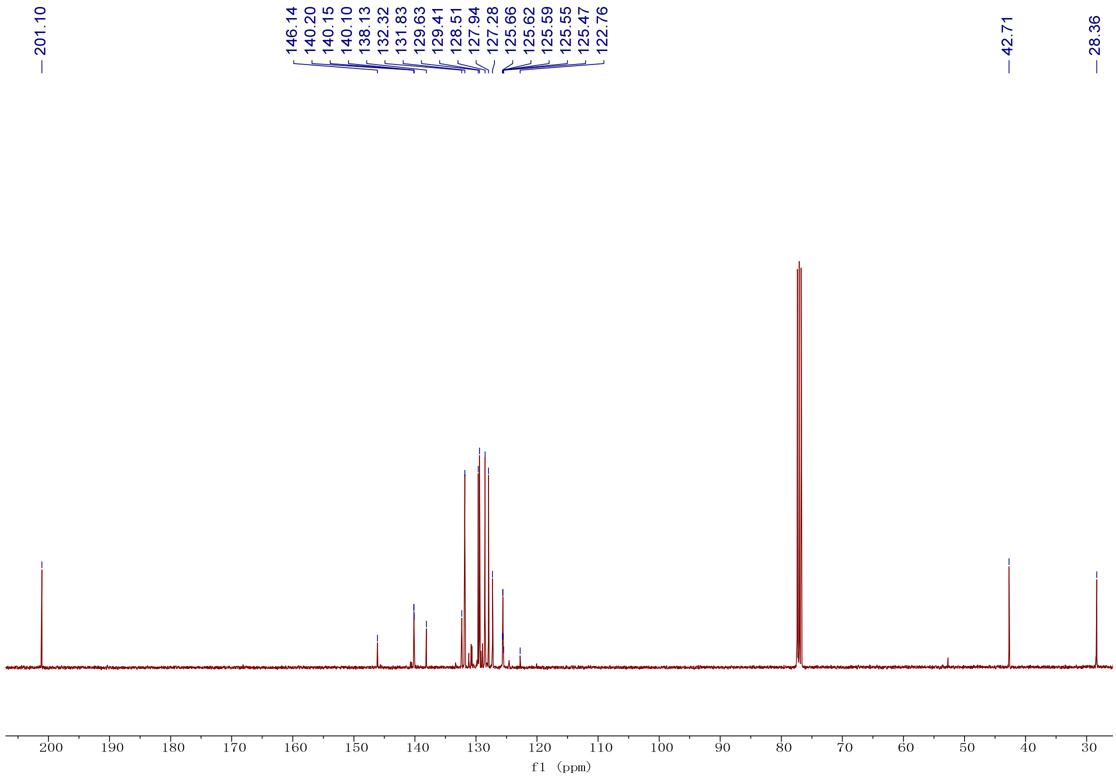

Supplementary Figure 140. ^{13}C NMR (101 MHz, $CDCl_3$) of **4f**

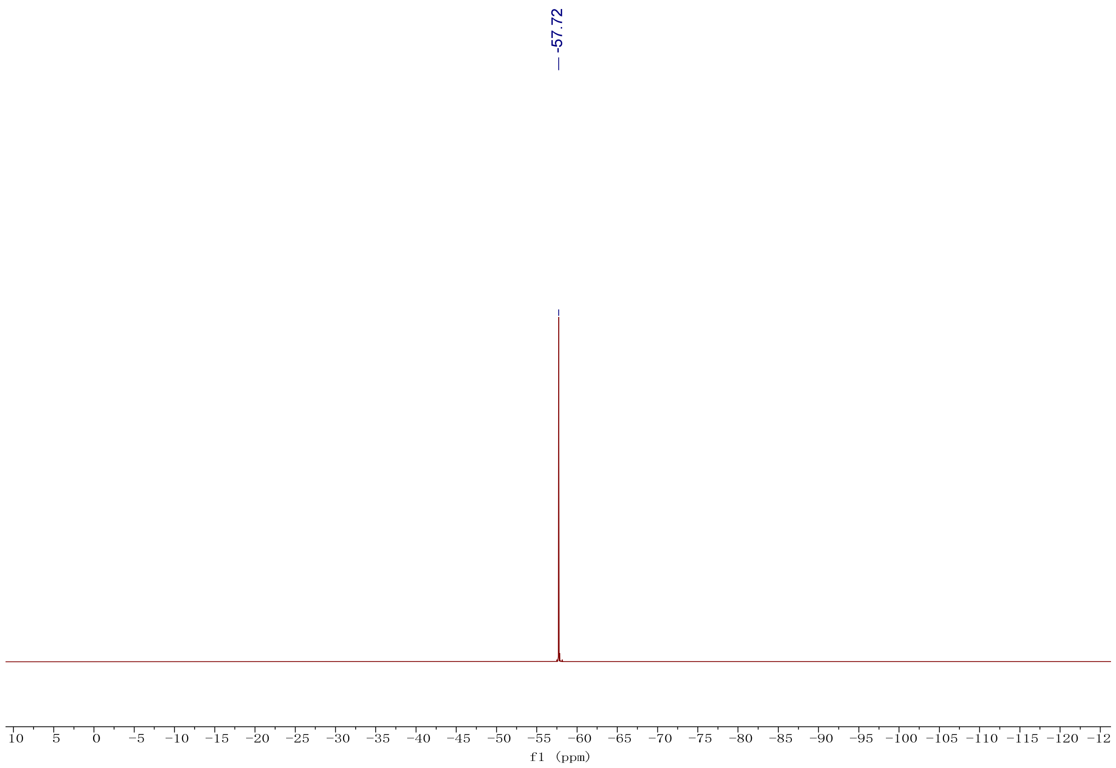

Supplementary Figure 141. ^{19}F NMR (376 MHz, $CDCl_3$) of **4f**

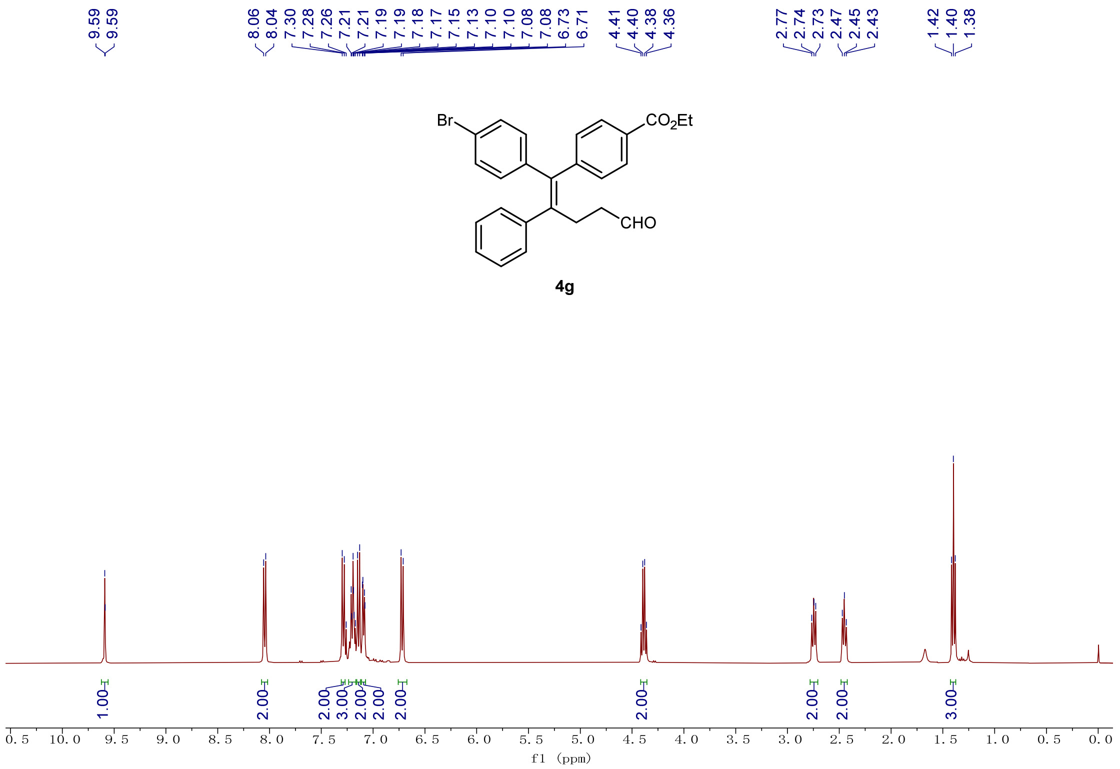

Supplementary Figure 142. ^{1}H NMR (400 MHz, $CDCl_3$) of **4g**

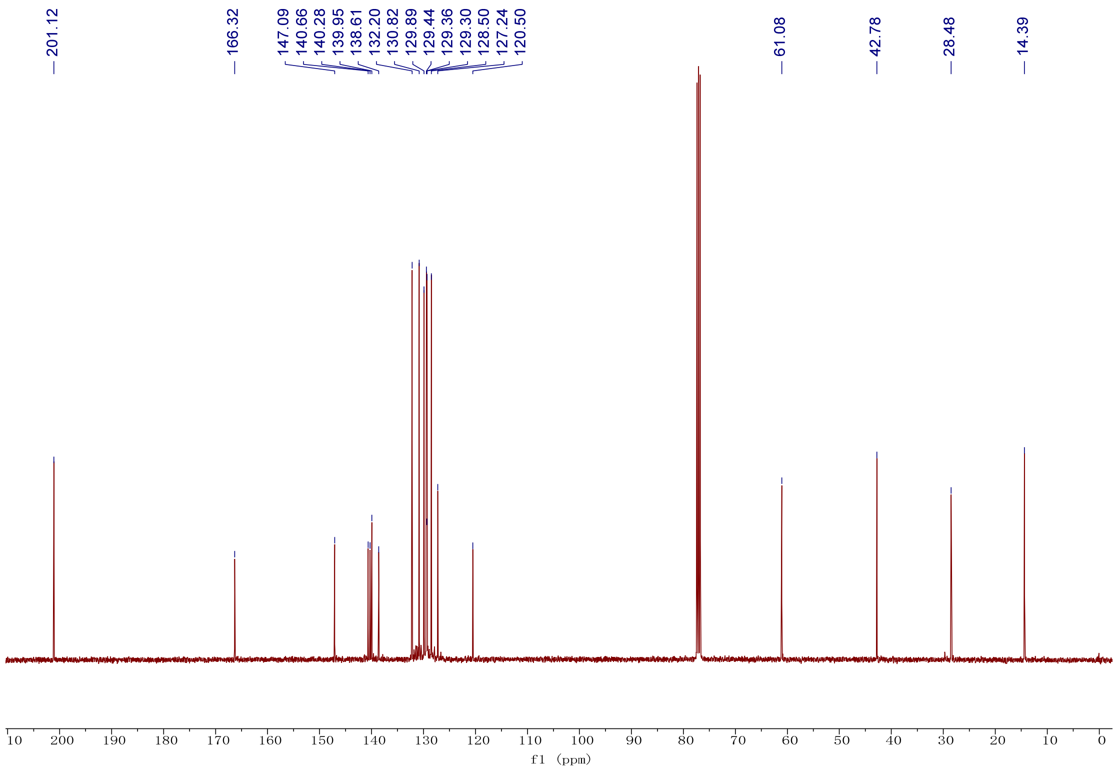

Supplementary Figure 143. ^{13}C NMR (101 MHz, $CDCl_3$) of **4g**

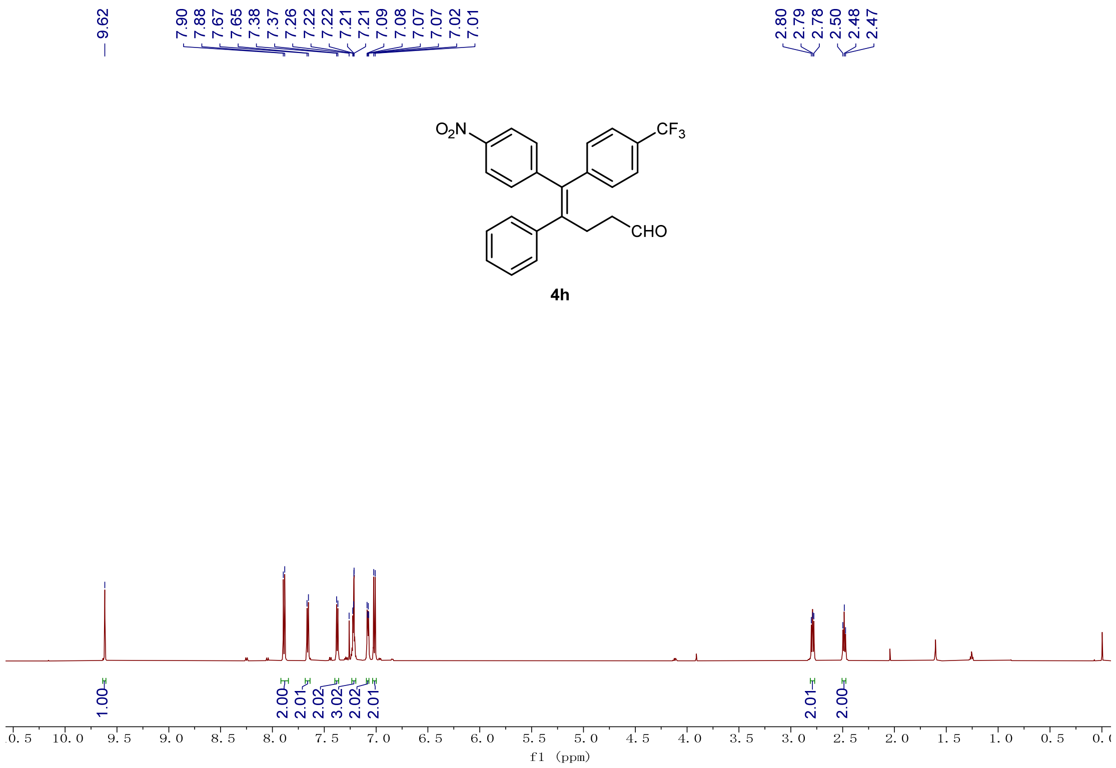

Supplementary Figure 144. ^{1}H NMR (600 MHz, $CDCl_3$) of **4h**

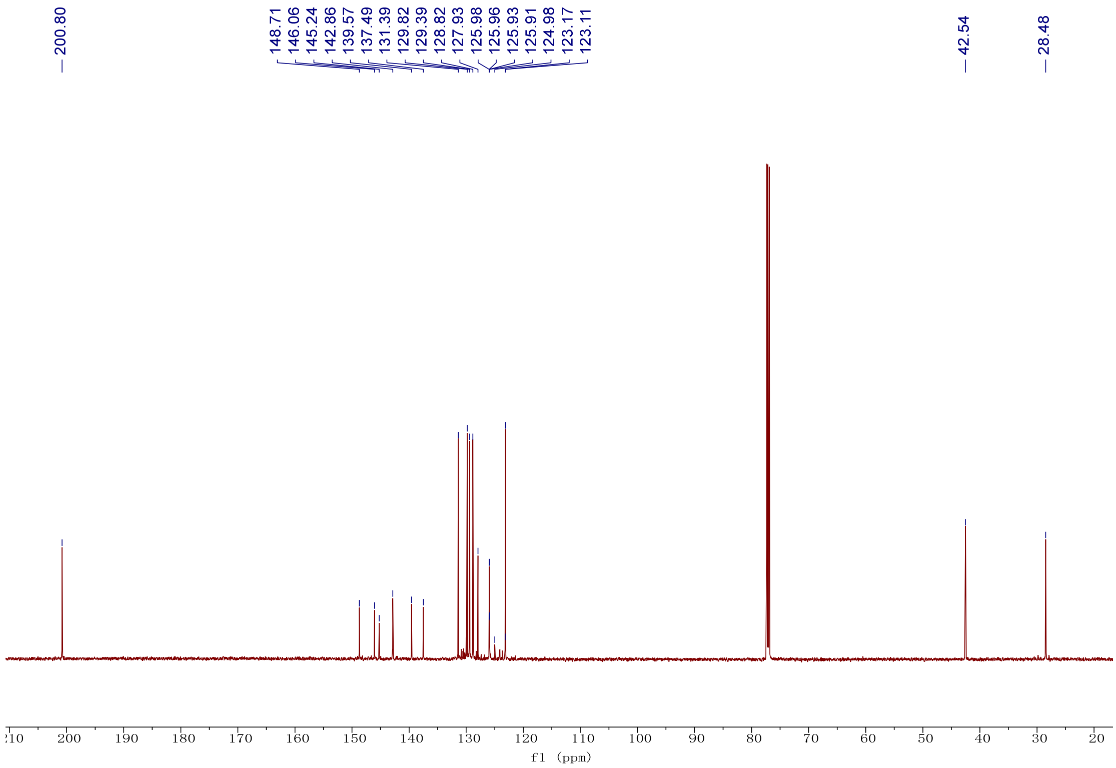

Supplementary Figure 145. ^{13}C NMR (151 MHz, $CDCl_3$) of **4h**

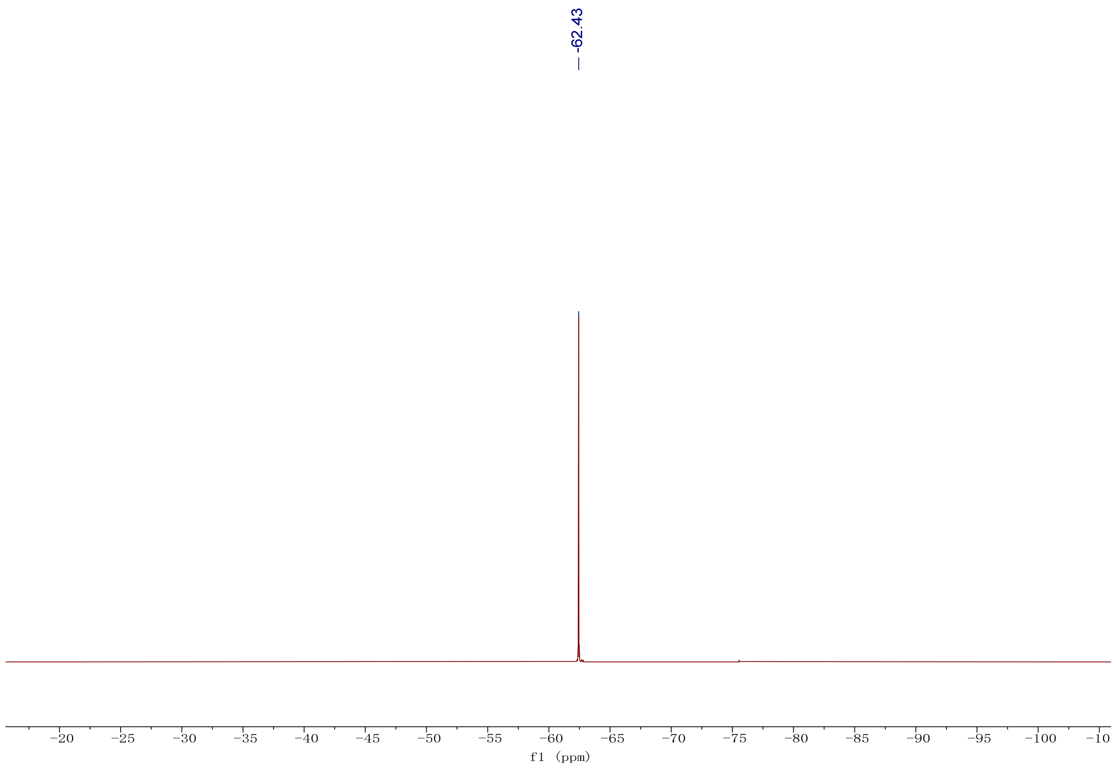

Supplementary Figure 146. ^{19}F NMR (565 MHz, $CDCl_3$) of **4h**

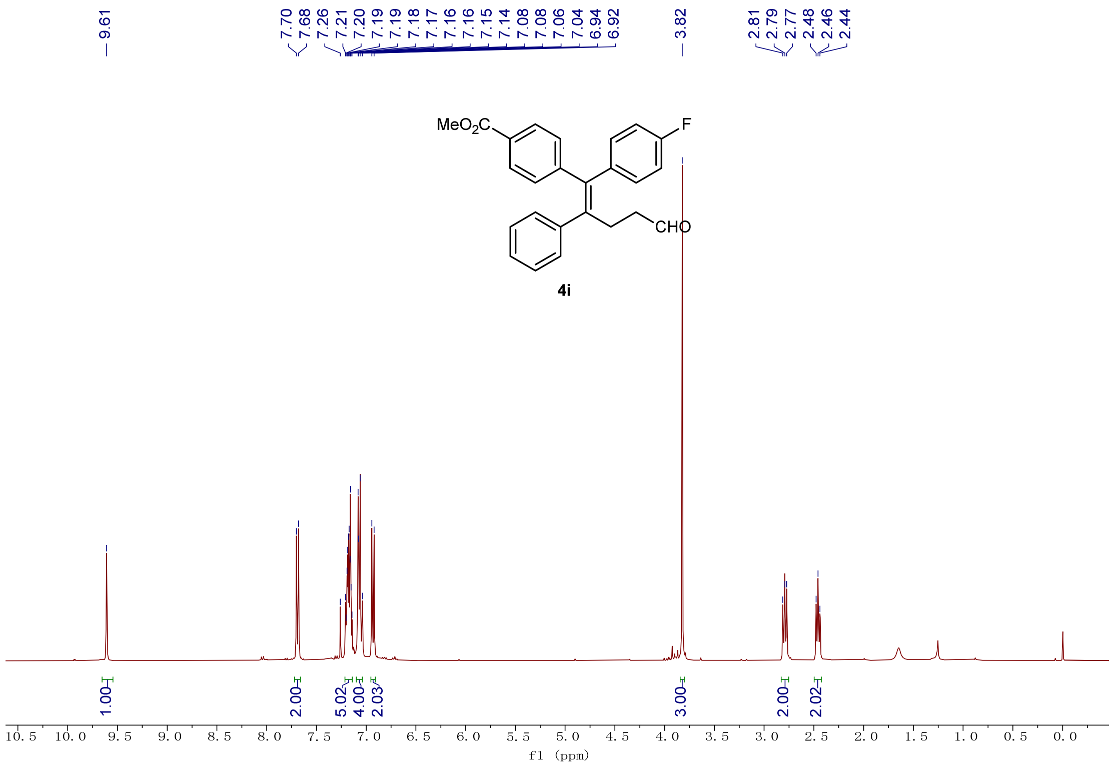

Supplementary Figure 147. ^{1}H NMR (400 MHz, $CDCl_3$) of **4i**

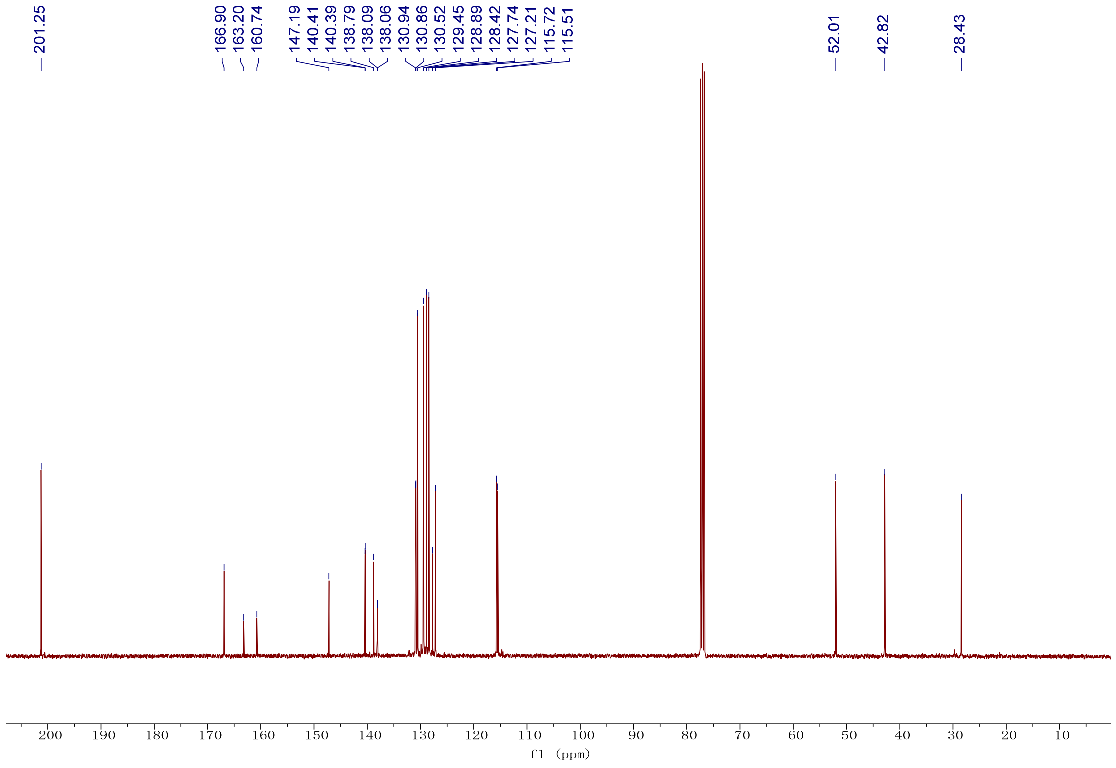

Supplementary Figure 148. ^{13}C NMR (101 MHz, $CDCl_3$) of **4i**

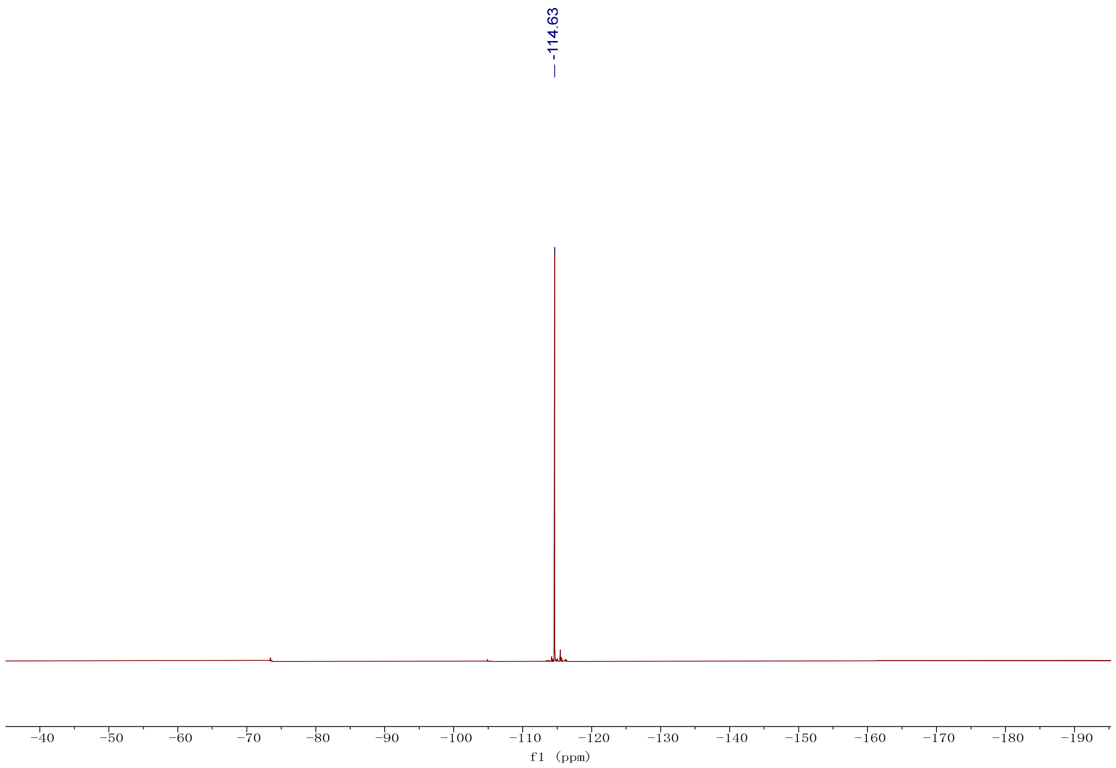

Supplementary Figure 149. ^{19}F NMR (376 MHz, $CDCl_3$) of **4i**

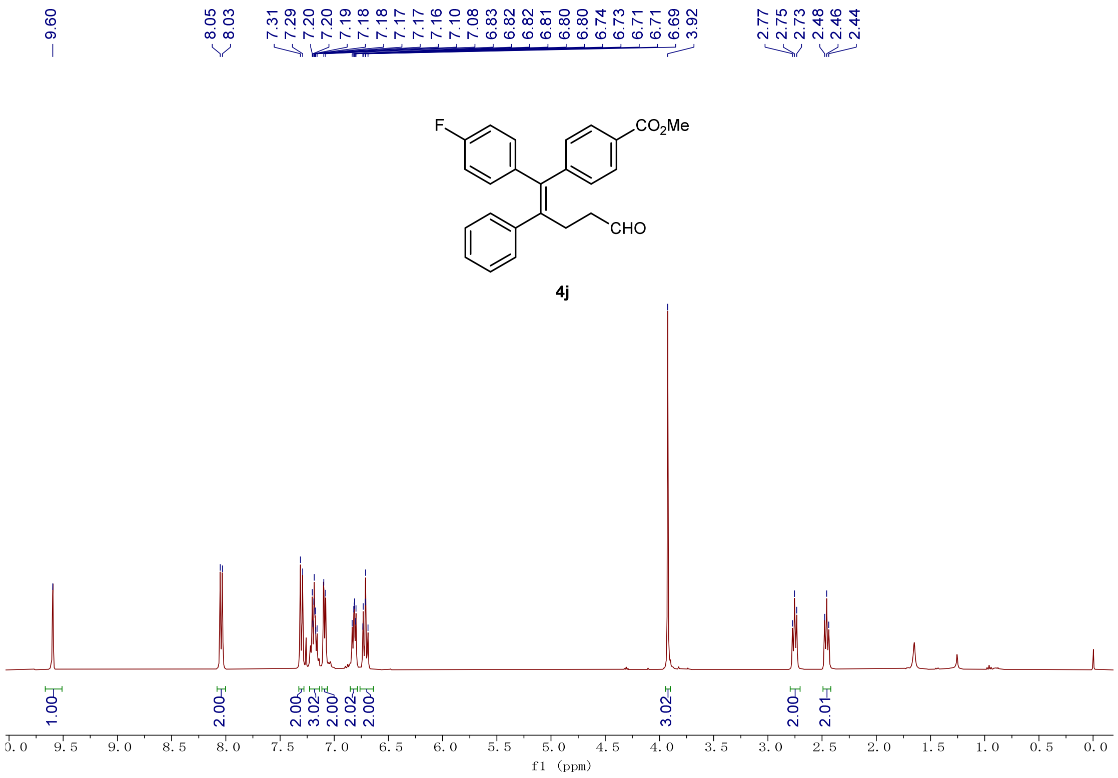

Supplementary Figure 150. ^{1}H NMR (400 MHz, $CDCl_3$) of **4j**

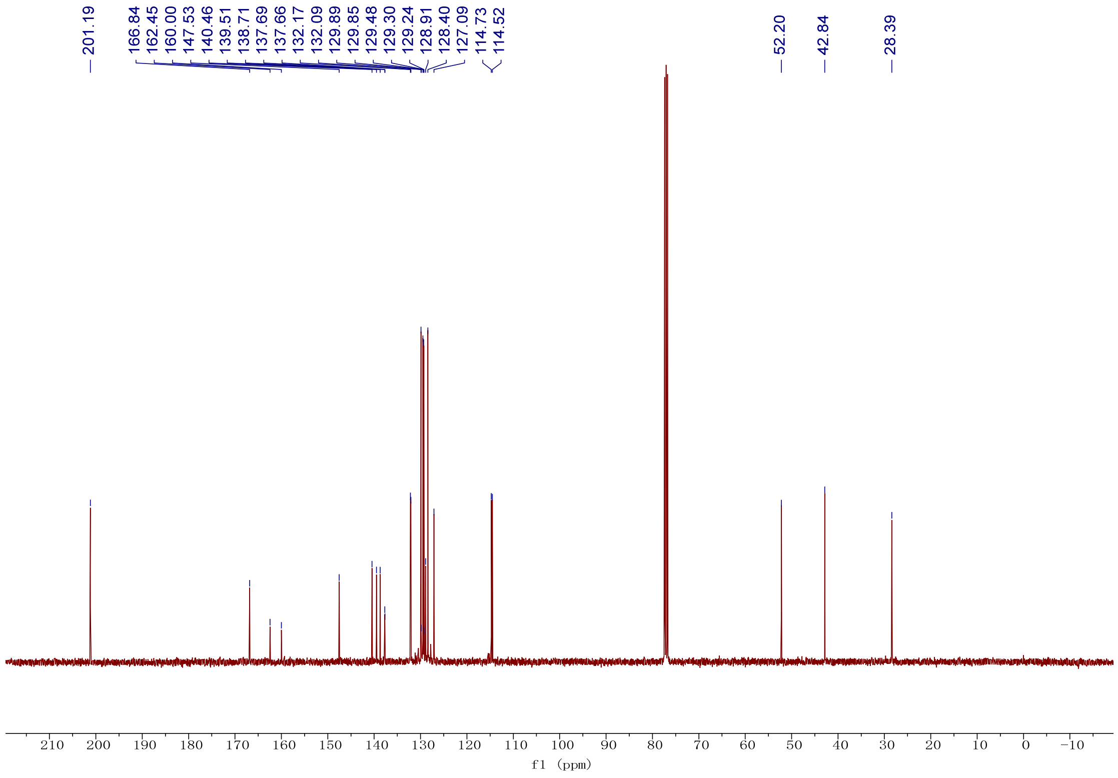

Supplementary Figure 151. ^{13}C NMR (101 MHz, $CDCl_3$) of **4j**

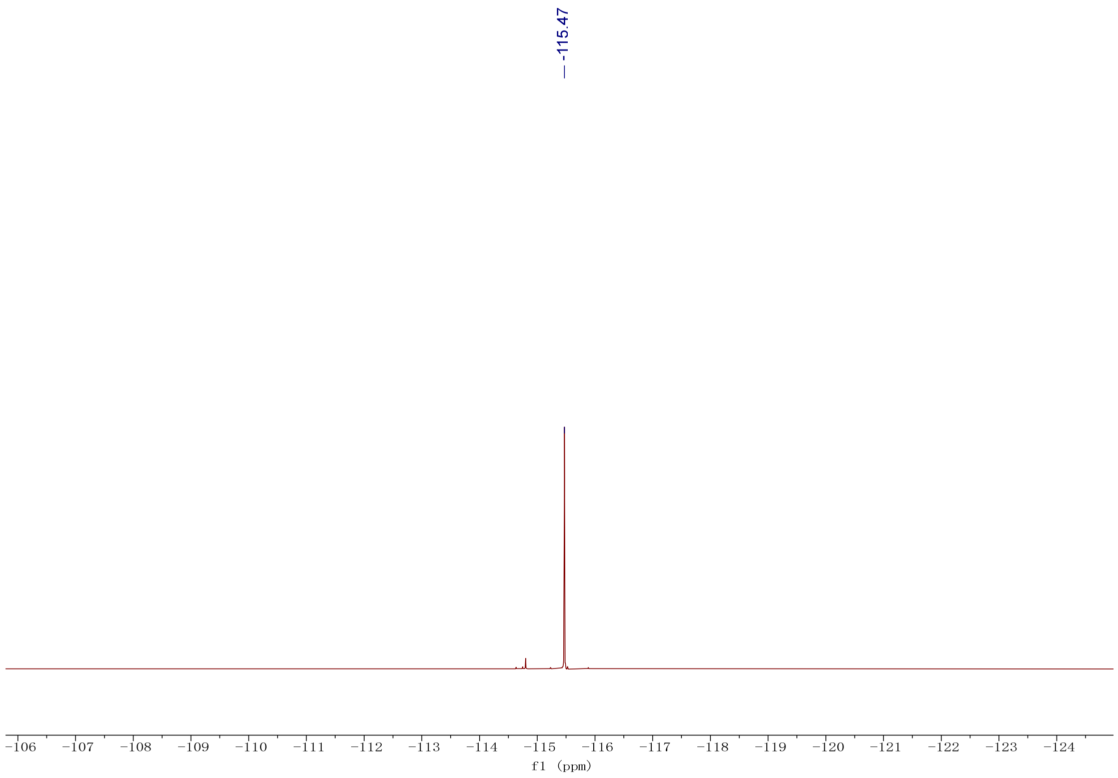

Supplementary Figure 152. ^{19}F NMR (376 MHz, $CDCl_3$) of **4j**

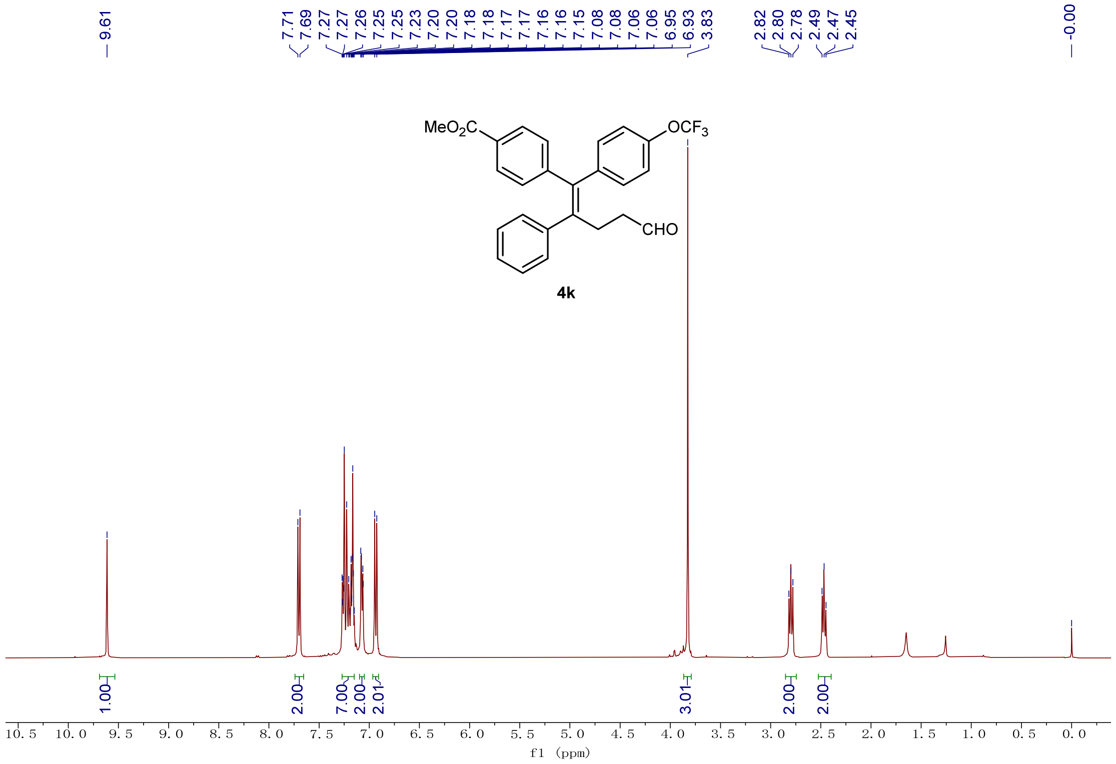

Supplementary Figure 153. ^{1}H NMR (400 MHz, $CDCl_3$) of **4k**

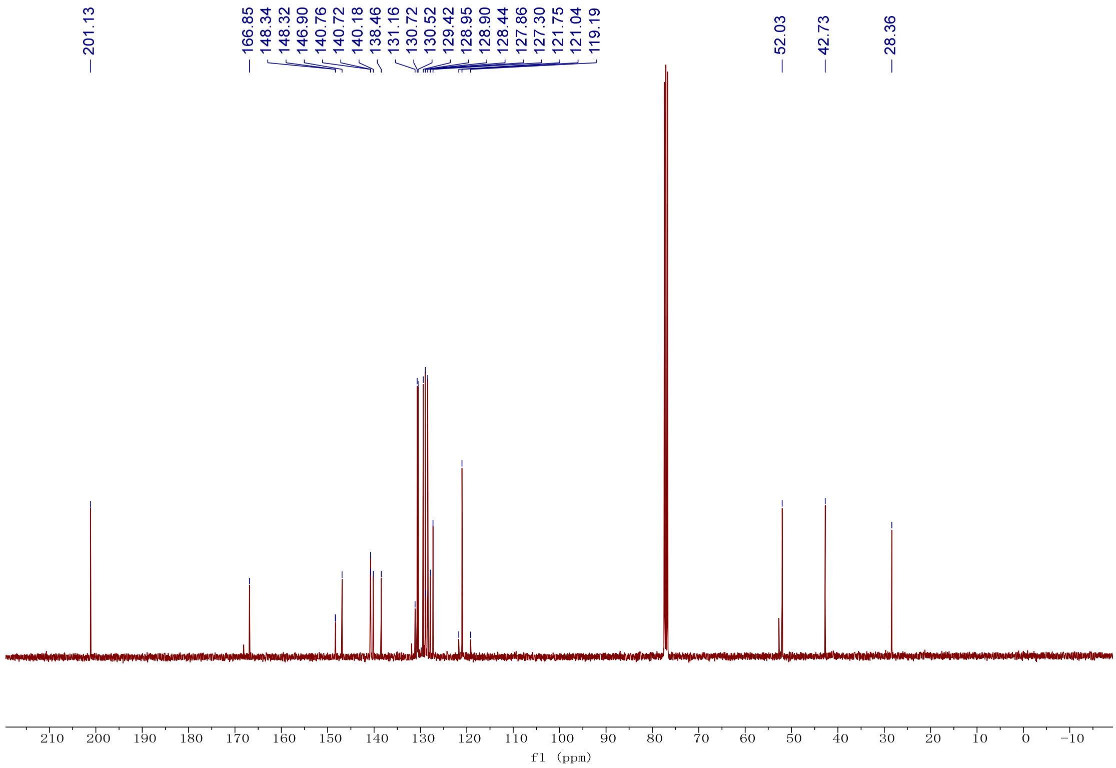

Supplementary Figure 154. ^{13}C NMR (101 MHz, $CDCl_3$) of **4k**

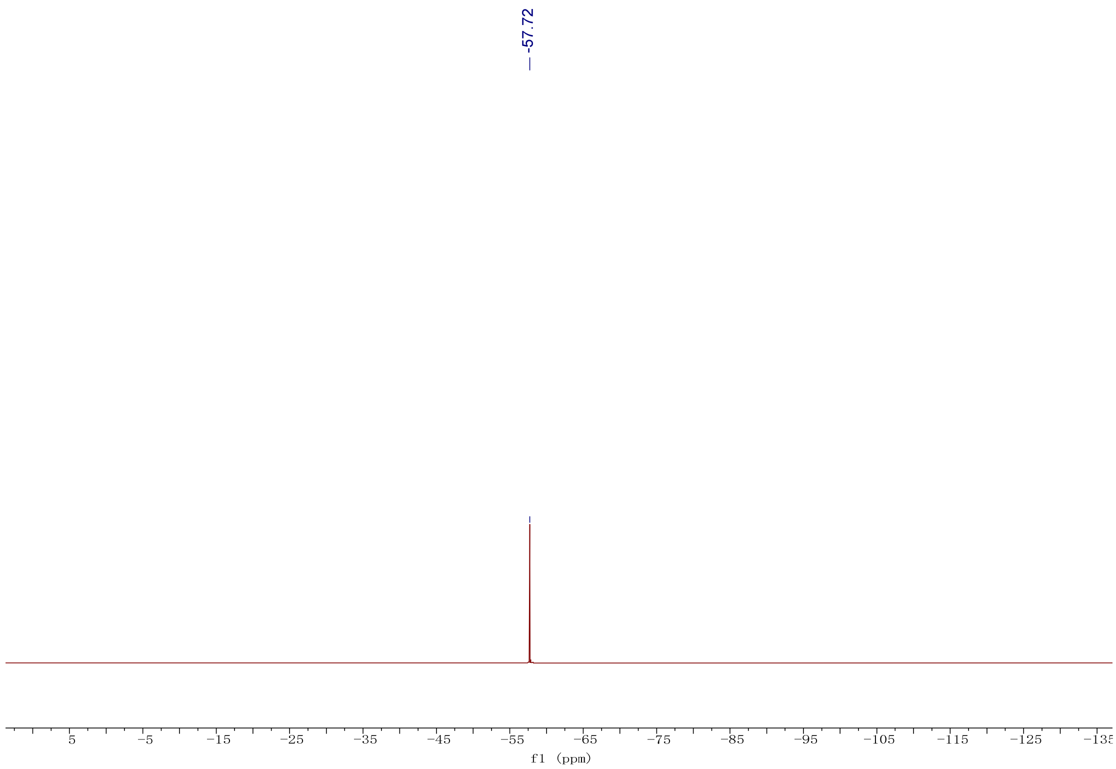

Supplementary Figure 155. ^{19}F NMR (376 MHz, $CDCl_3$) of **4k**

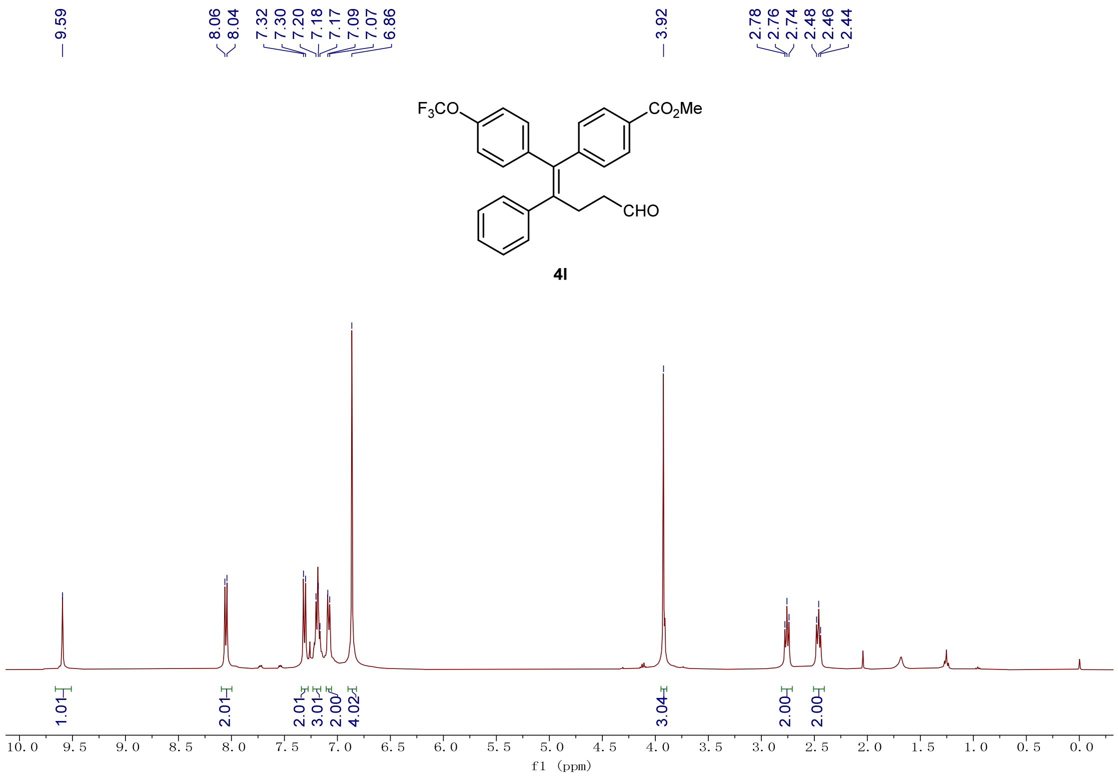

Supplementary Figure 156. ^{1}H NMR (400 MHz, $CDCl_3$) of **4l**

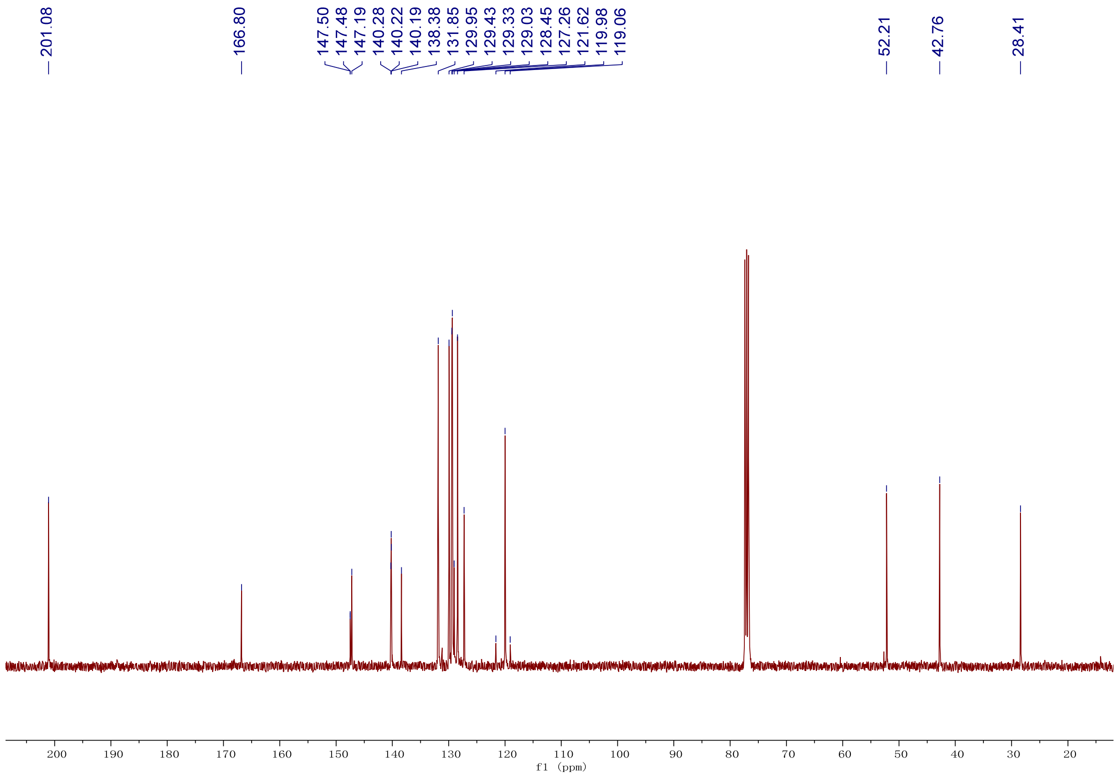

Supplementary Figure 157. ^{13}C NMR (101 MHz, $CDCl_3$) of **4l**

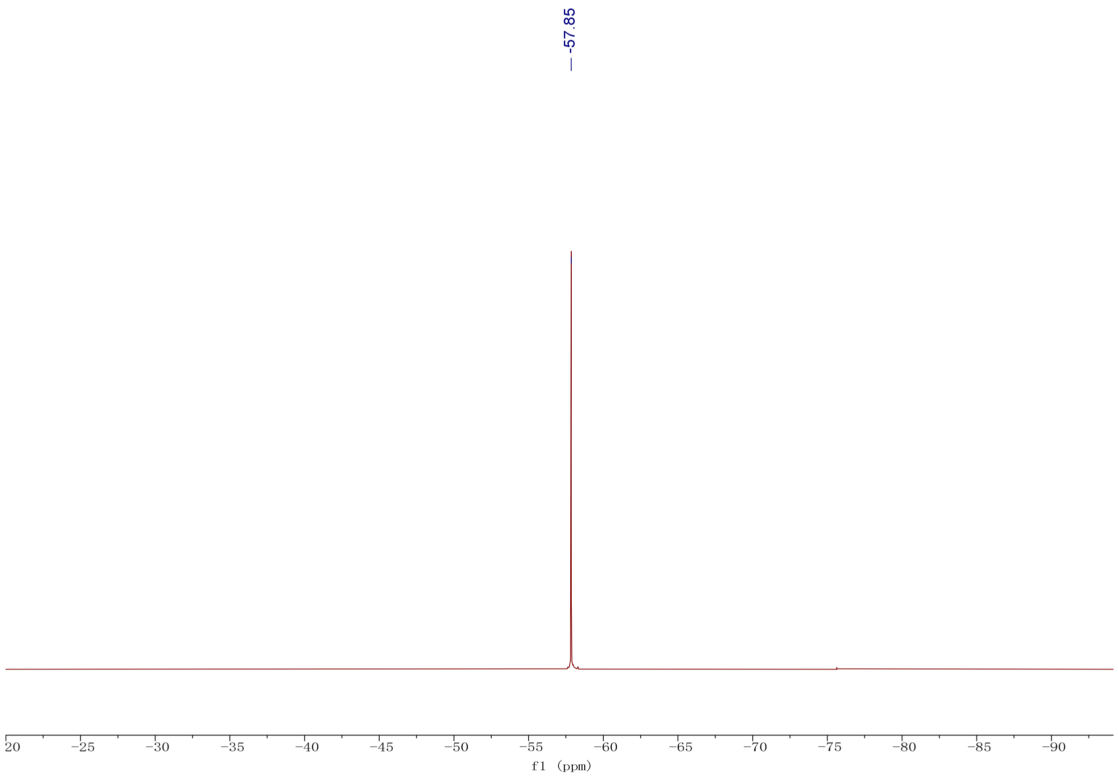

Supplementary Figure 158. ^{19}F NMR (376 MHz, $CDCl_3$) of **4l**

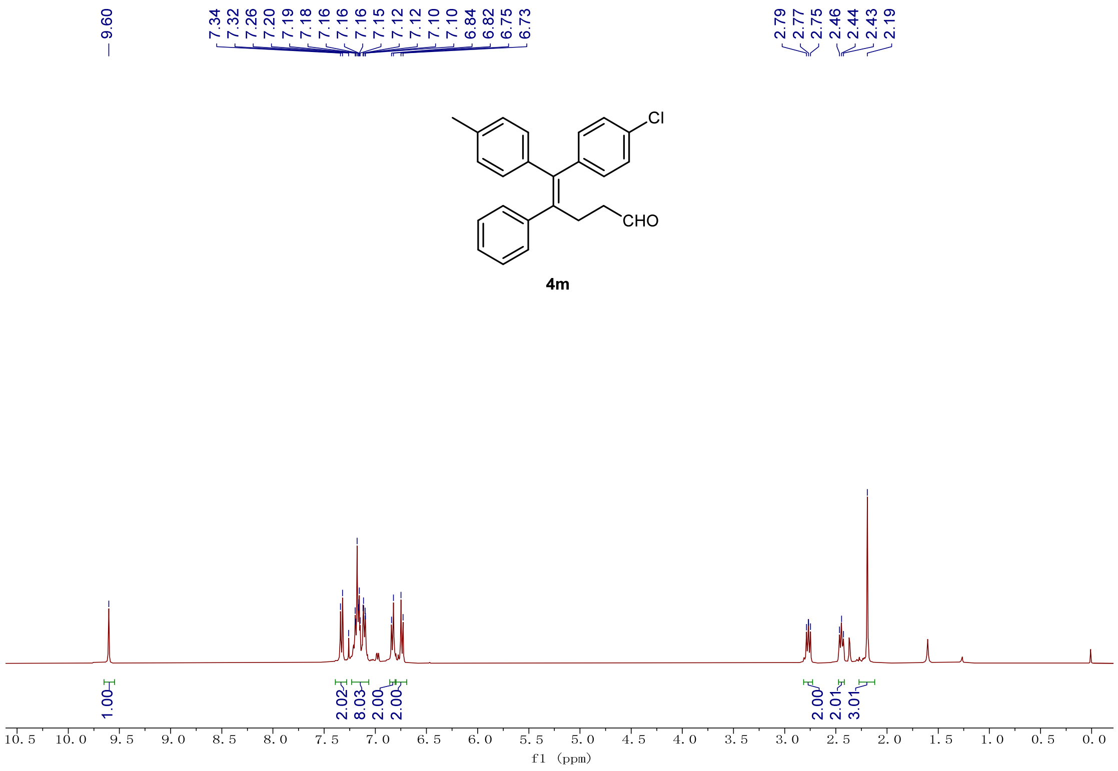

Supplementary Figure 159. ^{1}H NMR (400 MHz, $CDCl_3$) of **4m**

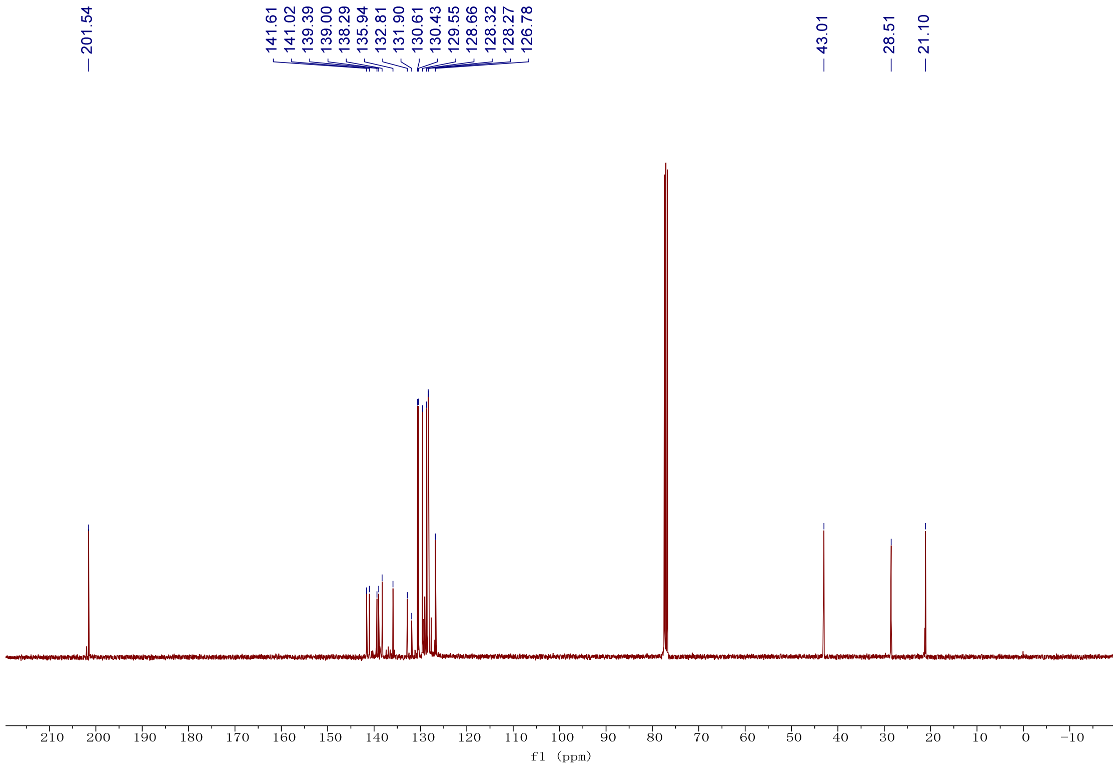

Supplementary Figure 160. ^{13}C NMR (101 MHz, $CDCl_3$) of **4m**

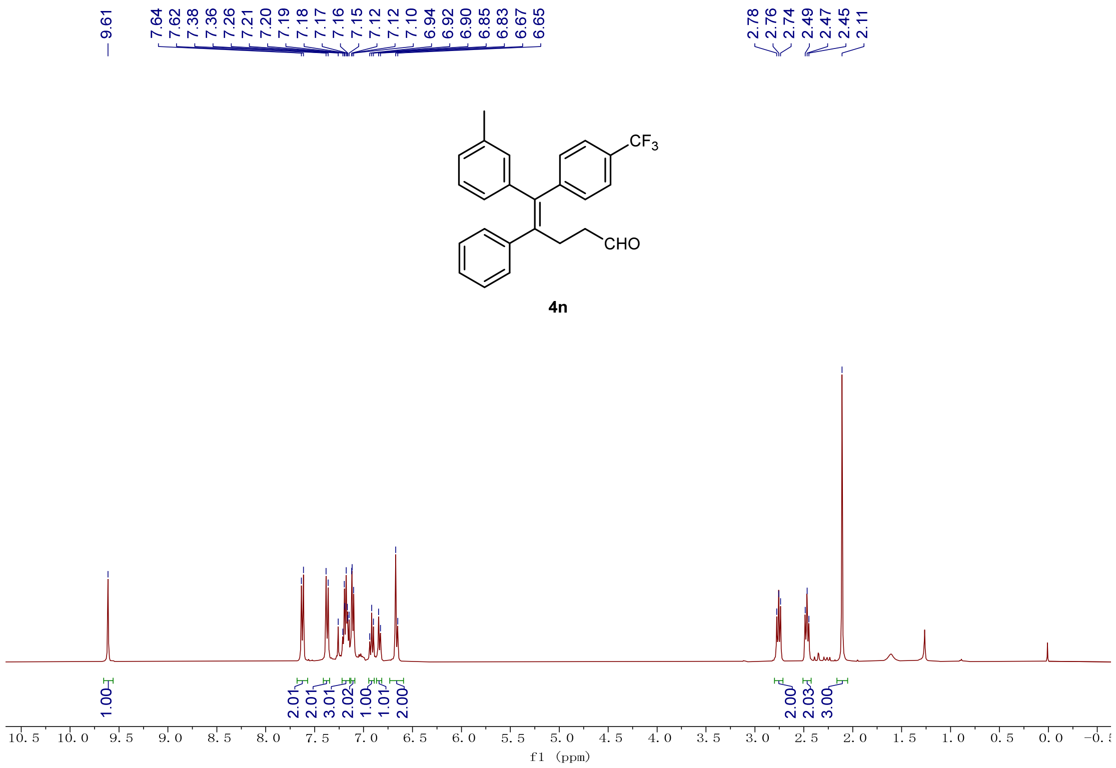

Supplementary Figure 161. ^{1}H NMR (400 MHz, $CDCl_3$) of **4n**

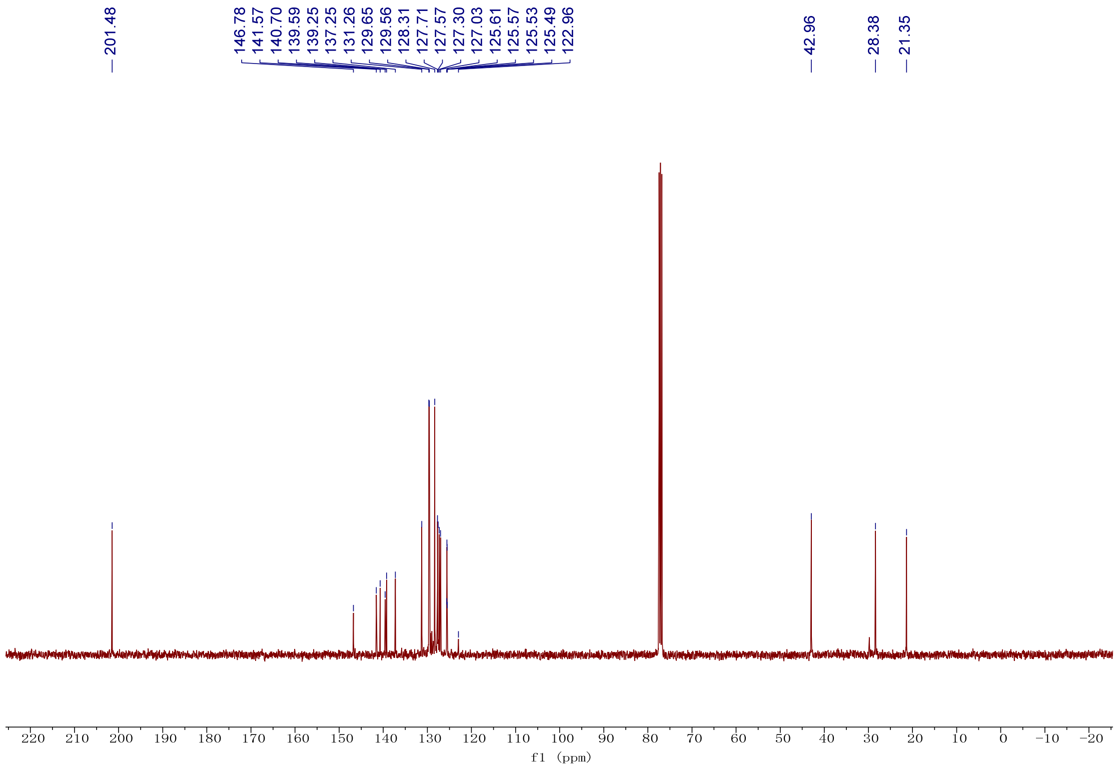

Supplementary Figure 162. ^{13}C NMR (101 MHz, $CDCl_3$) of **4n**

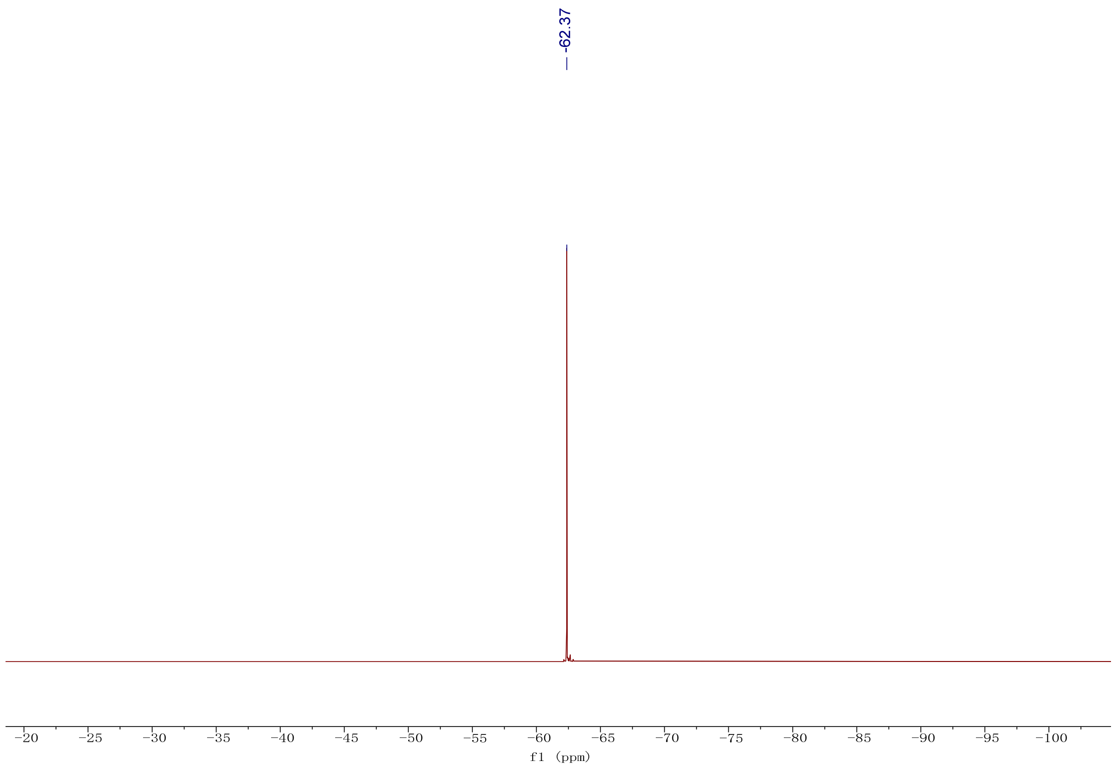

Supplementary Figure 163. ^{19}F NMR (376 MHz, $CDCl_3$) of **4n**

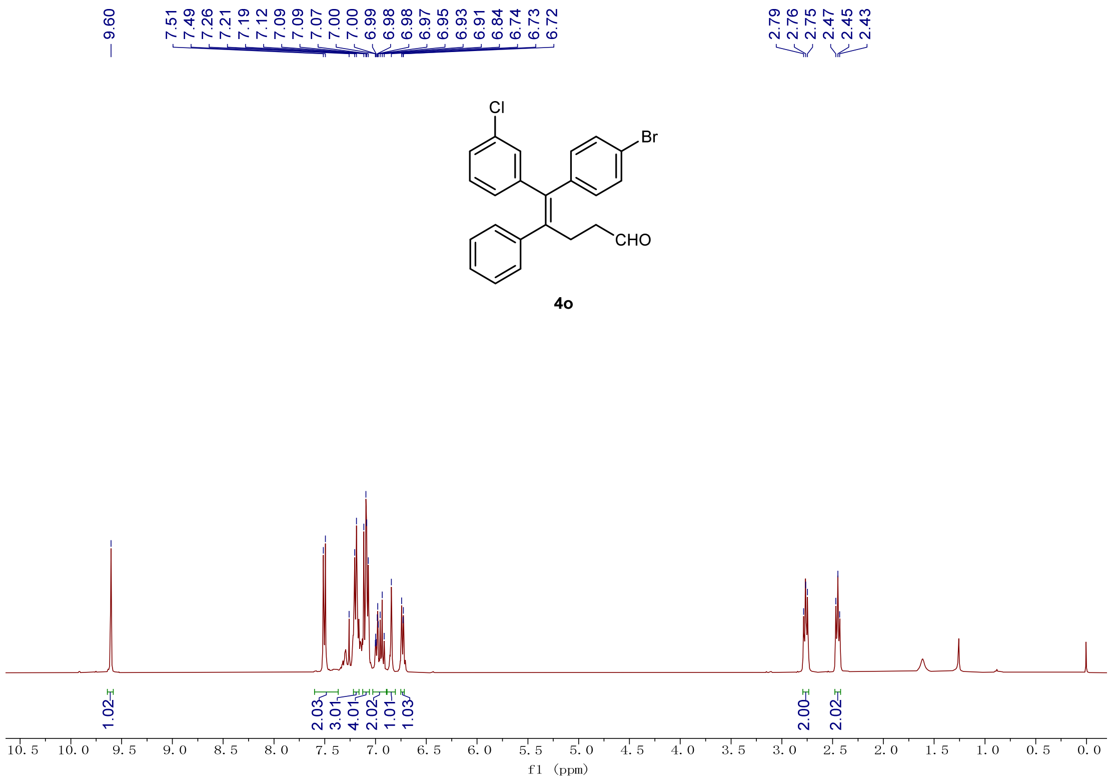

Supplementary Figure 164. ^{1}H NMR (400 MHz, $CDCl_3$) of **4o**

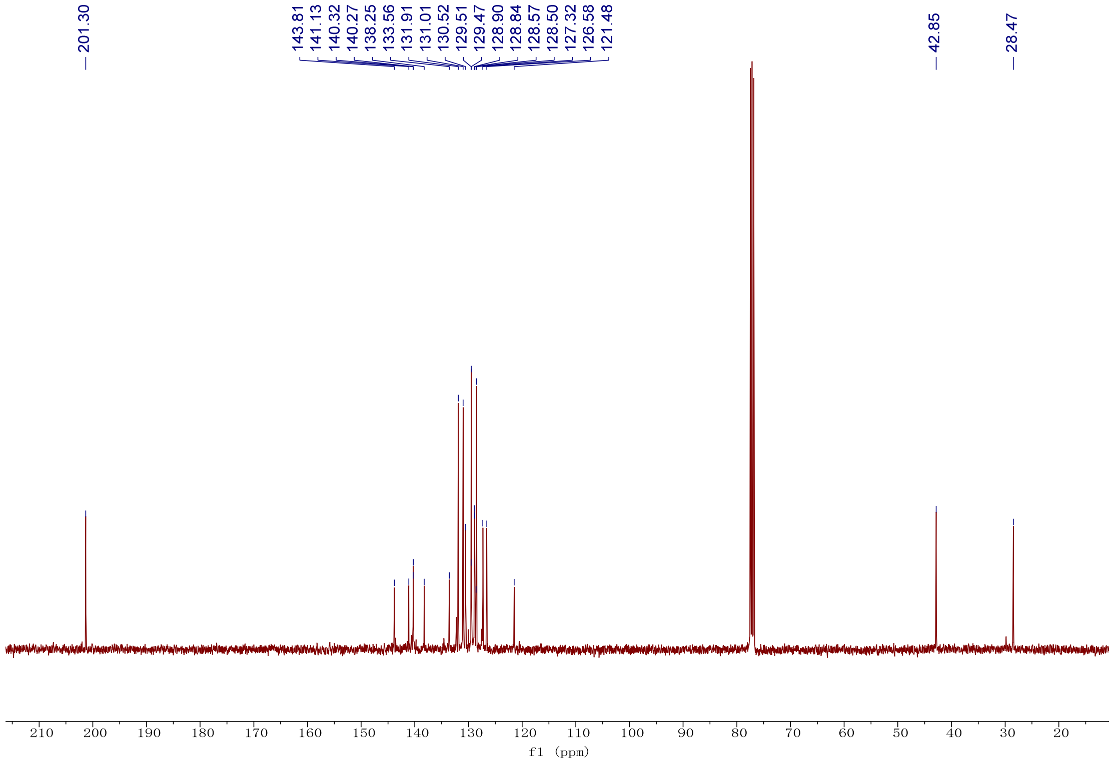

Supplementary Figure 165. ^{13}C NMR (101 MHz, $CDCl_3$) of **4o**

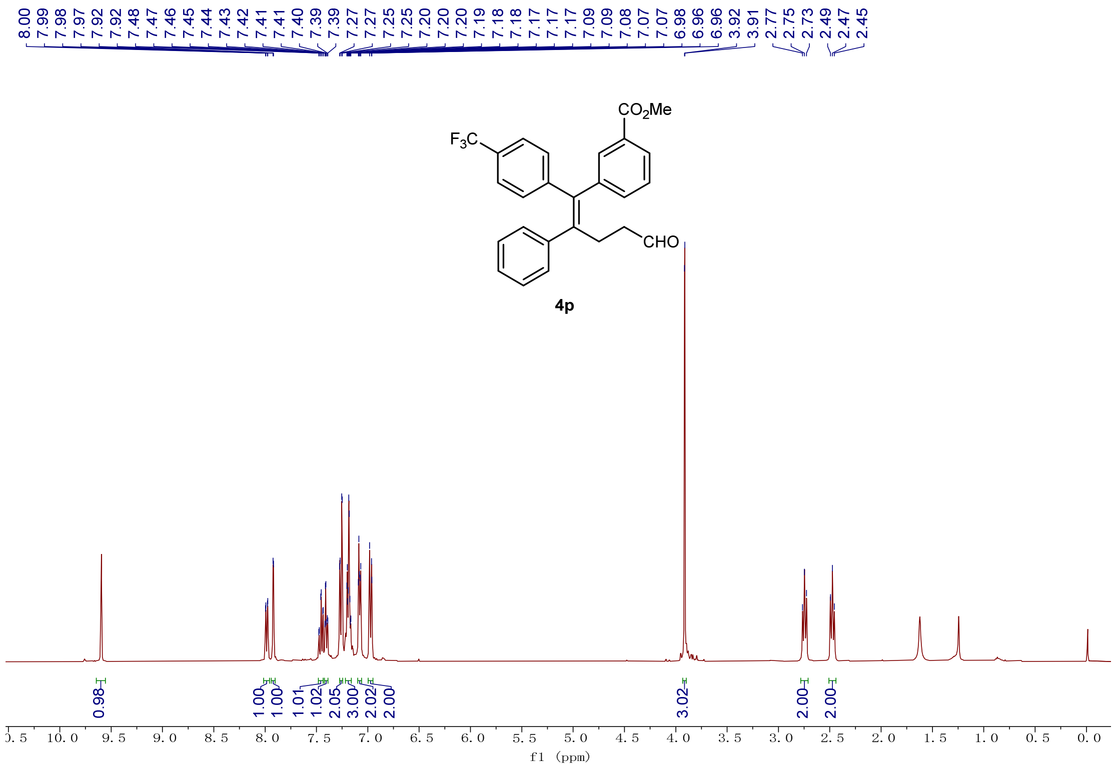

Supplementary Figure 166. ^{1}H NMR (400 MHz, $CDCl_3$) of **4p**

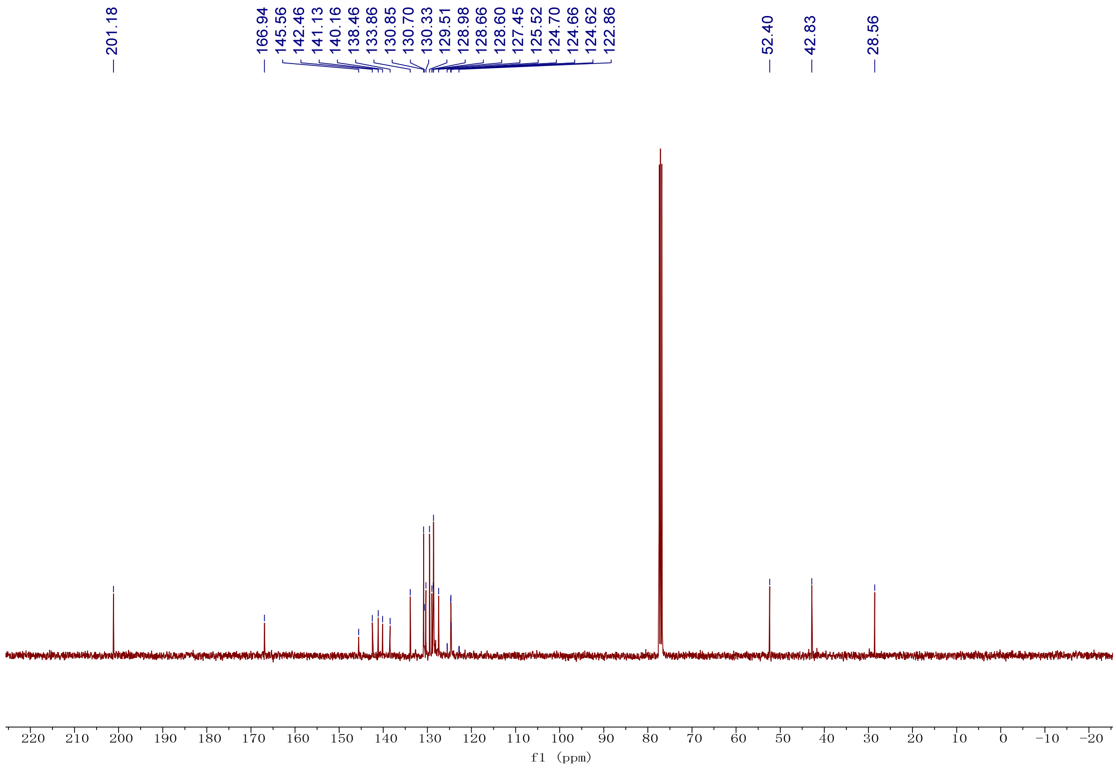

Supplementary Figure 167. ^{13}C NMR (101 MHz, $CDCl_3$) of **4p**

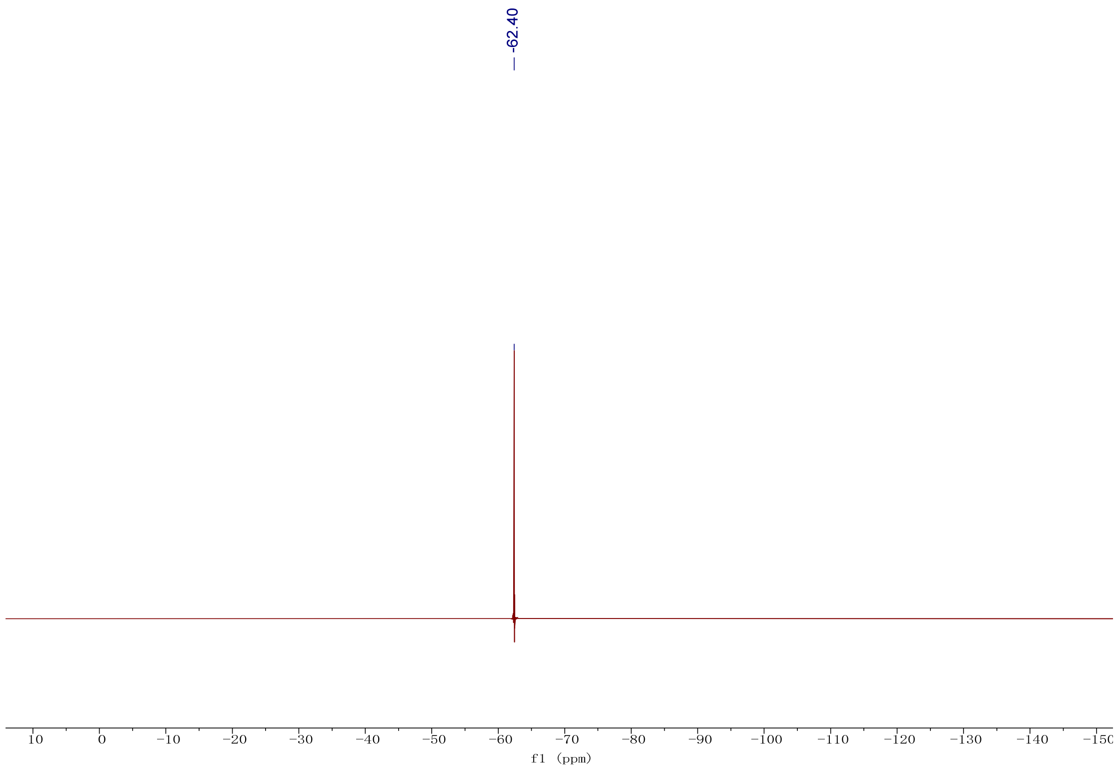

Supplementary Figure 168. ^{19}F NMR (376 MHz, $CDCl_3$) of **4p**

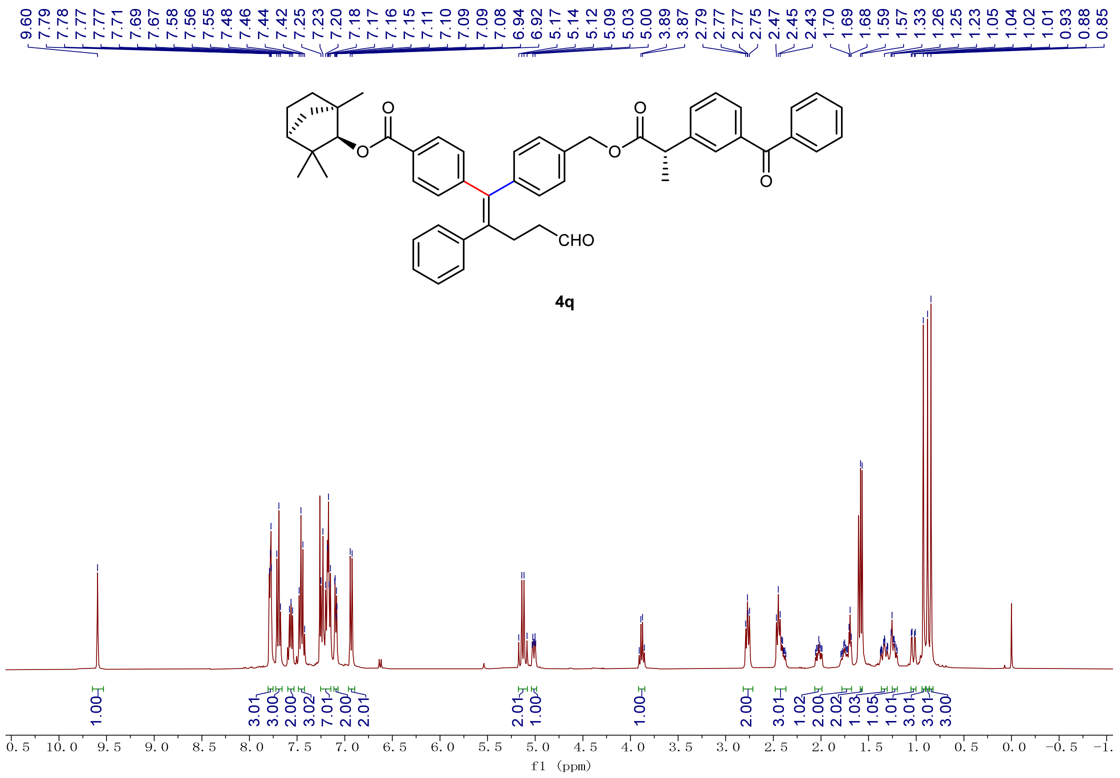

Supplementary Figure 169. ^{1}H NMR (400 MHz, $CDCl_3$) of **4q**

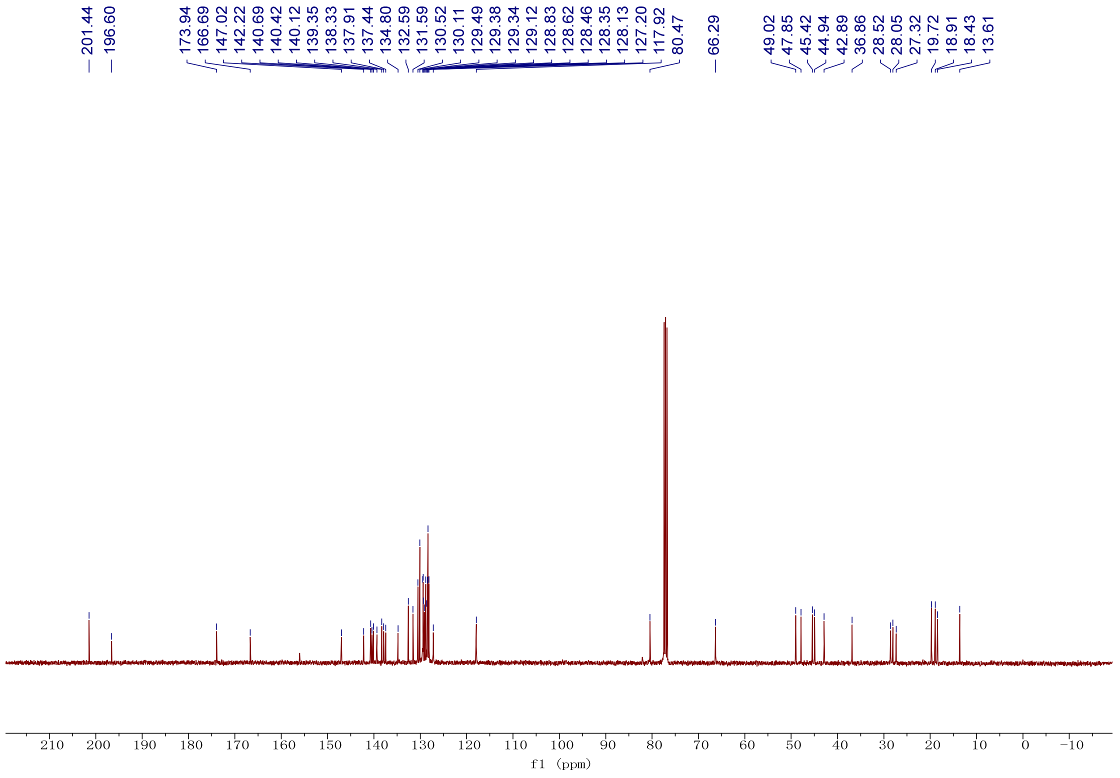

Supplementary Figure 170. ^{13}C NMR (101 MHz, $CDCl_3$) of **4q**

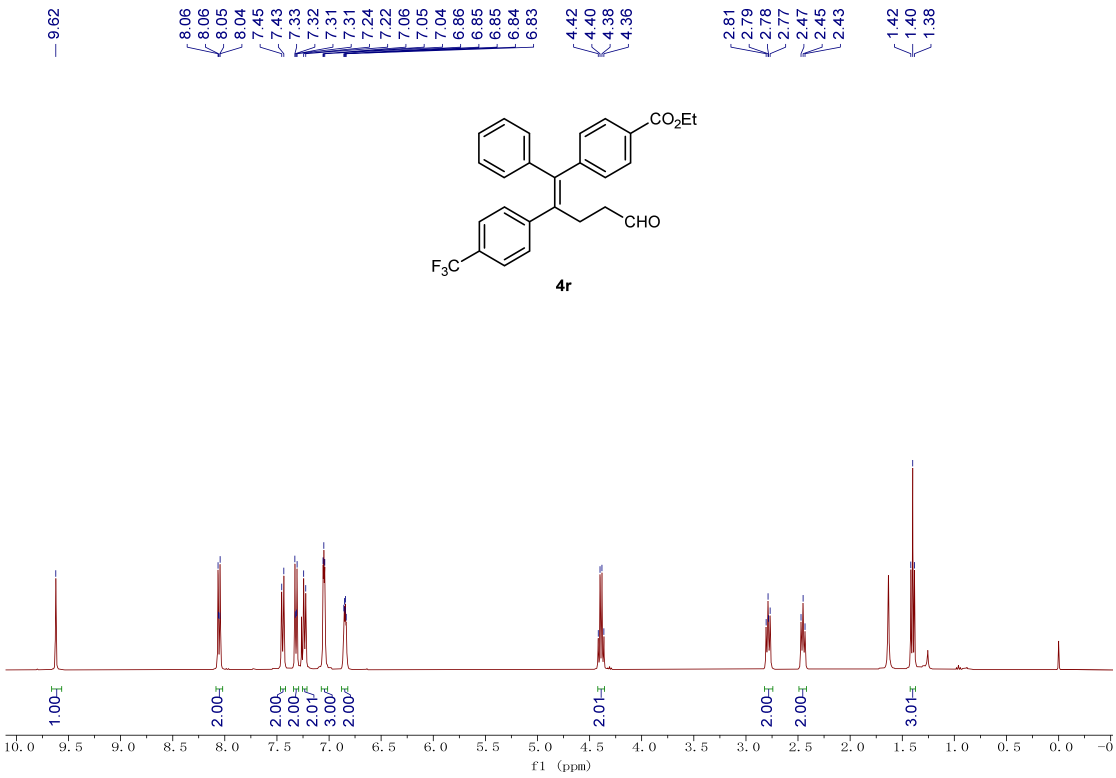

Supplementary Figure 171. ^{1}H NMR (400 MHz, $CDCl_3$) of **4r**

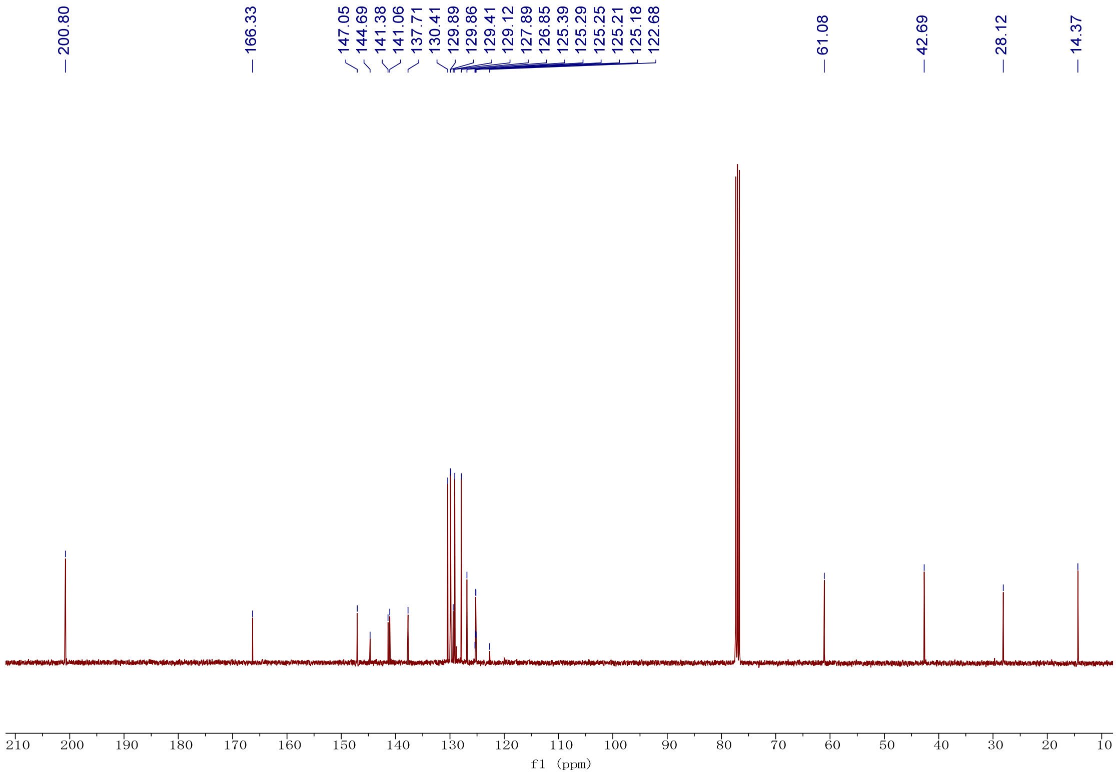

Supplementary Figure 172. ^{13}C NMR (101 MHz, $CDCl_3$) of **4r**

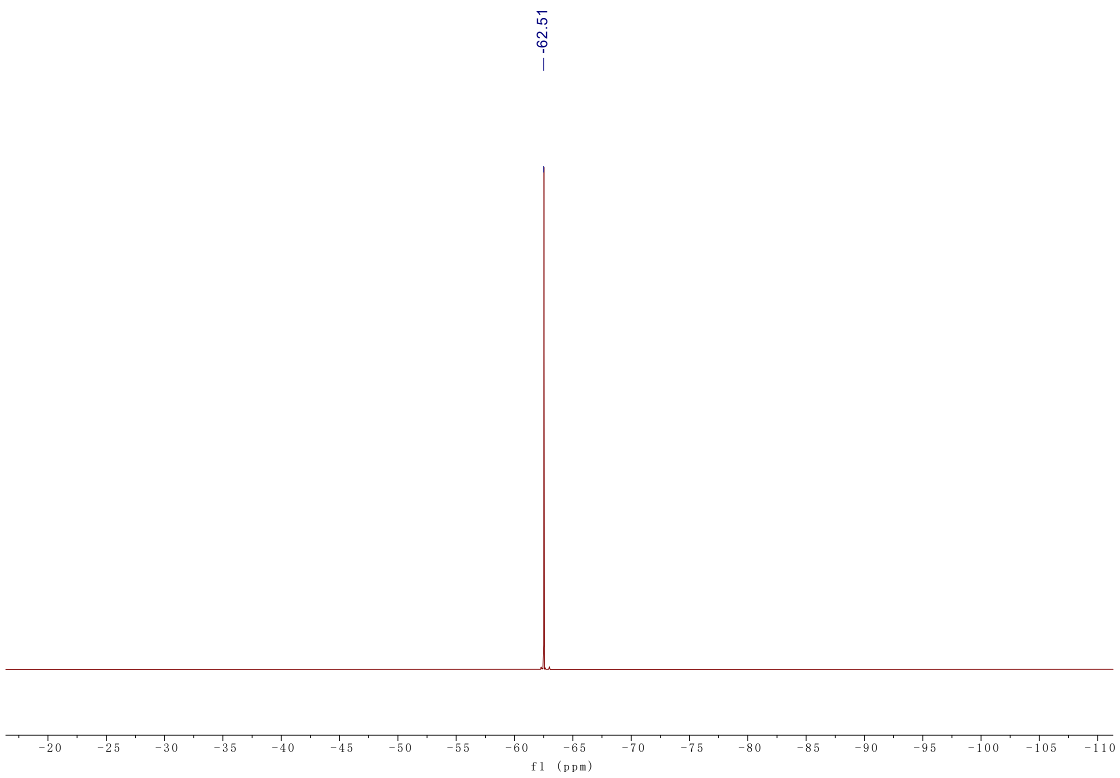

Supplementary Figure 173. ^{19}F NMR (376 MHz, $CDCl_3$) of **4r**

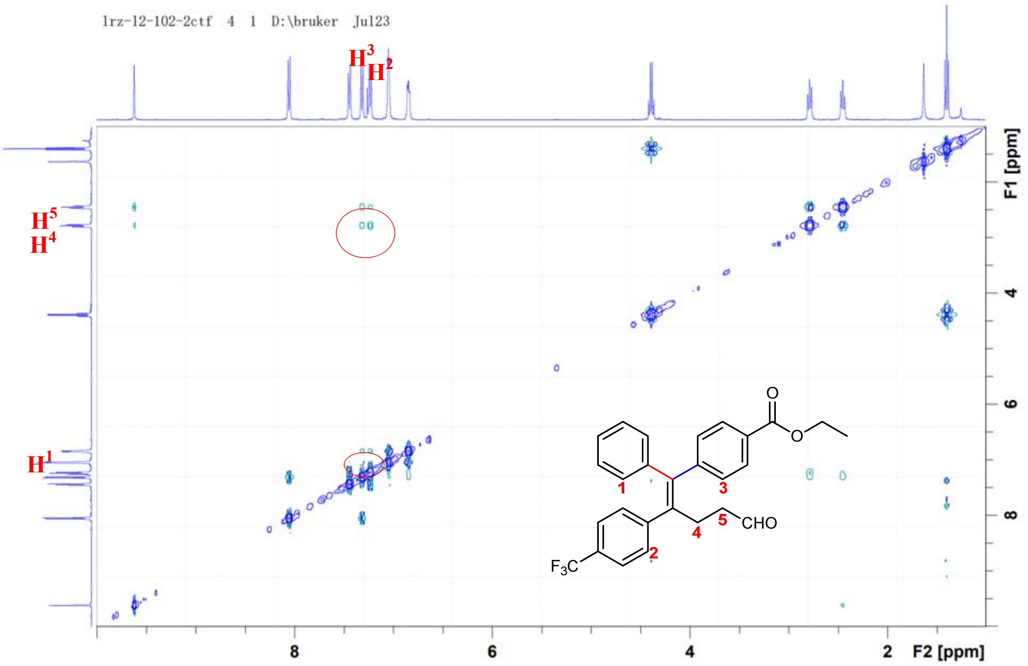

Supplementary Figure 174. NOESY spectrum of **4r**

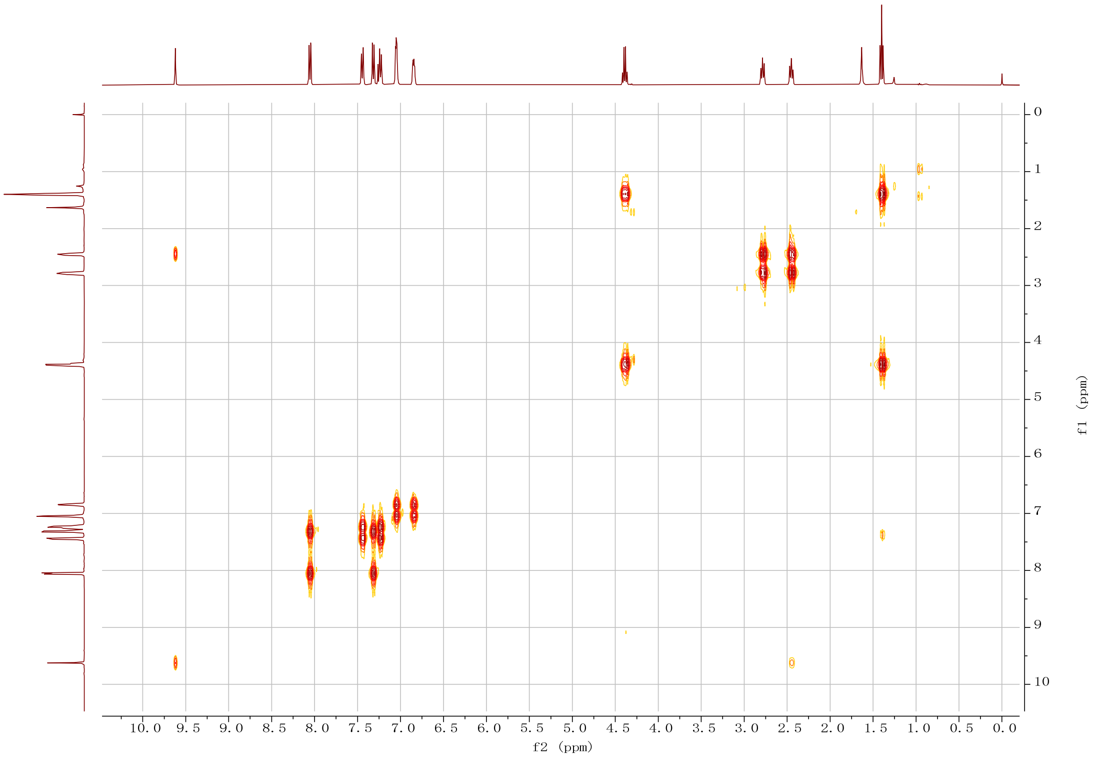

Supplementary Figure 175. COSY spectrum of **4r**

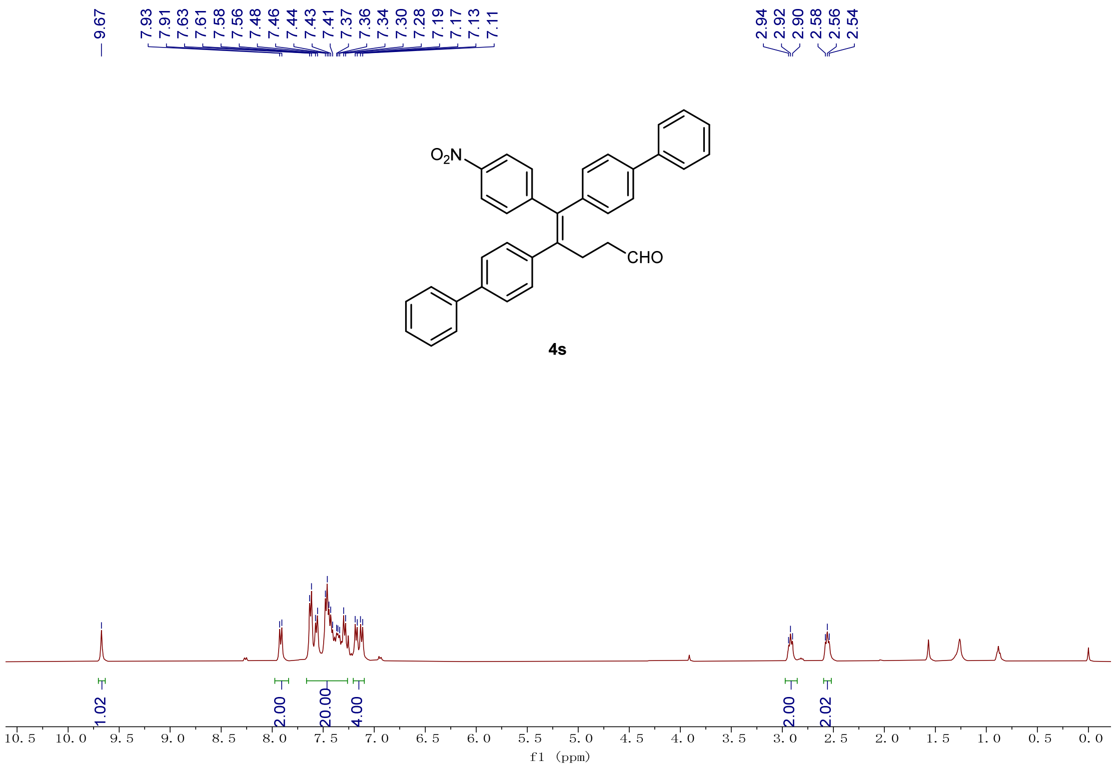

Supplementary Figure 176. ^{1}H NMR (400 MHz, $CDCl_3$) of **4s**

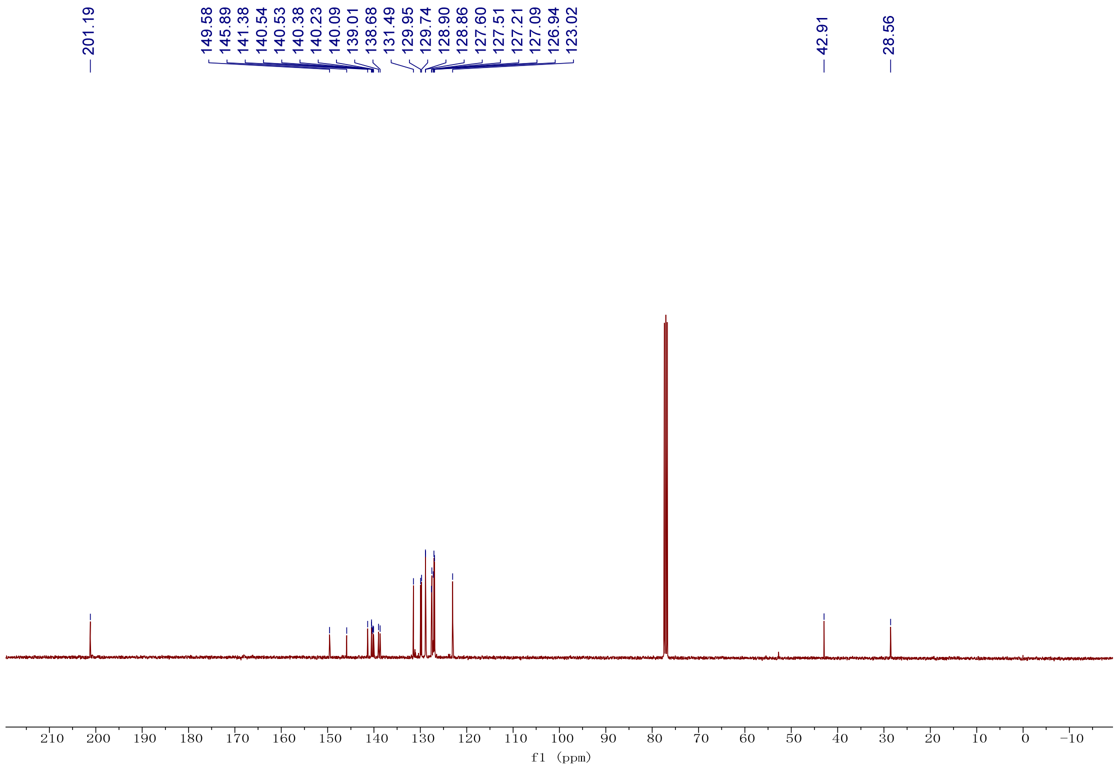

Supplementary Figure 177. ^{13}C NMR (101 MHz, $CDCl_3$) of **4s**

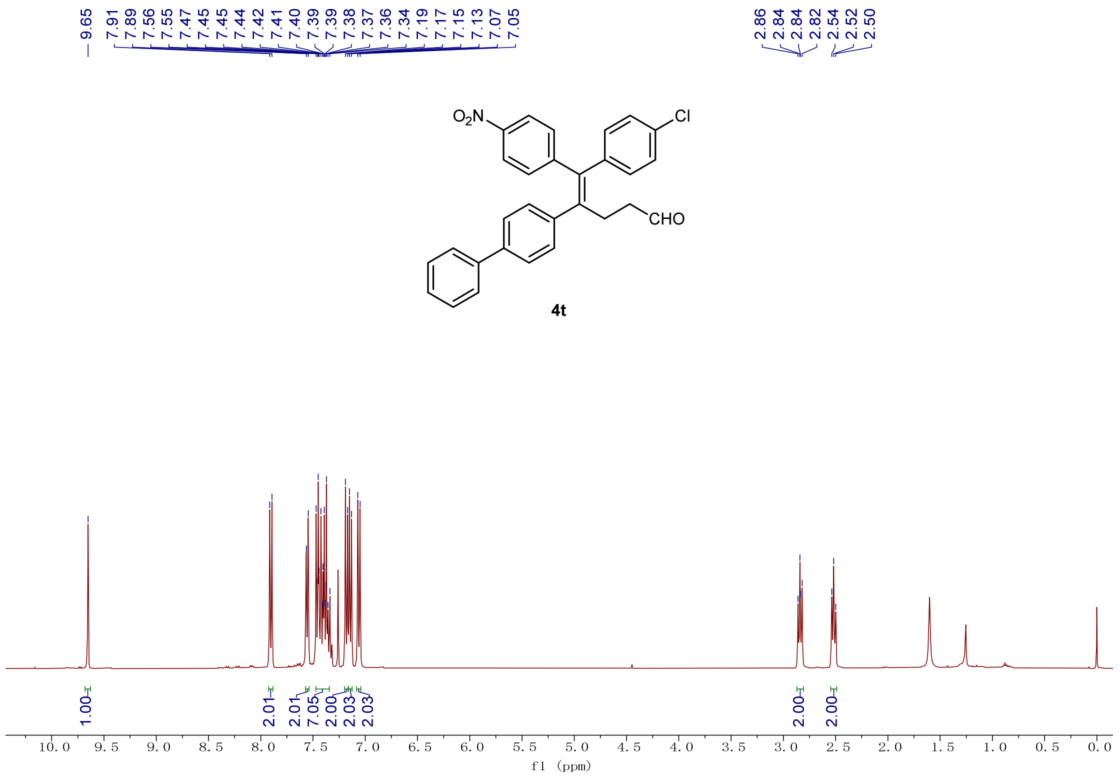

Supplementary Figure 178. ^{1}H NMR (400 MHz, $CDCl_3$) of **4t**

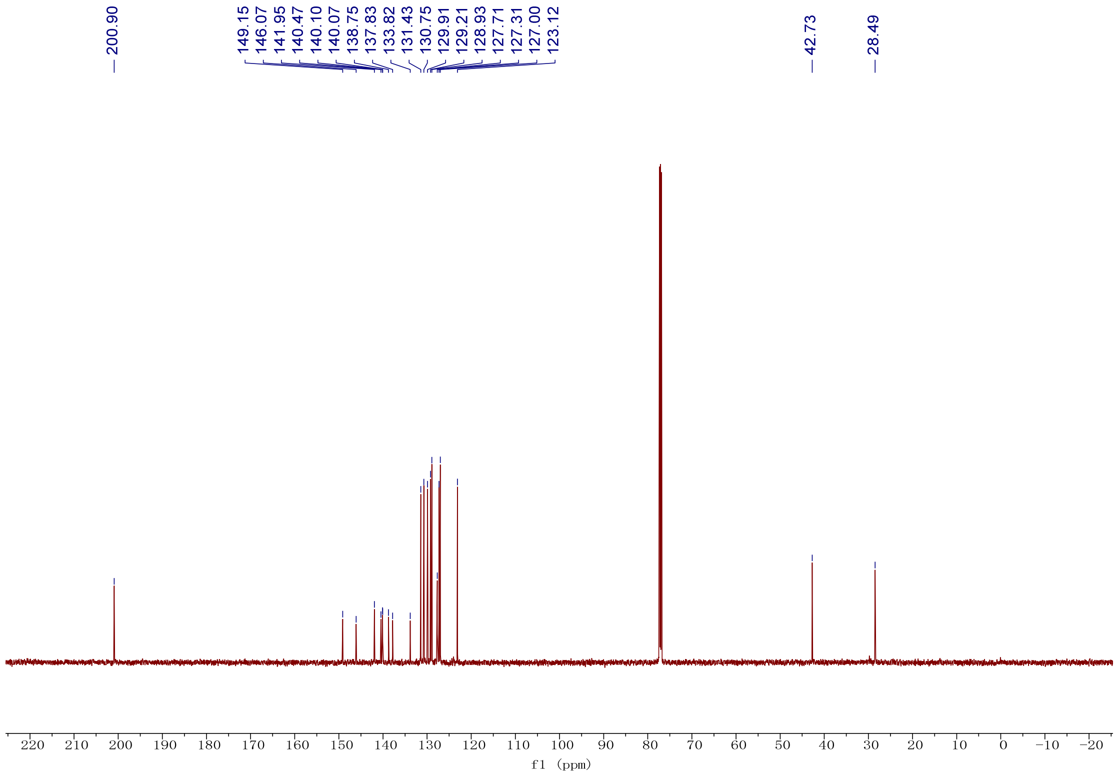

Supplementary Figure 179. ^{13}C NMR (101 MHz, $CDCl_3$) of **4t**

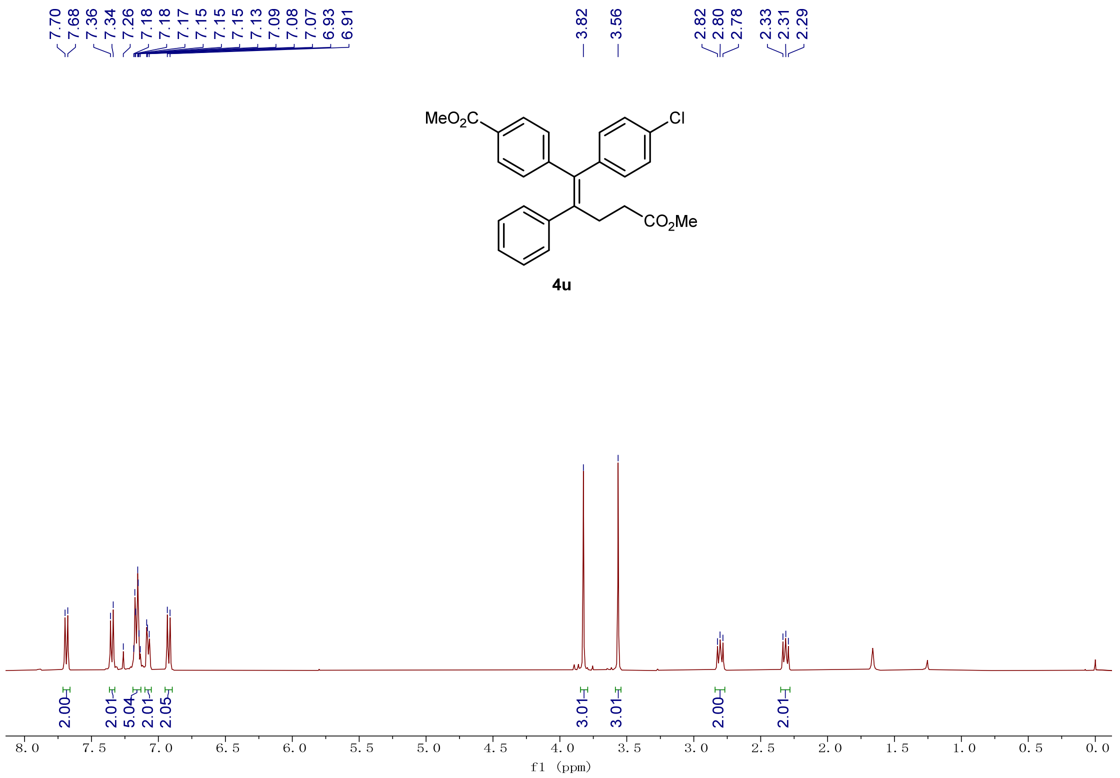

Supplementary Figure 180. ^{1}H NMR (400 MHz, $CDCl_3$) of **4u**

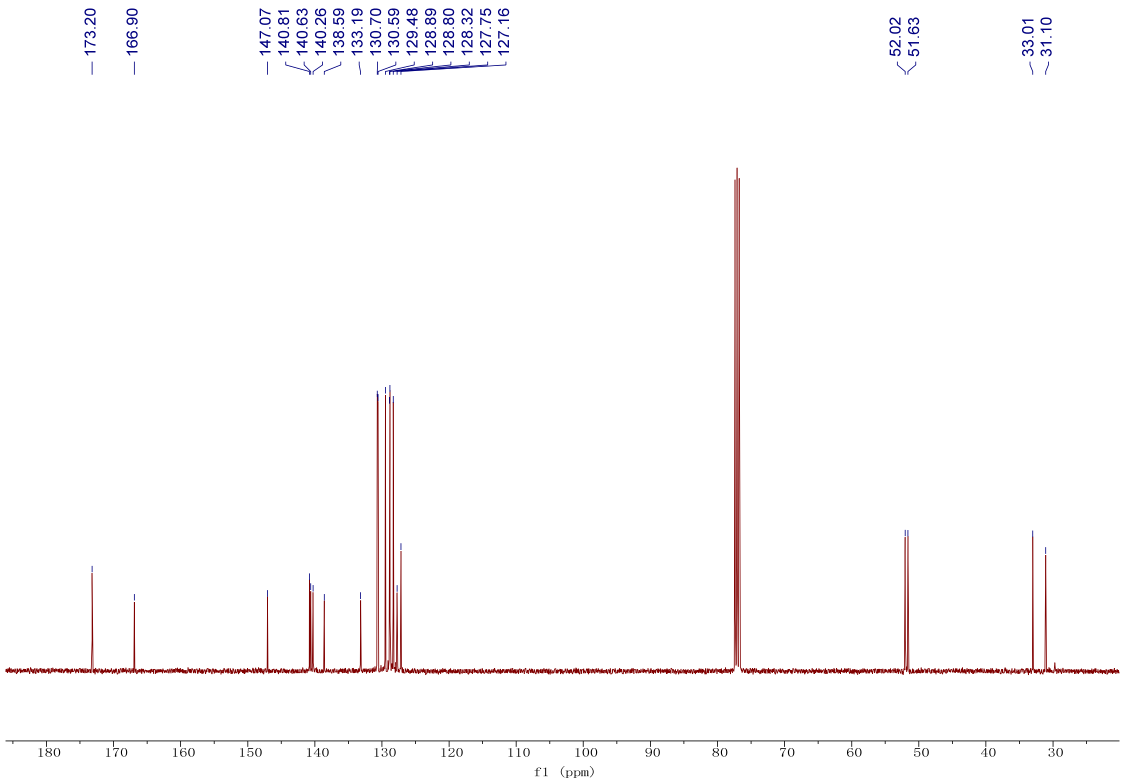

Supplementary Figure 181. ^{13}C NMR (101 MHz, $CDCl_3$) of **4u**

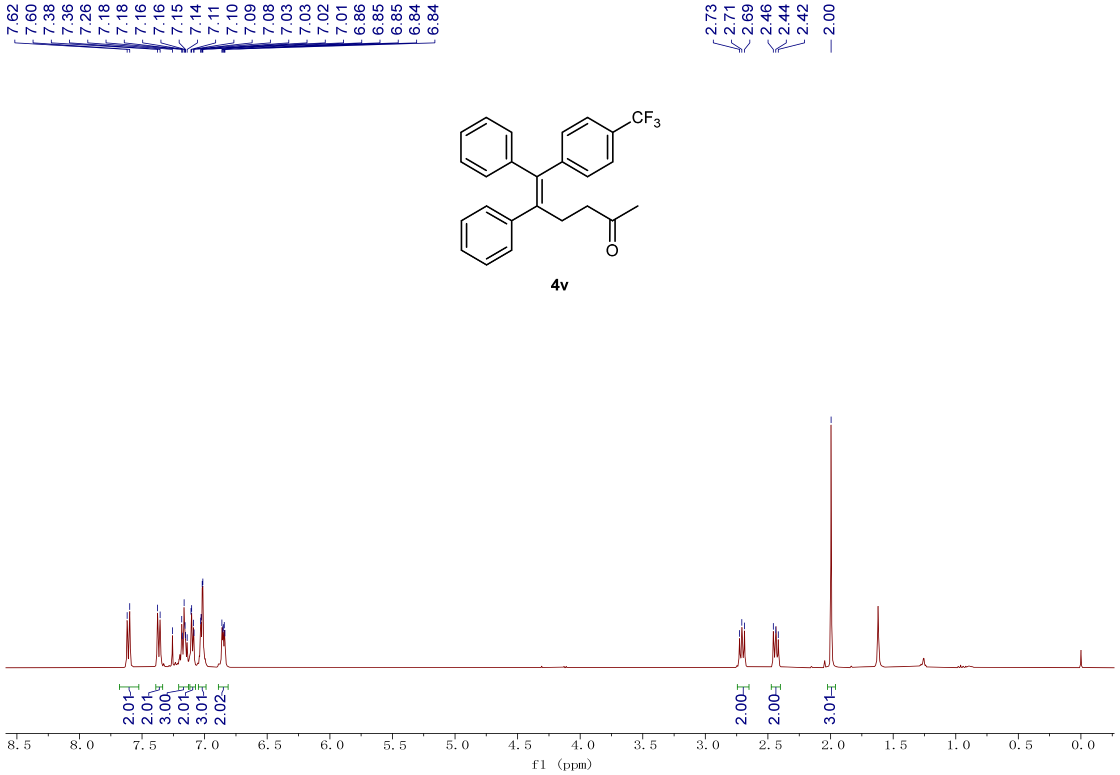

Supplementary Figure 182. ^{1}H NMR (400 MHz, $CDCl_3$) of **4v**

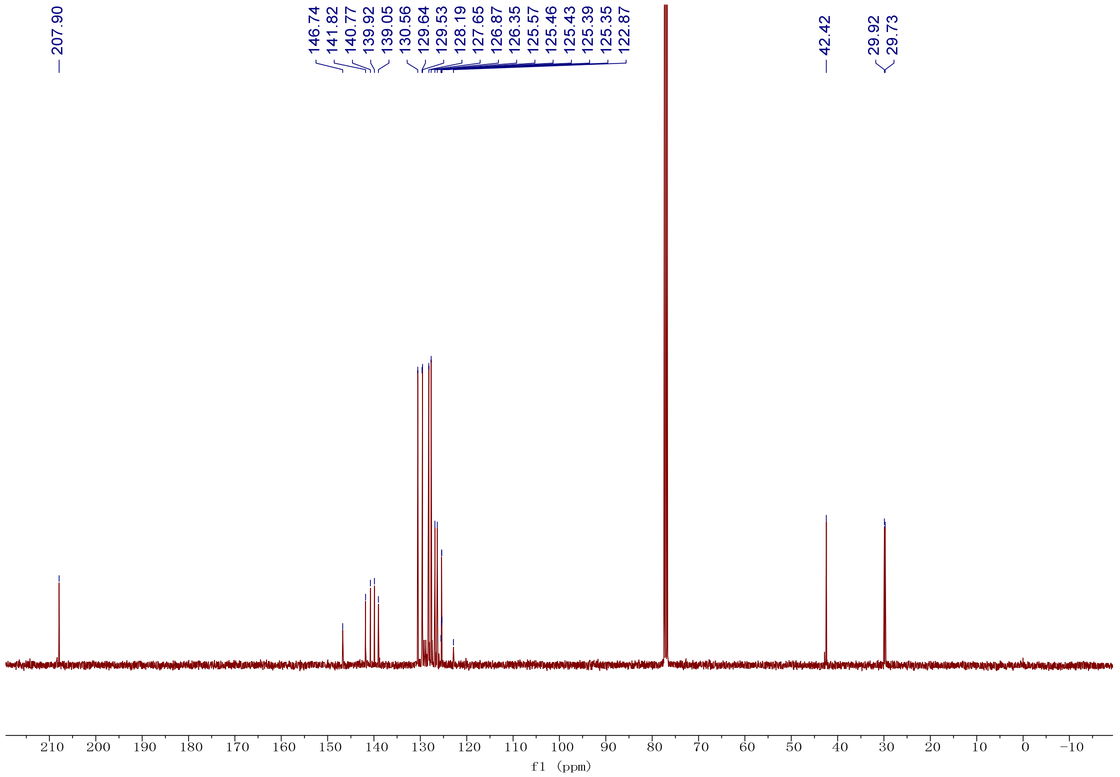

Supplementary Figure 183. ^{13}C NMR (101 MHz, $CDCl_3$) of **4v**

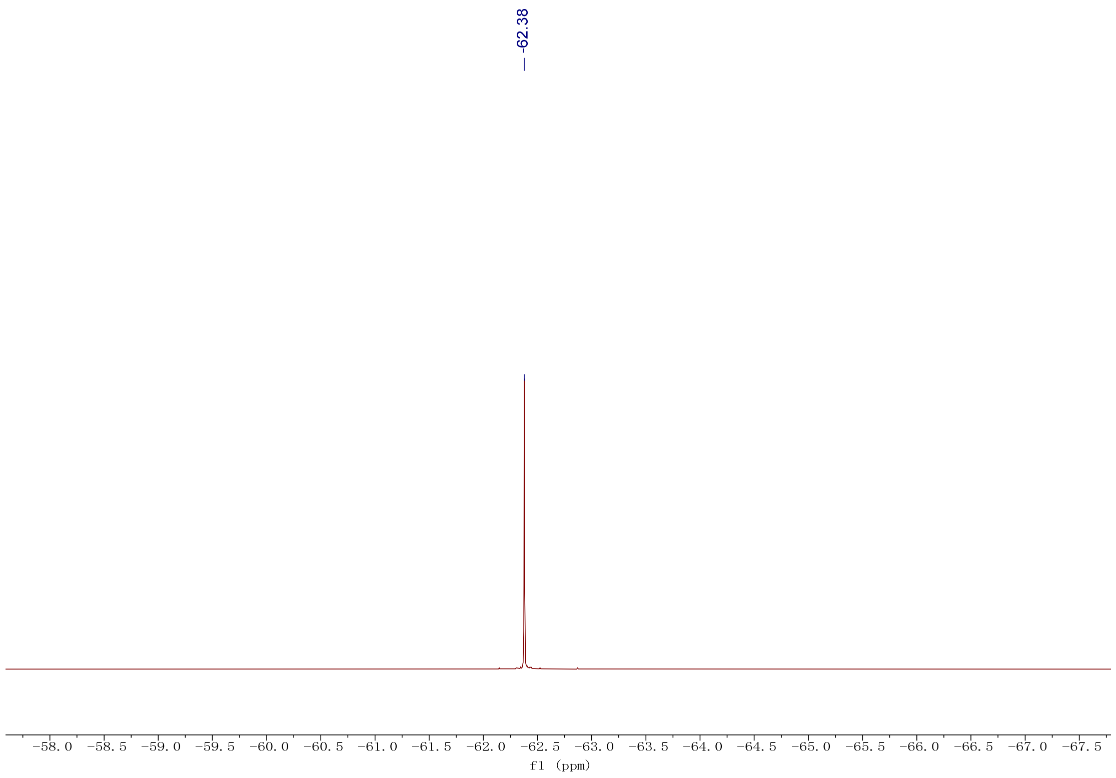

Supplementary Figure 184. ^{19}F NMR (376 MHz, $CDCl_3$) of **4v**

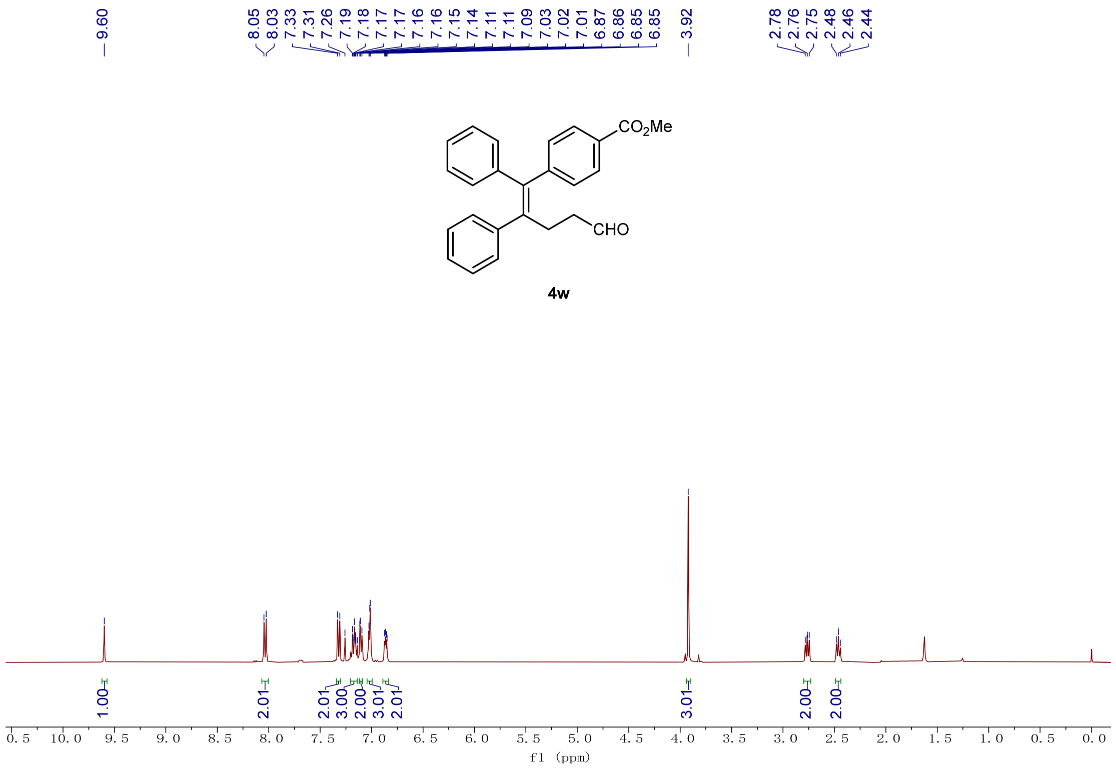

Supplementary Figure 185. ^{1}H NMR (400 MHz, $CDCl_3$) of **4w**

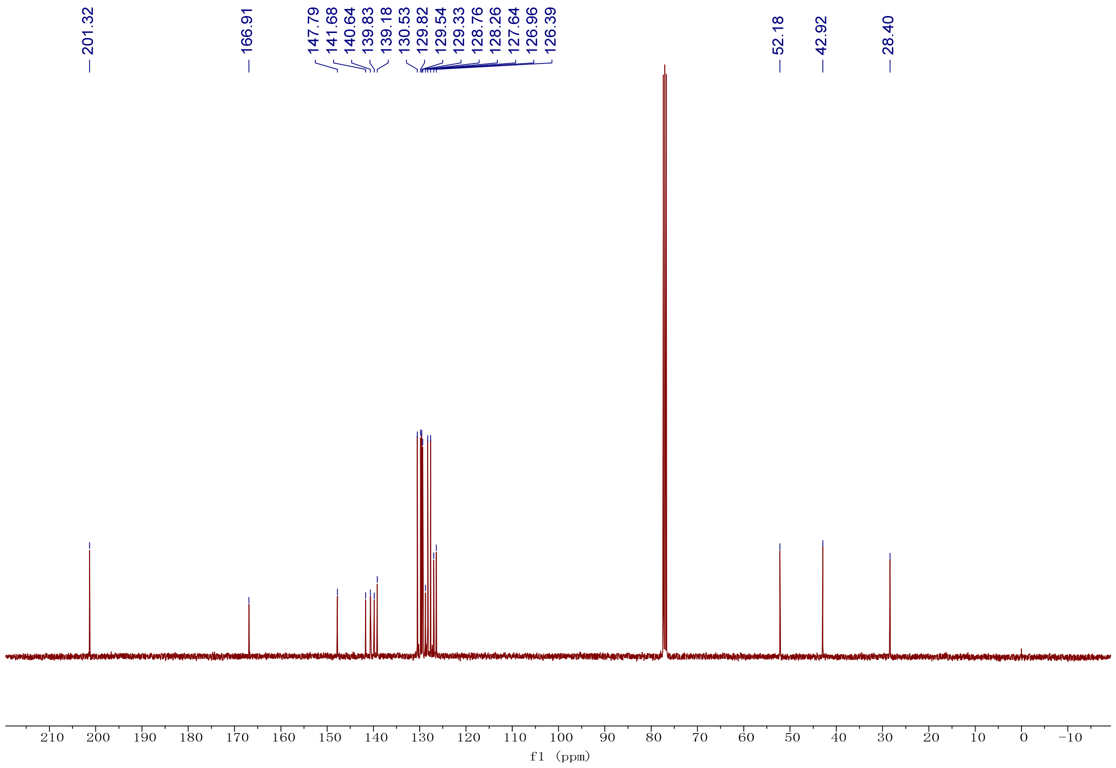

Supplementary Figure 186. ^{13}C NMR (101 MHz, $CDCl_3$) of **4w**

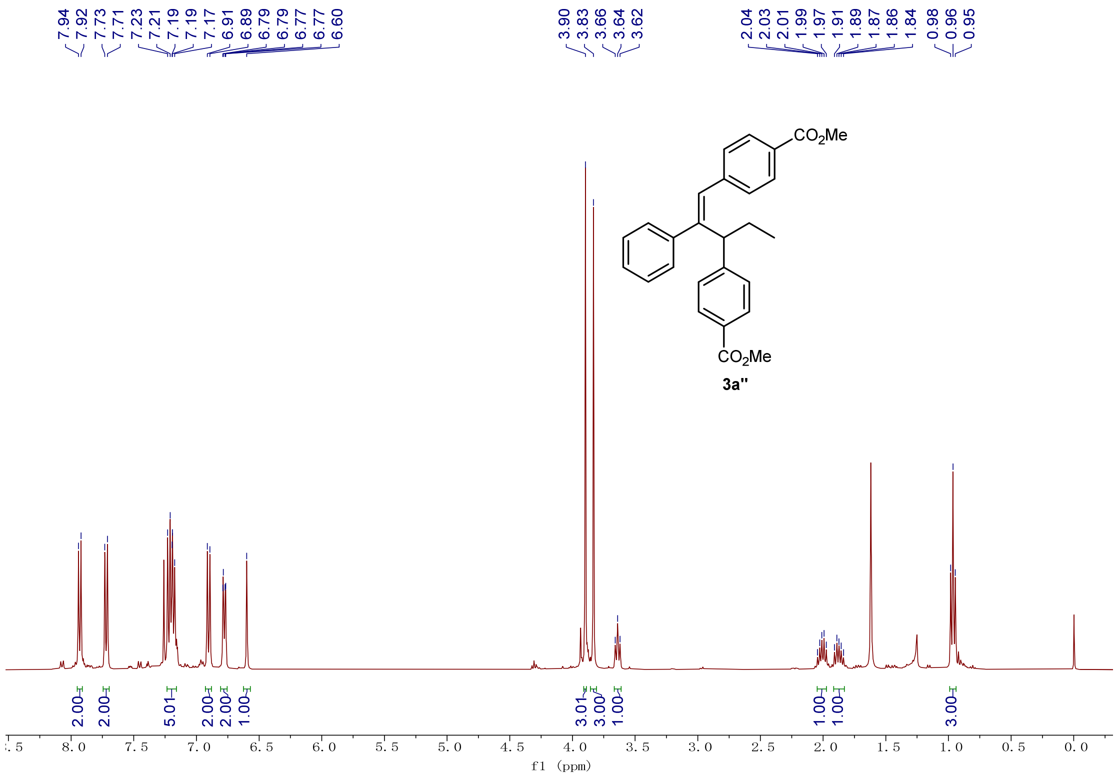

Supplementary Figure 187. ^{1}H NMR (400 MHz, $CDCl_3$) of **3a''**

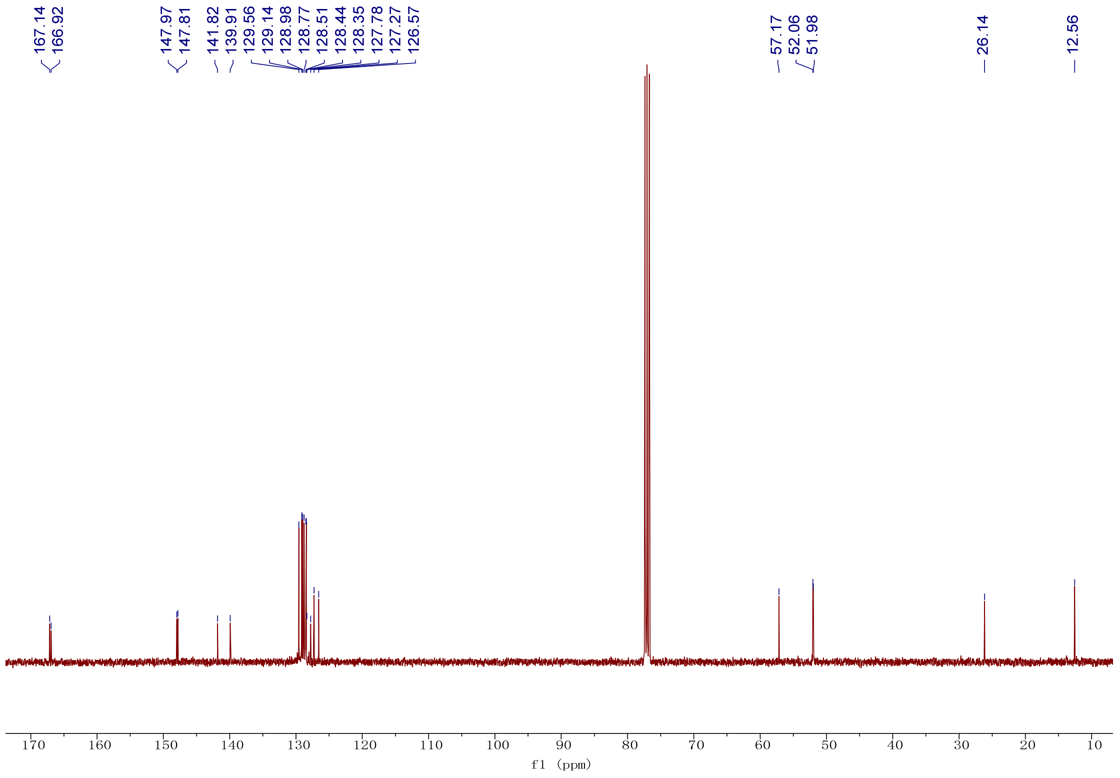

Supplementary Figure 188. ^{13}C NMR (101 MHz, $CDCl_3$) of **3a''**

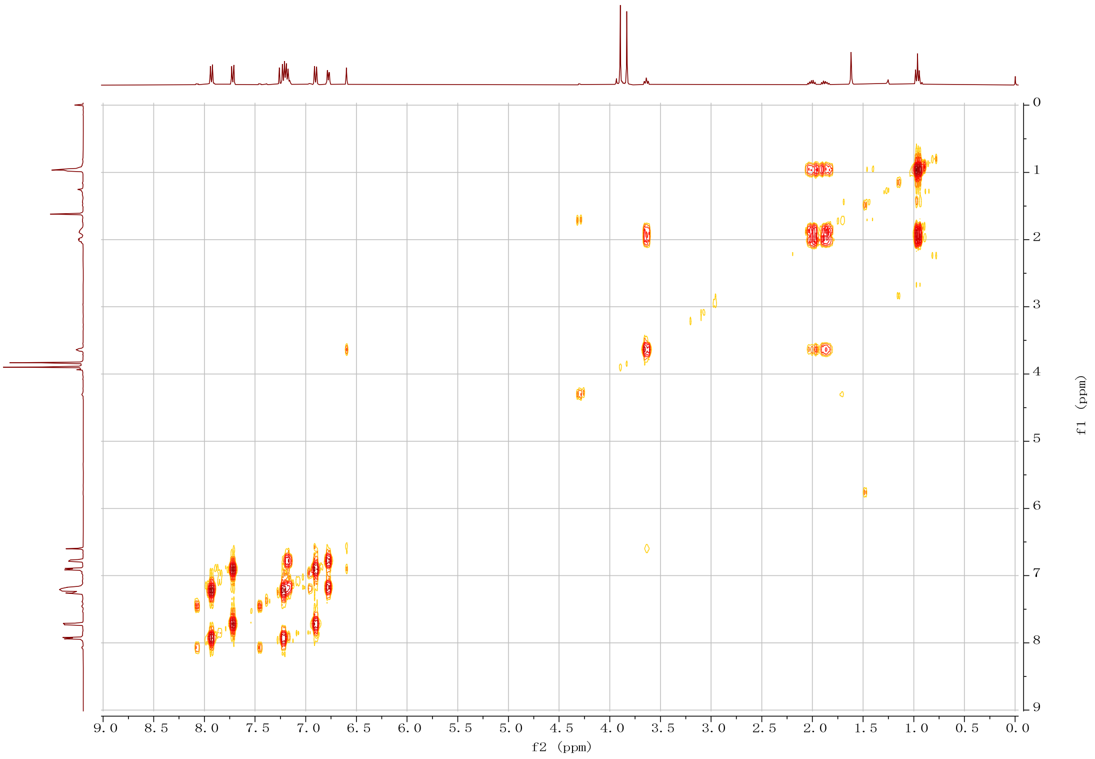

Supplementary Figure 189. COSY spectrum of **3a''**

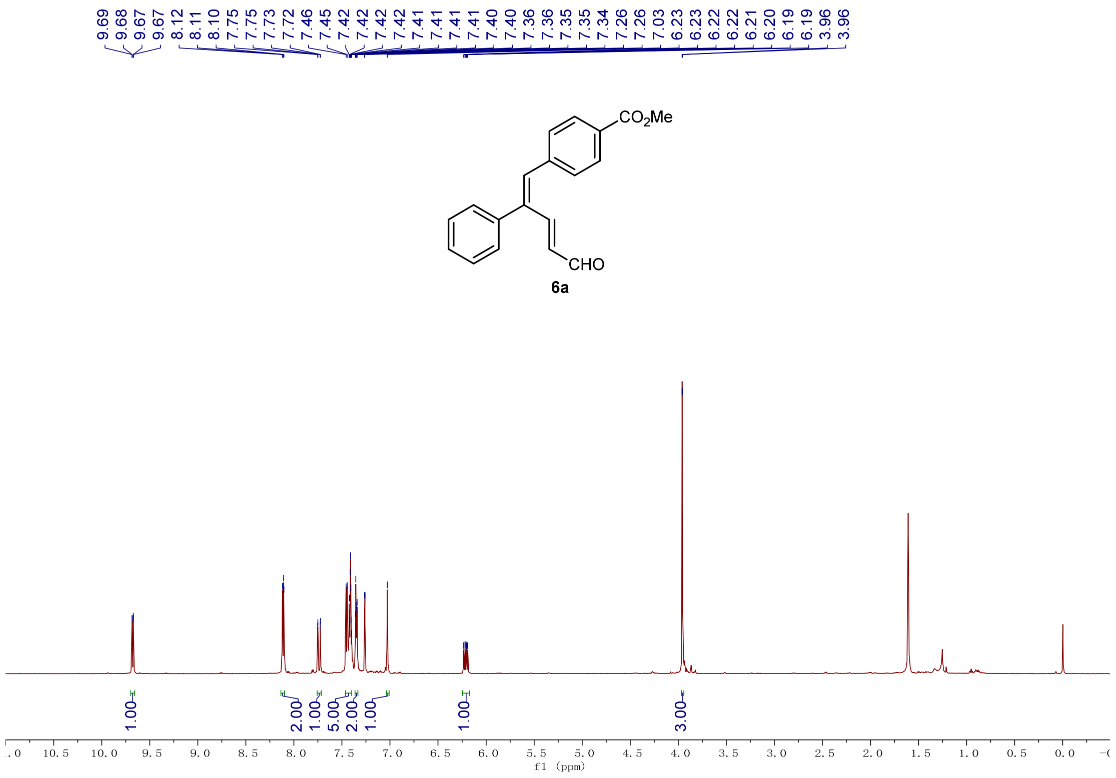

Supplementary Figure 190. ^{1}H NMR (600 MHz, $CDCl_3$) of **6a**

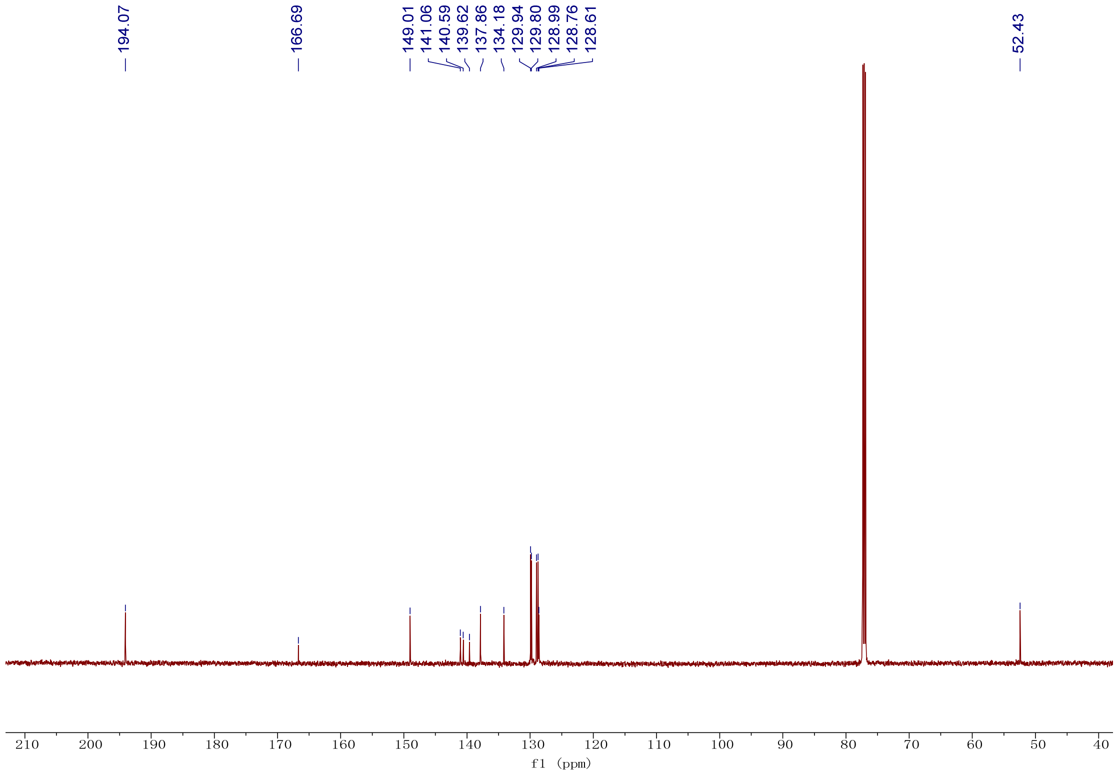

Supplementary Figure 191. ^{13}C NMR (151 MHz, $CDCl_3$) of **6a**

4. Supplementary References

1. Wang, X. *et al.* Access to saturated oxygen heterocycles and lactones via electrochemical sulfonylative oxycyclization of alkenes with sulfonyl hydrazides. *J. Org. Chem.* **88**, 2505–2520 (2023).

2. Takemiya, A. & Hartwig, J. F. Rhodium-catalyzed intramolecular, anti-Markovnikov hydroamination. Synthesis of 3-arylpiperidines. *J. Am. Chem. Soc.* **128**, 6042–6043 (2006).

3. Janssen-Müller, D., Schedler, M., Fleige, M., Daniliuc, C. G. & Glorius, F. Enantioselective intramolecular hydroacylation of unactivated alkenes: an NHC-catalyzed robust and versatile formation of cyclic chiral ketones. *Angew. Chem. Int. Ed.* **54**, 12492–12496 (2015).

4. Hoffman, T. J. & Carreira, E. M. Catalytic asymmetric intramolecular hydroacylation with rhodium/phosphoramidite-alkene ligand complexes. *Angew. Chem. Int. Ed.* **50**, 10670–10674 (2011).

5. Schroeder, M., Mathys, M., Ehrensperger, N. & Büchel, M. *γ*-Unsaturated aldehydes as potential *Lilial* replacers. *Chem. Biodivers.* **11**, 1651–1673 (2014).

6. Zhang, S. *et al.* Cobalt(II)-catalyzed stereoselective olefin isomerization: facile access to acyclic trisubstituted alkenes. *J. Am. Chem. Soc.* **142**, 8910–8917 (2020).

7. Hemric, B. N., Shen, K. & Wang, Q. Copper-catalyzed amino lactonization and amino oxygenation of alkenes using *O*-benzoylhydroxylamines. *J. Am. Chem. Soc.* **138**, 5813–5816 (2016).

8. Koser, L., Lechner, V. M. & Bach, T. Biomimetic total synthesis of enterocin. *Angew. Chem. Int. Ed.* **60**, 20269–20273 (2021).

9. Gesmundo, N. J. & Nicewicz, D. A. Cyclization-endoperoxidation cascade reactions of dienes mediated by a pyrylium photoredox catalyst. *Beilstein J. Org. Chem.* **10**, 1272–1281 (2014).

10. Munnuri, S. & Falck, J. R. Directed, remote dirhodium C(sp^3)–H functionalization, desaturative annulation, and desaturation. *J. Am. Chem. Soc.* **144**, 17989–17998 (2022).

11. Page, C. G. *et al.* Quaternary charge-transfer complex enables photoenzymatic intermolecular hydroalkylation of olefins. *J. Am. Chem. Soc.* **143**, 97–102 (2021).

12. Huang, Y., Du, Y. & Su, W. Convenient and flexible syntheses of *gem*-dimethyl carboxylic triggers via mono-selective *β*-C(sp3)–H arylation of pivalic acid with *ortho*-substituted aryl iodides. *Org. Chem. Front.* **9**, 3293–3300 (2022).
13. Che, Y.-Y. *et al.* Palladium-catalyzed electrophilic functionalization of pyridine derivatives through phosphonium salts. *Angew. Chem. Int. Ed.* **59**, 16414–16419 (2020).
14. Loup, J., Larin, E. M. & Lautens, M. Iron-catalyzed reductive cyclization by hydromagnesiation: a modular strategy towards *N*-heterocycles. *Angew. Chem. Int. Ed.* **60**, 22345–22351 (2021).
